# Meroterpenoids From *Ganoderma lucidum* Mushrooms and Their Biological Roles in Insulin Resistance and Triple-Negative Breast Cancer

**DOI:** 10.3389/fchem.2021.772740

**Published:** 2021-11-03

**Authors:** Jiao-Jiao Zhang, Dai-Wei Wang, Dan Cai, Qing Lu, Yong-Xian Cheng

**Affiliations:** ^1^ Institute for Inheritance-Based Innovation of Chinese Medicine, School of Pharmaceutical Sciences, Health Science Center, Shenzhen University, Shenzhen, China; ^2^ Institute of Microscale Optoelectronics, Shenzhen University, Shenzhen, China; ^3^ Guangdong Key Laboratory for Functional Substances in Medicinal Edible Resources and Healthcare Products, School of Life Sciences and Food Engineering, Hanshan Normal University, Chaozhou, China

**Keywords:** *Ganoderma lucidum*, meroterpenoids, Akt phosphorylation, AMPK phosphorylation, glucose uptake, cell migration

## Abstract

*Ganoderma* fungi as popular raw materials of numerous functional foods have been extensively investigated. In this study, five pairs of meroterpenoid enantiomers beyond well-known triterpenoids and polysaccharides, dayaolingzhiols I−M (**1**–**5**), were characterized from *Ganoderma lucidum.* Their structures were identified using spectroscopic and computational methods. Structurally, compound **1** features a novel dioxabicyclo[2.2.2]octan-3-one motif in the side chain. Ethnoknowledge-derived biological evaluation found that (+)-**5** could activate Akt and AMPK phosphorylation in insulin-stimulated C2C12 cells, and (+)-**5** could activate glucose uptake dose dependently in C2C12 cells. Furthermore, we found that (+)-**1** (+)-**4**, and (–)-**4** could significantly inhibit cell migration of the MDA-MB-231 cell line, of which (+)-**4** showed significant inhibitory effects against cell migration of the MDA-MB-231 cell line in a dose-dependent manner. These findings revealed the meroterpenoidal composition of *G. lucidum* and its roles in the prevention of chronic diseases such as diabetes mellitus and triple-negative breast cancer.

## Introduction

Insulin resistance (IR) is defined as an impaired biological response to insulin stimulation of target tissues mainly including the liver, muscle, and adipose tissue. Insulin resistance syndrome, also known as metabolic syndrome, is associated with a broad spectrum of diseases such as obesity, hyperglycemia, hypertension, dyslipidemia, non-alcoholic fatty liver disease, cardiovascular disease, polycystic ovarian syndrome, and type 2 diabetes mellitus (Allahbadia and Merchant, 2008; Willard et al., 2016; Cortés-Rojo et al., 2020; García-Carretero et al., 2021). Insulin resistance is mainly related to excess body fat and also genetic causes, which affects as many as one in three Americans and becomes a tremendous burden for the healthcare system of the United States (Balasubramanyam, 2006; McCarthy, 2014; Sharma et al., 2019; Rajesh and Sarkar, 2021). Now, the incidence of insulin resistance is growing at an alarming speed, and it is actually becoming a non-ignorable public concern worldwide. Accumulating evidence revealed that the inactivation of Akt and the activation of Foxo1 via inhibiting insulin receptor substrate 1 (IRS1) and insulin receptor substrate 2 (IRS2) might act as the underlying mechanism of metabolic syndromes (Guo, 2014). Targeting the pivotal molecules such as IRS or Akt or Foxo1 in the signaling cascade will therefore be a precise strategy for the intervention of insulin resistance–related diseases (Guo, 2014).

Breast cancer is still the most common cancer among women (Chowdhury et al., 2021; Loibl et al., 2021). The incidence rate accounts for about 30%, and the mortality rate is as high as 15% (Loibl et al., 2021). Triple-negative breast cancer (TNBC) is one of the subgroups of breast cancer which has an incidence of 15–20% and many characteristics such as invasive, resistant, and rapid growth rate (Chen et al., 2021; Chowdhury et al., 2021). Due to its aggressive behavior, metastasis usually occurs in the liver, lungs, and brain (Chen et al., 2021; Chowdhury et al., 2021). Discovering some potential molecules which could inhibit cell migration of TNBC may provide some enlightenment for its treatment.

*Ganoderma* fungi, well-known mushrooms of numerous functional foods, have received long-term attention worldwide. For example, 28785 documents are related to *Ganoderma* when searched using SciFinder till August 29, 2021. The taste and functional components of the *Ganoderma* fungus lead to its applications as food additives, raw materials, dietary supplements and remedies, even in Western countries (Leskosek-Cukalovic et al., 2010; Veljović et al., 2019; Krobthonga and Yingchutrakul, 2020). Polysaccharides and triterpenoids have long been considered the major chemical compositional elements of *Ganoderma*, whereas the other chemical composition remains largely unknown. In recent years, a number of meroterpenoids in *Ganoderma* have been reported (Jiang et al., 2021), representing a new research trend for *Ganoderma* metabolites. Phenol and terpene moieties are considered biologically active, and *Ganoderma* meroterpenoids possess both groups (Lu et al., 2007; Tan et al., 2018). We have focused on the investigation of *Ganoderma* meroterpenoids since 2009. As a result, many structurally intriguing and biologically important meroterpenoids have been characterized by us (Yan et al., 2019; Dai et al., 2020; Qin et al., 2020; Zhang et al., 2020). Due to considerable economic values, *Ganoderma* species have been cultivated in several places of our country. The concept of “one strain many compounds (OSMAC)” has been widely accepted in the related field. *Ganoderma* belongs to a higher fungal family; hence, it is necessary to gain an insight into the chemical profiling of *Ganoderma* meroterpenoids of *Ganoderma lucidum* produced in different locations, despite that much work has been carried out on this fungal species. *G. lucidum*, cultivated at Dayao County of Yunnan Province, is locally dictated to have distinct effects on diabetes, which thus inspired our interest. *G. lucidum* was reported to have antitumor effects, and triterpenoids are generally regarded as its antitumor ingredients. Given the increasing number of *Ganoderma* meroterpenoids being discovered, it is worth being concerned whether they have a role in tumor treatment. As a consequence, this study afforded five pairs of meroterpenoid enantiomers, dayaolingzhiol I−M (**1**–**5**), with biological activities toward insulin resistance and metastasis of TNBC. Herein, we describe their isolation, structure characterization, and biological evaluation.

## Results and Discussion

**FIGURE 1:**
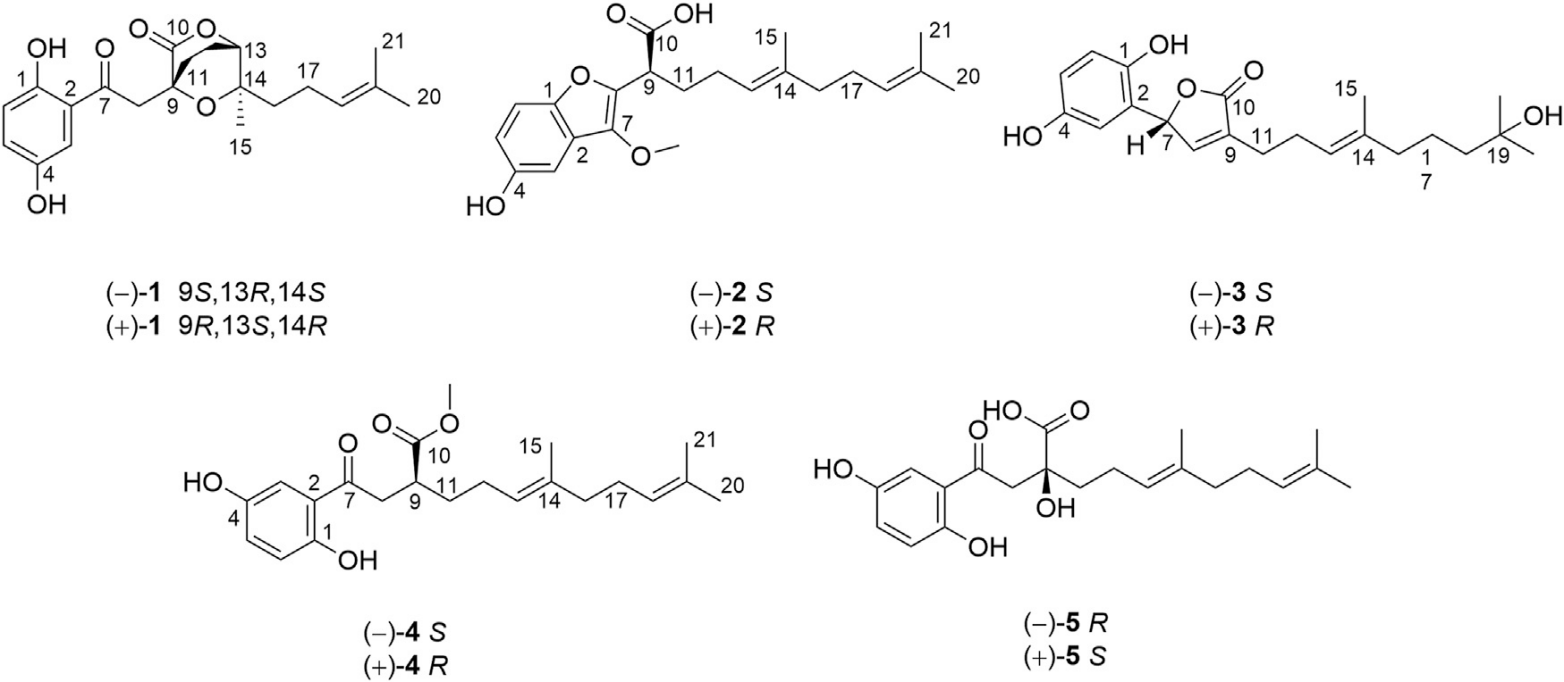
Structures of **1**–**5** from *Ganoderma lucidum*.

**FIGURE 2:**
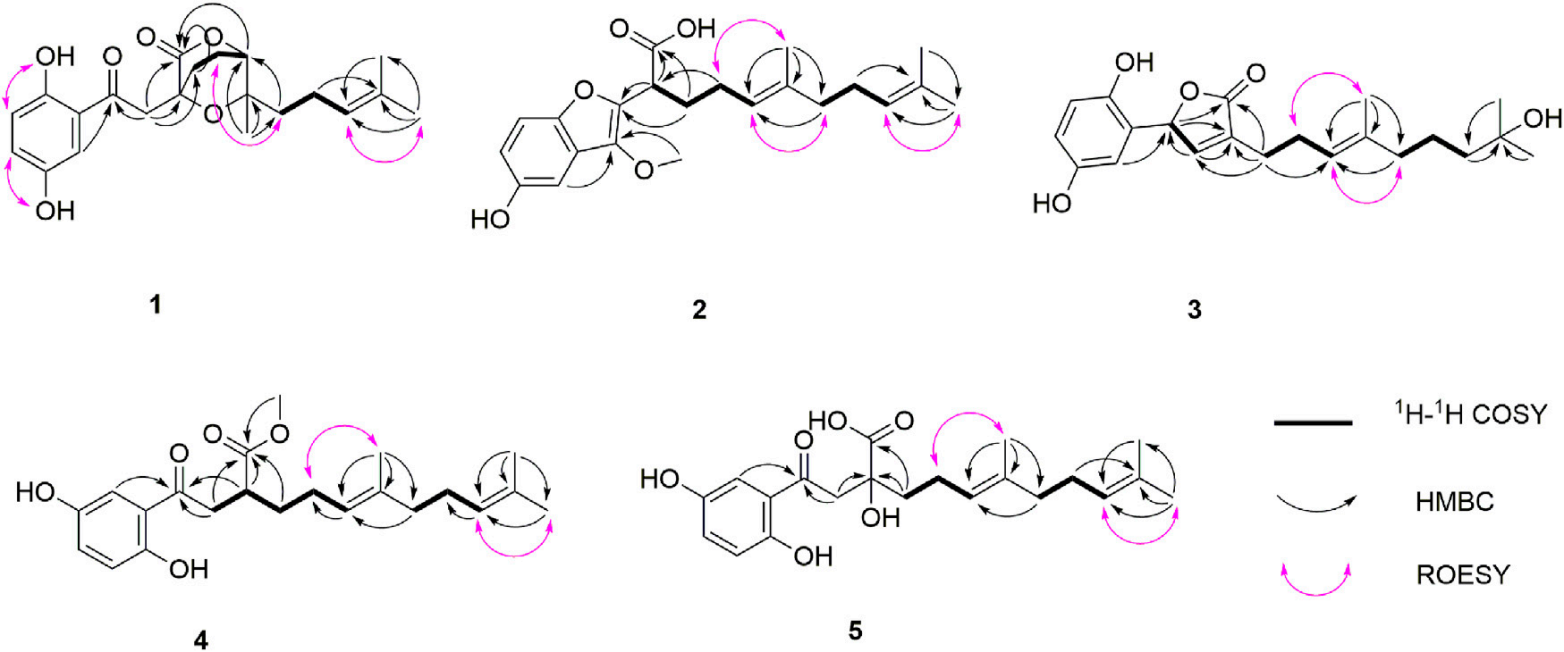
Key ^1^H–^1^H COSY, HMBC, and ROESY correlations of **1**–**5**.

**FIGURE 3:**
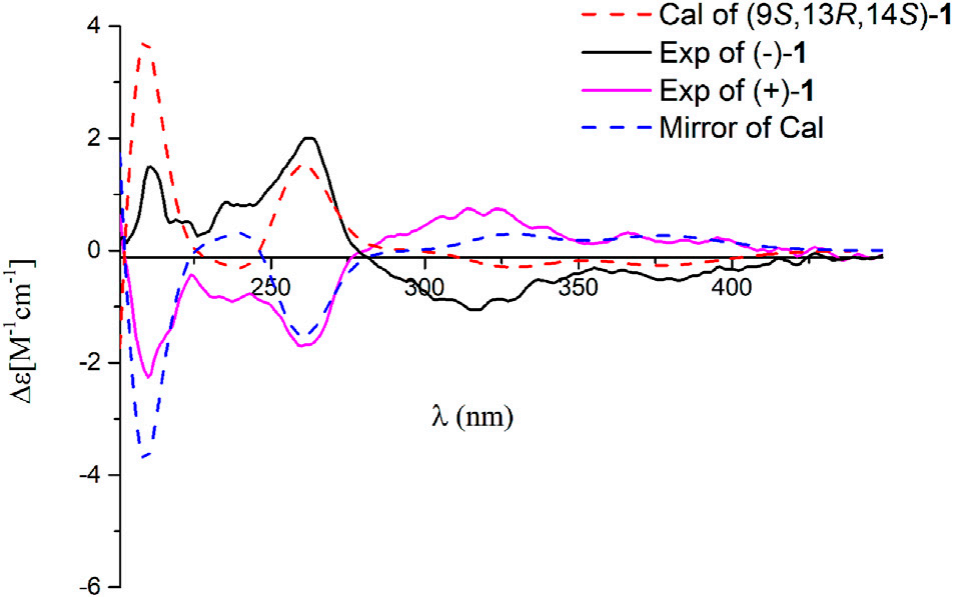
Comparison of the calculated ECD spectrum of **1** at the PBE1PBE/def2SVP level with the experimental one in MeOH. *σ* = 0.25 eV; shift = 13 nm.

Dayaolingzhiol I (**1**) (Figure 1) was isolated as yellowish gums, and its molecular formula was assigned as C_21_H_26_O_6_ (nine degrees of unsaturation) by the analysis of its HRESIMS, ^13^C NMR, and DEPT spectra. The ^1^H NMR spectrum (Table 1) shows a typical ABX spin system [*δ*_H_ 7.27 (1H, d, *J* = 2.9 Hz, H-3), 7.00 (1H, dd, *J* = 8.9, 2.9 Hz, H-5), and 6.78 (1H, d, *J* = 8.9 Hz, H-6)], suggesting the presence of a 1,2,4-trisubstituted benzene ring. The ^13^C NMR and DEPT (Table 1) spectra show three methyl, five sp^3^ methylene, one oxygenated sp^3^ methine (*δ*_C_ 81.1), four sp^2^ methine, eight non-protonated carbons (one ketonic carbonyl at *δ*_C_ 203.8, one ester carbonyl at *δ*_C_ 173.9, and four oxygenated quaternary carbons). The planar structure of **1** was mainly constructed by the results of 2D NMR experiments. The ^1^H–^1^H COSY spectrum (Figure 2) shows correlations of H_2_-11/H_2_-12/H-13 and H_2_-16/H_2_-17/H-18. The HMBC correlations of H-3, H_2_-8/C-7 (*δ*_C_ 203.8) indicate that the ketonic carbonyl C-7 is connected with C-2; the HMBC correlations (Figure 2) of H_2_-8, Ha-11, H-13 (*δ*_H_ 4.55)/C-10 (*δ*_C_ 173.9), and H_2_-8/C-9 (*δ*_C_ 74.4), C-11 (*δ*_C_ 27.8) imply that C-9 is connected with C-8, C-10, and C-11 by carbon bonds, while C-10 and C-13 (*δ*_C_ 81.1) are connected via oxygen bridges. These data show that the six-membered lactone ring is formed in **1**. In addition, the HMBC correlations of H_3_-15 (*δ*_H_ 1.24)/C-13, C-14 (*δ*_C_ 78.6), C-16, and H_2_-16 (*δ*_H_ 1.53)/C-13 imply that C-14 is connected with C-13, C-14, and C-15 through carbon bonds, H_2_-17/C-19 (*δ*_C_ 133.0), H_3_-20/C-18 (*δ*_C_ 124.8), C-19 (*δ*_C_ 133.0), C-21, and H_3_-21/C-18 indicate that C-18 and C-19 are connected via a double bond, and two terminal methyl are attached to C-19. The ROESY correlations (in DMSO-*d*_6_) (Figure 2) of 1-OH (*δ*_H_ 11.06)/H-6 (*δ*_H_ 6.79) and 4-OH (*δ*_H_ 9.14)/H-3 (*δ*_H_ 7.16), H-5 (*δ*_H_ 6.96) indicate the presence of 1-OH and 4-OH groups. One benzene ring, one six-membered lactone ring, one ketone group, and one double bond account for eight degrees of unsaturation, and the remaining one degree of unsaturation, in consideration of the diagnostic chemical shift for C-9 (*δ*_C_ 74.4) and C-14 (*δ*_C_ 78.6), allows to conclude the presence of an ether bond formed by C-9 and C-14. Therefore, the planar structure of **1** was deduced. The relative configuration of **1** was assigned by the ROESY experiments. The ROESY correlation (Figure 2) of Ha-12/H_2_-16 indicates the relative configuration of **1** as 9S*,13R*,14S*. To further confirm this structure, the density functional theory (DFT) at the B3LYP/6–311g(d,p) level was used to calculate chemical shifts of carbon atoms in the deduced structure with different relative configurations as 9S*,13R*,14S* (**1**–**1**) and 9R*,13S*,14S* (**1**–**2**), as well as the ether linkage broken structures of **1** with the relative configurations as 9R*,13S*,14R* (**1**–**3**); 9R*,13S*,14S* (**1**–**4**); 9R*,13R*,14R* (**1**–**5**); and 9R*,13R*,14S* (**1**–**6**) (Supplementary Figure S42). By the analysis of ^13^C NMR calculations, it is shown that the correlation coefficient (*R* ^2^) for calculated vs experimental chemical shifts in **1**–**1** to **1**–**6** were 0.99928, 0.99927, 0.99570, 0.99694, 0.99771, and 0.99776, respectively (Supplementary Figure S49), and the corrected mean absolute error (CMAE) values were 1.2, 1.2, 2.8, 2.5, 2.2, and 2.1 ppm (Supplementary Table S7–12). These values suggest that structural and stereochemical assignments to these substances are highly dependable. Moreover, the results of DP4+ probability analysis (Supplementary Figure S50) indicated a 100% matching degree between the experimental values and the calculated values of **1**–**1**, which further confirmed the correctness of the deduced structure and its stereochemistry. To clarify the absolute configuration of **1**, which is an enantiomeric isomer, ECD calculations at the PBE1PBE/def2SVP level and B3LYP/6–31(d,p) level were carried out. It was found that the ECD spectrum of (9*S*,13*R*,14*S*)**-1** matches well with the experimental one of (–)-**1** (Figure 3 and Supplementary Figure S51). Similarly, the calculated specific optical rotation for (9*S*,13*R*,14*S*)-**1** was –4.14 (Supplementary Table S13), which is closer to the experimental one of (–)-**1** ( ${\left[\alpha \right]}_{D}^{\text{25}}$ –6.67), supporting the absolute configuration of (–)-**1**. Thus, their absolute configurations were determined as 9*S*,13*R*,14*S* for (–)-**1** and 9*R*,13*S*,14*R* for (+)-**1**. Therefore, compound **1** was deduced and named dayaolingzhiol I. Of note, compound **1** is characteristic of the presence of a dioxabicyclo[2.2.2]octan-3-one motif in the terpenoidal chain, which makes it a structurally intriguing compound.

**TABLE 1:** ^1^H (600 MHz) and ^13^C NMR (150 MHz) data of **1** and **2** in methanol-*d*_4_ (*δ* in ppm, *J* in Hz).

No.	1		2	
	*δ*_H_	*δ*_C_	*δ*_H_	*δ*_C_
1		156.8 s		154.1 s
2		121.5 s		125.0 s
3	7.27 d (2.9)	116.7 d	6.92 d (2.5)	104.0 d
4		150.5 s		148.5 s
5	7.00 dd (8.9, 2.9)	126.0 d	6.72 dd (8.8, 2.5)	113.9 d
6	6.78 d (8.9)	119.5 d	7.18 d (8.8)	119.7 d
7		203.8 s		141.1 s
8	Ha: 3.52 d (15.8)	42.7 t		144.2 s
	Hb: 3.15 d (15.8)			
9		74.4 s	3.87 dd (8.5, 6.0)	43.6 d
10		173.9 s		175.7 s
11	Ha: 2.22 overlap	27.8 t	2.06 overlap	30.8 t
	Hb: 2.09 m		2.00 overlap	
12	Ha: 2.22 overlap	22.4 t	2.00 overlap	26.7 t
	Hb: 2.02 m			
13	4.55 dd (3.0, 1.7)	81.1 d	5.11 t (7.0)	124.4 days
14		78.6 s		137.4 s
15	1.24 s	22.4 q	1.52 s	16.1 q
16	1.53 overlap	40.1 t	1.96 t (7.5)	40.8 t
17	1.94 m	23.6 t	2.06 overlap	27.7 t
18	5.06 t (7.3)	124.8 d	5.10 t (7.1)	125.4 d
19		133.0 s		132.2 s
20	1.64 s	25.8 q	1.67 s	25.9 q
21	1.51 s	17.6 q	1.60 s	17.8 q
-OCH_3_			3.90 s	61.5 q
1-OH^*a*^	11.06 s			
4-OH^*a*^	9.14 s			

a These signals are observed in DMSO-d_6_.

**FIGURE 4:**
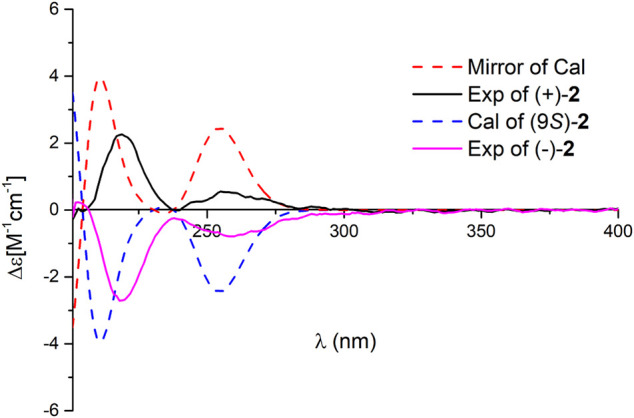
Comparison of the calculated ECD spectrum of **2** at the APFD/6–311+ *g* (2 d,p) level with the experimental one in MeOH. *σ* = 0.25 eV; shift = −25 nm.

Dayaolingzhiol J (**2**) was obtained as yellowish gums. It has a molecular formula C_22_H_30_O_5_ deduced by its HRESIMS, ^13^C NMR, and DEPT spectra. The ^1^H NMR spectrum of **2** (Table 1) shows a typical ABX spin system [(*δ*_H_ 6.92, d, *J* = 2.5 Hz, H-3; *δ*_H_ 6.72, dd, *J* = 8.8, 2.5 Hz, H-5; and *δ*_H_ 7.18, d, *J* = 8.8 Hz, H-6)]. The ^13^C NMR and DEPT spectra show four methyl (one oxygenated), four methylene, six methine (five sp^2^ and one sp^3^), and eight non-protonated carbons (one carbonyl and three oxygenated quaternary carbons). These data are similar to those of ganofuran B (Adams et al., 2010). The only difference is that a methoxy group appears at C-7 in **2** supported by the HMBC correlations (Figure 2) of -OCH_3_ (*δ*_H_ 3.90)/C-7 (*δ*_C_ 141.1). The significant ROESY correlation of H_2_-11 (*δ*_H_ 2.00)/H_3_-20 (*δ*_H_ 1.52) indicates that the *Δ* ^12(13)^ double bond is *E* form. Compound **2** was isolated as a racemic mixture, and chiral HPLC was used to afford (+)-**2** and (–)-**2**. In general, it is a great challenge to assign the absolute configuration at C-9 in the side chain. In this case, computational methods such as ECD calculations at the APFD/6–311+g(2d,p) and B3LYP/6–31(d,p) level were carried out (Figure 4 and Supplementary Figure S52). The results reveal that the ECD spectrum of (9*S*)-**2** matches well with the experimental one of (–)-**2** *.* Thus, the absolute configurations of the two enantiomers were assigned as 9*R* for (+)-**2** and 9*S* for (–)-**2**, respectively. In this way, the structure of **2** was deduced and named dayaolingzhiol J.

Dayaolingzhiol K (**3**) has the molecular formula C_21_H_28_O_5_ (eight degrees of unsaturation) on the basis of its HRESIMS, ^13^C NMR, and DEPT spectra. The ^1^H NMR spectrum (Table 2) shows a typical ABX spin system [*δ*_H_ 6.46 (1H, d, *J* = 2.9 Hz, H-3), 6.61 (1H, dd, *J* = 8.7, 2.9 Hz, H-5), 6.68 (1H, d, *J* = 8.7 Hz, H-6)]. The ^13^C NMR and DEPT spectra show four methyl, five methylene, five sp^2^ methine, one oxygenated sp^3^ methine, and seven non-protonated carbons (one ester carbonyl and three oxygenated quaternary carbons). These data resemble those of ganomycin K (Niedermeyer et al., 2013), differing in that the OH-18 in ganomycin K is reduced in compound **3**. This alteration is supported by the ^1^H–^1^H COSY correlation of H_2_-16/H_2_-17/H_2_-18 and the HMBC correlations of H_2_-16 (*δ*_H_ 1.96)/C-18 (*δ*_C_ 44.3) and H_3_-21 (*δ*_H_ 1.15)/C-18 (*δ*_C_ 44.3). Moreover, the significant ROESY correlations of H_2_-12 (*δ*_H_ 2.30)/H_3_-15 (*δ*_H_ 1.58) and H-13 (*δ*_H_ 5.14)/H_2_-16 (*δ*_H_ 1.96) indicate that the *Δ* ^13(14)^ double bond is *E* form. Compound **3** was isolated as a racemic mixture, and chiral HPLC was used to afford (+)-**3** and (–)-**3**. By carefully comparing the CD spectra of (+)-**3** and (–)-**3** with those of (+)- and (–)-zizhine A (Cao et al., 2016), the absolute configurations of the two enantiomers were assigned as *R* for (+)-**3** and *S* for (–)-**3**, respectively. Thus, the structure of **3** was deduced and named dayaolingzhiol K.

**TABLE 2:** ^1^H and ^13^C NMR data of **3** and **4** in methanol-*d*_4_ (*δ* in ppm, *J* in Hz).

No	3		4	
	*δ*_H_	*δ*_C_	*δ*_H_	*δ*_C_
1		148.9 s		156.5 s
2		123.5 s		120.3 s
3	6.46 d (2.9)	113.3 d	7.25 d (2.9)	115.4 d
4		151.5 s		150.6 s
5	6.61 dd (8.7, 2.9)	117.2 d	7.01 dd (8.9, 2.9)	125.9 d
6	6.68 d (8.7)	117.2 d	6.79 d (8.9)	119.7 d
7	6.23 d (1.6)	79.8 d	205.5 s
8	7.35 d (1.6)	151.1 d	Ha: 3.43 dd (17.9, 9.6)	41.4 t
			Hb: 3.17 dd (17.9, 4.3)
9		133.1 s	2.99 m	41.0 d
10		176.8 s	177.7 s
11	2.35 t (7.7)	26.2 t	Ha: 1.73 m	33.1 t
			Hb: 1.64 m
12	2.30 m	26.9 t	2.08 overlap	26.4 t
13	5.14 t (7.0)	124.1 d	5.13 t (7.6)	124.5 d
14		137.9 s		137.3 s
15	1.58 s	16.1 q	1.61 s	16.1 q
16	1.96 t (7.1)	41.1 t	2.00 t (7.5)	40.8 t
17	1.43 m	23.6 t	2.08 overlap	27.6 t
18	1.38 m	44.3 t	5.09 t (7.0)	125.3 d
19		71.5 s		132.2 s
20	1.15 overlap	29.2 q	1.66 s	25.9 q
21	1.15 overlap	29.2 q	1.59 s	17.8 q
-OCH_3_			3.68 s	52.3 q

^1^H recorded at 600 MHz and ^13^C NMR recorded at 150 MHz (compound **3**).

^1^H recorded at 500 MHz and ^13^C NMR recorded at 125 MHz (compound **4**).

Dayaolingzhiol L (**4**) was obtained as yellowish gums, and its molecular formula was assigned as C_22_H_30_O_5_ by analysis of its HRESIMS, ^13^C NMR, and DEPT spectra. The ^1^H NMR spectrum of **4** (Table 2) shows a typical ABX spin system (*δ*_H_ 7.25, d, *J* = 2.9 Hz, H-3; *δ*_H_ 7.01, dd, *J* = 8.9, 2.9 Hz, H-5; *δ*_H_ 6.79, d, *J* = 8.9 Hz, H-6), suggesting the presence of a 1,2,4-trisubstituted benzene ring. The ^13^C NMR and DEPT spectra show four methyl (one oxygenated), five methylene, six methine (five sp^2^ and one sp^3^), and seven non-protonated carbons (one ketone and one carbonyl). These data are similar to those of fornicin C (Niu et al., 2006). The only difference is a methyl ester in **4**, instead of the free carboxyl group in fornicin C, supported by the HMBC correlations (Figure 2) of -OCH_3_ (*δ*_H_ 3.68)/C-7 (*δ*_C_ 177.7). The ROESY correlation of H_2_-12 (*δ*_H_ 2.08)/H_3_-15 (*δ*_H_ 1.61) indicates that the *Δ* ^13(14)^ double bond is *E* form. Compound **4** is also a racemic mixture, and the absolute configurations of the two enantiomers after chiral separation by HPLC were assigned as *R* for (+)-**4** and *S* for (–)-**4**, respectively, by comparison of their CD spectra with those of (+)− and (–)−applanatumol S (Luo et al., 2016). The structure of **4** was thus determined and named dayaolingzhiol L.

**FIGURE 5:**
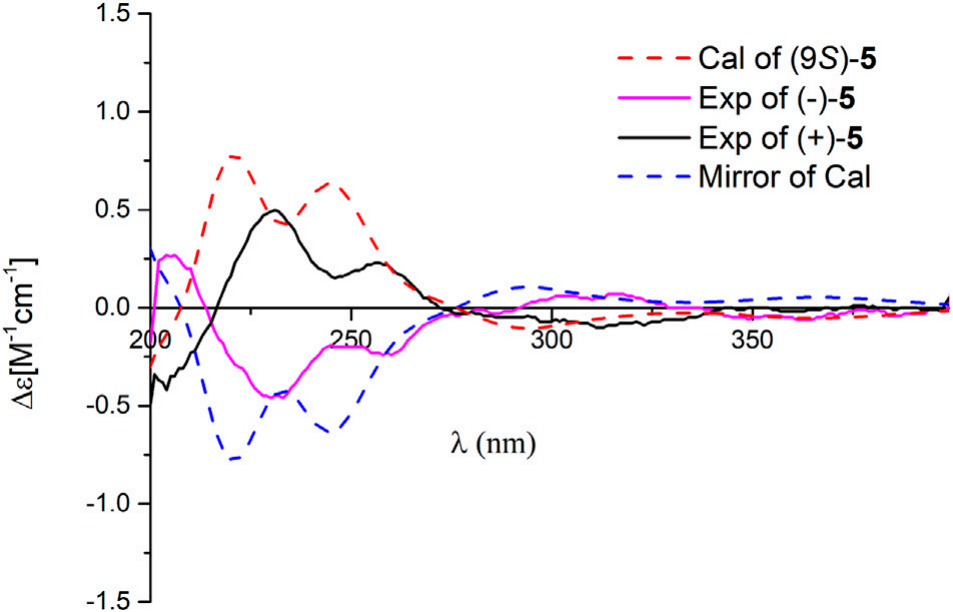
Comparison of the calculated ECD spectrum of **5** at the PBE1PBE/def2SVP level with the experimental one in MeOH. *σ* = 0.25 eV; shift = 0 nm.

Dayaolingzhiol M (**5**) was isolated as yellow gums, and it bears the same carbon skeleton with that of **4** by careful analysis of their NMR spectra. The ^1^H NMR spectrum of **5** (Table 3) shows a typical ABX spin system (*δ*_H_ 7.22, d, *J* = 2.9 Hz, H-3; *δ*_H_ 7.01, dd, *J* = 8.9, 2.9 Hz, H-5; *δ*_H_ 6.79, d, *J* = 8.9 Hz, H-6). The ^13^C NMR and DEPT spectra show three methyl, five methylene, five sp^2^ methine, and eight non-protonated carbons (one ketone, one carbonyl, and three oxygenated quaternary carbons). These data also resemble those of fornicin C (Niu et al., 2006). Differently, a hydroxyl is attached to C-9 (*δ*_C_ 75.8) in **5**, gaining support from the HMBC correlations of H_2_-8 and H_2_-11/C-9. The ROESY correlation of H_2_-12 (*δ*_H_ 2.23)/H_3_-15 (*δ*_H_ 1.62) indicates that the *Δ* ^13(14)^ double bond is *E* form. Compound **5** is also a racemate, which was further purified by chiral HPLC to afford (+)-**5** and (–)-**5**. To clarify their absolute configurations, ECD calculation was used at the PBE1PBE/def2SVP level and B3LYP/6–31(d,p) level (Figure 5 and Supplementary Figure S53). It was found that the ECD spectrum of (9*S*)*-* **5** matches well with the experimental one of (+)-**5** (Figure 5); thus, the absolute configurations of the two enantiomers were assigned as 9*S* for (+)-**5** and 9*R* for (–)-**5**, respectively. Therefore, the structure of **5**, named dayaolingzhiol M, was deduced.

**TABLE 3:** ^1^H (500 MHz) and ^13^C NMR (125 MHz) data of **5** in methanol-*d*_4_ (*δ* in ppm, *J* in Hz).

No.	*δ*_H_	*δ*_C_	No.	*δ*_H_	*δ*_C_
1		156.6 s	12	Ha: 2.23 m	22.9 t
2		120.8 s		Hb: 2.00 overlap	
3	7.22 d (2.9)	115.7 d	13	5.14 t-like (6.8)	124.7 d
4		150.7 s	14		136.9 s
5	7.01 d (8.9, 2.9)	126.1 d	15	1.62 s	16.0 q
6	6.79 d (8.9)	119.7 d	16	2.00 overlap	40.8^*a*^ t
7		204.8 s	17	2.08 m	27.7 t
8	Ha: 3.54 d (17.3)	48.4 t	18	5.10 t (7.0)	125.4 d
	Hb: 3.45 d (17.3)		19		132.2 s
9		75.8 s	20	1.67 s	25.9 q
10		178.6 s	21	1.60 s	17.8 q
11	1.78 m	41.0^*a*^ t			

a Signals with the same symbol might be interchangeable.

In this experiment, we obtained five long-chain meroterpenoids dayaolingzhiols I−M (**1**–**5**). Among them, dayaolingzhiol I (**1**) is a relatively rare meroterpenoid with dioxabicyclo[2.2.2]octan-3-one motif of *G. lucidum.* At the same time, dayaolingzhiol J (**2**) belongs to the benzofuran type of meroterpenoid, and few benzofuran type of meroterpenoids have been reported from *Ganoderma* so far (Adams et al., 2010). These two pairs of meroterpenoids together with the remaining three pairs of new meroterpenoids reveal the structural diversity of *Ganoderma* meroterpenoids.

**FIGURE 6:**
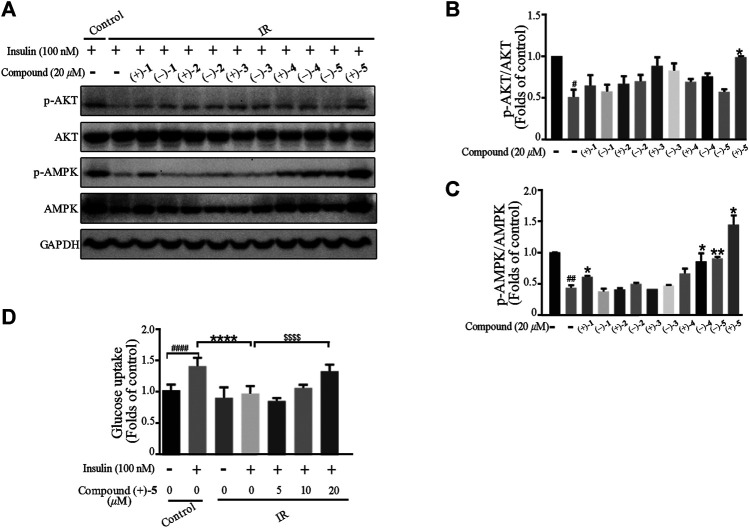
Effects of the compounds on insulin-induced insulin resistance in myotubes. **(A–C)** Compounds attenuated insulin signal pathway disruption. Bar graphs show the quantification of the indicated proteins. ^#^ *p* < 0.05 and ^##^ *p* < 0.01 compared with without insulin treatment control; ^*^ *p* < 0.05 and ^**^ *p* < 0.01 compared with insulin treatment alone. **(D)** Compound (+)-**5** improved insulin-stimulated glucose uptake in myotubes. ^####^ *p* < 0.0001 compared with without insulin-stimulated control; ^****^ *p* < 0.0001 compared with the insulin treatment group; ^$$$$^ *p* < 0.0001 compared with insulin treatment alone.

*Ganoderma lucidum* cultivated at Dayao County of Yunnan Province is locally used as a health-care product with a pronounced effect on diabetes. To explore the protective effect of *Ganoderma* meroterpenoids against IR, insulin exposed C2C12 myotubes were used. C2C12 cells were pretreated with different compounds for 24 h, and cell viability was not decreased at 20 *μ*M. The CCK-8 assay showed no obvious cytotoxicity of compounds, except (+)-**4** and (–)-**4** (Supplementary Figure S54). When the cells differentiated into myotubes, they were pretreated with 100 nM insulin for 24 h to mimic the IR model and then exposed to 20 *μ*M compounds for an additional 24 h. In the cellular model of IR, insulin-stimulated glucose uptake and the expression of phospho-adenosine monophosphate–activated protein kinase (p-AMPK) and phospho-AKT (p-AKT) were lower than those in control cells. The results showed that incubation with (+)-**1**, (–)-**4**, and (–)-**5** caused an increase in the phosphorylation of AMPK in IR C2C12 cells after insulin stimulation. At the same time, compound (+)-**5** treatment caused an increase in protein expression of p-AMPK and p-AKT in IR C2C12 cells (Figure 6A−C). Further glucose uptake experiments showed that compound (+)-**5** improved the capacity of glucose uptake in IR cells in a concentration-dependent manner (Figure 6D). So far, some meroterpenoids were found to have beneficial effects to insulin sensitivity in PA-induced C2C12 cells, such as ganomycin C and ganodercin D (Qin et al., 2021). Ganomycin I exhibited potent insulin-sensitizing effects in KK-A^y^ mice, and its analog (*R,E*)-5-(4-(tert-butyl)phenyl)-3-(4,8-dimethylnona-3,7-dien-1-yl)furan-2(5H)-one ameliorates insulin resistance (Wang et al., 2017; Wang et al., 2018). These meroterpenoids with long side chains showed advantages in the amelioration of insulin resistance.

**FIGURE 7:**
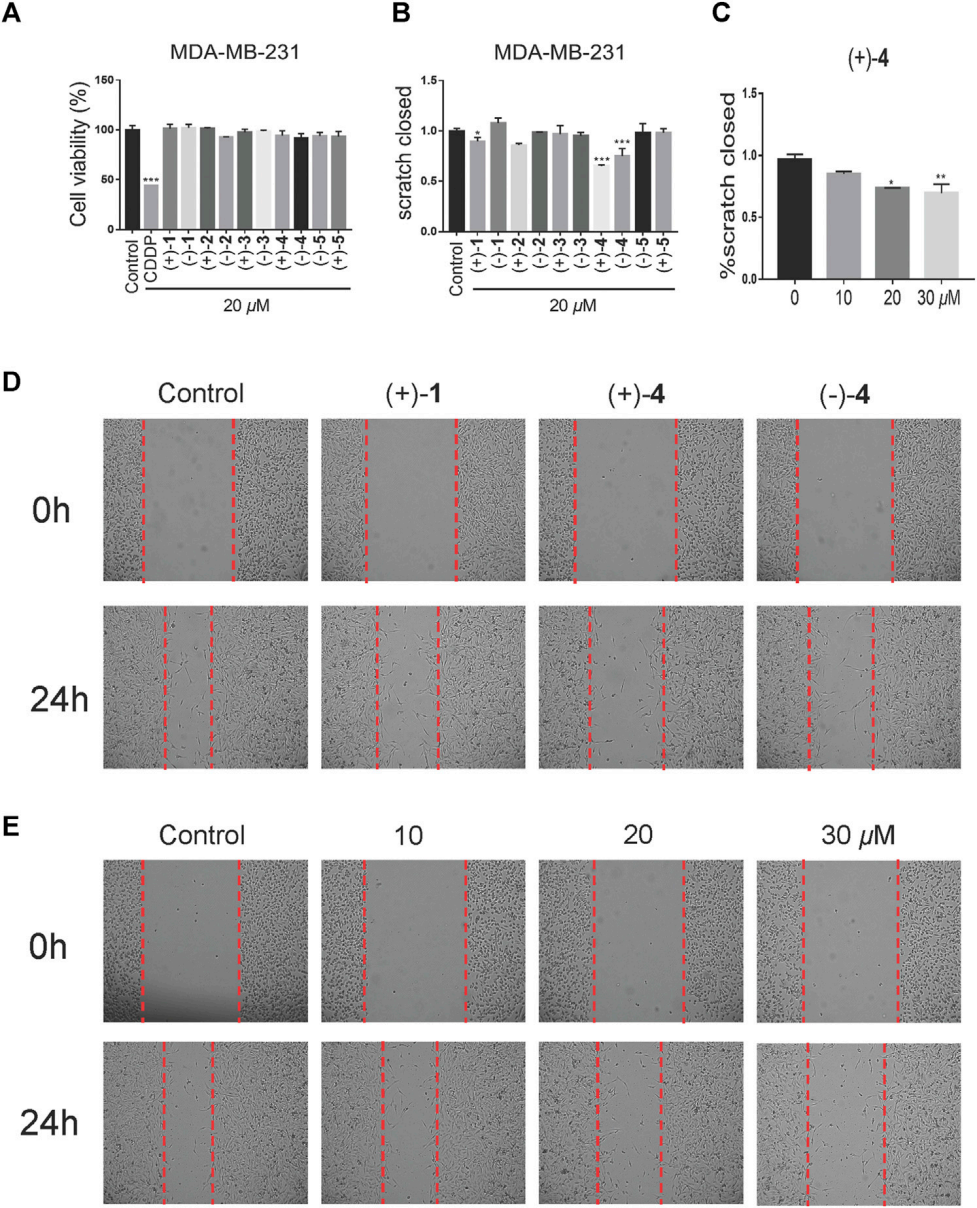
Effects of compounds on cell migration at low-toxicity doses. **(A)** Cell viability of MDA-MB-231 was treated with the vehicle or 20 *μ*M compounds for 48 h (*n* = 3). **(B)** Wound healing assay of the MDA-MB-231 cell line, treated with the vehicle or 20 *μ*M compounds for 24 h (*n* = 3). **(C)** The dose-dependent migratory inhibition effect of the compound (+)-**4**. **(D)** Representative pictures of compounds (+)-**1** (+)-**4** and (–)-**4** at 20 *μ*M for 24 h of MDA-MB-231. **(E)** Representative pictures of compound (+)-**4** at different doses for 24 h in MDA-MB-231 cells. Data are of three replicates (*n* = 3) ^*^ *p* < 0.05, ^**^ *p* < 0.01, one-way ANOVA.

Our previous study showed that *Ganoderma* meroterpenoids with long side chains are active toward breast cancer cell migration (Cai et al., 2021). In this article, 10 optically active compounds, all possessing a long side chain, were tested for their suppressive activity in the triple-negative breast cancer cell line (MDA-MB-231) by using cell viability and cell migration assays. First, the cell viability assay was carried out in the MDA-MB-231 cell line. As shown in Figure 7A, compounds demonstrated weak effect on cell viability at the concentration of 20 *μ*M. Wound healing assay was investigated in the MDA-MB-231 cell line at 20 *μ*M. The results showed that compounds (+)-**1**, (+)-**4**, and (–)-**4** exhibit a migration inhibitory effect compared to the DMSO group in the MDA-MB-231 cell line, of which (+)-**4** showed stronger inhibitory effect (Figures 7B, D). Furthermore, the effect of compound (+)-**4** was found to be dose-dependent at concentrations of 10 *μ*M, 20 *μ*M, and 30 *μ*M (Figures 7C,E).

## Experimental

### General

Optical rotations of (+)-**1**, (–)-**1**, (+)-**3**, and (–)-**3** were recorded on an Anton Paar MCP-100 digital polarimeter, and the optical rotations of (+)-**2**, (–)-**2**, (+)-**4**, (–)-**4**, (+)-**5**, and (–)-**5** were tested on an Anton Paar MCP-500 digital polarimeter. UV and CD spectra were measured on a Chirascan instrument. NMR spectra were recorded on a Bruker 600 MHz or a Bruker 500 MHz spectrometer, with TMS as an internal standard. Positive HRESIMS data of **1** were collected by a Shimazu LC-20AD AB SCIEX triple TOF 6600+ MS spectrometer. The HRESIMS data of **2** and **4** were recorded on a Waters Xevo G2-XS Qtof MS spectrometer. An Agilent 6210 ESI/TOF MS spectrometer was used to collect HRESIMS data of **3** and **5**. C-18 silica gel (40–60 *μ*m; Daiso Co., Japan), MCI gel CHP 20 P (75–150 *μ*m, Mitsubishi Chemical Industries, Tokyo, Japan), and Sephadex LH-20 (Amersham Pharmacia, Uppsala, Sweden) were used for column chromatography. Silica gel (Qingdao Marine Chemical Inc. Qingdao, People’s Republic of China) was used for preparative TLC. Semi-preparative HPLC was carried out on a Saipuruisi chromatograph with a YMC-Pack ODS-A column (250 mm × 10 mm, i.d. 5 *μ*m), and preparative HPLC on a Chuangxin–Tongheng chromatograph equipped with a Thermo Hypersil GOLD-C18 column (250 mm × 21.2 mm, i.d. 5 *μ*m). Chiral HPLC analysis of **1** and **4** were carried out on an Agilent 1260 chromatograph with a Cellulose-3 column (LC, 250 mm × 4.6 mm, i.d. 5 *μ*m), chiral HPLC analysis of **2** and **5** were carried out on an Agilent 1260 chromatograph with an i-Amylose-1 column (LC, 250 mm × 10 mm, i.d. 5 *μ*m), and chiral HPLC analysis of **3** was carried out on a Saipuruisi chromatograph with a Daicel Chiralpak column (IC, 250 mm × 4.6 mm,i.d. 5 *µ*m).

#### Fungal Material

The fruiting bodies of *G. lucidum* were collected from Dayao County, Yunnan Province, People’s Republic of China, in April 2018. The authentication of this material was finished by Prof. Zhu-Liang Yang at the Kunming Institute of Botany, Chinese Academy of Sciences, Kunming, People’s Republic of China, and the voucher specimen (CHYX-0615) of it is deposited at the Institute for Inheritance-Based Innovation of Chinese Medicine, School of Pharmaceutical Sciences, Shenzhen University Health Science Center, People’s Republic of China.

#### Extraction and Isolation

The powdered fruiting bodies of *G. lucidum* (30.0 kg) were extracted with 95% EtOH under percolation (240 L) at room temperature, and a crude extract (2.1 kg) was provided and then was suspended in H_2_O and partitioned with EtOAc three times to obtain an EtOAc part (1.1 kg). This extract was separated by an MCI gel CHP 20P column (MeOH/H_2_O, 40–100%) to afford thirteen fractions (Fr.1–Fr.13).

Fr.10 (30.3 g) was gel-filtrated over Sephadex LH-20 (MeOH) and further subjected to a silica gel column with increasing acetyl acetate in petroleum ether (15:1–1:1) to afford three portions (Fr.10.1−Fr.10.3). The second portion (2.1 g) was cut into five parts (Fr.10.2.1−Fr.10.2.5) using preparative HPLC (MeOH/H_2_O containing 0.05% TFA in water, 55–100%). Fr.10.2.1 (197.0 mg) was separated by preparative thin-layer chromatography (PTLC) (CH_2_Cl_2_/acetone = 10:1) to obtain Fr.10.2.1.1−Fr.10.2.1.4. Among them, Fr.10.2.1.3 (13.0 mg) was purified by semi-preparative HPLC (MeOH/H_2_O containing 0.05% TFA in water, 58%, flow rate: 3 mL/min) to afford compound **3** (3.0 mg, t_R_ = 36.9 min).

Fr.11 (123.0 g) was subjected to a silica gel column with increasing acetone in petroleum ether (10:1–5:1) to give four parts (Fr.11.1−Fr.11.4). Among them, the first part (2.6 g) was purified by Sephadex LH-20 (MeOH) to obtain a fraction (129.0 mg), which was subjected to semi-preparative HPLC (MeOH/H_2_O, 65%, 100%, flow rate: 3 mL/min) to give two fractions (Fr.11.1.1 and Fr.11.1.2). Fr.11.1.2 (70.0 mg) was then fractionated by semi-preparative HPLC (aqueous AcCN, 75%, flow rate: 3 mL/min) to produce compound **4** (0.5 mg, t_R_ = 19.4 min). Fr.11.2 (3.4 g) was subjected to Sephadex LH-20 (MeOH) to acquire three parts (Fr.11.2.1−Fr.11.2.3). Then, Fr.11.2.3 (147.0 mg) was segregated by semi-preparative HPLC (MeOH/H_2_O, 73%, flow rate: 3 mL/min) to afford compound **1** (0.9 mg, t_R_ = 13.6 min). Fr.11.4 (99.0 g) was gel-filtrated over Sephadex LH-20 (MeOH) to afford Fr.11.4.1−Fr.11.4.3. Then, the last part (1.7 g) was cut into five parts (Fr.11.4.3.1−Fr.11.4.3.5) by vacuum liquid chromatography (VLC) by increasing acetone in petroleum ether (15:1–5:1). Fr.11.4.3.5 (1.3 g) was subjected to PTLC (petroleum ether/acetone = 3:2) to obtain three parts (Fr.11.4.3.5.1−Fr.11.4.3.5.3), of which, Fr.11.4.3.5.1 (307.0 mg) (R_f_ = 0.8) was subjected to preparative HPLC (MeOH/H_2_O, 66–95%) to obtain six parts (Fr.11.4.3.5.1.1−Fr.11.4.3.5.1.6). Fr.11.4.3.5.1.1 (16.0 mg) was purified by semi-preparative HPLC (MeOH/H_2_O, 73%, flow rate: 3 mL/min) to afford compound **2** (2.8 mg, t_R_ = 40.7 min). Fr.11.4.3.5.3 (374.0 mg) (R_f_ = 0.2) was cut by semi-preparative HPLC (aqueous AcCN with 0.05% TFA in water, 62%, flow rate: 3 mL/min) to afford eleven parts (Fr.11.4.3.5.3.1−Fr.11.4.3.5.3.11). Among them, Fr.11.4.3.5.3.1 (23.0 mg) was purified by semi-preparative HPLC (MeOH/H_2_O containing 0.05% TFA in water, 74%, flow rate: 3 mL/min) to produce compound **5** (1.3 mg, t_R_ = 25.8 min).

In addition, racemic **1**, **4**, **5** and an aliquot of **2**, **3** were further purified by chiral HPLC to provide their enantiomers (flow rate: 1 mL/min): (+)-**1** (0.4 mg, t_R_ = 29.0 min) and (–)-**1** (0.5 mg, t_R_ = 31.9 min) (*n*-hexane/ethanol, 93:7); (+)-**2** (0.5 mg, t_R_ = 14.5 min) and (–)-**2** (0.5 mg, t_R_ = 15.4 min) (*n*-hexane/ethanol containing 0.05% TFA, 95:5); (+)-**3** (0.9 mg, t_R_ = 11.5 min) and (–)-**3** (0.6 mg, t_R_ = 14.0 min) (*n*-hexane/ethanol, 90:10); (+)-**4** (0.2 mg, t_R_ = 17.1 min) and (–)-**4** (0.2 mg, t_R_ = 21.3 min) (*n*-hexane/ethanol containing 0.05% TFA, 95:5); (–)-**5** (0.6 mg, t_R_ = 25.4 min) and (+)-**5** (0.7 mg, t_R_ = 33.0 min) (*n*-hexane/ethanol containing 0.05% TFA, 92:8). Due to the less quantity of compounds **4** and **5**, these two compounds were enriched from fungal material of *Ganoderma cochlear* (CHYX-0589 for *G*. *cochlear*, and the detail isolation was not described here).

#### Compound Characterization Data

(±)*-*Dayaolingzhiol I (**1**), yellowish gum; UV (MeOH) λ_max_ (log *ε*) 366 (3.53), 258 (3.82), 229 (4.11) nm; {[α] ${}_{{\text{D}}^{25}}$ +6.7 (*c* 0.03, MeOH); CD (MeOH) *Δ* *ε*_208_ –3.26, *Δ* *ε*_222_ –0.19, *Δ* *ε*_261_ –1.76, *Δ* *ε*_314_ +0.87; (+)-**1**}; {[α] ${}_{{\text{D}}^{25}}$ –6.7 (*c* 0.03, MeOH); CD (MeOH) *Δ* *ε*_211_ +1.96, *Δ* *ε*_222_ +1.26, *Δ* *ε*_262_ +2.32, *Δ* *ε*_316_ –1.18; (–)-**1**}; HRESIMS *m/z* 375.1805 [M + H]^+^ (calcd for C_21_H_27_O_6_, 375.1802); ^1^H and ^13^C NMR data (see Table 1).

(±)-Dayaolingzhiol J (**2**), yellowish gum; UV (MeOH) *λ*_max _(log *ε*) 370 (2.66), 297 (3.02), 260 (3.47) nm; {[α] ${}_{{\text{D}}^{25}}$ +8.2 (*c* 0.05, MeOH); CD (MeOH) *Δ* *ε*_216_ +1.46, *Δ* *ε*_239_ –0.02, *Δ* *ε*_255_ +0.35; (+)-**2**}; {[α] ${}_{{\text{D}}^{25}}$ –11.9 (*c* 0.04, MeOH); CD (MeOH) *Δ* *ε*_218_ –1.64, *Δ* *ε*_238_ –0.16, *Δ* *ε*_260_ –0.49; (–)-**2**}; HRESIMS *m/z* 395.1835 [M + Na]^+^ (calcd for C_22_H_28_O_5_Na, 395.1829); ^1^H and ^13^C NMR data (see Table 1).

(±)-Dayaolingzhiol K (**3**), yellowish gum; UV (MeOH) *λ*_max_ (log *ε*) 299 (3.24) nm; {[α] ${}_{{\text{D}}^{25}}$ +29.4 (*c* 0.09, MeOH); CD (MeOH) *Δ* *ε*_209_ +11.29, *Δ* *ε*_260_ –0.30, *Δ* *ε*_306_ +0.68; (+)-3}; {[α] ${}_{{\text{D}}^{25}}$ –41.9 (*c* 0.06, MeOH); CD (MeOH) *Δ* *ε*_209_ –11.17, *Δ* *ε*_259_ +0.17, *Δ* *ε*_303_ –0.84; (–)-**3**}; HRESIMS *m/z* 383.1849 [M + Na]^+^ (calcd for C_21_H_28_O_5_Na, 383.1834); ^1^H and ^13^C NMR data (see Table 2).

(±)-Dayaolingzhiol L (**4**), yellowish gum; UV (MeOH) *λ*_max_ (log *ε*) 366 (3.08), 257 (3.35), 226 (3.69) nm; {[α] ${}_{{\text{D}}^{25}}$ +9.9 (*c* 0.04, MeOH); CD (MeOH) *Δ* *ε*_215_ –0.30, *Δ* *ε*_229_ +0.08; (+)-**4**}; {[α] ${}_{{\text{D}}^{25}}$ –7.5 (*c* 0.04, MeOH); CD (MeOH) *Δ* *ε*_215_ +0.51, *Δ* *ε*_225_ –0.76; (–)-**4**}; HRESIMS *m/z* 397.1984 [M + Na]^+^ (calcd for C_22_H_30_O_5_Na, 397.1985); ^1^H and ^13^C NMR data (see Table 2).

(±)-Dayaolingzhiol M (**5**), Yellow Gum; UV (MeOH) *λ*_Max_ (log *ε*) 367 (2.89), 258 (3.18), 228 (3.48) nm; {[α] ${}_{{\text{D}}^{25}}$ +7.4 (*c* 0.18, MeOH); CD (MeOH) *Δ* *ε*_204_ –0.42, *Δ* *ε*_231_ +0.50, *Δ* *ε*_257_ +0.23, *Δ* *ε*_313_ –0.10; (+)-**5**}; {[α] ${}_{{\text{D}}^{25}}$ –5.8 (*c* 0.20, MeOH); CD (MeOH) *Δ* *ε*_204_ +0.28, *Δ* *ε*_233_ –0.46, *Δ* *ε*_260_ –0.24, *Δ* *ε*_316_ +0.07; (–)-**5**}; HRESIMS *m/z* 399.1782 [M + Na]^+^ (Calcd for C_21_H_28_O_6_Na, 399.1784); ^1^H and ^13^C NMR data (see Table 3).

### Insulin Resistance Assay

#### Cell Culture

C2C12 cells, a mouse skeletal muscle myoblast line (Procell Life Science and Technology Co., Wuhan, China), were cultured by using the same method as previously described (Qin et al., 2021). When the cells differentiated into elongated, multinucleated myotubes, they were exposed to 100 nM insulin for an additional 24 h to mimic insulin resistance.

#### Cell Viability Assay

C2C12 (5× 10^5^ cells/mL) were seeded into 96-well plates with complete DMEM. After being cultured overnight, C2C12 cells were treated with five pairs of meroterpenoids or DMSO for 24 h. Then Cell Count Kit-8 (CCK-8, Beyotime, Shanghai, China) was added into each well for 1 h at 37 °C. The absorbance of each well was recorded at 450 nm using a microplate reader (BioTek, United States).

#### Glucose Uptake Assay

C2C12 cells were incubated in DMEM (low glucose) for 6 h and placed in high-glucose DMEM before adding compounds, and 30 min after compound treatment, insulin (100 nmol/L) was added and cultured for 4 h. Glucose content of the culture supernatant was measured using the Glucose Colorimetric/Fluorometric Assay Kit (BioVison, K606-100).

#### Western Blot

After different compound treatment for 24 h in the IR model, total protein was extracted from the cell lines using radioimmunoprecipitation assay (RIPA) buffer (Beyotime, China) containing protease cocktail (Roche, Germany) and quantified the protein samples using the BCA assay (Thermo Scientific, United States). Equal amounts of protein extracts were separated by 8% SDS-PAGE and transferred to PVDF membranes. The membranes were blocked with 5% BSA, then with the indicated antibodies overnight at 4 °C, and followed the incubation with horseradish peroxidase (HRP)–conjugated secondary antibody at room temperature. The bands were visualized and measured using the ECL kit (Pierce, United States). The densitometry analysis of the immunoblot results was performed using ImageJ software (NIH, United States).

#### Statistical Analysis

All experimental data in this study were performed in triplicate. The results were represented as mean ± SD. Statistical analyses were performed using Graphpad Prism 6 (GraphPad Software, San Diego, CA, United States) with one-way ANOVA. Differences were considered significant when ^*^ *p* ≤ 0.05, ^**^ *p* ≤ 0.01, ^***^ *p* ≤ 0.001, and ^****^ *p* ≤ 0.0001.

### Wound Healing Assay in MDA-MB-231 Cells

The biological evaluation for wound healing in MDA-MB-231 cells was conducted as the previously reported protocols (Cai et al., 2021).

## Conclusion

To conclude, triterpenoids and polysaccharides as chemical compositions of *Ganoderma* mushrooms have been traditional gnosia for decades. Our present study resulted in the isolation of five pairs of meroterpenoidal enantiomers, representing new chemical composition of *G. lucidum* and aiding an in-depth insight into *Ganoderma* metabolites. The activation of (+)-**5** on Akt, AMPK, and glucose uptake in C2C12 cells indicates its role in diabetes prevention. In addition, the inhibitory effects of compounds (+)-**1**, (+)-**4**, and (–)-**4** on cell mobility in MDA-MB-231 cells indicate their role in TNBC. These results shed light on the traditional uses of *G. lucidum*, implying *G. lucidum*–derived compounds’ potential in insulin-resistant diseases and cancer, and might inspire a consideration of *Ganoderma* meroterpenoids as dietary supplements.

## Data Availability

The original contributions presented in the study are included in the article/Supplementary Material; further inquiries can be directed to the corresponding author.
