# Erratum: Meroterpenoids From *Ganoderma lucidum* Mushrooms and Their Biological Roles in Insulin Resistance and Triple-Negative Breast Cancer

**DOI:** 10.3389/fchem.2021.817898

**Published:** 2021-12-23

**Authors:** Frontiers Production Office

**Affiliations:** Frontiers Media SA, Lausanne, Switzerland

**Keywords:** *Ganoderma lucidum*, meroterpenoids, Akt phosphorylation, AMPK phosphorylation, glucose uptake, cell migration

Due to an error during the final proofing stage, the incorrect version of the article was published.

The publisher apologizes for this mistake. The original article has now been updated to reflect the correct version.

