# Supplementary material for: Meroterpenoids From Ganoderma lucidum Mushrooms and Their Biological Roles in Insulin Resistance and Triple-Negative Breast Cancer
Source: Front Chem. 2021 Nov 3;9:772740. doi: 10.3389/fchem.2021.772740 (PMC8595597; doi:10.3389/fchem.2021.772740)
Supplement: Supplementary file 2 [file DataSheet4.docx]

Original Images Ⅰ

**Contents**

Figure SS1. Original image of p-AKT in Figure 6 (A).

Figure SS2. Original image of AKT in Figure 6 (A).

Figure SS3. Original image of p-AMPK in Figure 6 (A).

Figure SS4. Original image of AMPK in Figure 6 (A).

Figure SS5. Original image of GAPDH in Figure 6 (A).


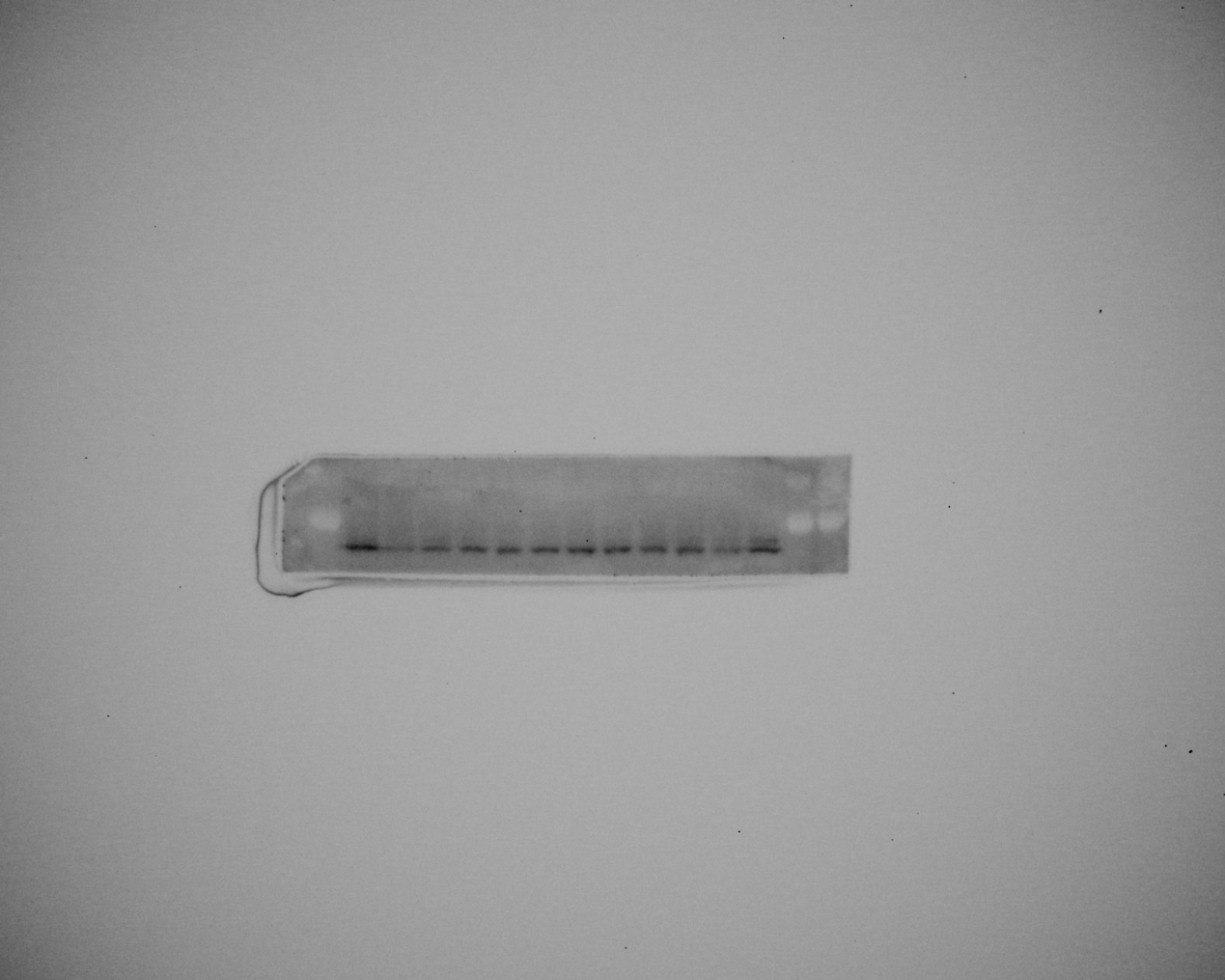


Figure SS1. Original image of p-AKT in Figure 6 (A).


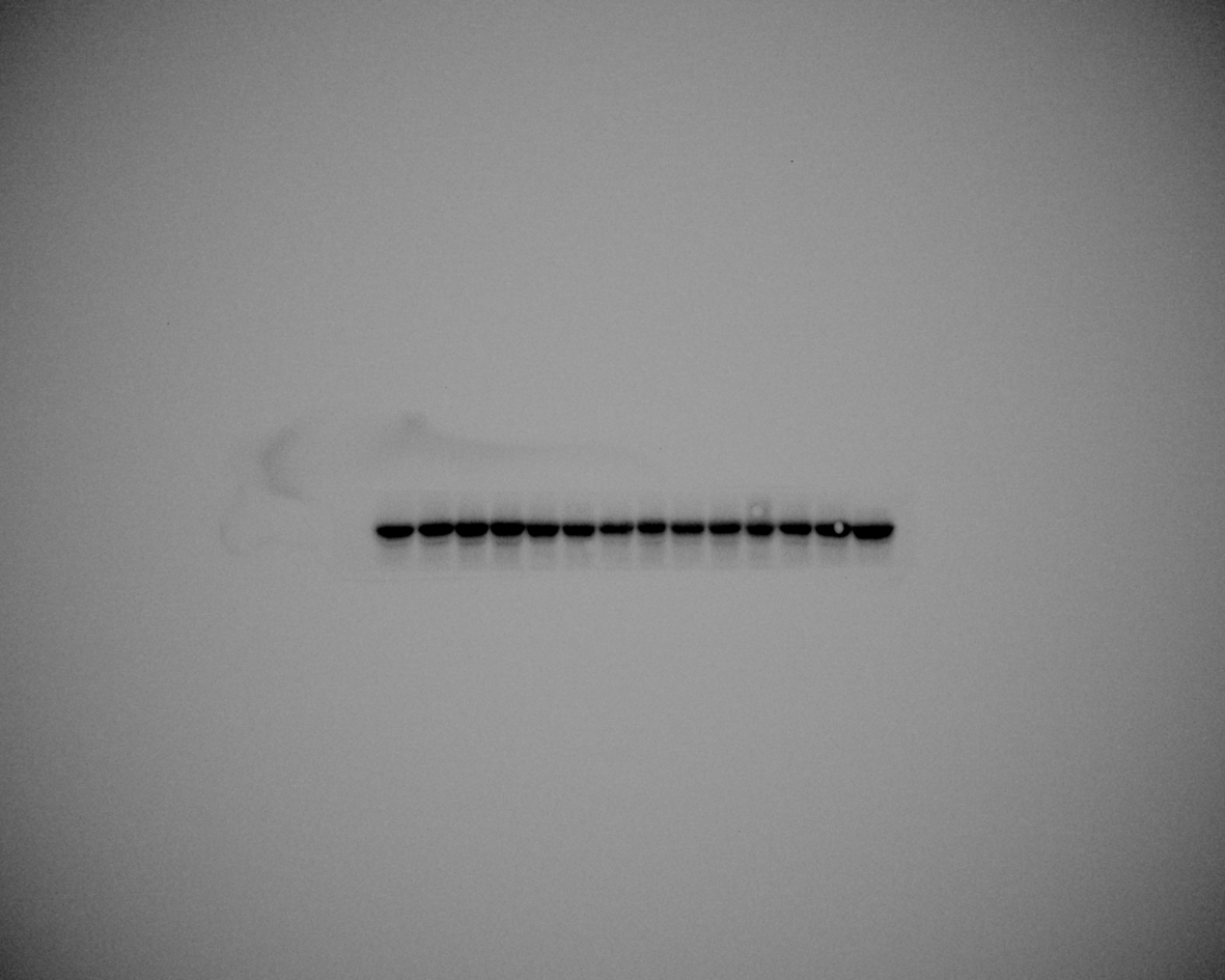


Figure SS2. Original image of AKT in Figure 6 (A).


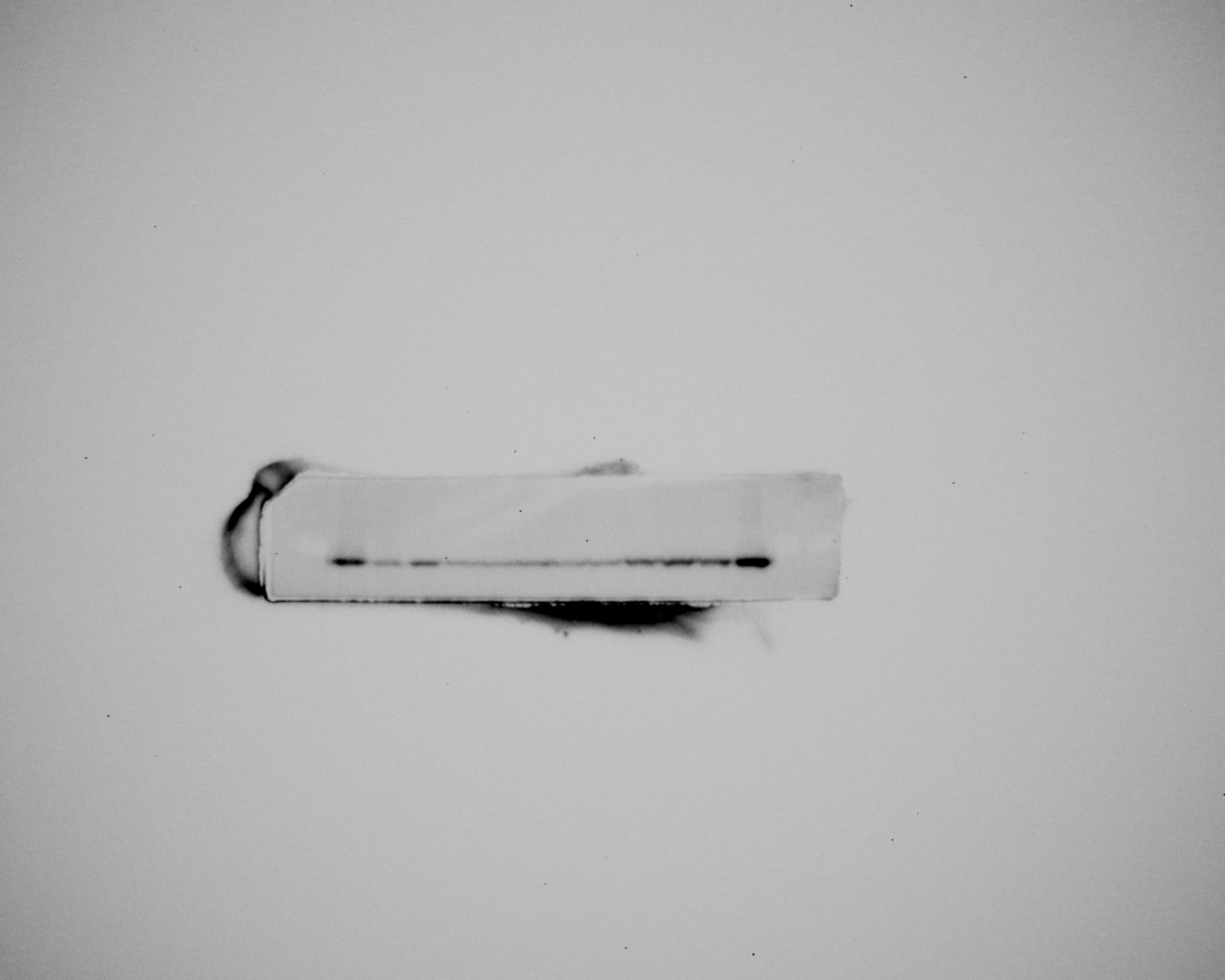


Figure SS3. Original image of p-AMPK in Figure 6 (A).


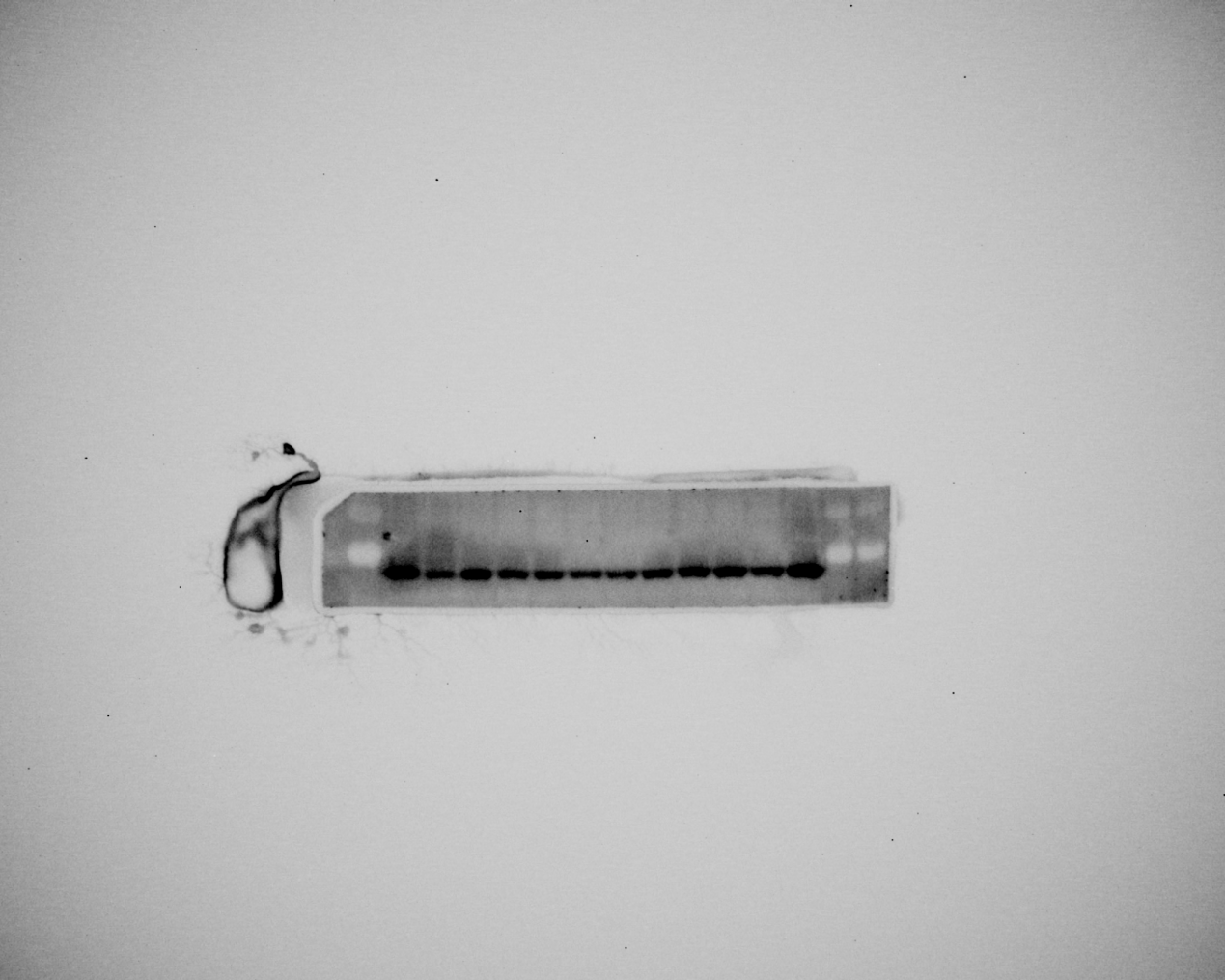


Figure SS4. Original image of AMPK in Figure 6 (A).


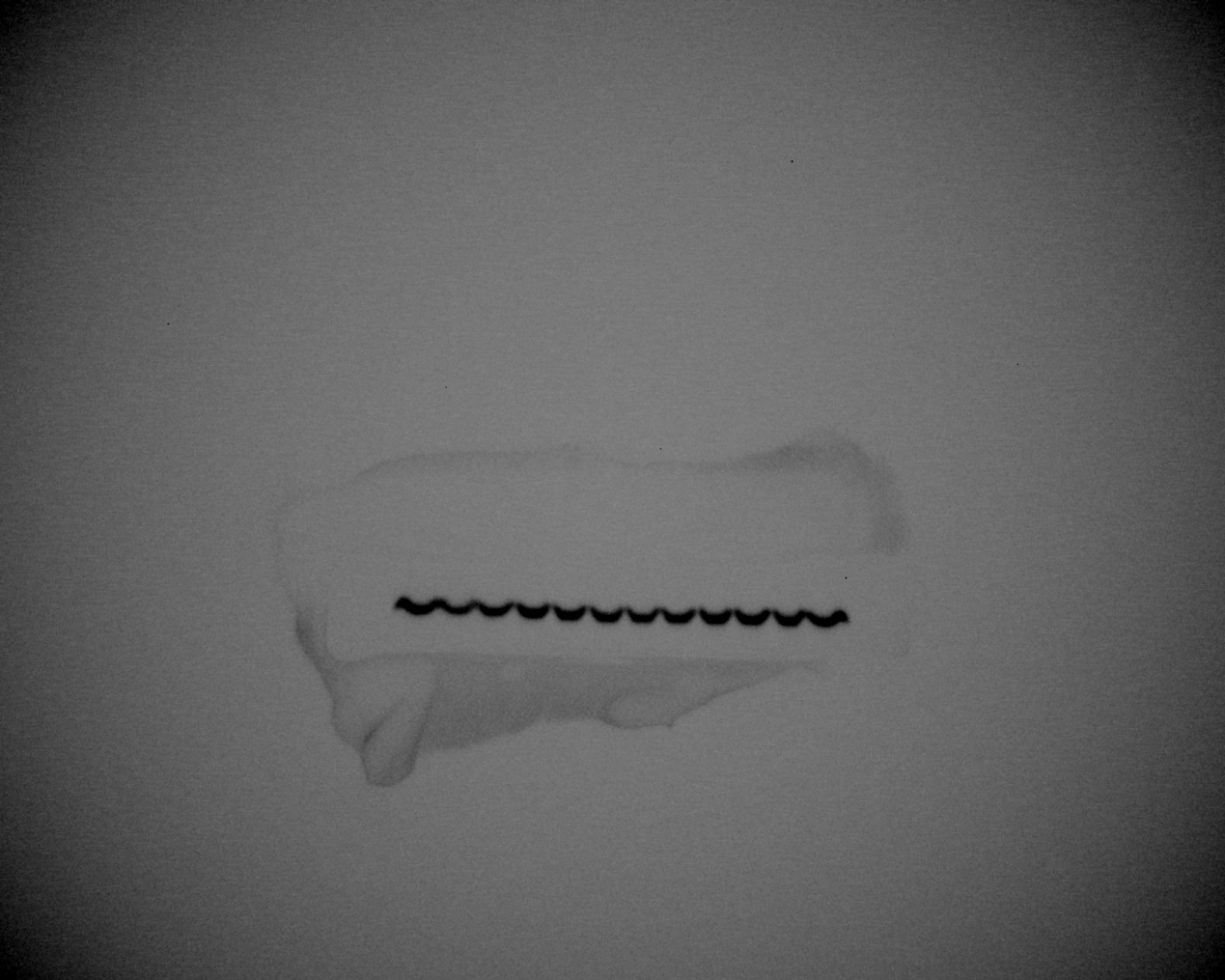


Figure SS5. Original image of GAPDH in Figure 6 (A).
