# Supplementary material for: Meroterpenoids From Ganoderma lucidum Mushrooms and Their Biological Roles in Insulin Resistance and Triple-Negative Breast Cancer
Source: Front Chem. 2021 Nov 3;9:772740. doi: 10.3389/fchem.2021.772740 (PMC8595597; doi:10.3389/fchem.2021.772740)
Supplement: Supplementary file 3 [file DataSheet5.docx]

Original Images Ⅱ

**Contents**

Figure SS6. Original image of Control at 0 h in Figure 7 (D).

Figure SS7. Original image of Control at 24 h in Figure 7 (D).

Figure SS8. Original image of (+)-**1** at 0 h in Figure 7 (D).

Figure SS9. Original image of (+)-**1** at 24 h in Figure 7 (D).

Figure SS10. Original image of (+)-**4** at 0 h in Figure 7 (D).

Figure SS11. Original image of (+)-**4** at 24 h in Figure 7 (D).

Figure SS12. Original image of (–)-**4** at 0 h in Figure 7 (D).

Figure SS13. Original image of (–)-**4** at 24 h in Figure 7 (D).

Figure SS14. Original image of Control at 0 h in Figure 7 (E).

Figure SS15. Original image of Control at 24 h in Figure 7 (E).

Figure SS16. Original image of 10 *μ*M at 0 h in Figure 7 (E).

Figure SS17. Original image of 10 *μ*M at 24 h in Figure 7 (E).

Figure SS18. Original image of 20 *μ*M at 0 h in Figure 7 (E).

Figure SS19. Original image of 20 *μ*M at 24 h in Figure 7 (E).

Figure SS20. Original image of 30 *μ*M at 0 h in Figure 7 (E).

Figure SS21. Original image of 30 *μ*M at 24 h in Figure 7 (E).


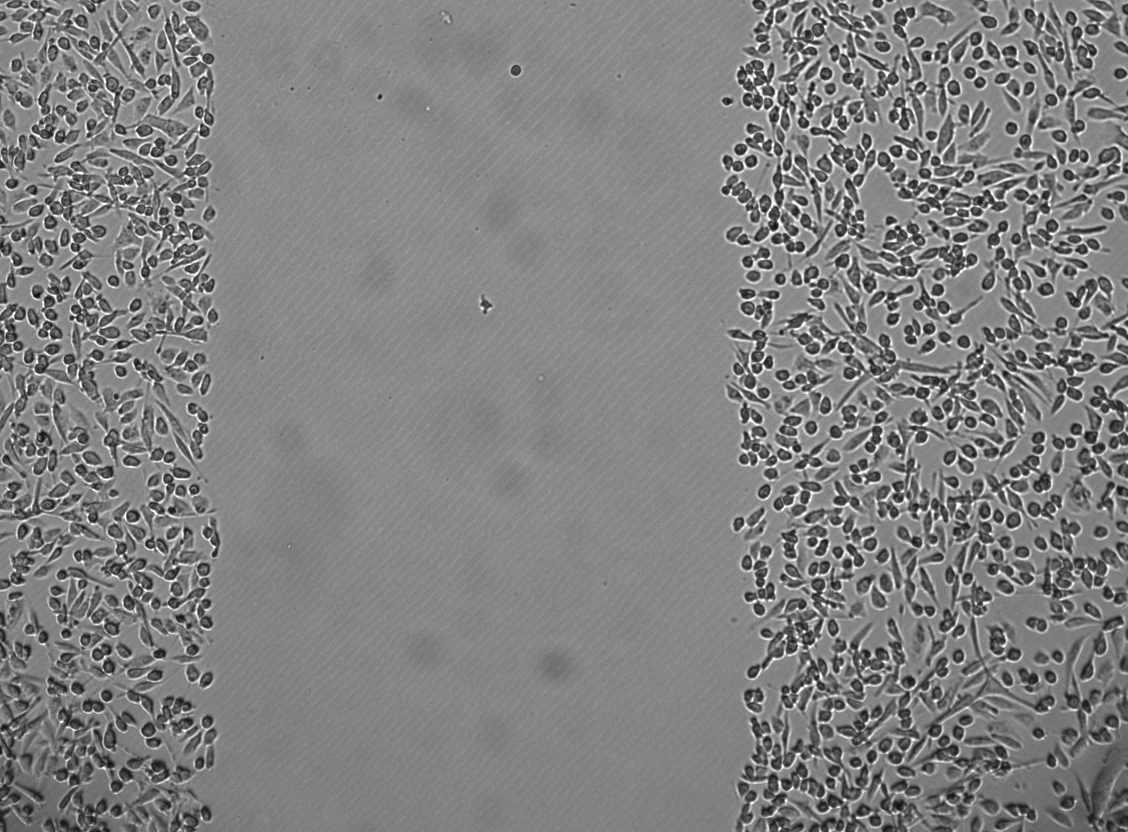


Figure SS6. Original image of Control at 0 h in Figure 7 (D).


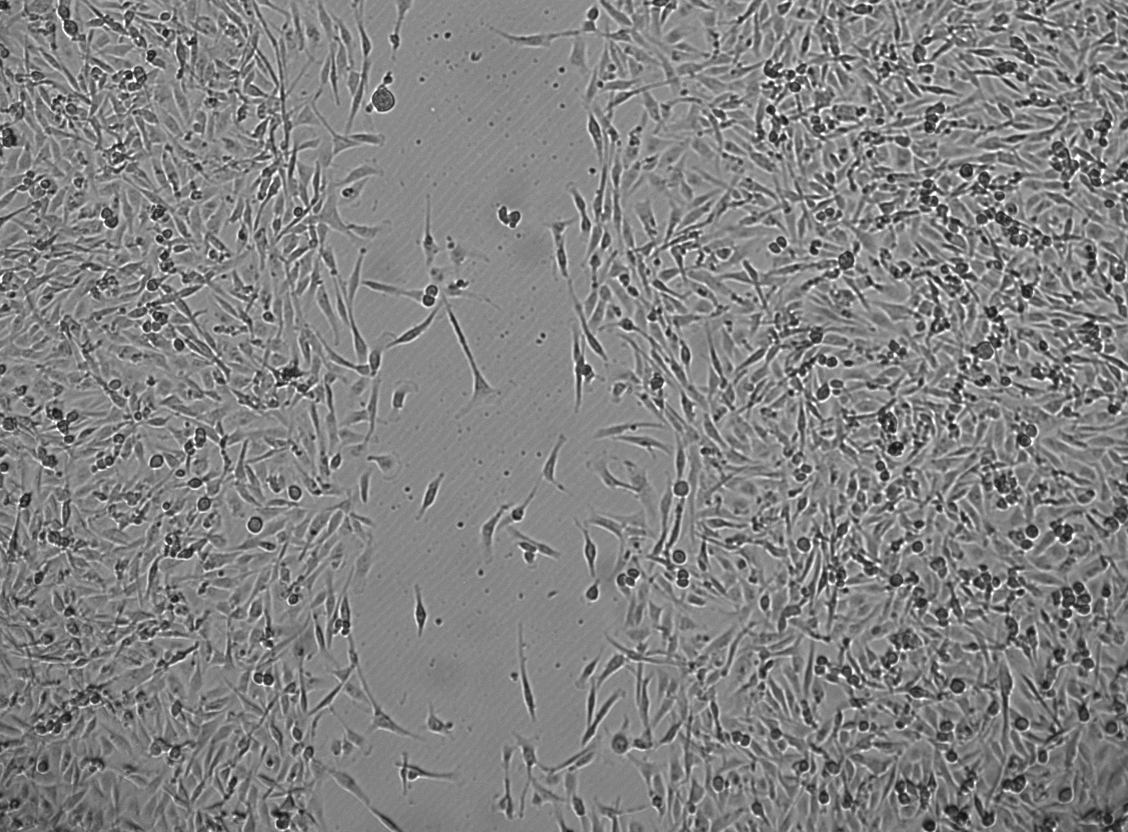


Figure SS7. Original image of Control at 24 h in Figure 7 (D).


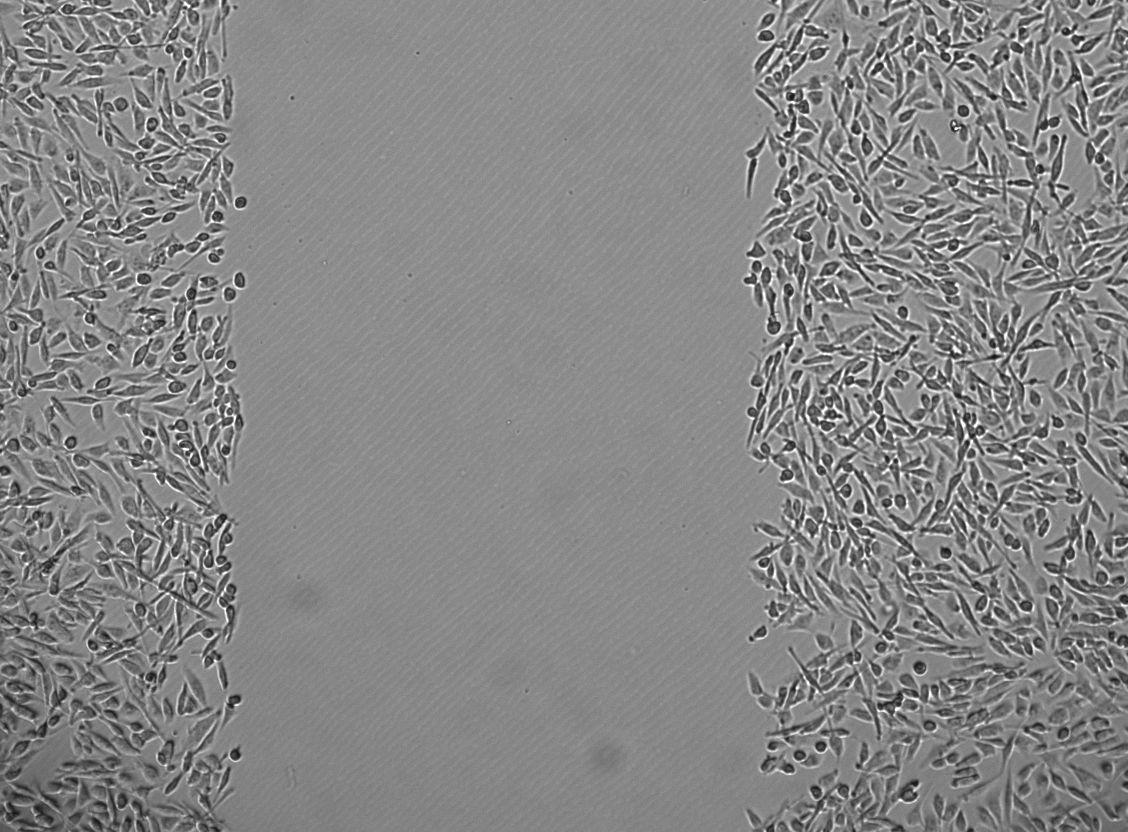


Figure SS8. Original image of (+)-**1** at 0 h in Figure 7 (D).


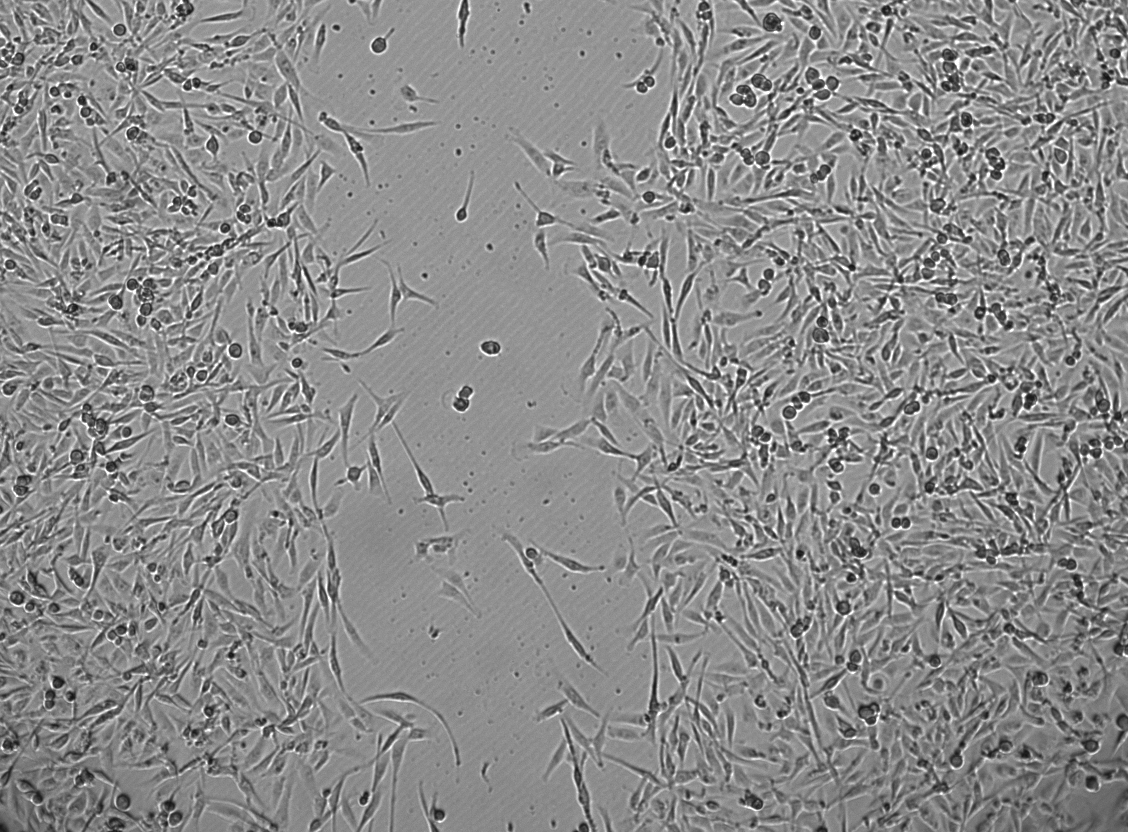


Figure SS9. Original image of (+)-**1** at 24 h in Figure 7 (D).


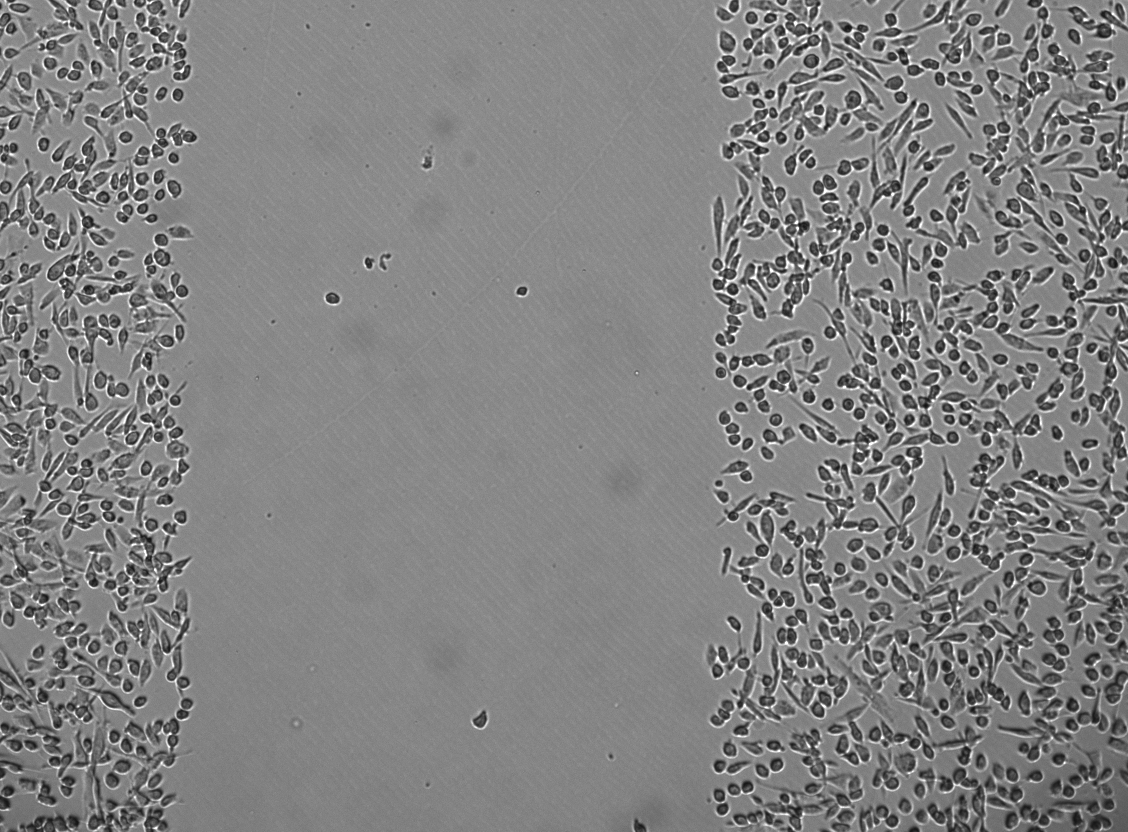


Figure SS10. Original image of (+)-**4** at 0 h in Figure 7 (D).


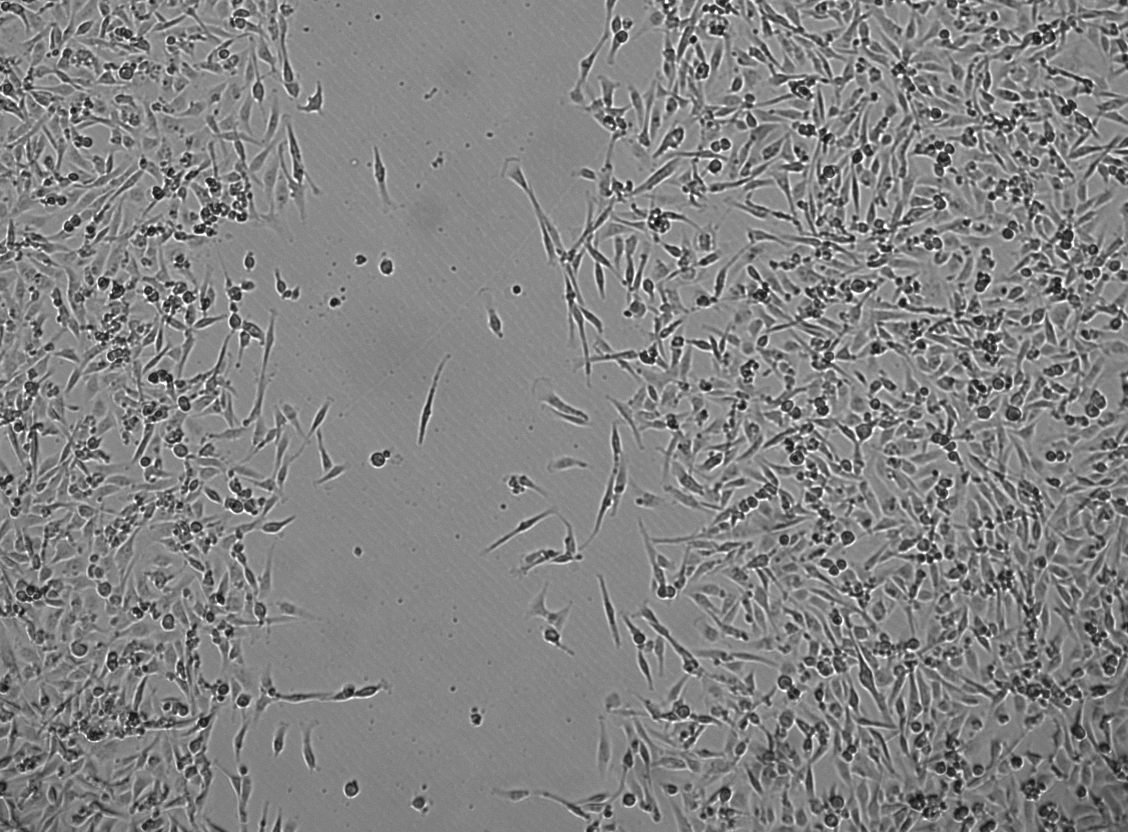


Figure SS11. Original image of (+)-**4** at 24 h in Figure 7 (D).


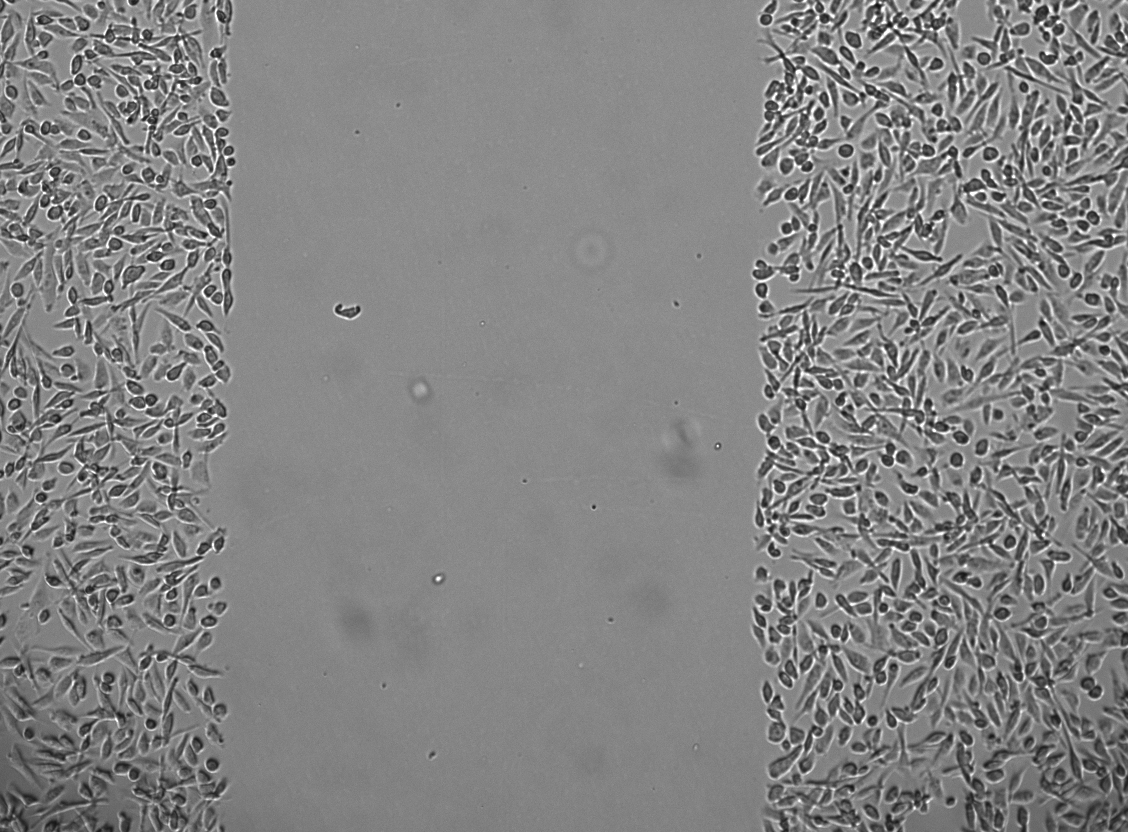


Figure SS12. Original image of (–)-**4** at 0 h in Figure 7 (D).


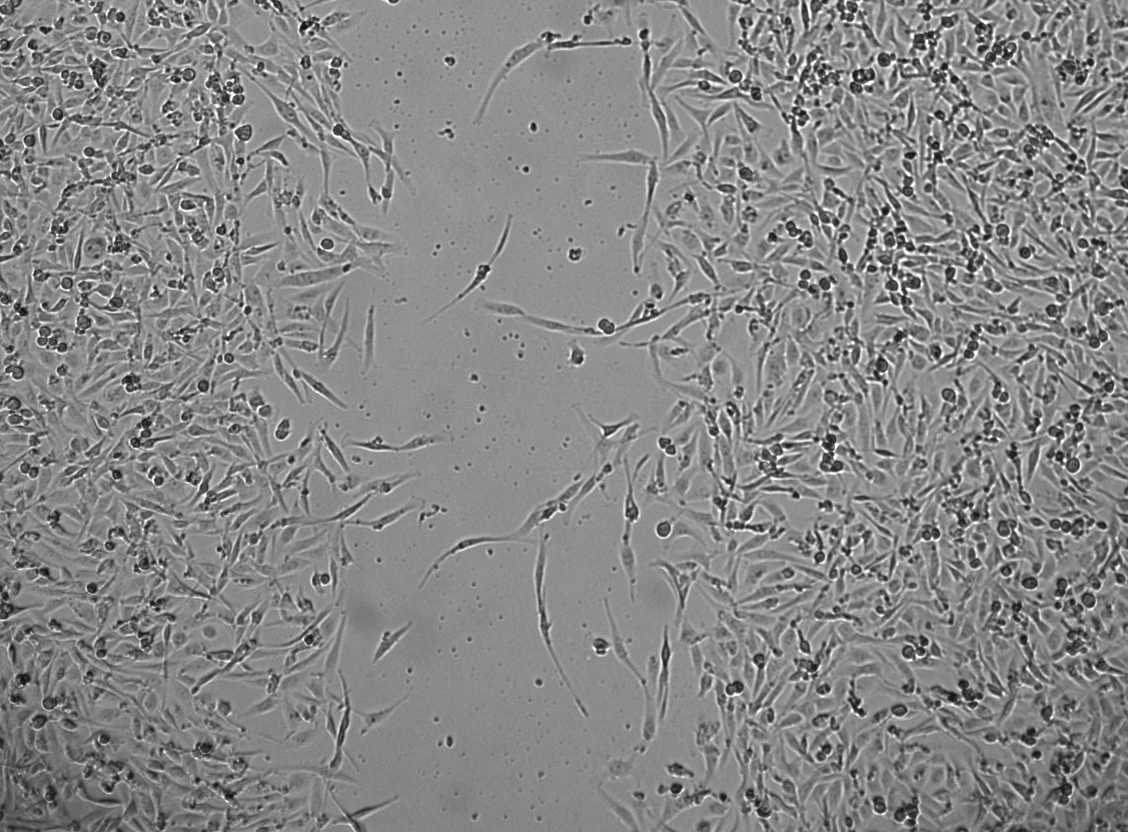


Figure SS13. Original image of (–)-**4** at 24 h in Figure 7 (D).


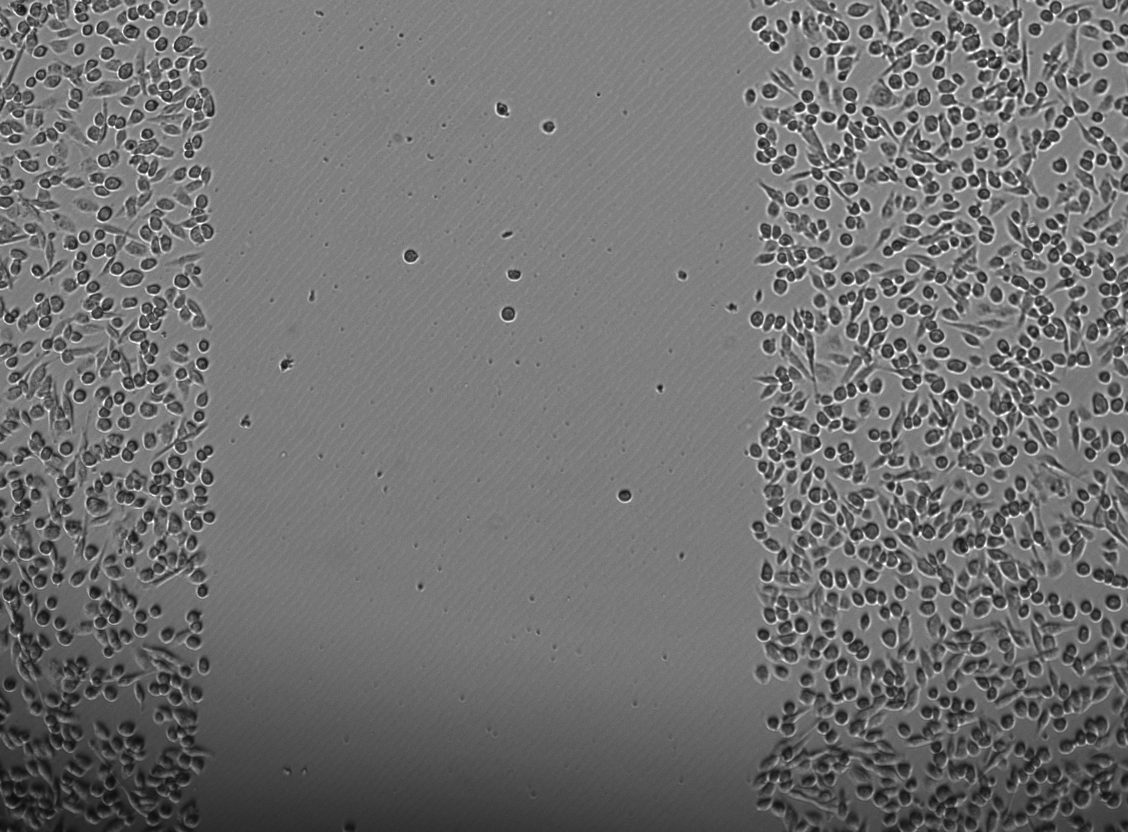


Figure SS14. Original image of Control at 0 h in Figure 7 (E).


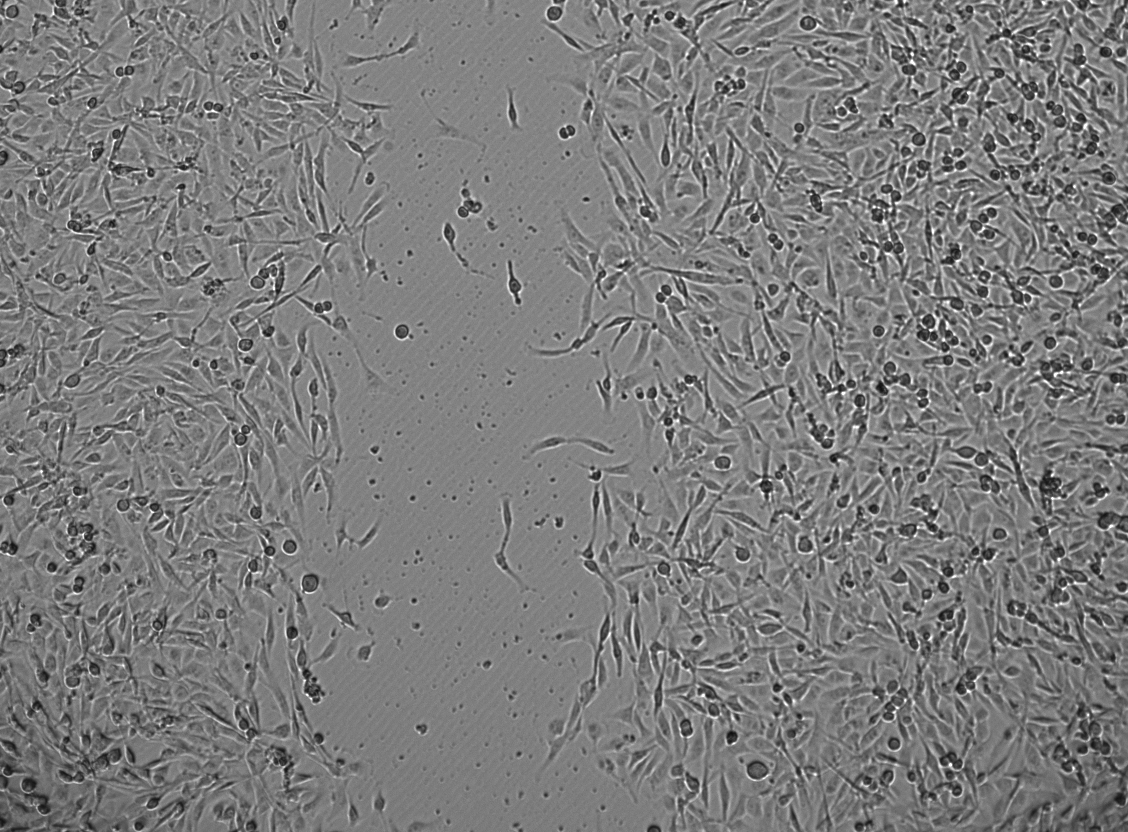


Figure SS15. Original image of Control at 24 h in Figure 7 (E).


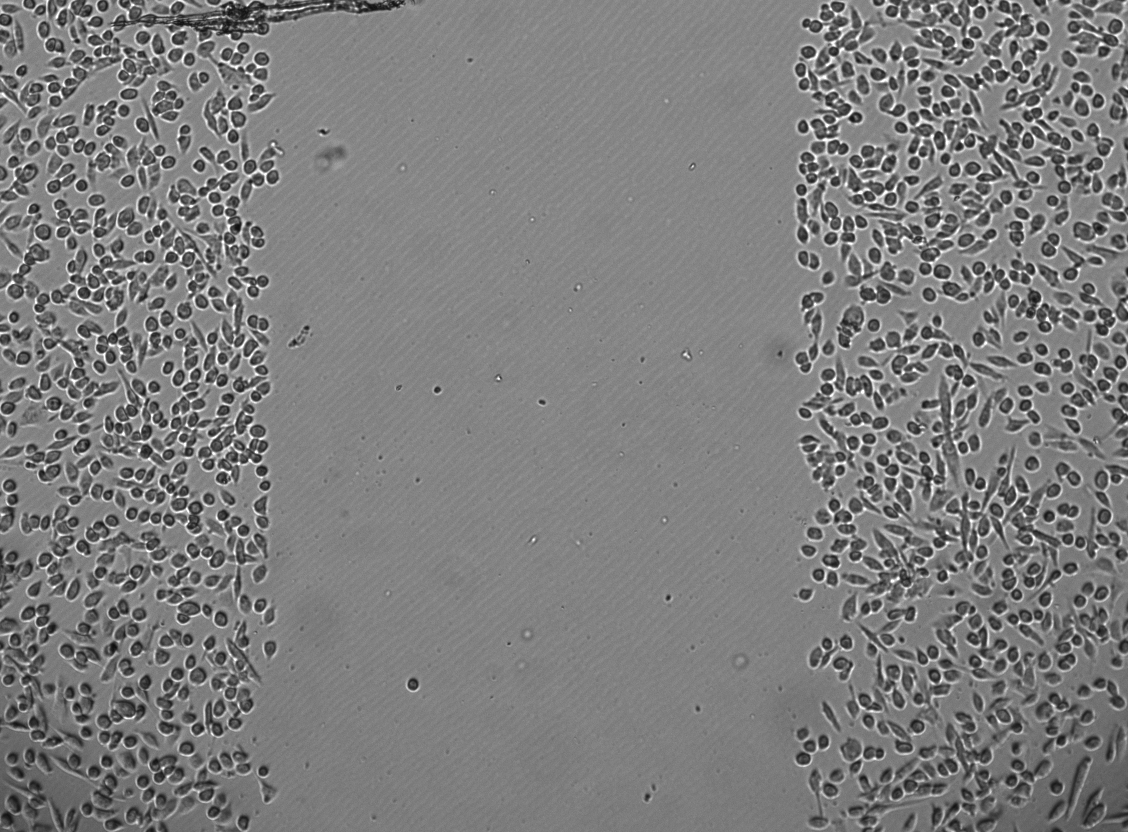


Figure SS16. Original image of 10 *μ*M at 0 h in Figure 7 (E).


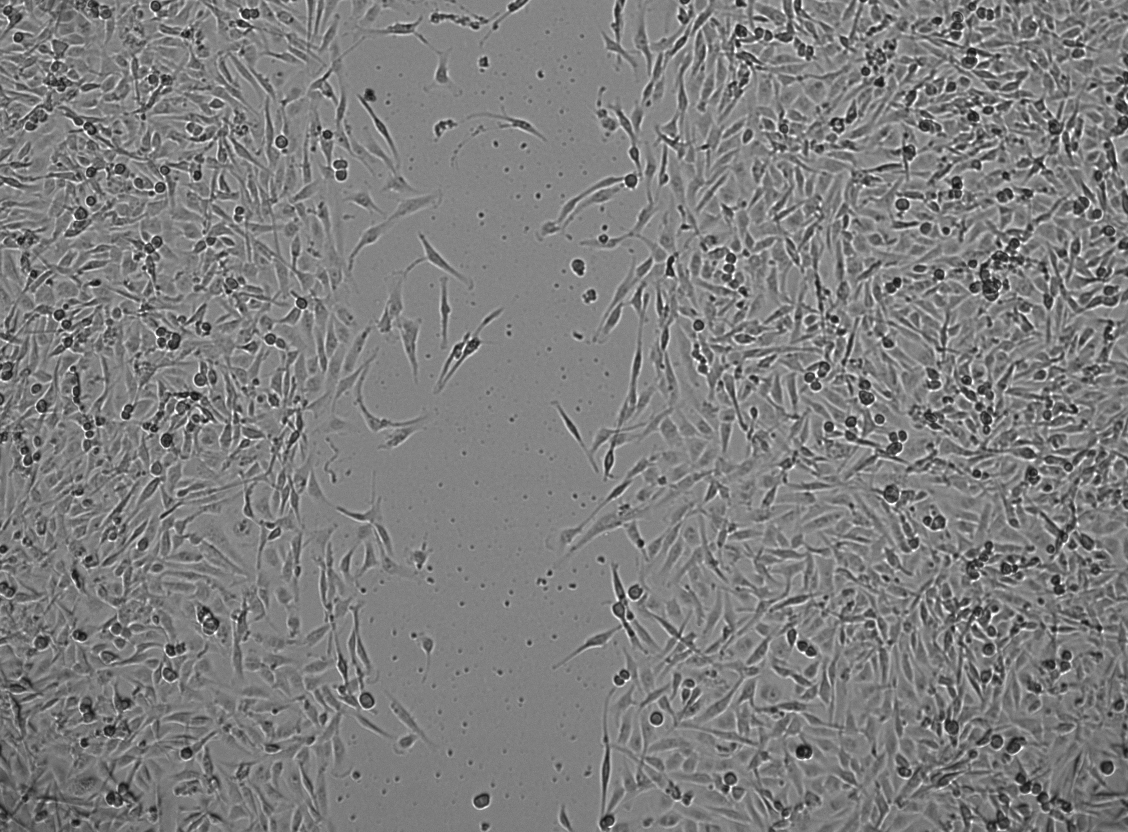


Figure SS17. Original image of 10 *μ*M at 24 h in Figure 7 (E).


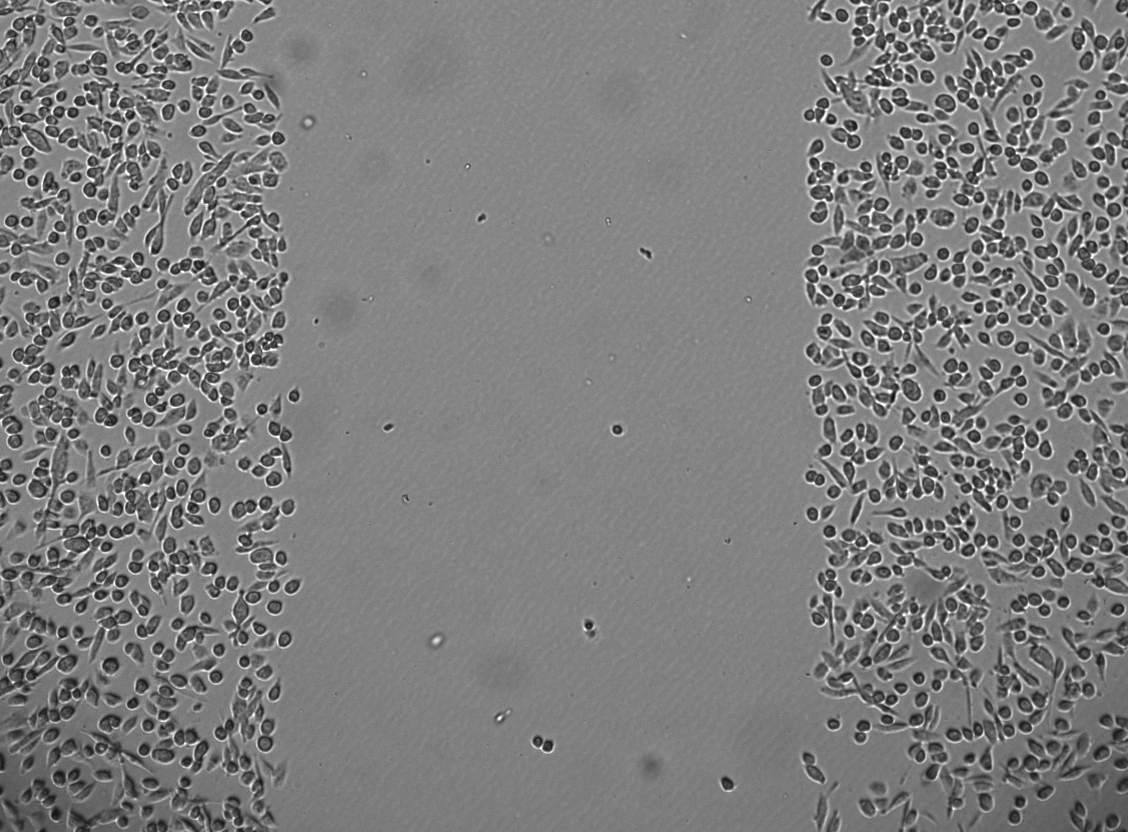


Figure SS18. Original image of 20 *μ*M at 0 h in Figure 7 (E).


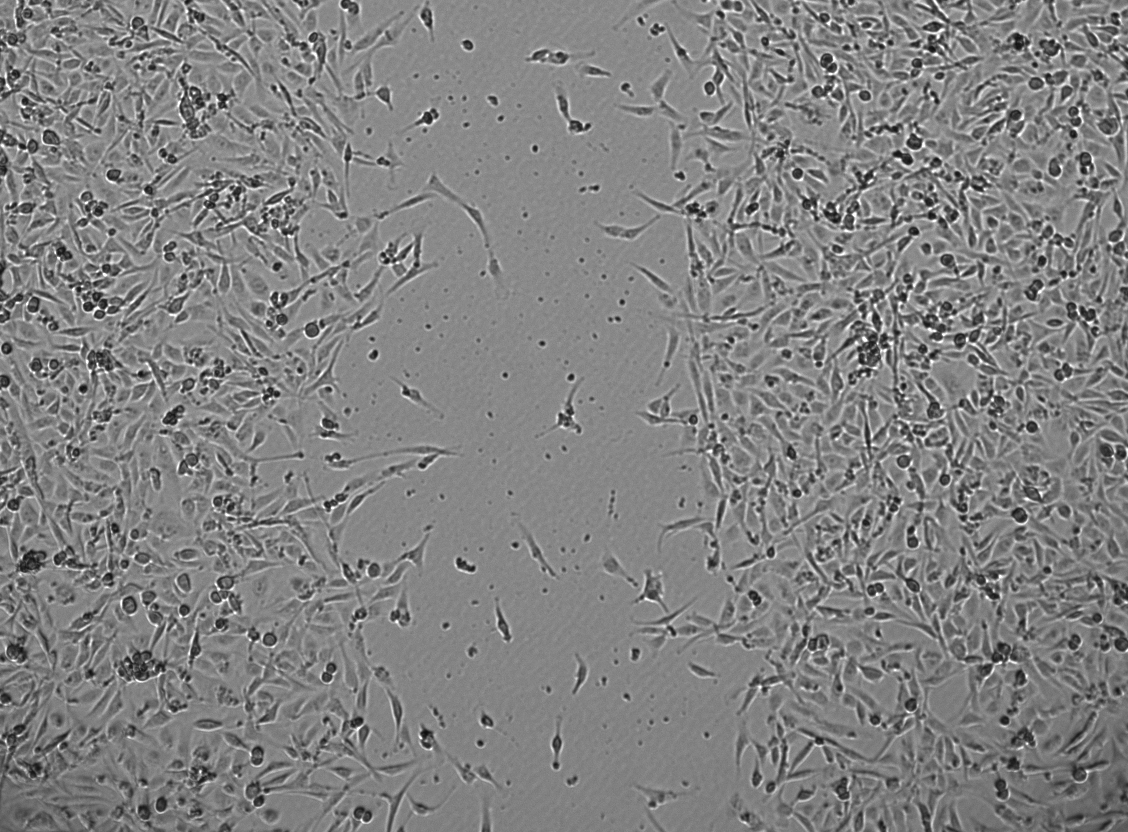


Figure SS19. Original image of 20 *μ*M at 24 h in Figure 7 (E).


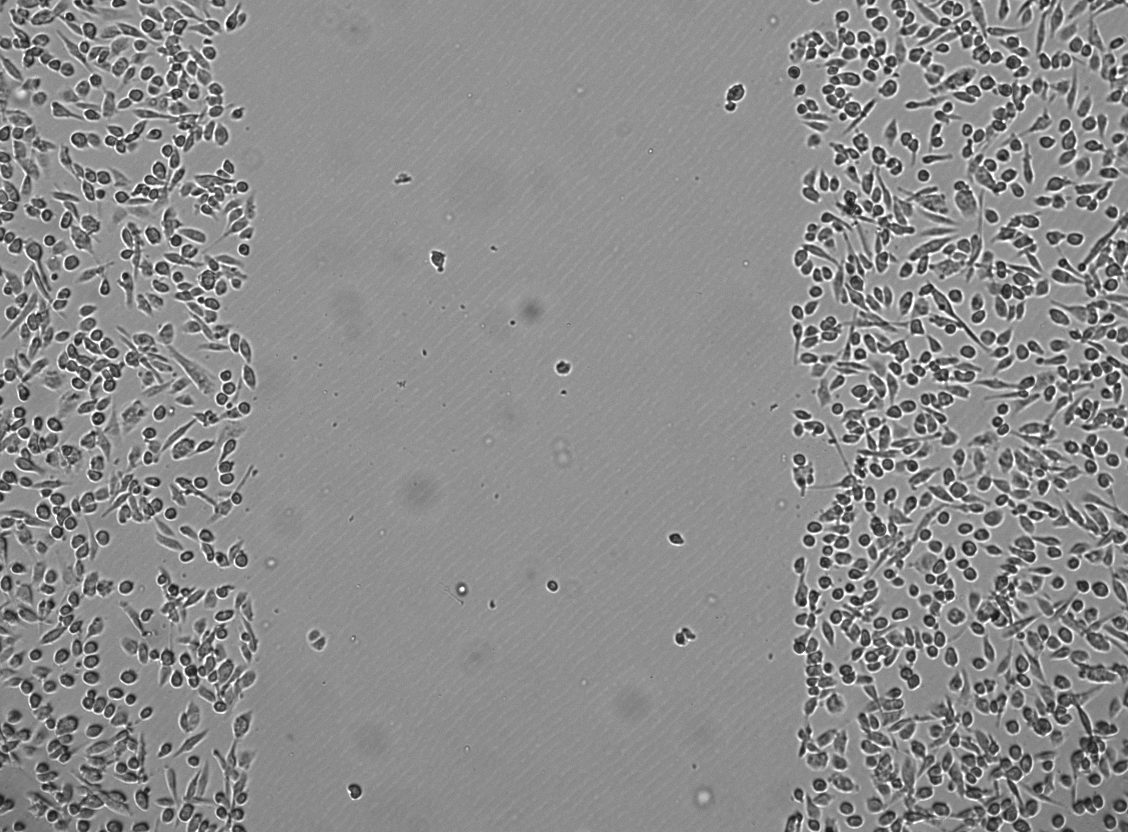


Figure SS20. Original image of 30 *μ*M at 0 h in Figure 7 (E).


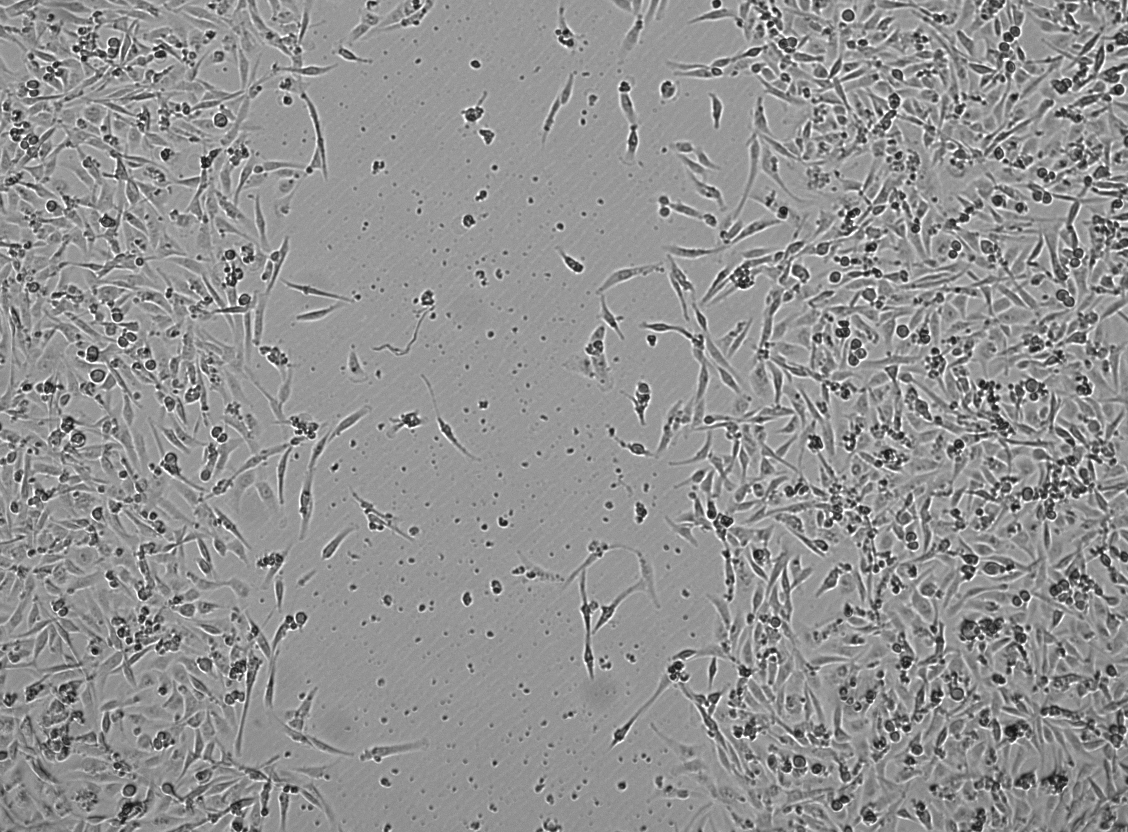


Figure SS21. Original image of 30 *μ*M at 24 h in Figure 7 (E).
