# Supplementary material for: Meroterpenoids From Ganoderma lucidum Mushrooms and Their Biological Roles in Insulin Resistance and Triple-Negative Breast Cancer
Source: Front Chem. 2021 Nov 3;9:772740. doi: 10.3389/fchem.2021.772740 (PMC8595597; doi:10.3389/fchem.2021.772740)
Supplement: Supplementary file 4 [file DataSheet3.doc]

Supplementary Material

# Meroterpenoids from *Ganoderma lucidum* mushrooms and their biological roles in insulin resistance and triple negative breast cancer

Jiao-Jiao Zhang^1,2^, Dai-Wei Wang^1^, Dan Cai^1^, Qing Lu^1,3^, Yong-Xian Cheng^1,3*^

^1^Institute for Inheritance-Based Innovation of Chinese Medicine, School of Pharmaceutical Sciences, Health Science Center, Shenzhen University, Shenzhen 518060, People’s Republic of China

^2^Institute of Microscale Optoelectronics, Shenzhen University, Shenzhen, 518060, People’s Republic of China

^3^Guangdong Key Laboratory for Functional Substances in Medicinal Edible Resources and Healthcare Products, School of Life Sciences and Food Engineering, Hanshan Normal University, Chaozhou 521041, People’s Republic of China

**Contents**

Figure S1. ^1^H NMR spectrum of **1** in methanol-*d*_4_

Figure S2. ^13^C NMR and DEPT spectra of **1** in methanol-*d*_4_

Figure S3. HSQC spectrum of **1** in methanol-*d*_4_

Figure S4. HMBC spectrum of **1** in methanol-*d*_4_

Figure S5. ^1^H-^1^H COSY spectrum of **1** in methanol-*d*_4_

Figure S6. ROESY spectrum of **1** in methanol-*d*_4_

Figure S7. ^1^H NMR spectrum of **1** in DMSO-*d*_6_

Figure S8. ROESY spectrum of **1** in DMSO-*d*_6_

Figure S9. HRESIMS of **1**

Figure S10. ^1^H NMR spectrum of **2** in methanol-*d*_4_

Figure S11. ^13^C NMR and DEPT spectra of **2** in methanol-*d*_4_

Figure S12. HSQC spectrum of **2** in methanol-*d*_4_

Figure S13. HMBC spectrum of **2** in methanol-*d*_4_

Figure S14. ^1^H-^1^H COSY spectrum of **2** in methanol-*d*_4_

Figure S15. ROESY spectrum of **2** in methanol-*d*_4_

Figure S16. HRESIMS of **2**

Figure S17. ^1^H NMR spectrum of **3** in methanol-*d*_4_

Figure S18. ^13^C NMR and DEPT spectra of **3** in methanol-*d*_4_

Figure S19. HSQC spectrum of **3** in methanol-*d*_4_

Figure S20. HMBC spectrum of **3** in methanol-*d*_4_

Figure S21. ^1^H-^1^H COSY spectrum of **3** in methanol-*d*_4_

Figure S22. ROESY spectrum of **3** in methanol-*d*_4_

Figure S23. HRESIMS of **3**

Figure S24. CD and UV spectra of (+)-**3**

Figure S25. CD and UV spectra of (–)-**3**

Figure S26. ^1^H NMR spectrum of **4** in methanol-*d*_4_

Figure S27. ^13^C NMR and DEPT spectra of **4** in methanol-*d*_4_

Figure S28. HSQC spectrum of **4** in methanol-*d*_4_

Figure S29. HMBC spectrum of **4** in methanol-*d*_4_

Figure S30. ^1^H-^1^H COSY spectrum of **4** in methanol-*d*_4_

Figure S31. ROESY spectrum of **4** in methanol-*d*_4_

Figure S32. HRESIMS of **4**

Figure S33. CD spectrum of (+)-**4**

Figure S34. CD spectrum of (–)-**4**

Figure S35. ^1^H NMR spectrum of **5** in methanol-*d*_4_

Figure S36. ^13^C NMR and DEPT spectra of **5** in methanol-*d*_4_

Figure S37. HSQC spectrum of **5** in methanol-*d*_4_

Figure S38. HMBC spectrum of **5** in methanol-*d*_4_

Figure S39. ^1^H-^1^H COSY spectrum of **5** in methanol-*d*_4_

Figure S40. ROESY spectrum of **5** in methanol-*d*_4_

Figure S41. HRESIMS of **5**

Figure S42. ^13^C NMR calculated structures of **1**

Figure S43. Optimized geometries of dominant conformers of **1-1** at the B3LYP/6-31(d,p) level in the gas phase

Table S1. Conformational analysis of the optimized **1-1** at the B3LYP/6-31(d,p) level in the gas phase.

Figure S44. Optimized geometries of dominant conformers of **1-2** at the B3LYP/6-31(d,p) level in the gas phase

Table S2. Conformational analysis of the optimized **1-2** at the B3LYP/6-31(d,p) level in the gas phase.

Figure S45. Optimized geometries of dominant conformers of **1-3** at the B3LYP/6-31(d,p) level in the gas phase

Table S3. Conformational analysis of the optimized **1-3** at the B3LYP/6-31(d,p) level in the gas phase.

Figure S46. Optimized geometries of dominant conformers of **1-4** at the B3LYP/6-31(d,p) level in the gas phase

Table S4. Conformational analysis of the optimized **1-4** at the B3LYP/6-31(d,p) level in the gas phase.

Figure S47. Optimized geometries of dominant conformers of **1-5** at the B3LYP/6-31(d,p) level in the gas phase

Table S5. Conformational analysis of the optimized **1-5** at the B3LYP/6-31(d,p) level in the gas phase.

Figure S48. Optimized geometries of dominant conformers of **1-6** at the B3LYP/6-31(d,p) level in the gas phase

Table S6. Conformational analysis of the optimized **1-6** at the B3LYP/6-31(d,p) level in the gas phase.

Figure S49. Regression analysis of experimental versus calculated ^13^C NMR chemical shifts of **1-1**-**1-6** at the B3LYP/6-311g(d,p) level; linear fitting is shown as a line.

Table S7. Comparison of ^13^C NMR experimental values and calculated chemical shifts of **1-1**.

Table S8. Comparison of ^13^C NMR experimental values and calculated chemical shifts of **1-2**.

Table S9. Comparison of ^13^C NMR experimental values and calculated chemical shifts of **1-3**.

Table S10. Comparison of ^13^C NMR experimental values and calculated chemical shifts of **1-4**.

Table S11. Comparison of ^13^C NMR experimental values and calculated chemical shifts of **1-5**.

Table S12. Comparison of ^13^C NMR experimental values and calculated chemical shifts of **1-6**.

Figure S50. DP4+ analysis of experimental NMR data of **1** and shielding tensors of **1-1**-**1-6** (Isomer 1-6)

Figure S51. Comparison of the calculated ECD spectrum of (9*S*,13*R*,14*S*)-**1** at B3LYP/6-311G(d,p) level with the experimental one in MeOH

Figure S52. Comparison of the calculated ECD spectrum of (9*S*)-**2** at B3LYP/6-311G(d,p) level with the experimental one in MeOH

Figure S53. Comparison of the calculated ECD spectrum of (9*S*)-**5** at B3LYP/6-311G(d,p) level with the experimental one in MeOH

Table S13. Specific Optical Rotation for compound **1**.

Figure S54. C2C12 cell proliferation in response to compounds at 20 *μ*M by CCK-8 assay. ^****^*P* < 0.0001 compared with DMSO treatment group

Table S14. Cartesian Coordinates of conformers **1−1a** and **1−1b** in the gas phase (Å) at the B3LYP/6-31(d,p) level.

Table S15. Cartesian Coordinates of conformers **1−1c** and **1−1d** in the gas phase (Å) at the B3LYP/6-31(d,p) level.

Table S16. Cartesian Coordinates of conformers **1−1e** and **1−1f** in the gas phase (Å) at the B3LYP/6-31(d,p) level.

Table S17. Cartesian Coordinates of conformers **1−1g** and **1−1h** in the gas phase (Å) at the B3LYP/6-31(d,p) level.

Table S18. Cartesian Coordinates of conformers **1−1i** and **1−1j** in the gas phase (Å) at the B3LYP/6-31(d,p) level.

Table S19. Cartesian Coordinates of conformers **1−1k** and **1−1l** in the gas phase (Å) at the B3LYP/6-31(d,p) level.

Table S20. Cartesian Coordinates of conformers **1−2a** and **1−2b** in the gas phase (Å) at the B3LYP/6-31(d,p) level.

Table S21. Cartesian Coordinates of conformers **1−2c** and **1−2d** in the gas phase (Å) at the B3LYP/6-31(d,p) level.

Table S22. Cartesian Coordinates of conformers **1−2e** and **1−2f** in the gas phase (Å) at the B3LYP/6-31(d,p) level.

Table S23. Cartesian Coordinates of conformers **1−2g** and **1−2h** in the gas phase (Å) at the B3LYP/6-31(d,p) level.

Table S24. Cartesian Coordinates of conformers **1−2i** and **1−2j** in the gas phase (Å) at the B3LYP/6-31(d,p) level.

Table S25. Cartesian Coordinates of conformers **1−2k** and **1−2l** in the gas phase (Å) at the B3LYP/6-31(d,p) level.

Table S26. Cartesian Coordinates of conformers **1−3a** and **1−3b** in the gas phase (Å) at the B3LYP/6-31(d,p) level.

Table S27. Cartesian Coordinates of conformers **1−3c** and **1−3d** in the gas phase (Å) at the B3LYP/6-31(d,p) level.

Table S28. Cartesian Coordinates of conformers **1−3e** and **1−3f** in the gas phase (Å) at the B3LYP/6-31(d,p) level.

Table S29. Cartesian Coordinates of conformer **1−3g** in the gas phase (Å) at the B3LYP/6-31(d,p) level.

Table S30. Cartesian Coordinates of conformers **1−4a** and **1−4b** in the gas phase (Å) at the B3LYP/6-31(d,p) level.

Table S31. Cartesian Coordinates of conformers **1−4c** and **1−4d** in the gas phase (Å) at the B3LYP/6-31(d,p) level.

Table S32. Cartesian Coordinates of conformer **1−4e** in the gas phase (Å) at the B3LYP/6-31(d,p) level.

Table S33. Cartesian Coordinates of conformers **1−5a** and **1−5b** in the gas phase (Å) at the B3LYP/6-31(d,p) level.

Table S34. Cartesian Coordinates of conformers **1−5c** and **1−5d** in the gas phase (Å) at the B3LYP/6-31(d,p) level.

Table S35. Cartesian Coordinates of conformers **1−6a** and **1−6b** in the gas phase (Å) at the B3LYP/6-31(d,p) level.

Table S36. Cartesian Coordinates of conformers **1−6c** and **1−6d** in the gas phase (Å) at the B3LYP/6-31(d,p) level.


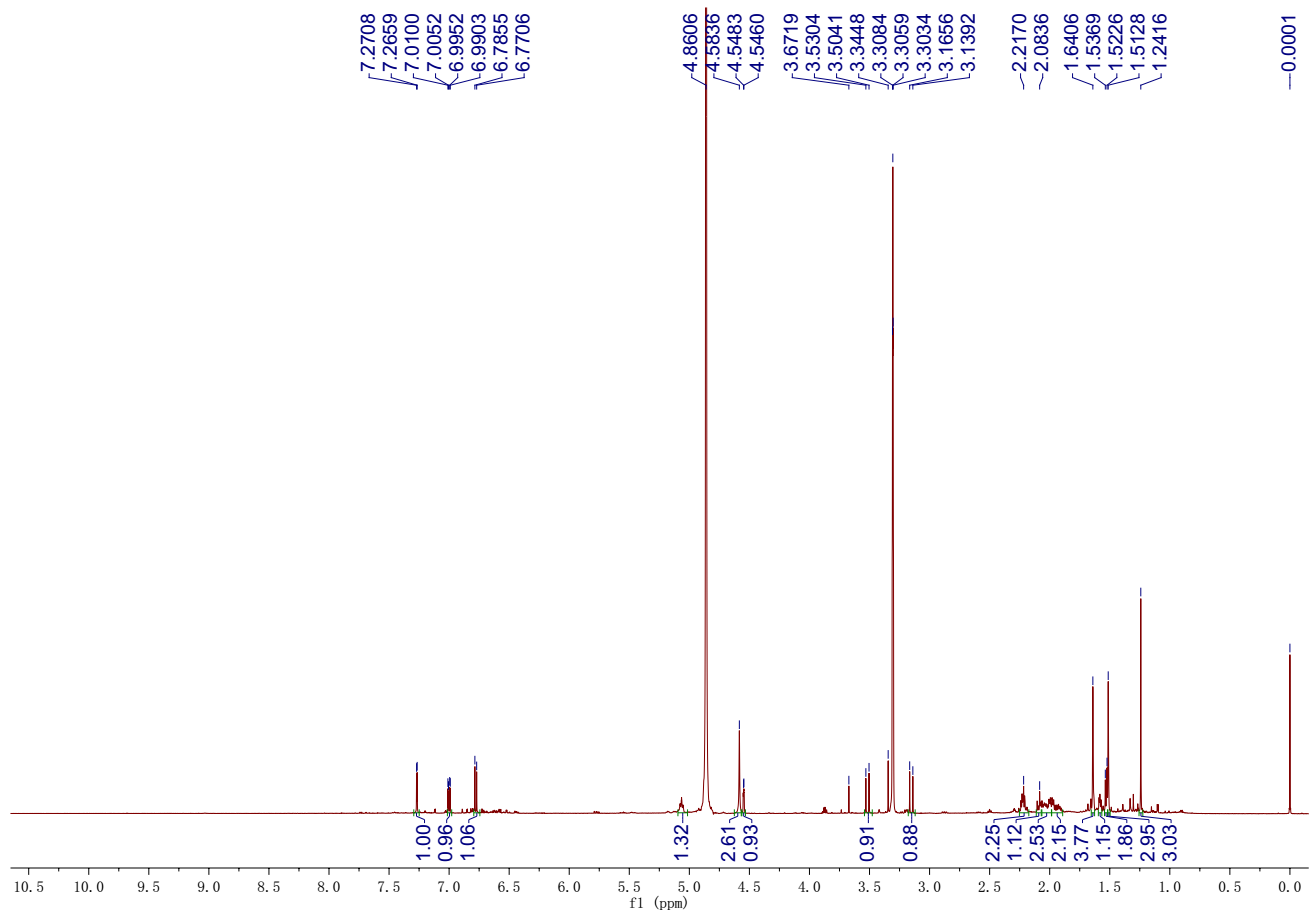


Figure S1. ^1^H NMR spectrum of **1** in methanol-*d*_4_


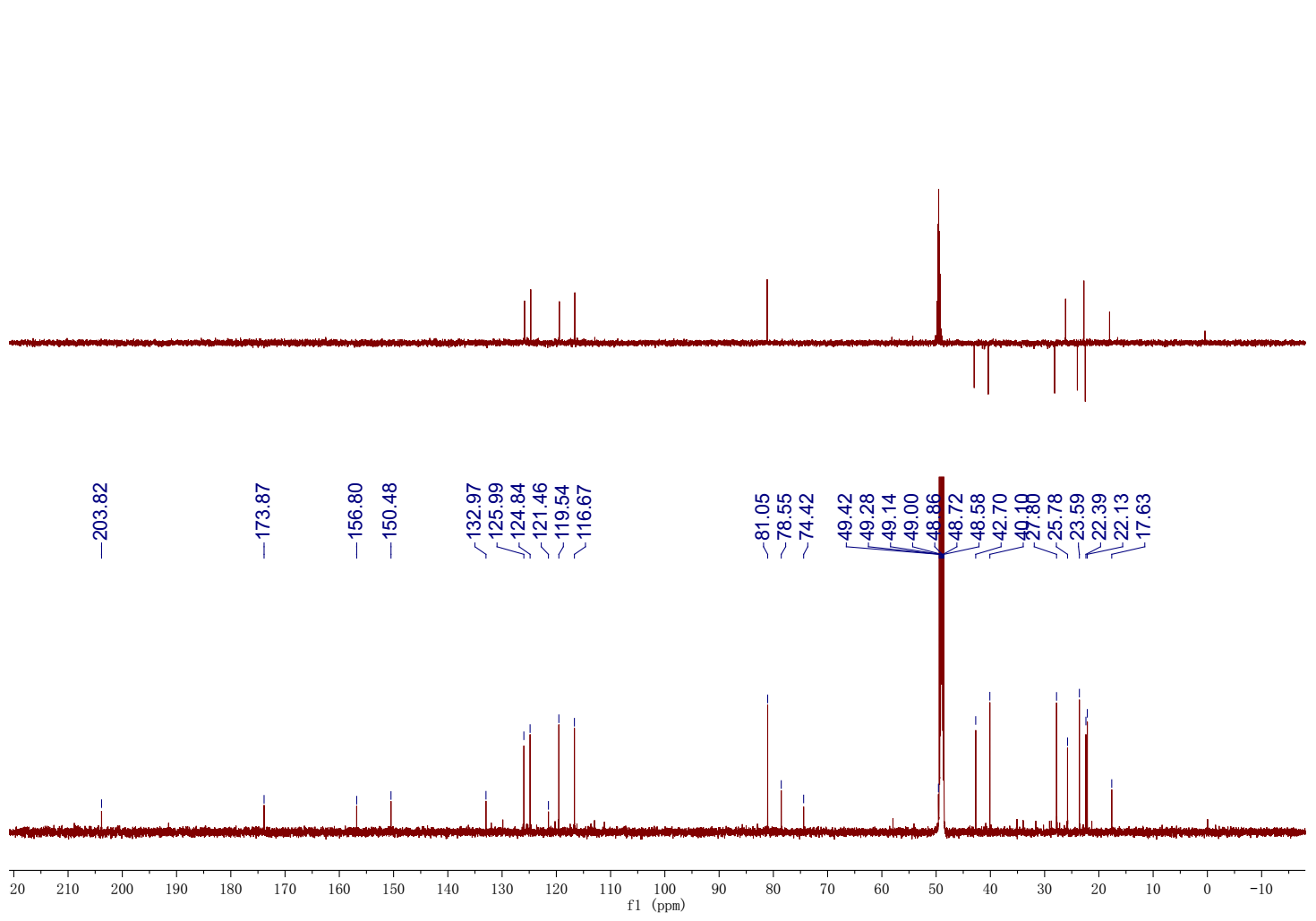


Figure S2. ^13^C NMR and DEPT spectra of **1** in methanol*-d*_4_


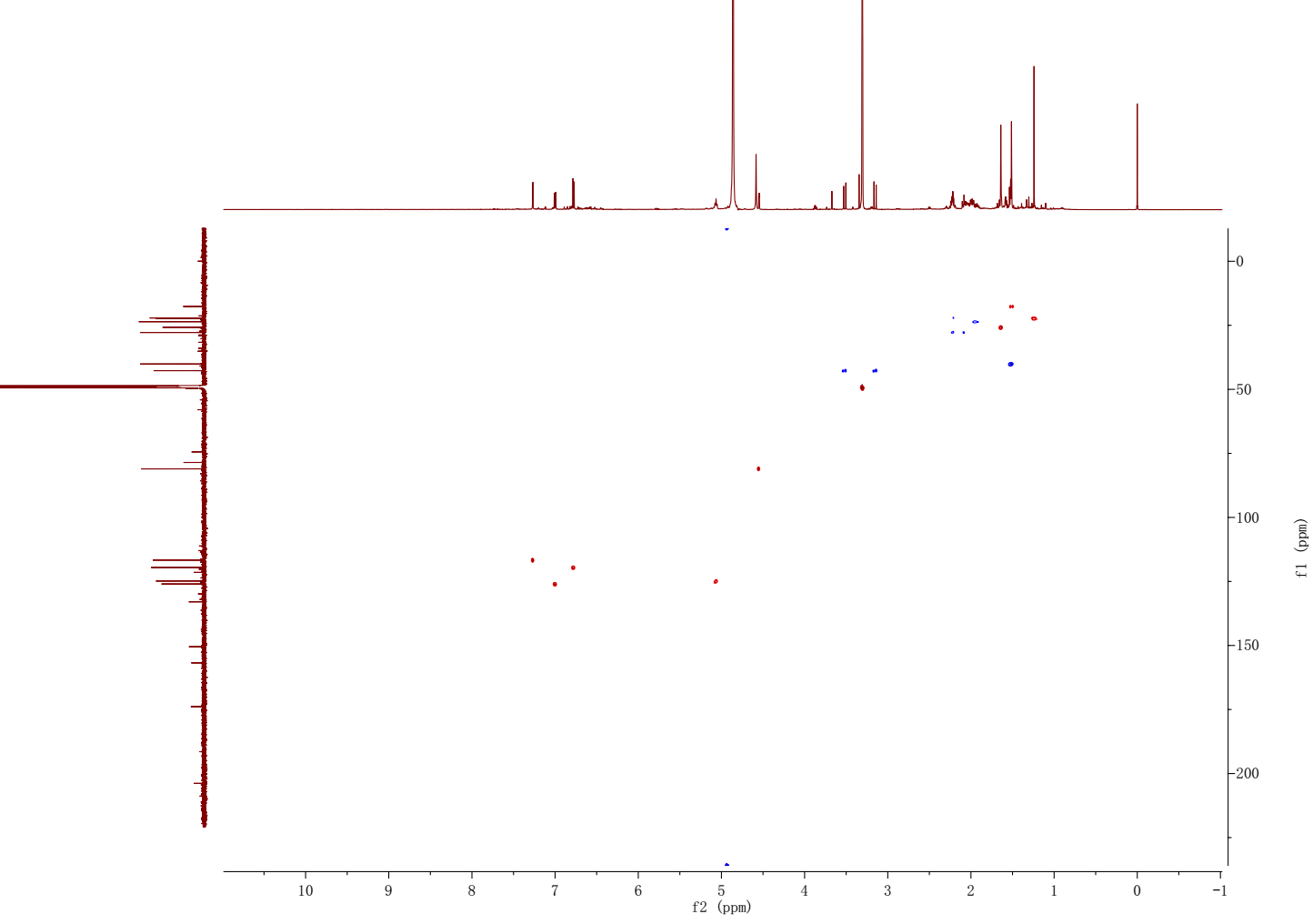


Figure S3. HSQC spectrum of **1** in methanol-*d*_4_


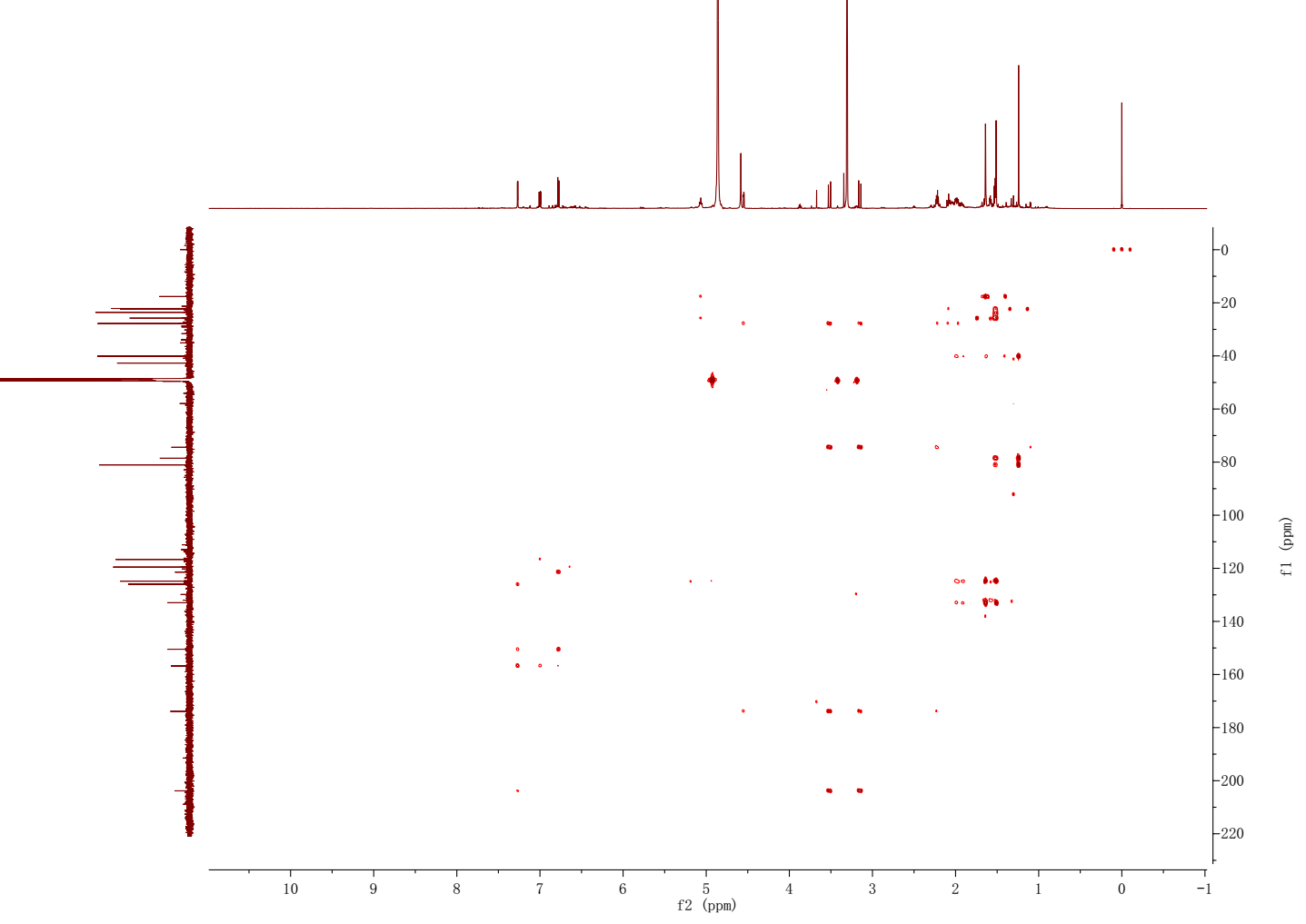


Figure S4. HMBC spectrum of **1** in methanol-*d*_4_


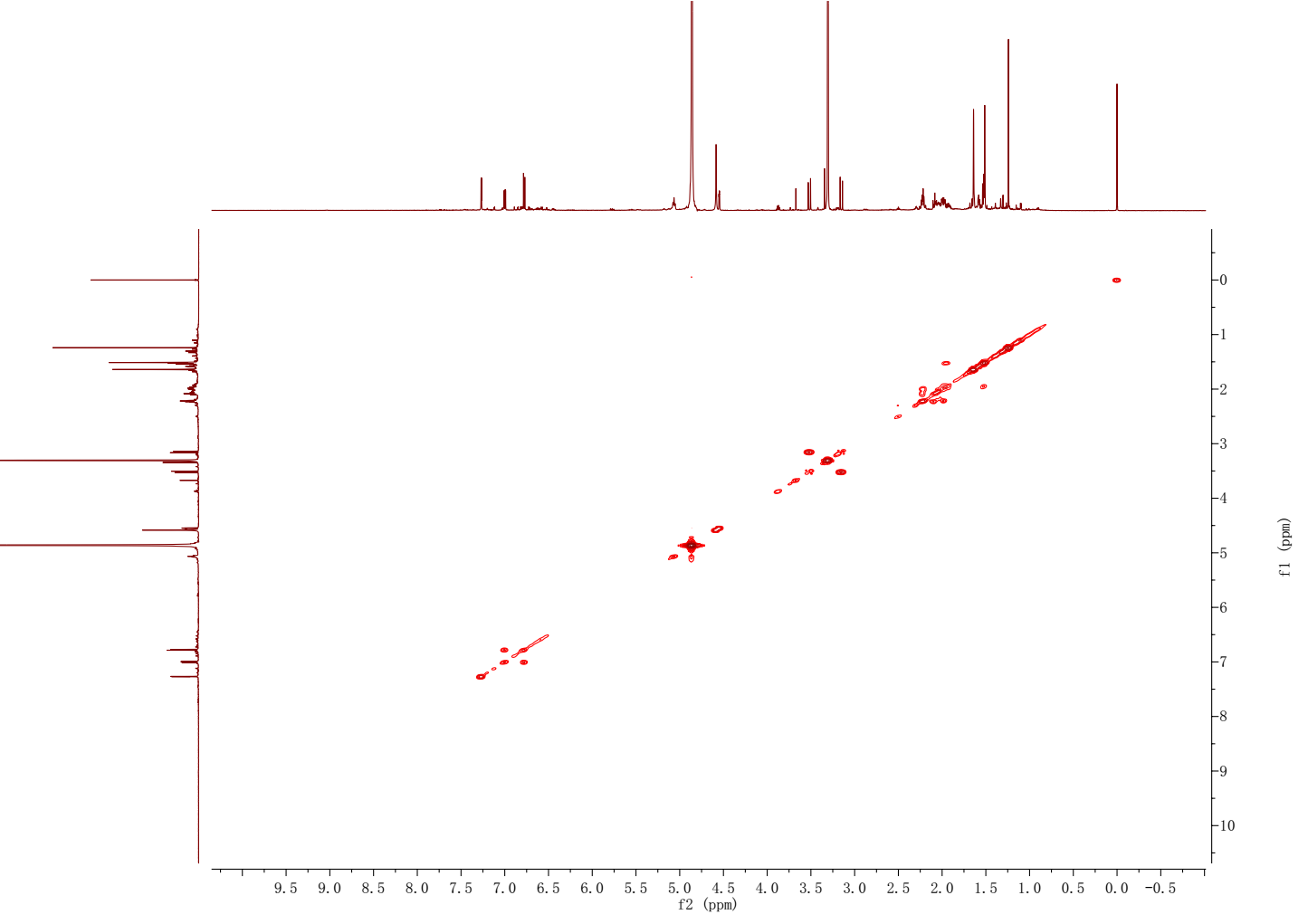


Figure S5. ^1^H-^1^H COSY spectrum of **1** in methanol-*d*_4_


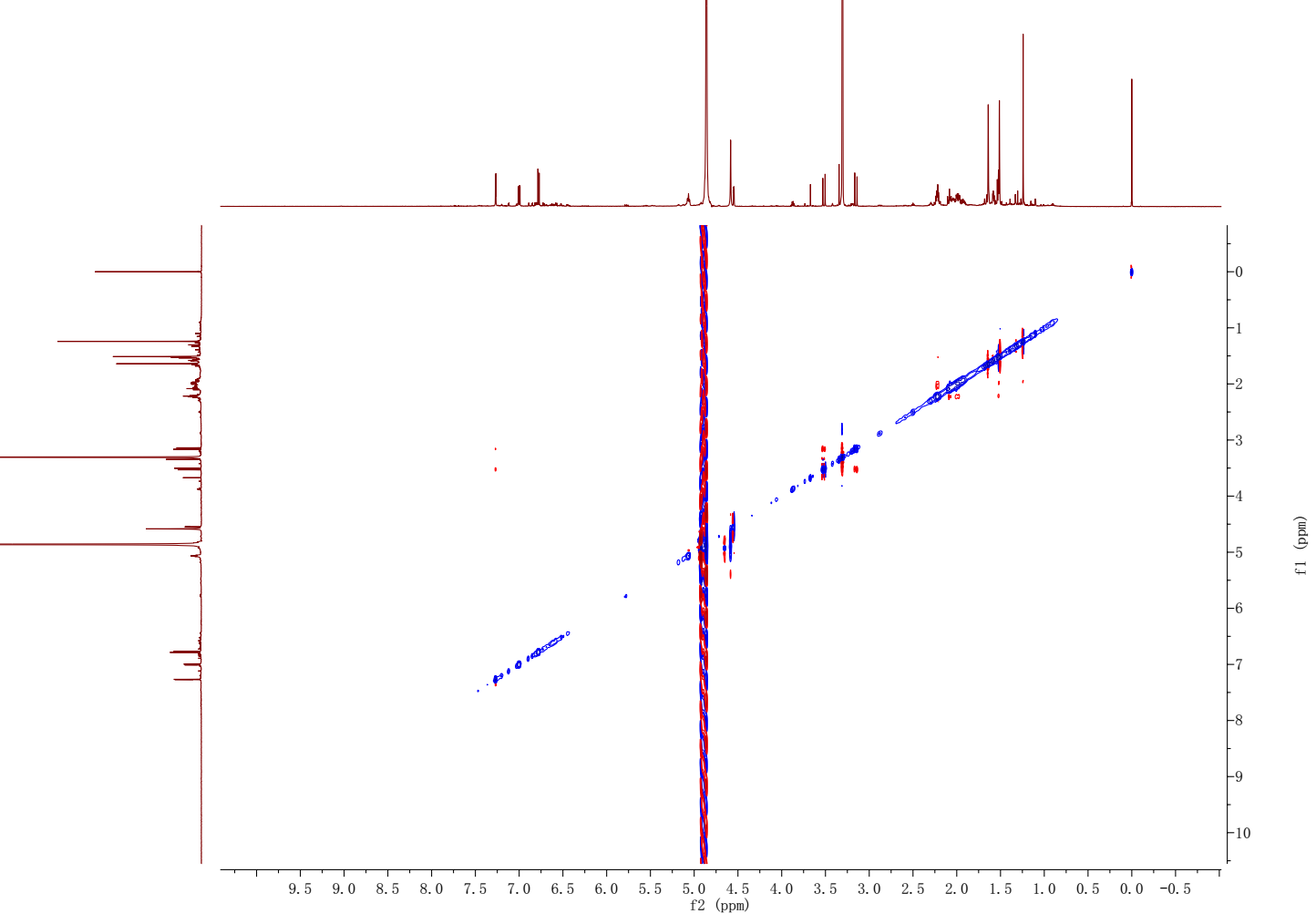


Figure S6. ROESY spectrum of **1** in methanol-*d*_4_


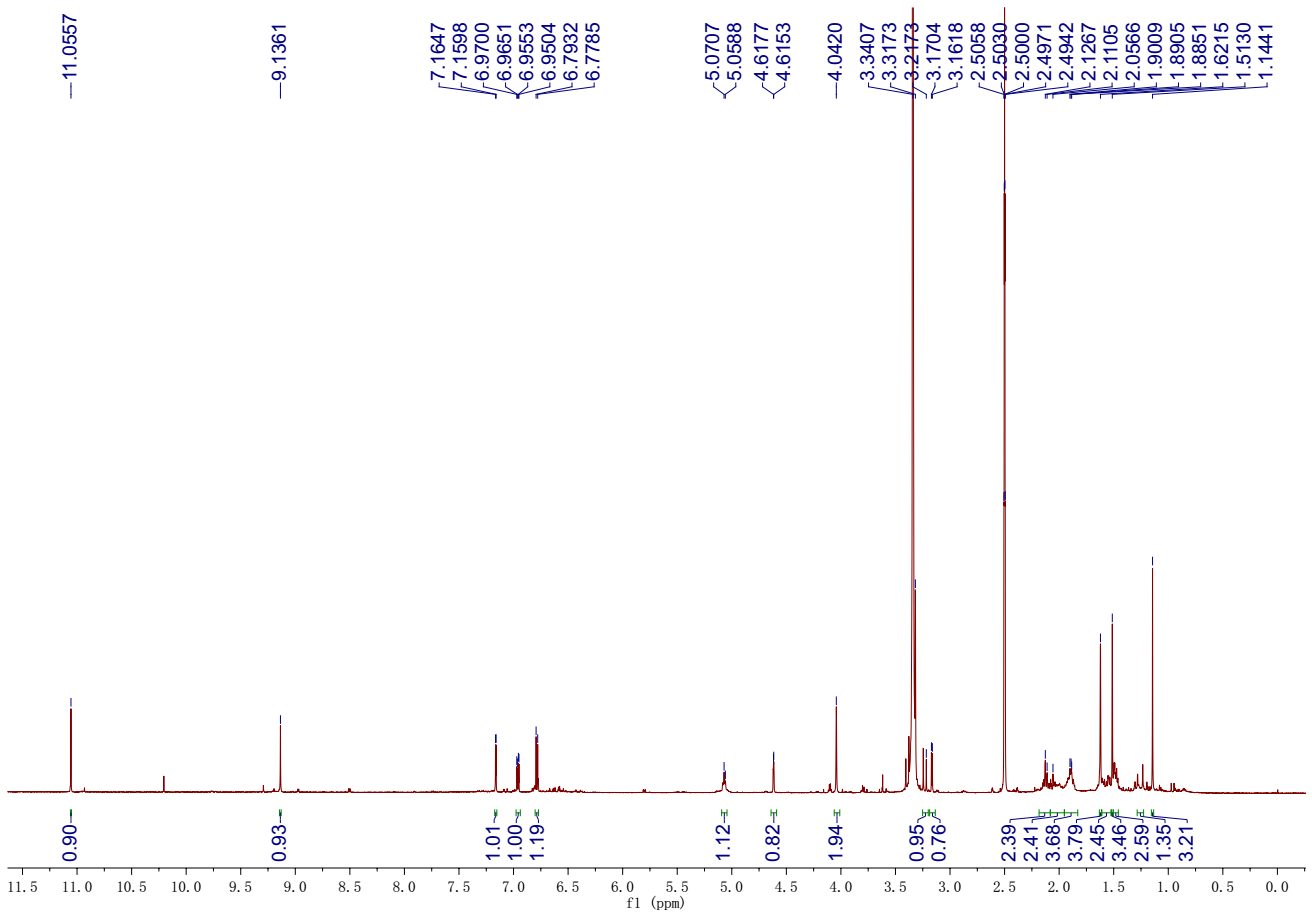


Figure S7. ^1^H NMR spectrum of **1** in DMSO-*d*_6_


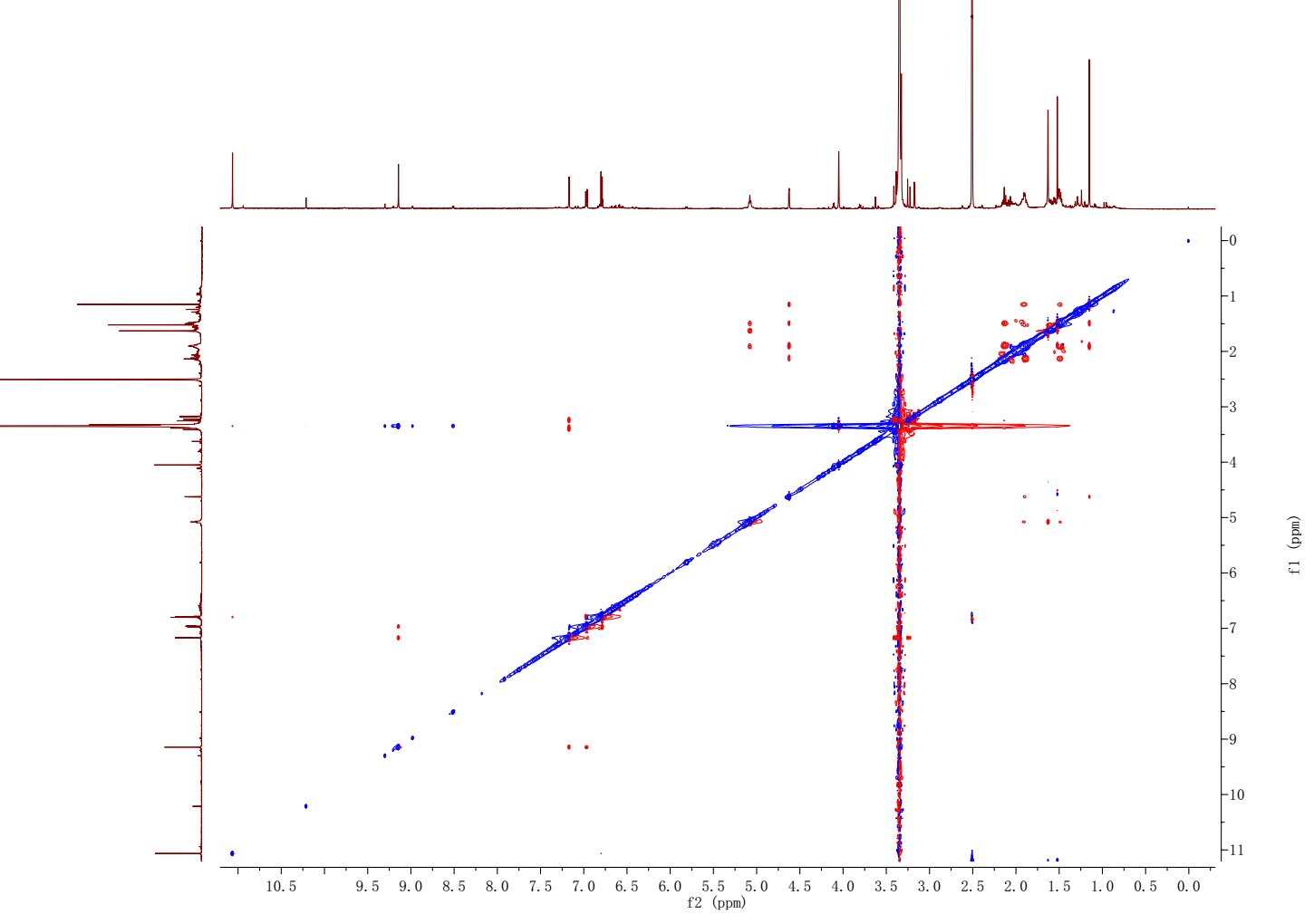


Figure S8. ROESY spectrum of **1** in DMSO-*d*_6_

Figure S9. HRESIMS of **1**


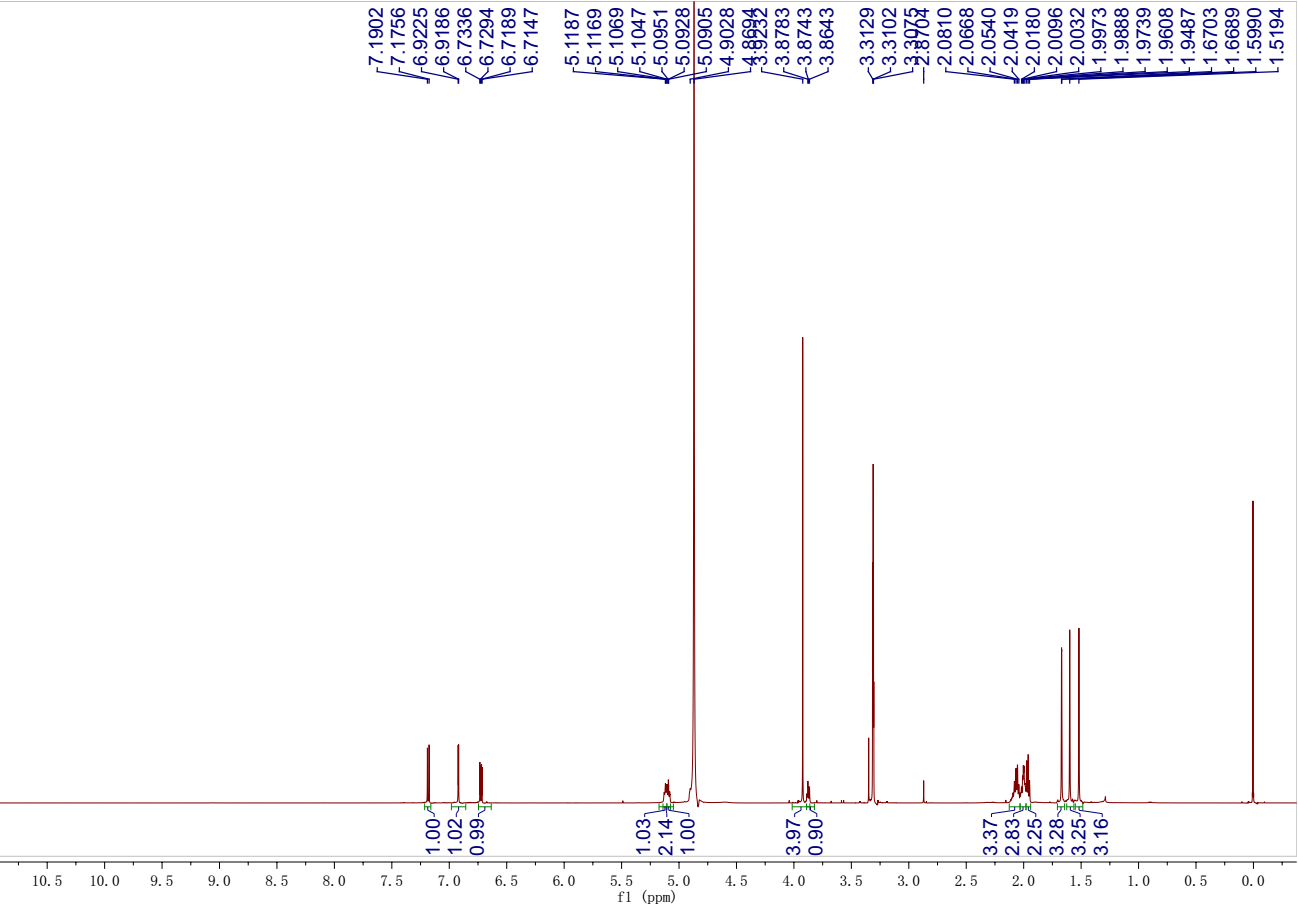


Figure S10. ^1^H NMR spectrum of **2** in methanol-*d*_4_


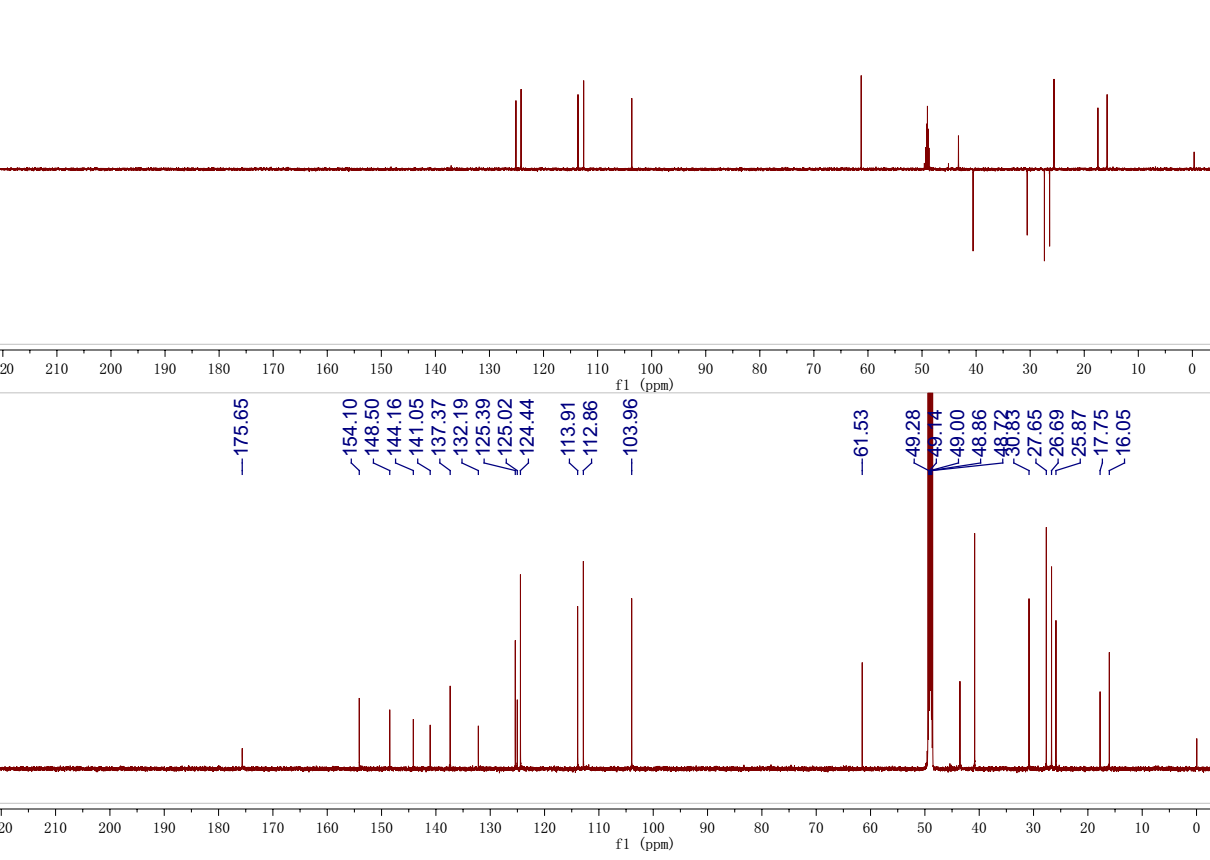


Figure S11. ^13^C NMR and DEPT spectra of **2** in methanol-*d*_4_


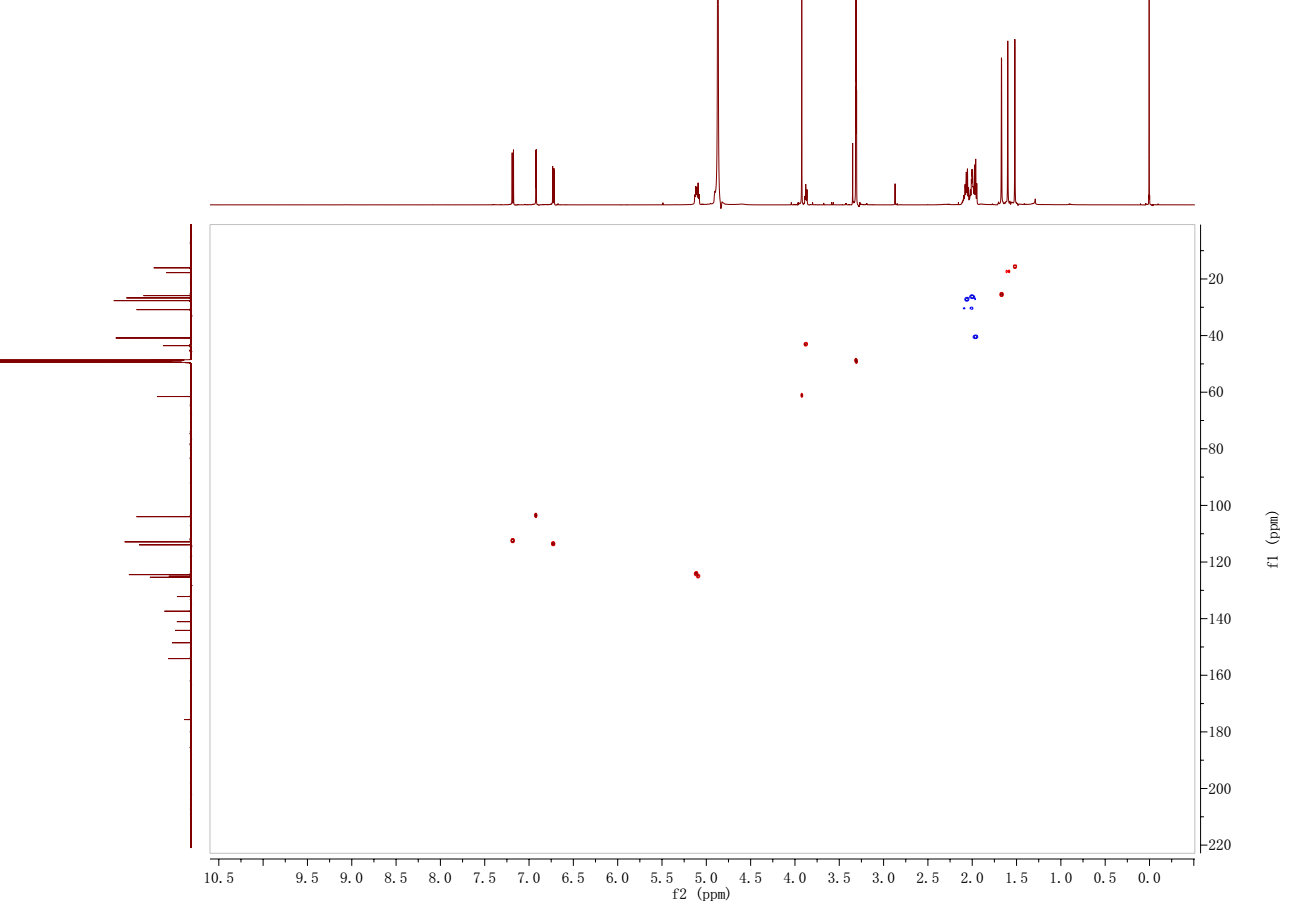


Figure S12. HSQC spectrum of **2** in methanol-*d*_4_


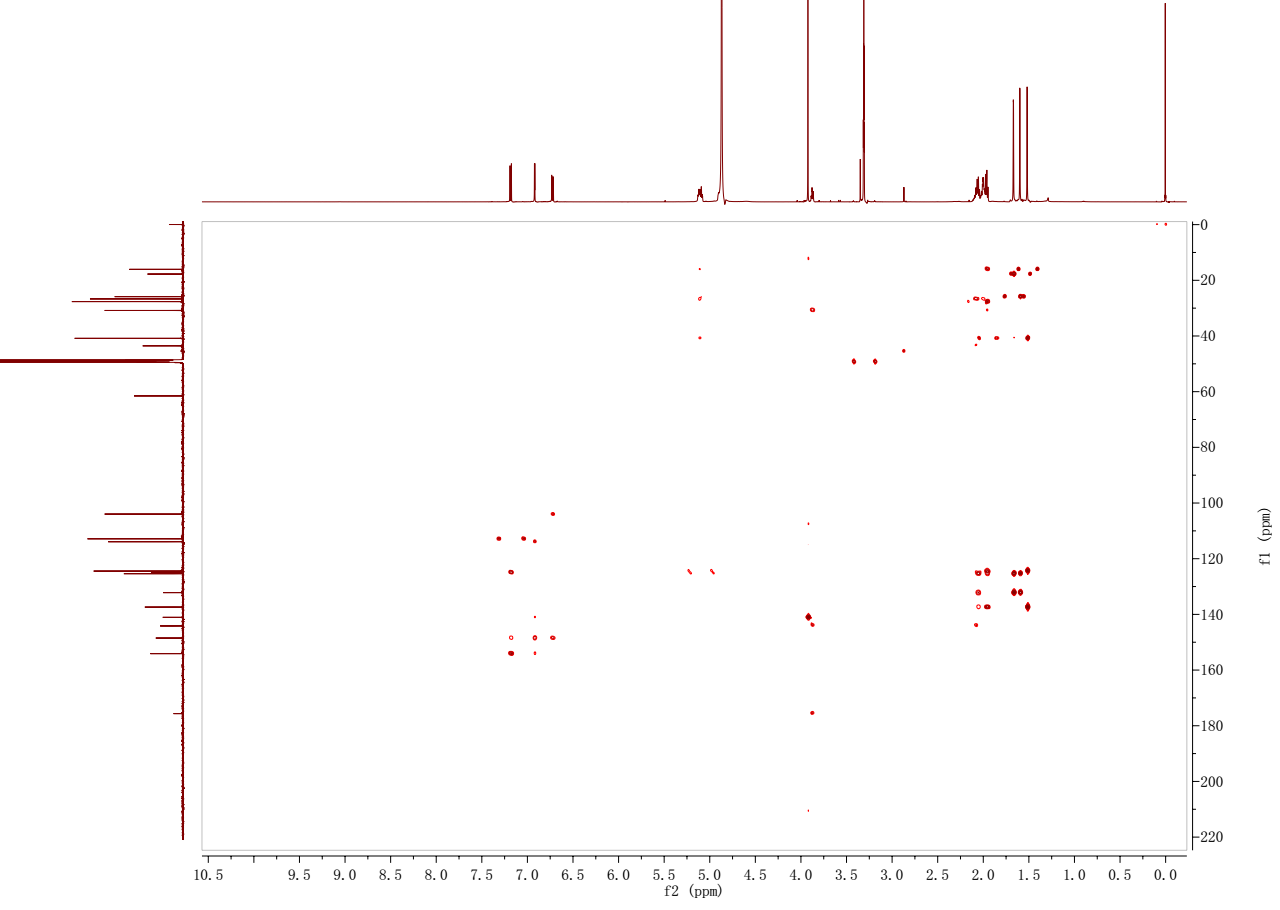


Figure S13. HMBC spectrum of **2** in methanol-*d*_4_


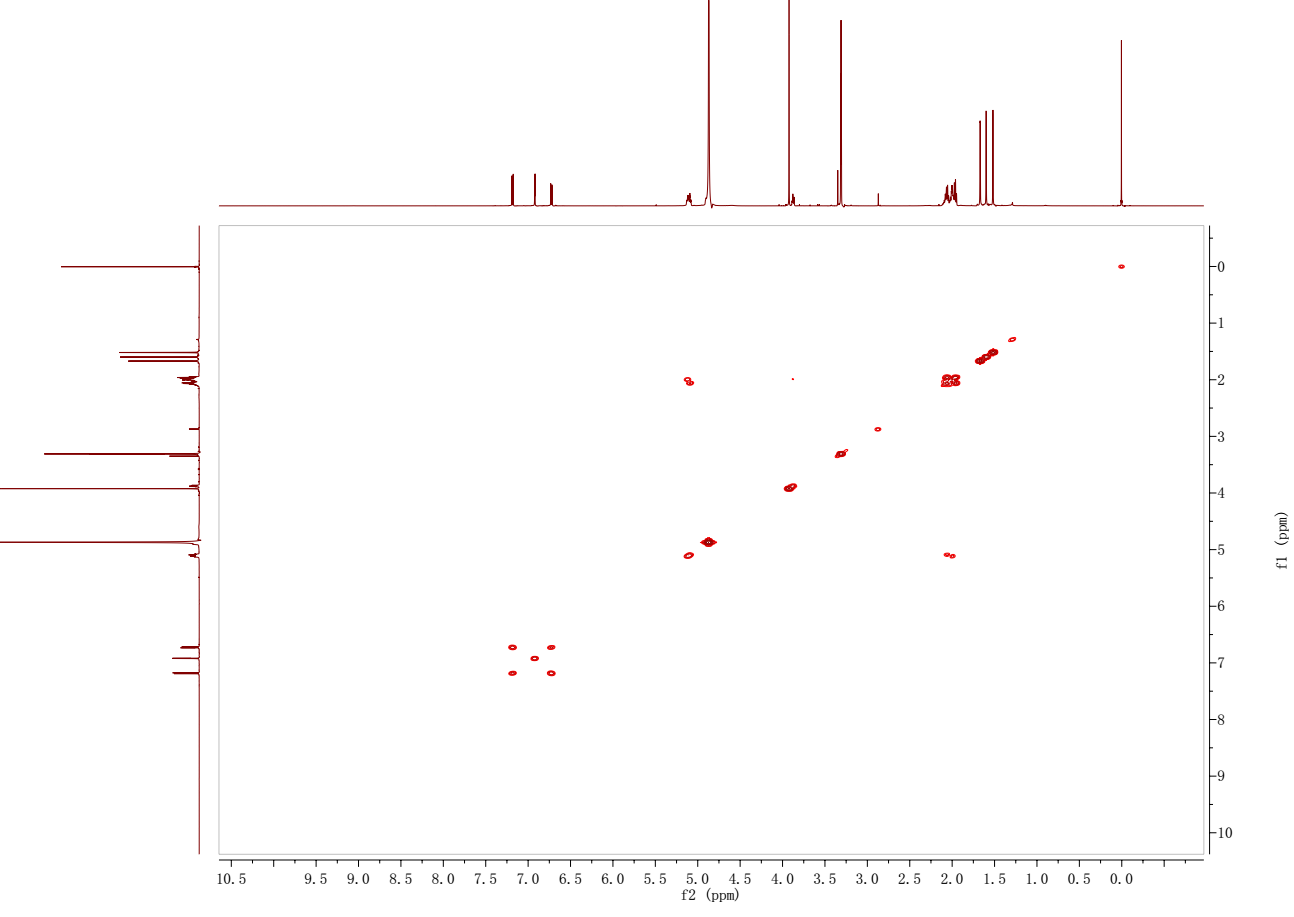


Figure S14. ^1^H-^1^H COSY spectrum of **2** in methanol-*d*_4_


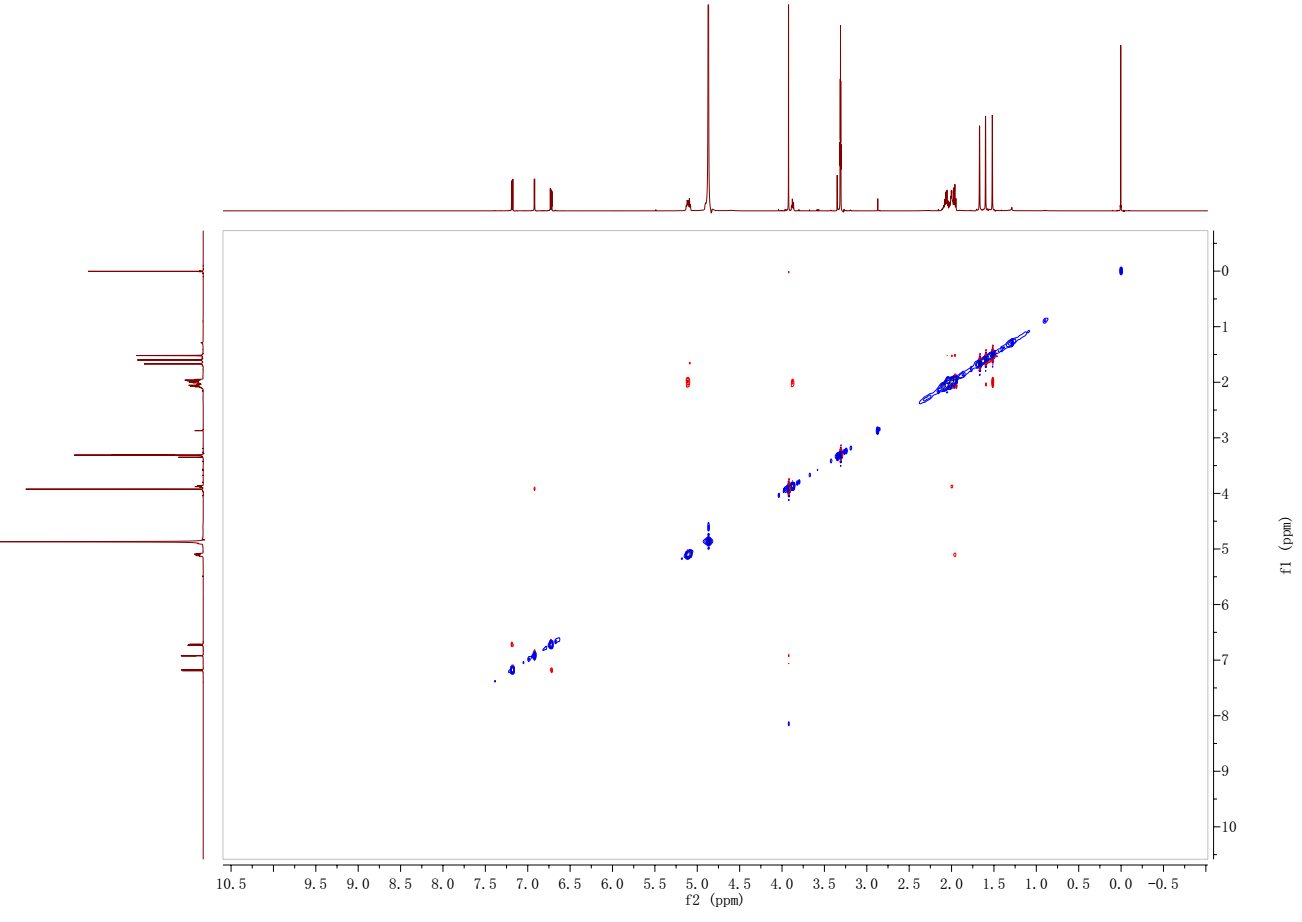


Figure S15. ROESY spectrum of **2** in methanol-*d*_4_


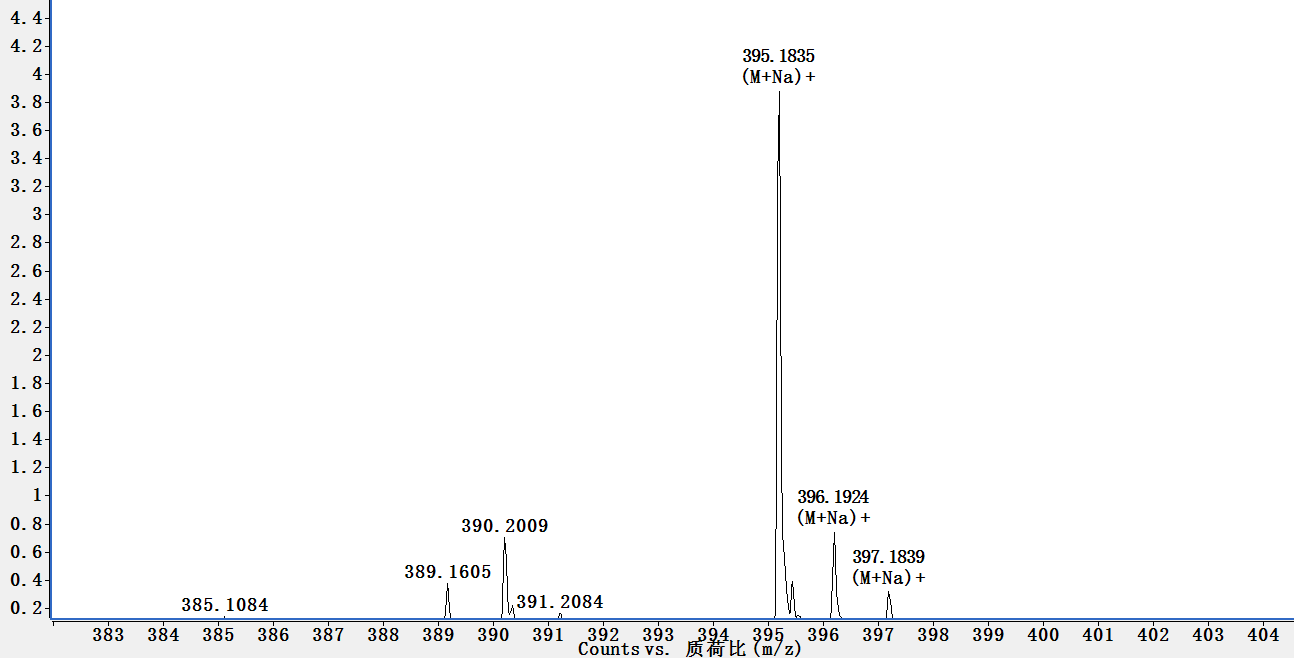


Figure S16. HRESIMS of **2**


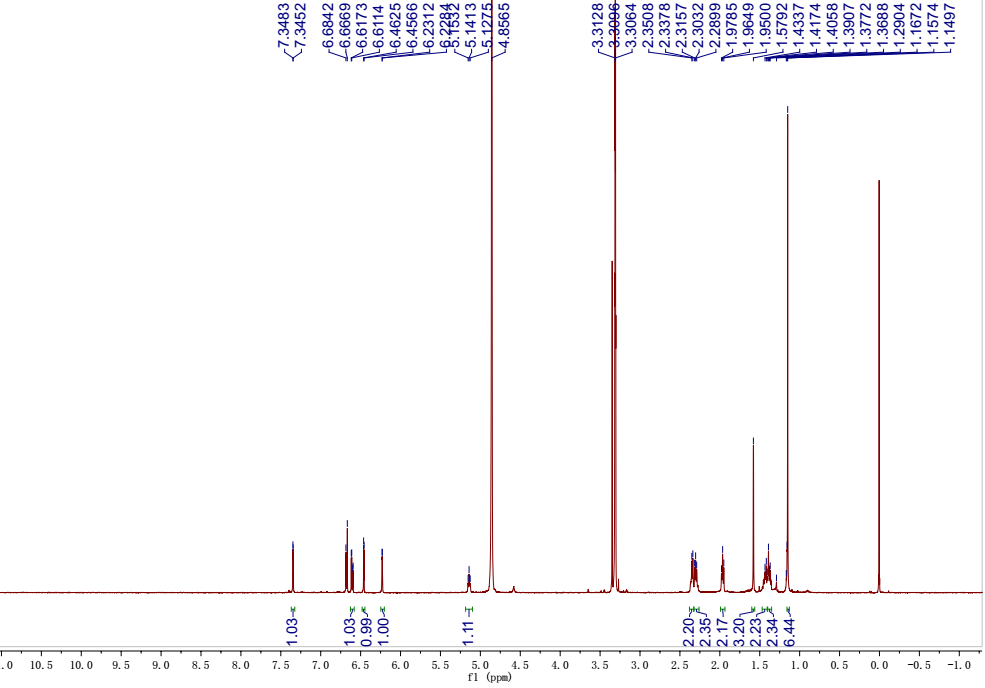


Figure S17. ^1^H NMR spectrum of **3** in methanol-*d*_4_


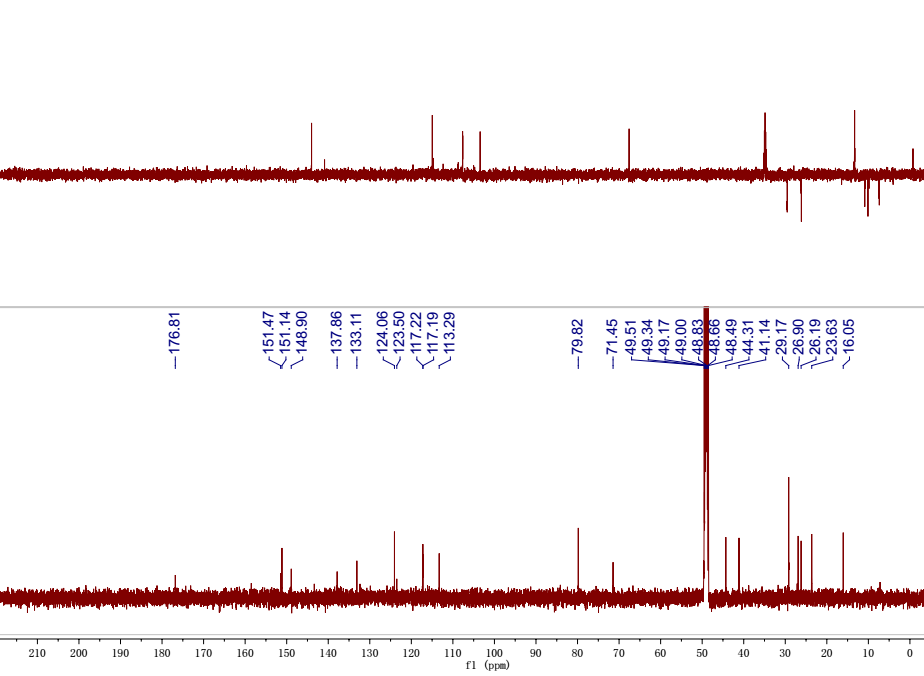


Figure S18. ^13^C NMR and DEPT spectra of **3** in methanol-*d*_4_


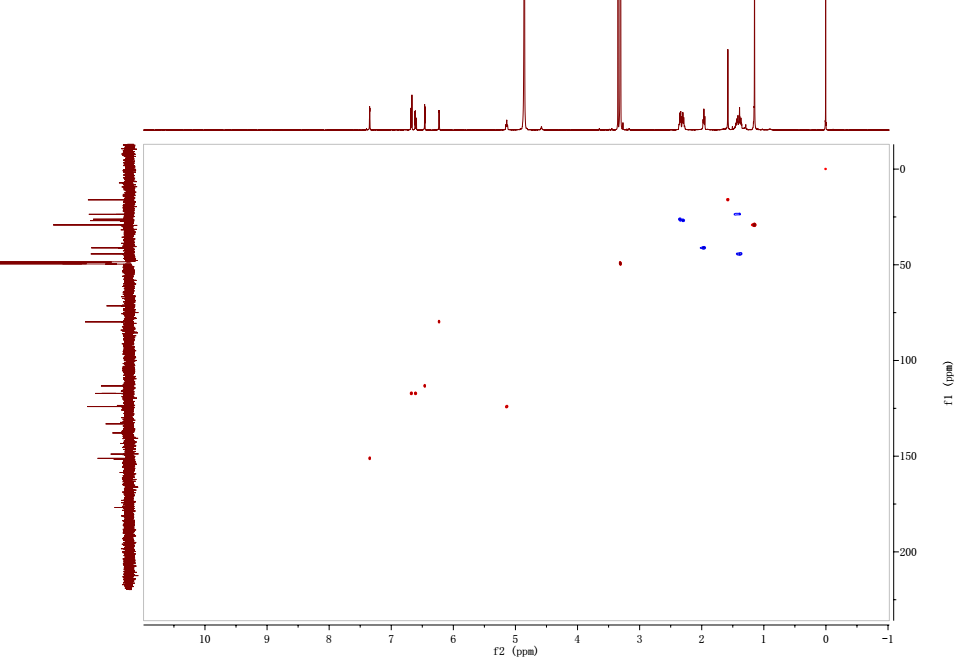


Figure S19. HSQC spectrum of **2** in methanol-*d*_4_


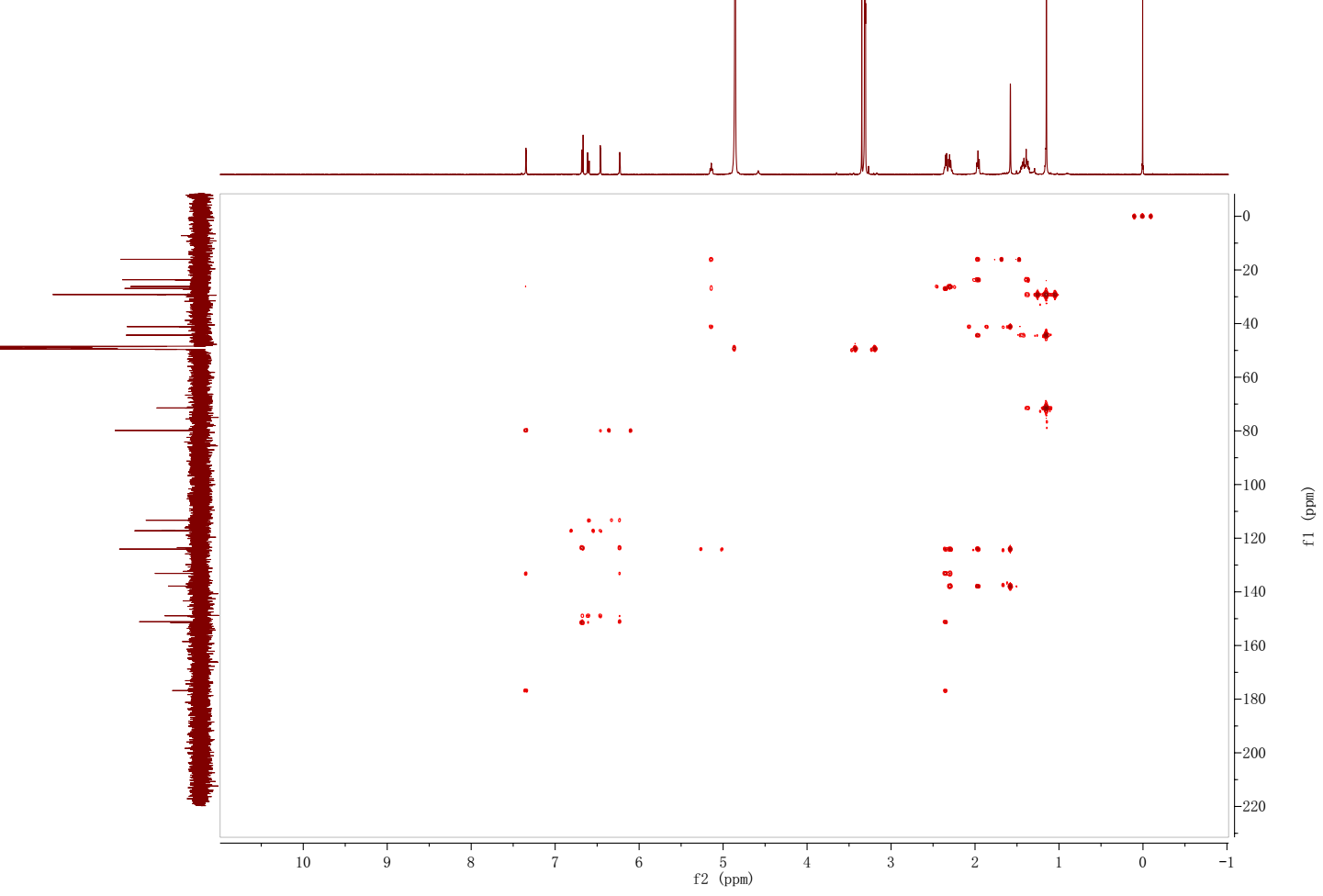


Figure S20. HMBC spectrum of **3** in methanol-*d*_4_


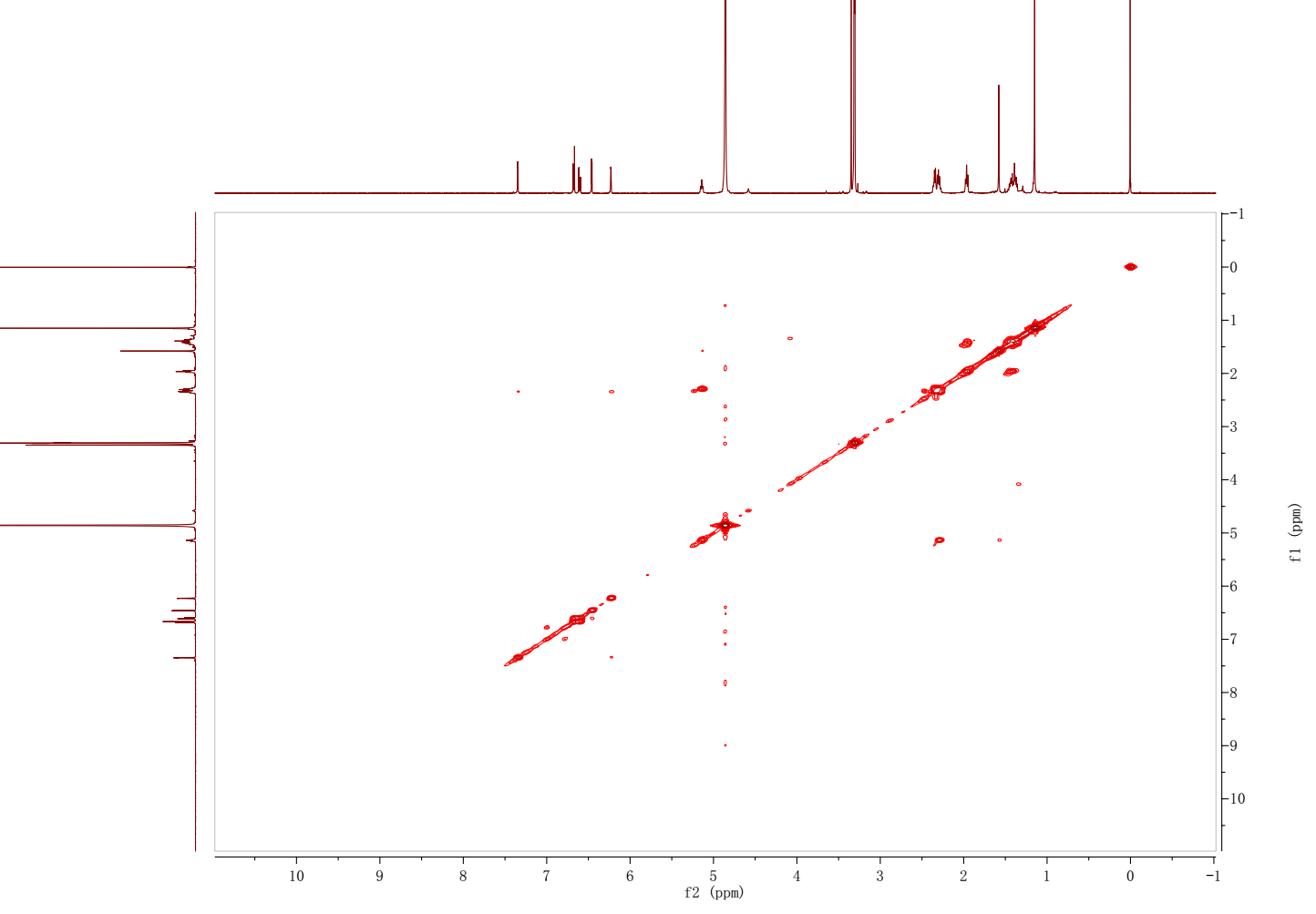


Figure S21. ^1^H-^1^H COSY spectrum of **3** in methanol-*d*_4_


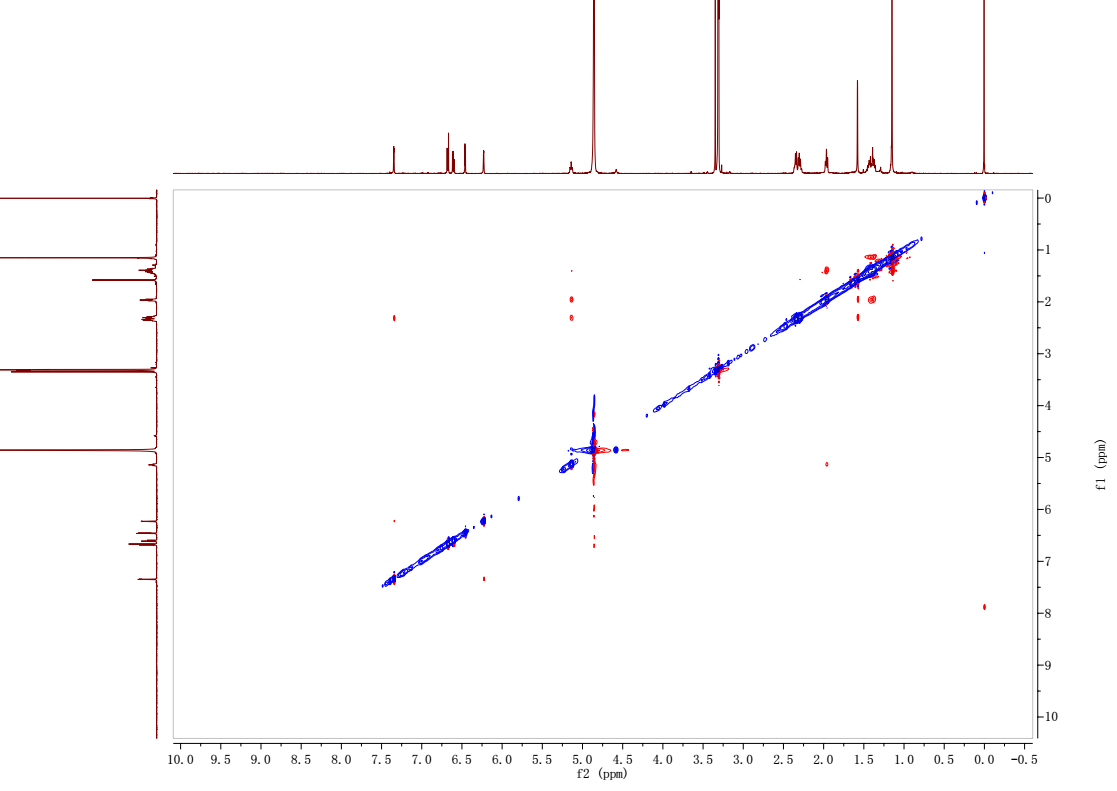


Figure S22. ROESY spectrum of **3** in methanol-*d*_4_


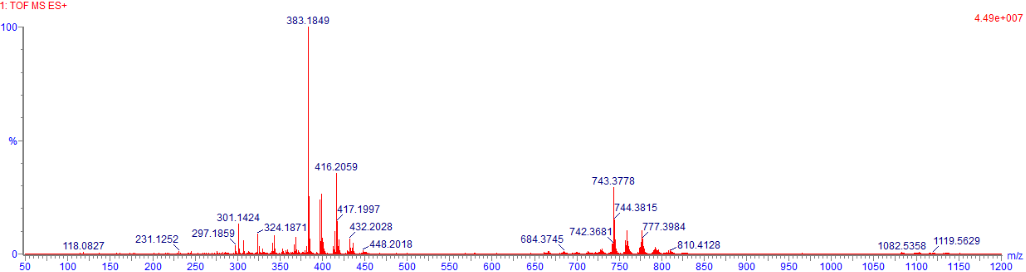


Figure S23. HRESIMS of **3**

**
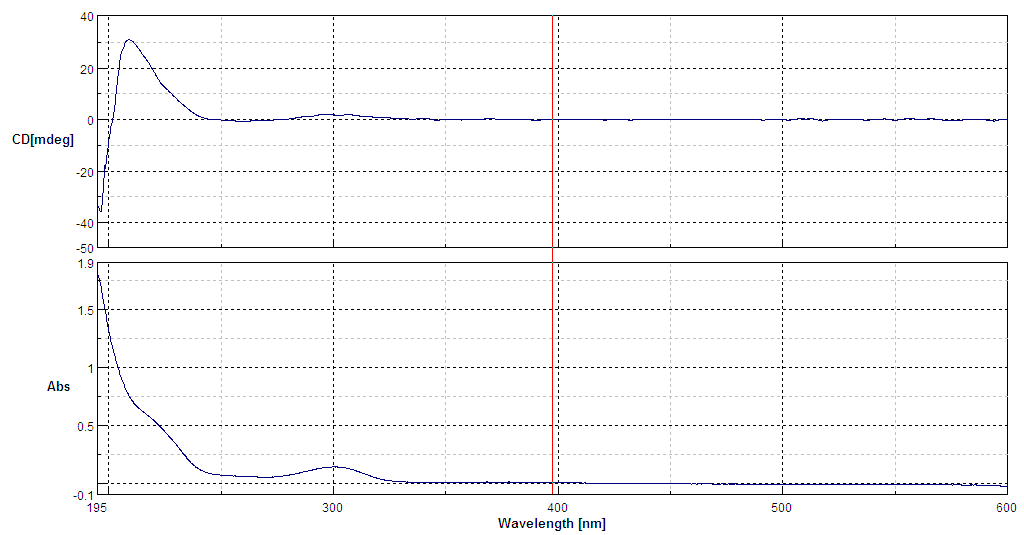
**

Figure S24. CD and UV spectra of (+)-**3**

**
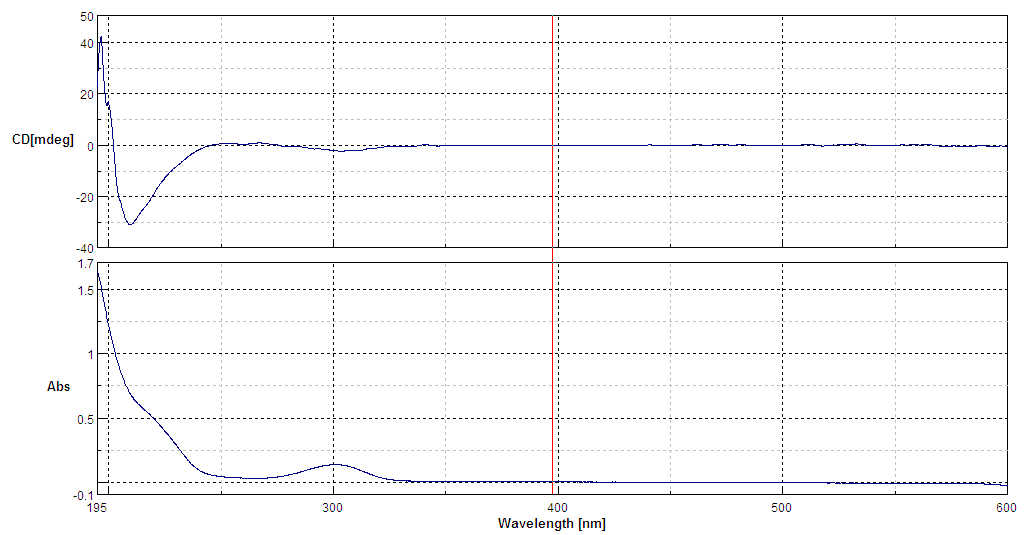
**

Figure S25. CD and UV spectra of (–)-**3**


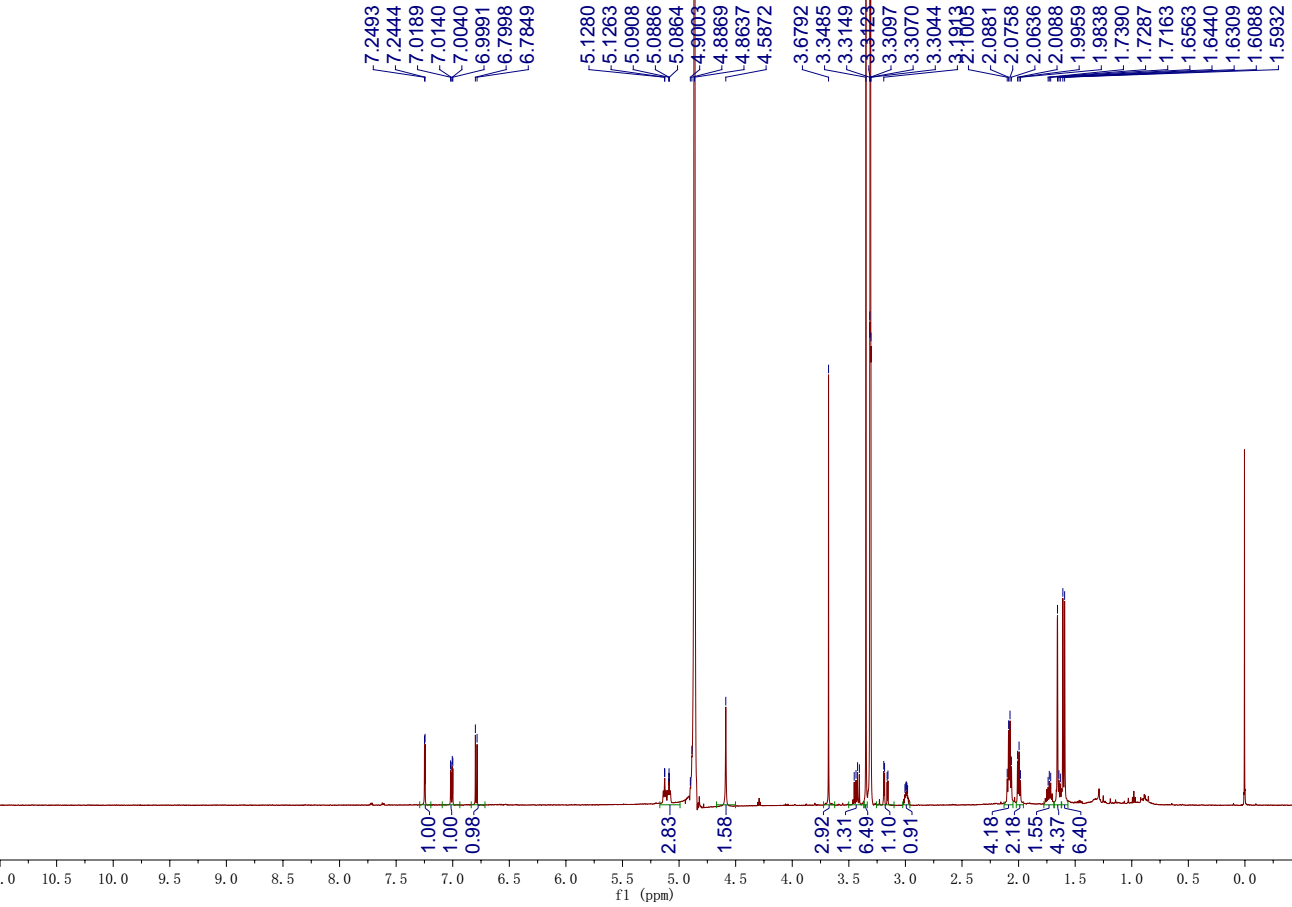


Figure S26. ^1^H NMR spectrum of **4** in methanol-*d*_4_


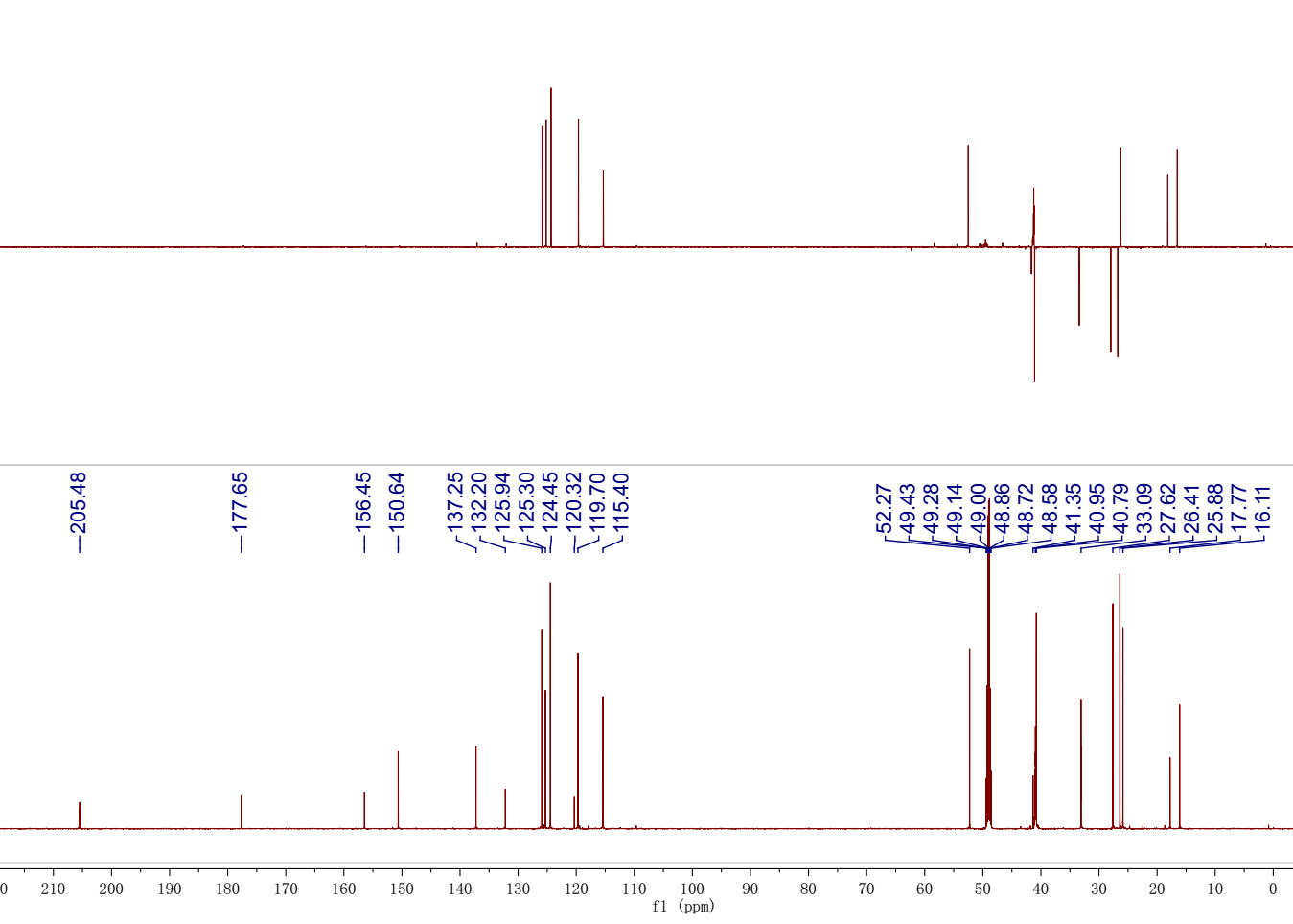


Figure S27. ^13^C NMR and DEPT spectra of **4** in methanol-*d*_4_


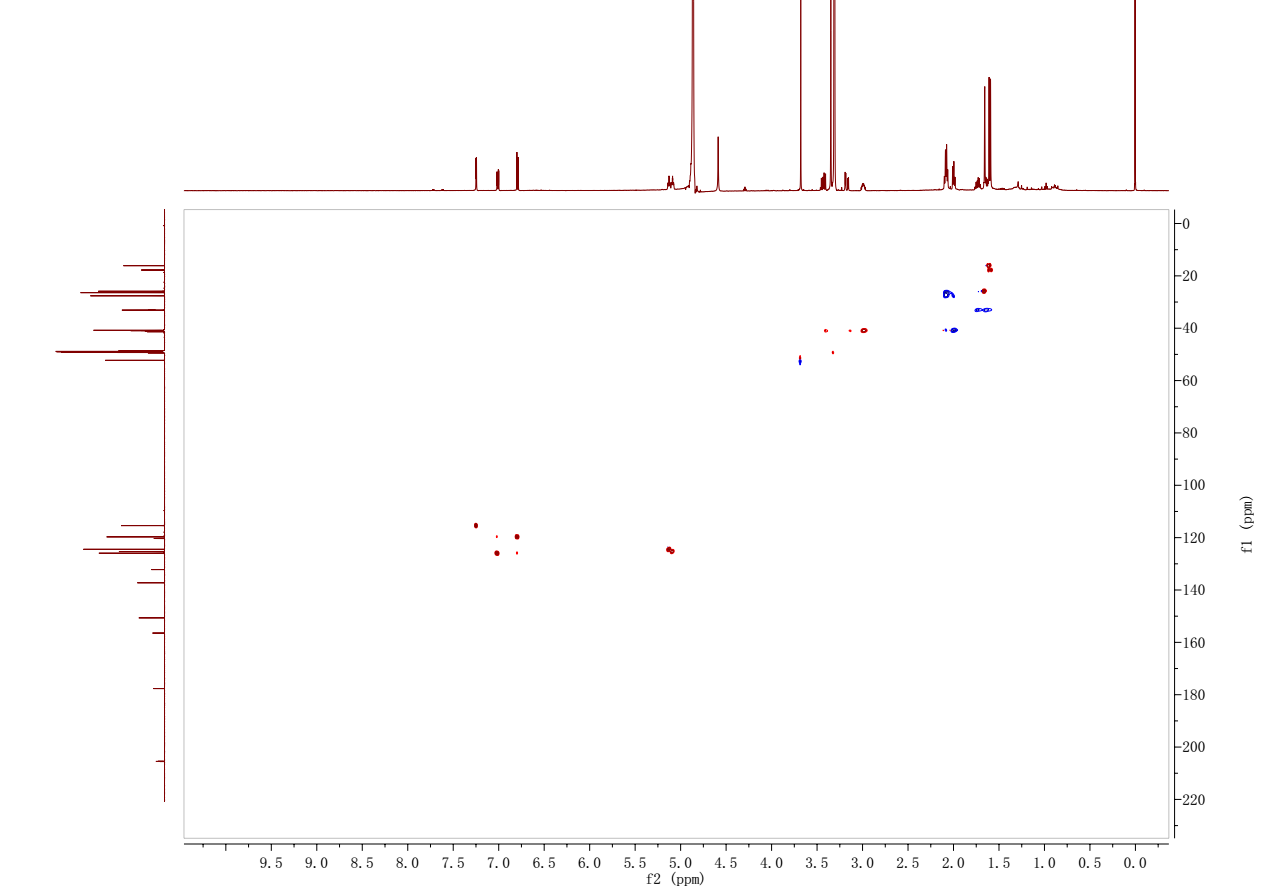


Figure S28. HSQC spectrum of **4** in methanol-*d*_4_


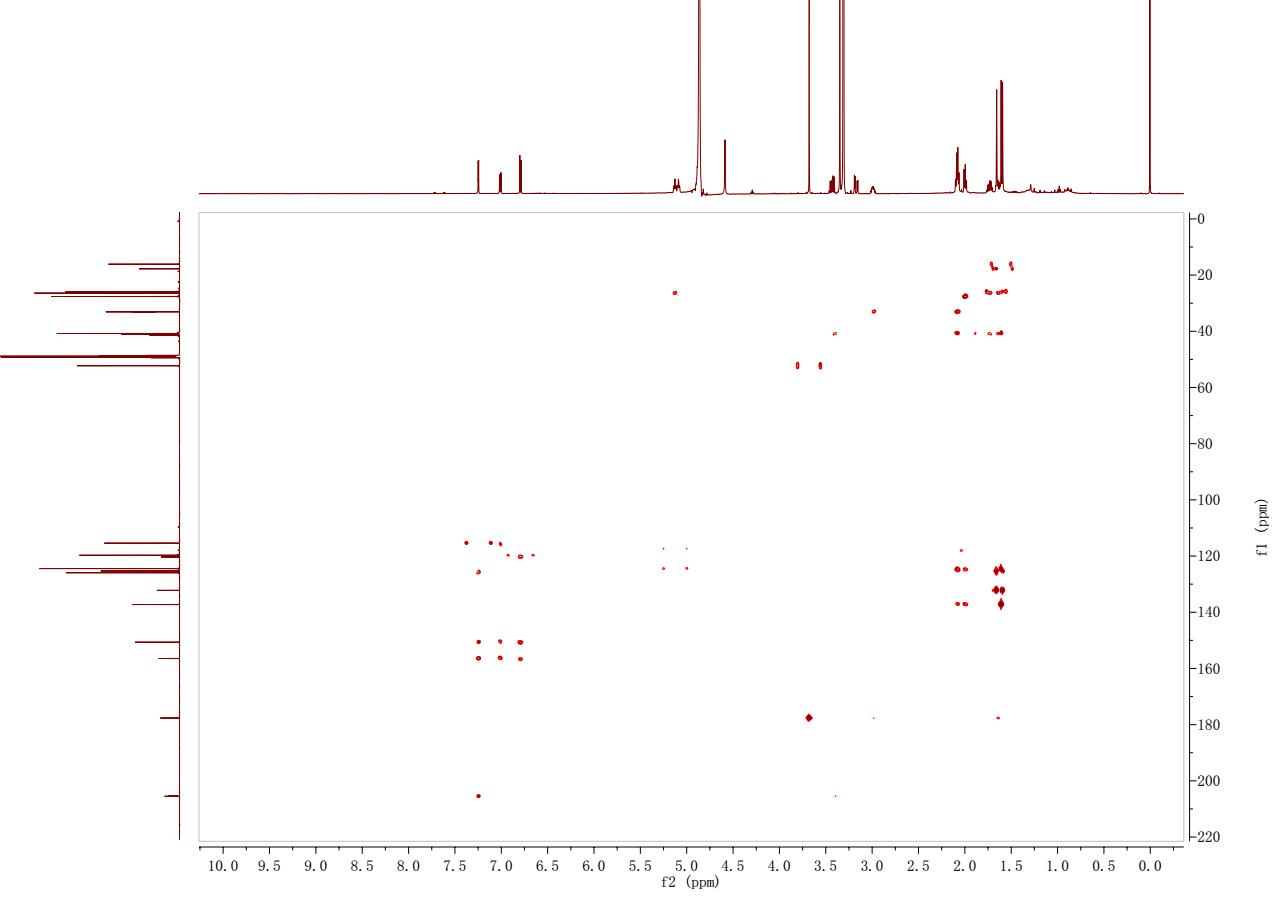


Figure S29. HMBC spectrum of **4** in methanol-*d*_4_


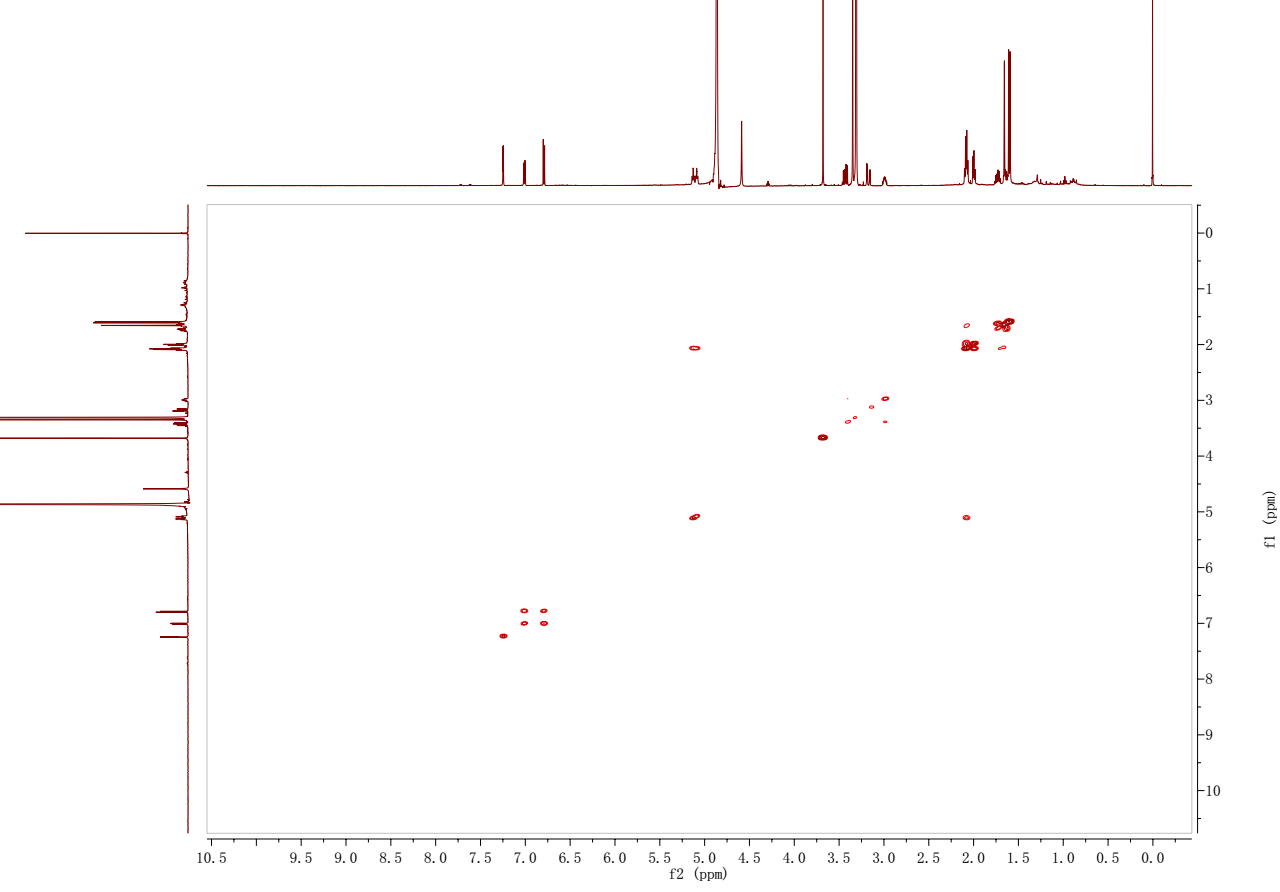


Figure S30. ^1^H-^1^H COSY spectrum of **4** in methanol-*d*_4_


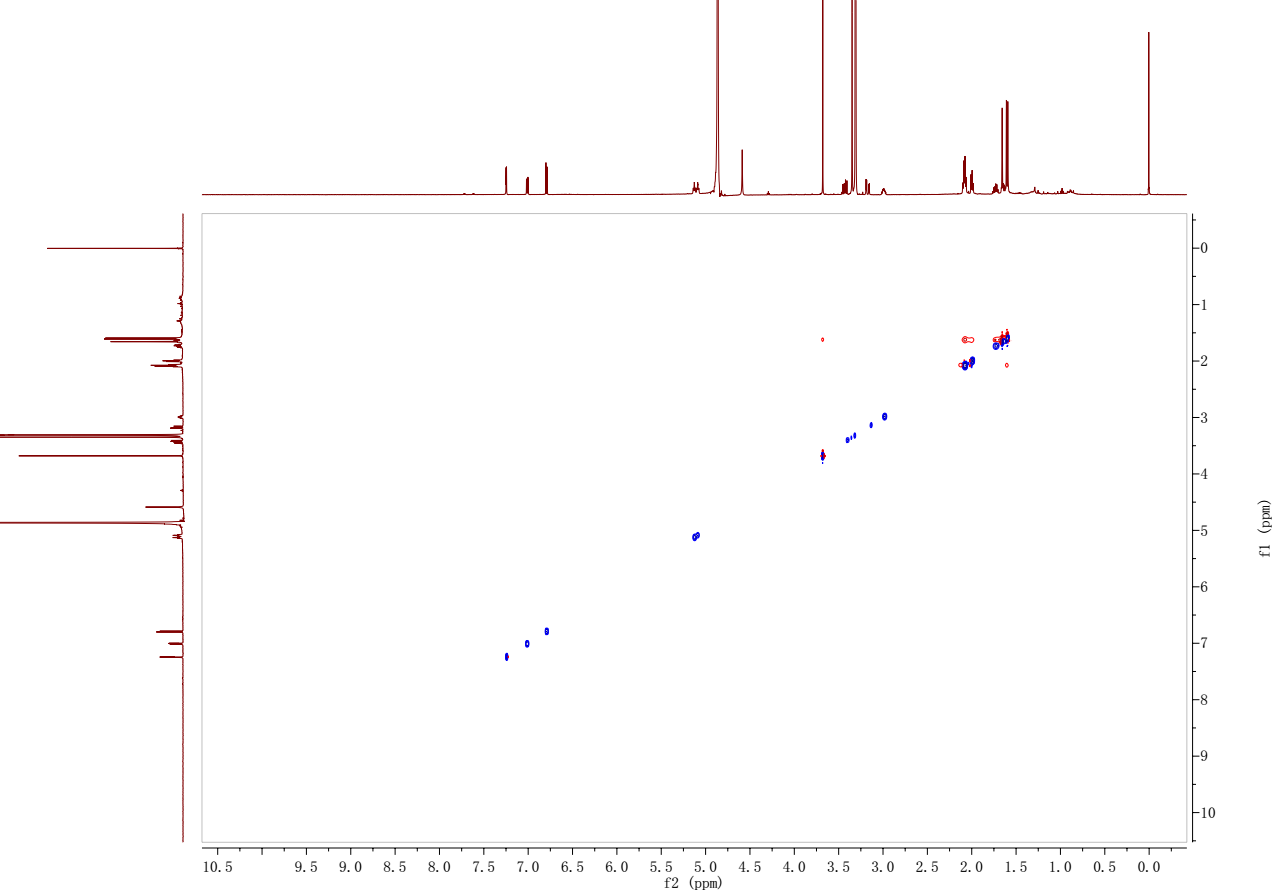


Figure S31. ROESY spectrum of **4** in methanol-*d*_4_


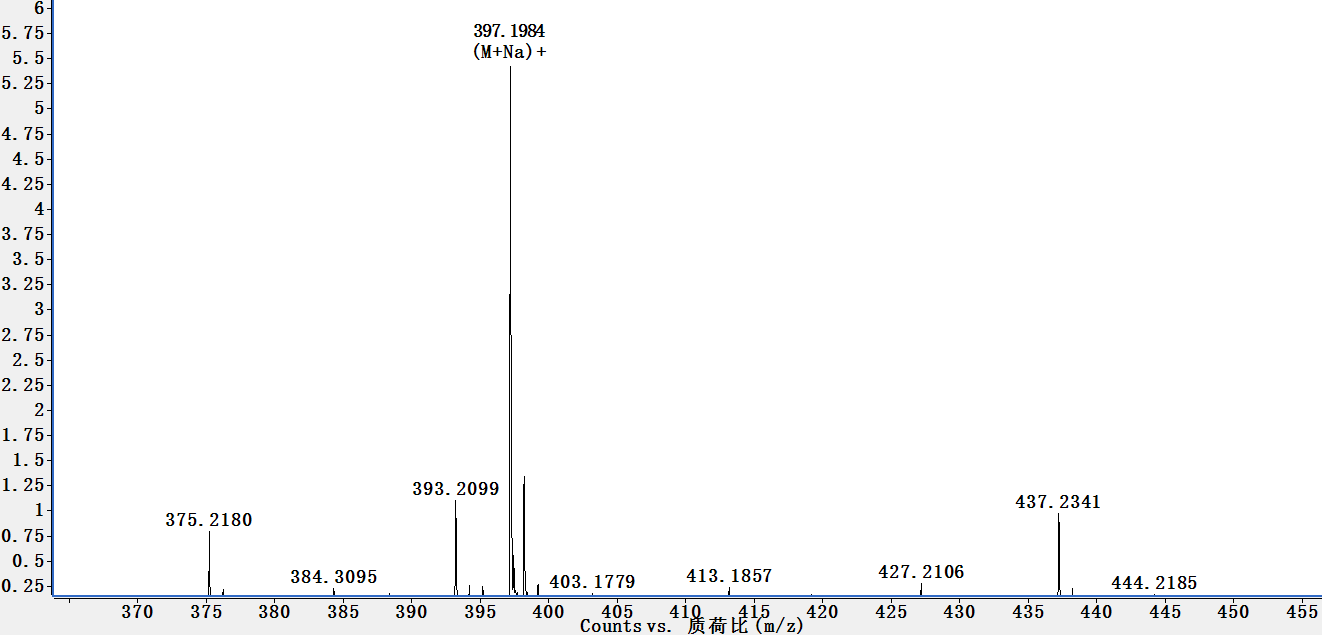


Figure S32. HRESIMS of **4**


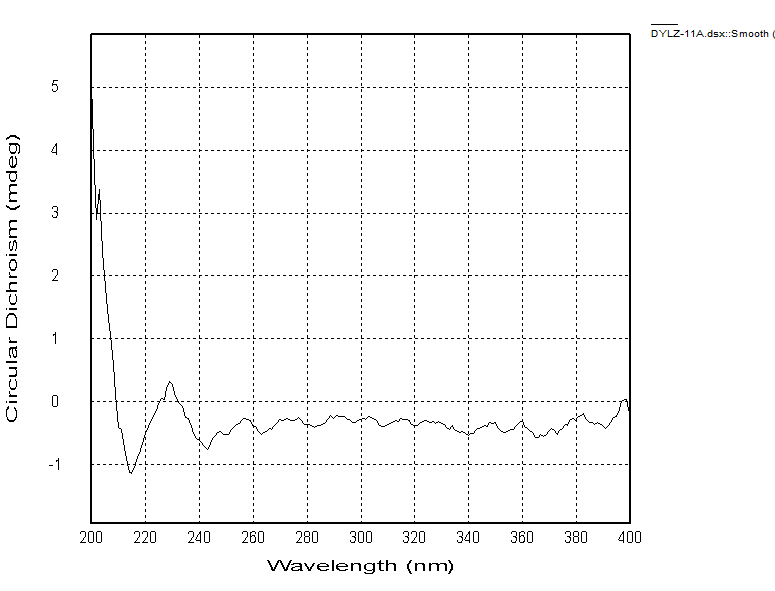


Figure S33. CD spectrum of (+)-**4**


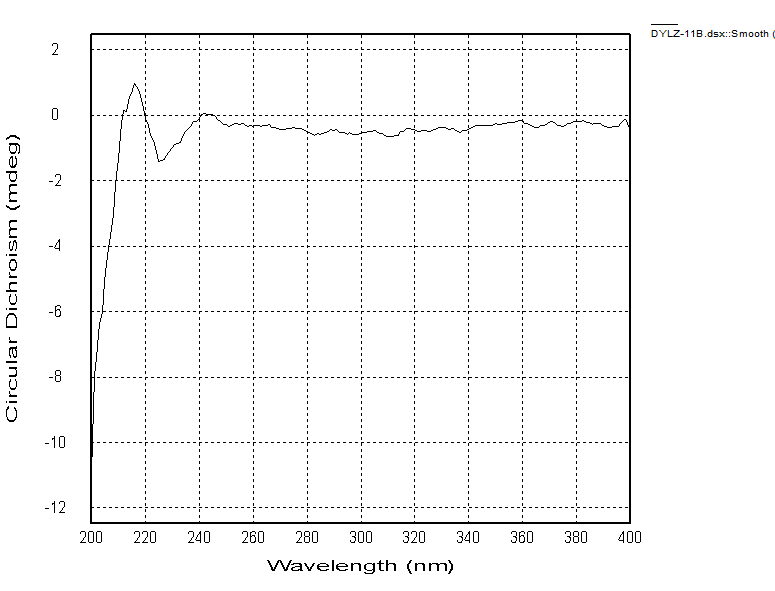


Figure S34. CD spectrum of (–)-**4**


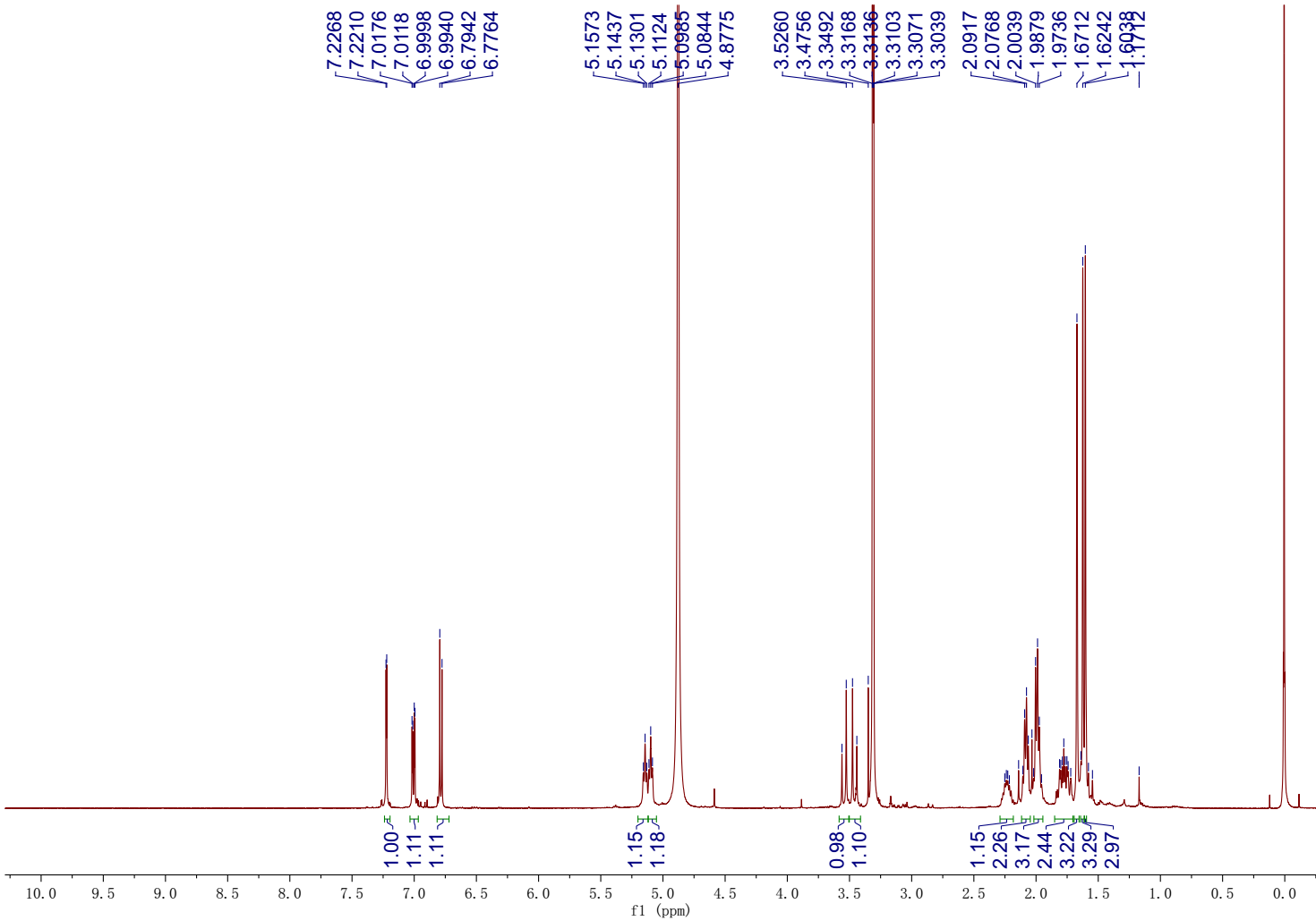


Figure S35. ^1^H NMR spectrum of **5** in methanol-*d*_4_


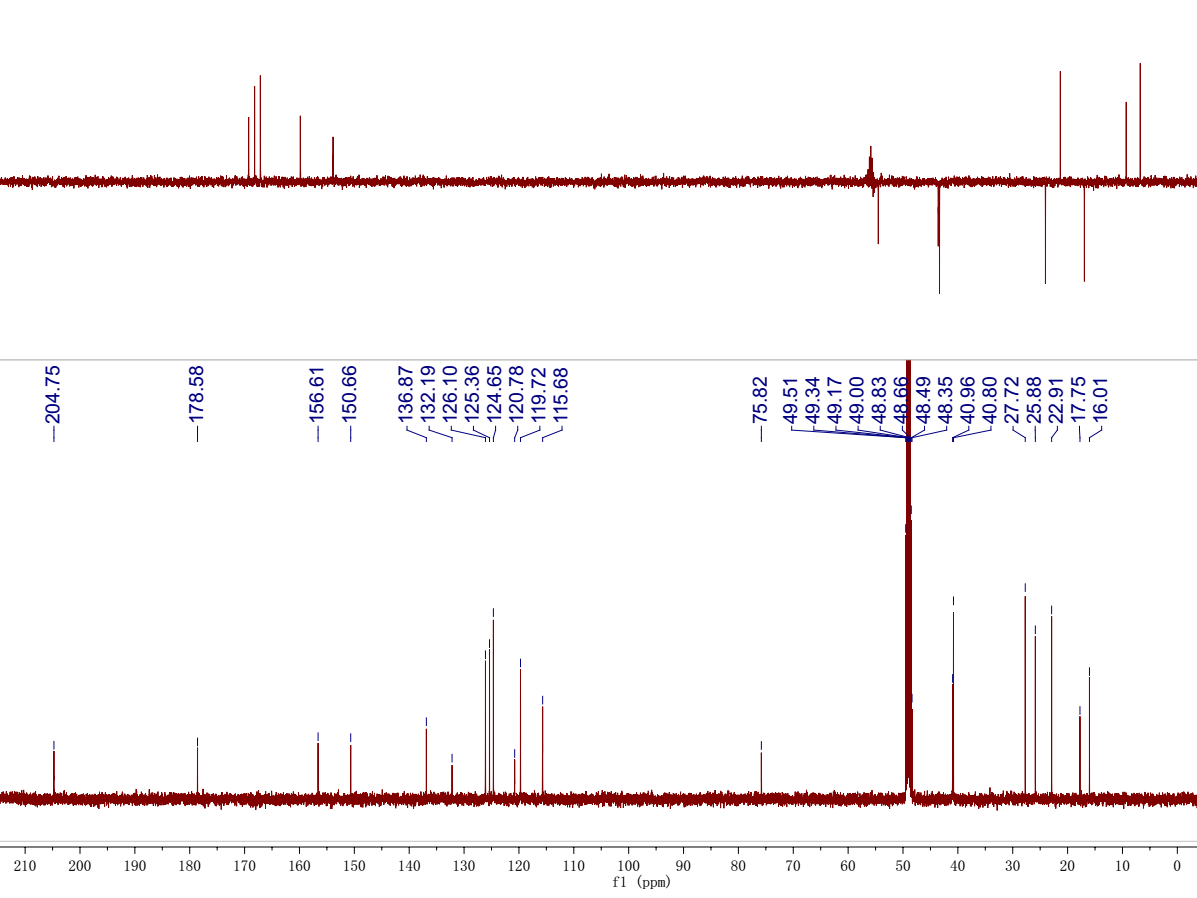


Figure S36. ^13^C NMR and DEPT spectra of **5** in methanol-*d*_4_


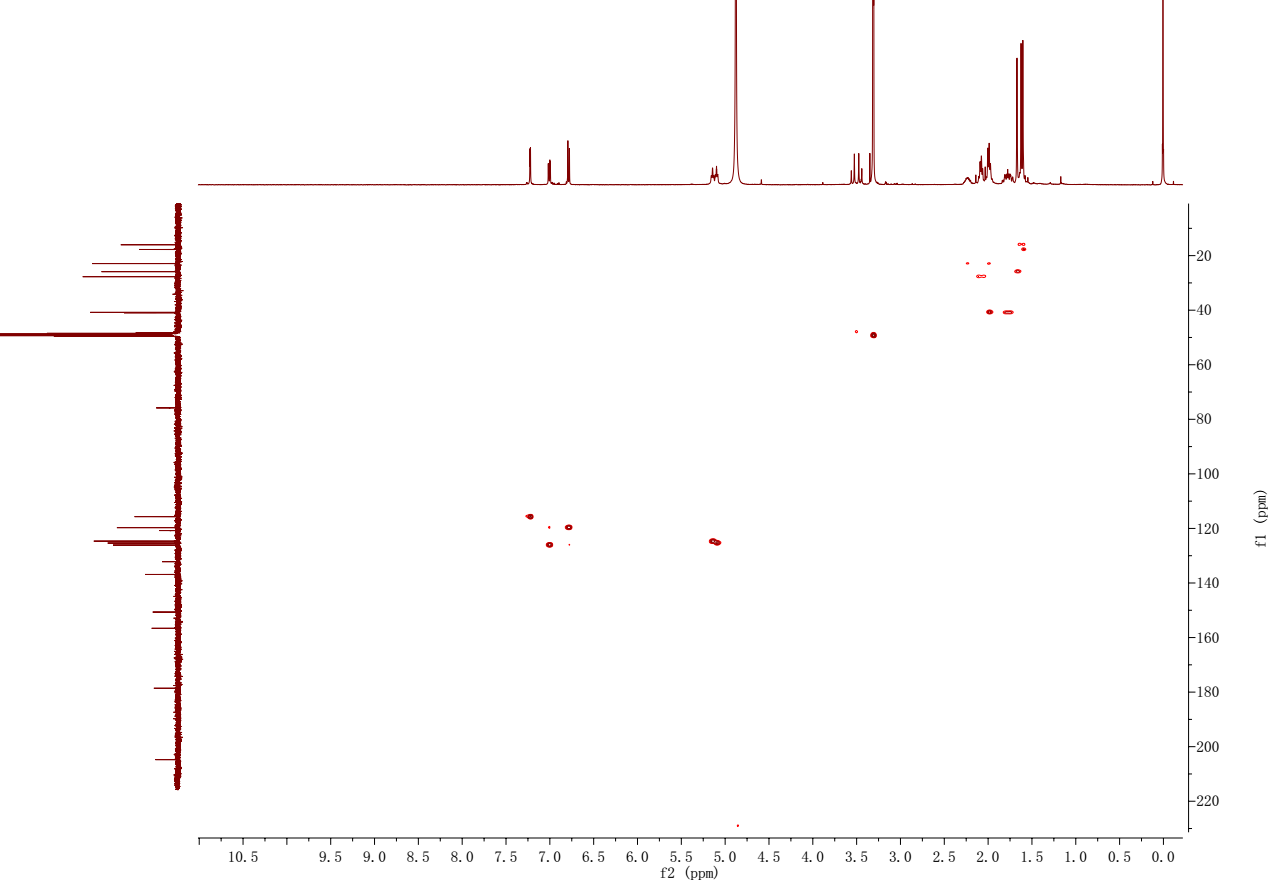


Figure S37. HSQC spectrum of **5** in methanol-*d*_4_


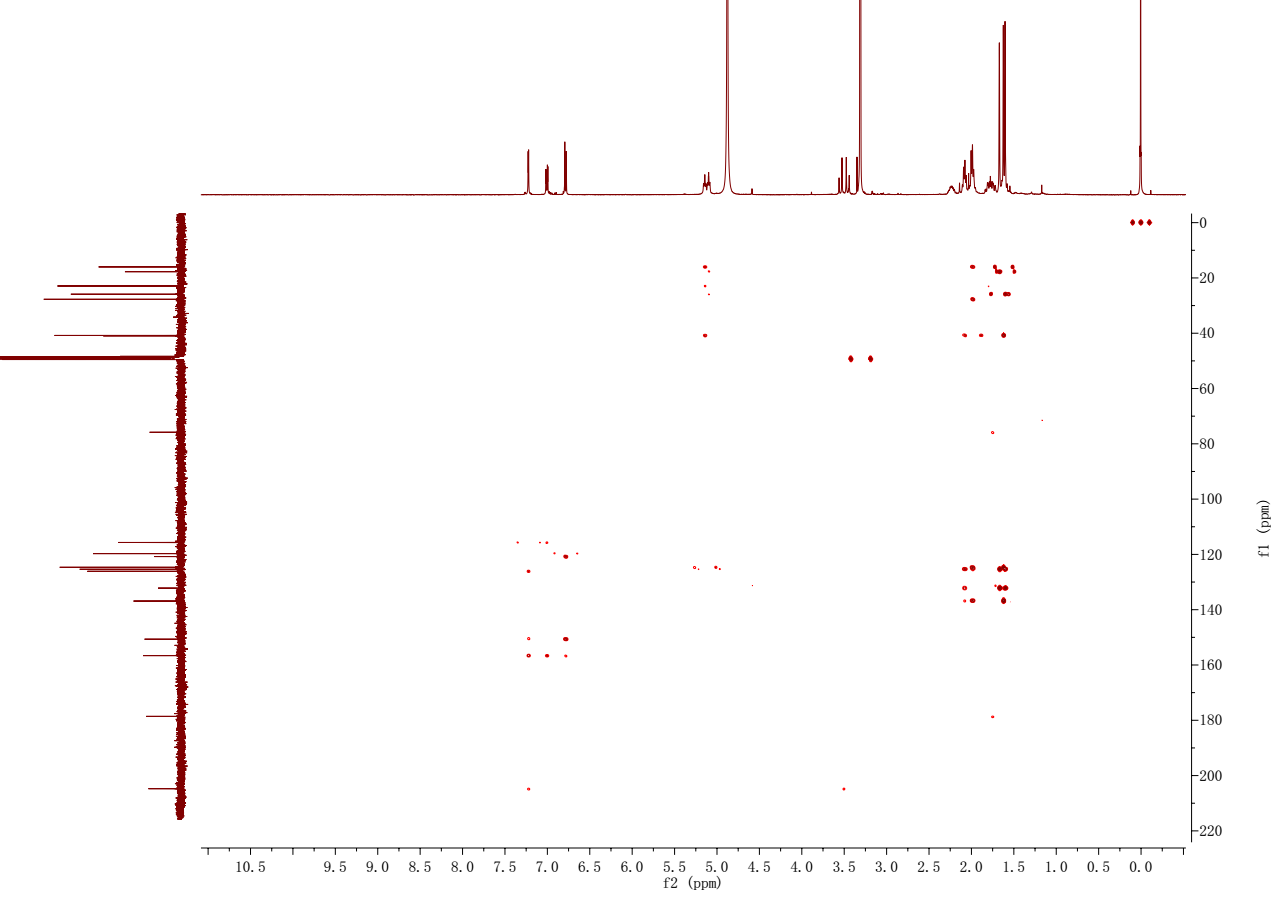


Figure S38. HMBC spectrum of **5** in methanol-*d*_4_


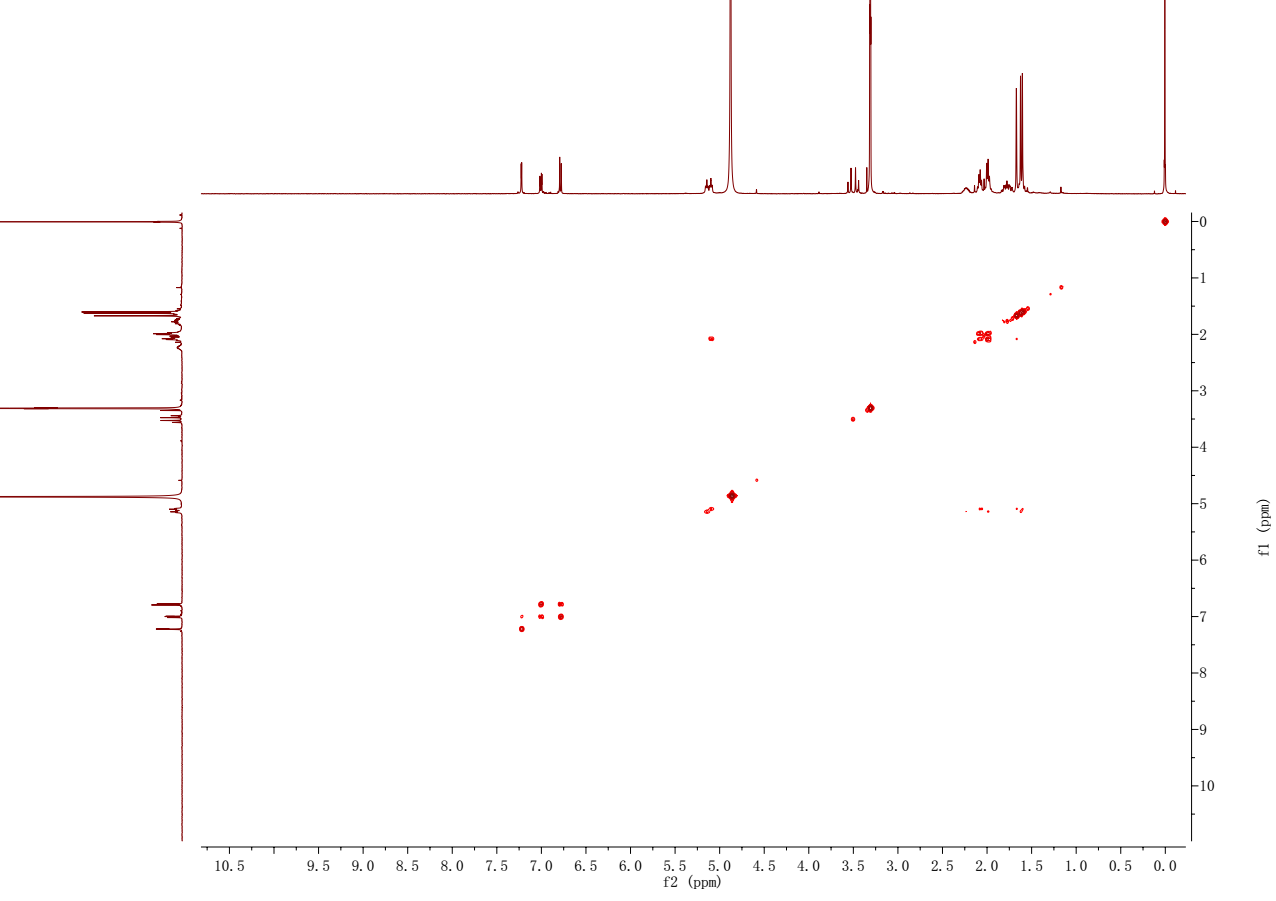


Figure S39. ^1^H-^1^H COSY spectrum of **5** in methanol-*d*_4_


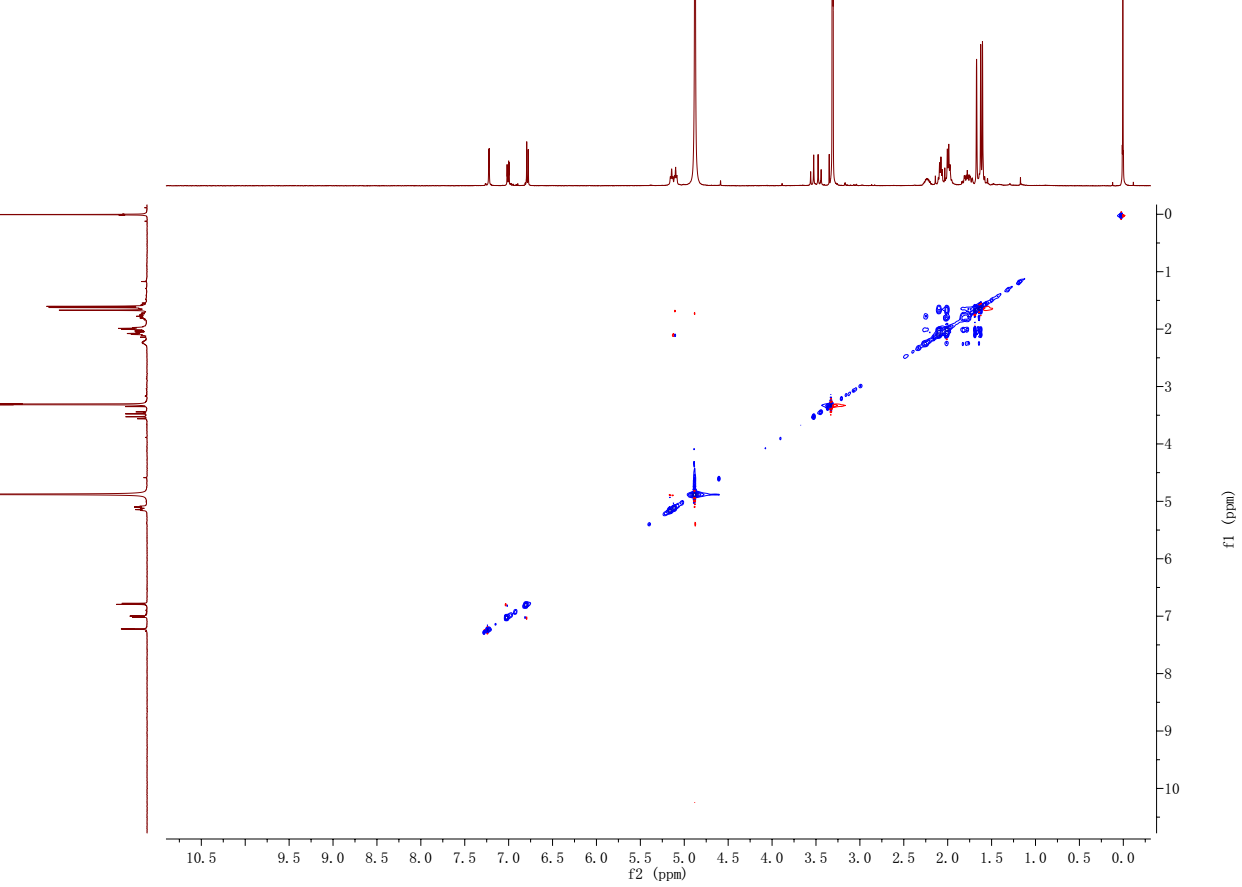


Figure S40. ROESY spectrum of **5** in methanol-*d*_4_


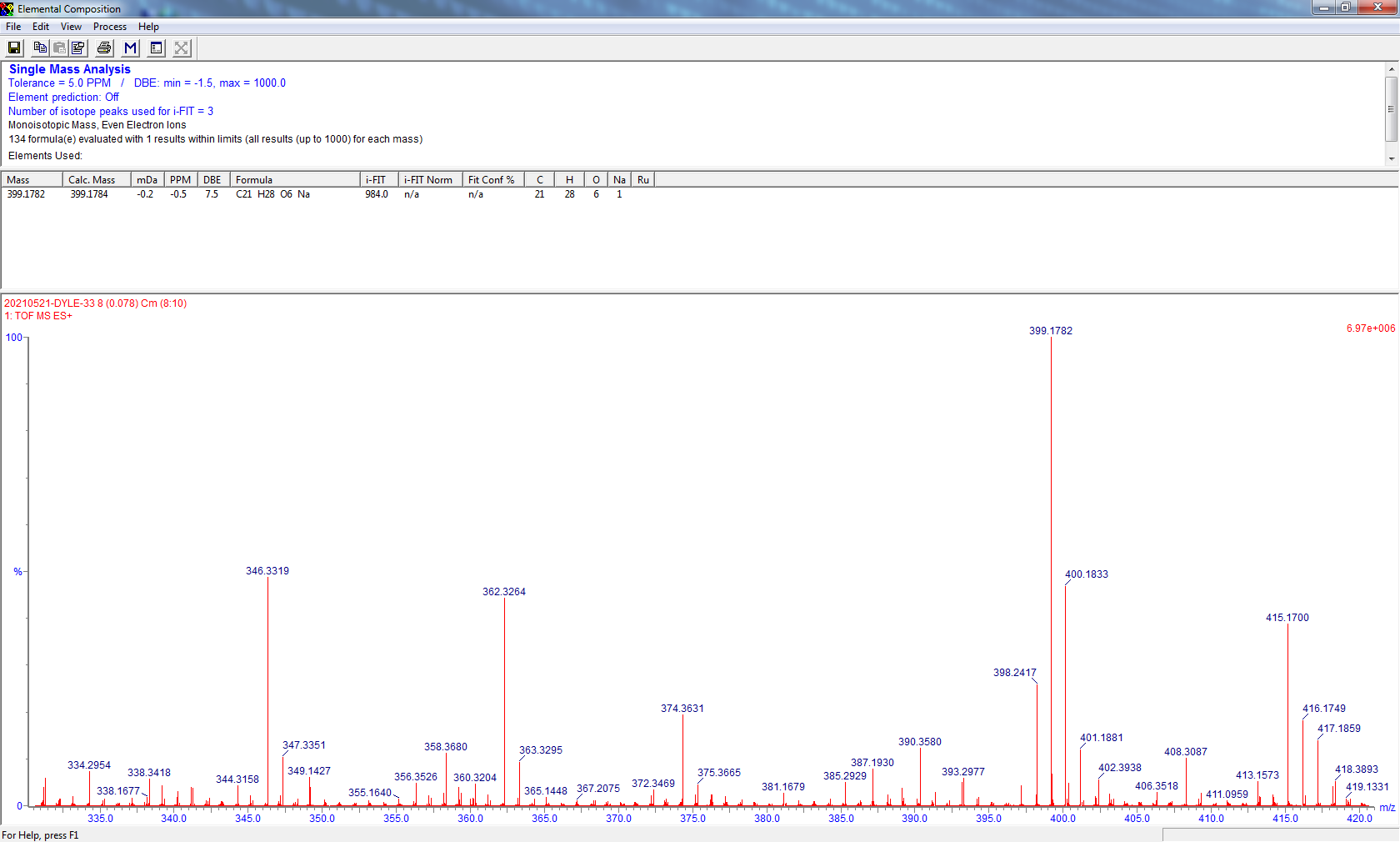


Figure S41. HRESIMS of **5**

**Figure S42.** ^13^C NMR calculated structures of **1**

**NMR calculation** **Methods**

NMR calculation was calculated by using the same software of conformation search and geometry optimization as in the literature ^[1]^, The optimized structures was submited to gauge-independent atomic orbital (GIAO) calculations of chemical shifts at the B3LYP/6-311G(d,p) level in methanol with the PCM solvent model in Gaussian 09 software. Regression analysis of calculated possible isomers versus experimental ^13^C NMR chemical shifts of **1**. Linear correlation coefficients (*R*^2^) was calculated for the evaluation of the results. After Boltzmann weighing of the predicted chemical shift of each isomers, the DP4+ parameters were calculated using the excel file provided by Ariel M. Sarotti.^[2]^

References:

[1] F.Y. Qin, H.X. Zhang, Q.Q. Di, Y. Wang, Y.M. Yan, W.L. Chen, Y.X. Cheng, Org. Lett. **2020**, 22, 2574–2578 .

[2] M.W. Lodewyk, M.R. Siebert, D.J. Tantillo, Chem. Rev*.* **2012**, *112*, 1839–1862.

**Figure S43.** Optimized geometries of dominant conformers of **1-1** at the B3LYP/6-31(d,p) level in the gas phase

**Table S1.** Conformational analysis of the optimized **1-1** at the B3LYP/6-31(d,p) level in the gas phase

| *Confermors* | G (Hartree) | *Δ*G (Kcal/mol) | Population |
| --- | --- | --- | --- |
| **1-1a** | -1266.90854 | 0.30622 | 12.3% |
| **1-1b** | -1266.90902 | 0 | 20.7% |
| **1-1c** | -1266.90902 | 0.00377 | 20.5% |
| **1-1d** | -1266.90847 | 0.34826 | 11.5% |
| **1-1e** | -1266.90633 | 1.68987 | 1.2% |
| **1-1f** | -1266.90865 | 0.23657 | 13.8% |
| **1-1g** | -1266.90784 | 0.74547 | 5.9% |
| **1-1h** | -1266.90835 | 0.42043 | 10.2% |
| **1-1i** | -1266.90671 | 1.45141 | 1.8% |
| **1-1j** | -1266.90508 | 2.47362 | 0.3% |
| **1-1k** | -1266.90556 | 2.17618 | 0.5% |
| **1-1l** | -1266.90641 | 1.64092 | 1.3% |

**Figure S44.** Optimized geometries of dominant conformers of **1-2** at the B3LYP/6-31(d,p) level in the gas phase

**Table S2.** Conformational analysis of the optimized **1-2** at the B3LYP/6-31(d,p) level in the gas phase

| *Confermors* | G (Hartree) | *Δ*G (Kcal/mol) | Population |
| --- | --- | --- | --- |
| **1-2a** | -1266.90799 | 0.79254 | 5.1% |
| **1-2b** | -1266.90894 | 0.19955 | 13.8% |
| **1-2c** | -1266.90885 | 0.25665 | 12.6% |
| **1-2d** | -1266.90841 | 0.53024 | 7.9% |
| **1-2e** | -1266.90647 | 1.74885 | 1.0% |
| **1-2f** | -1266.90648 | 1.74258 | 1.0% |
| **1-2g** | -1266.90793 | 0.83458 | 4.7% |
| **1-2h** | -1266.90884 | 0.26041 | 12.5% |
| **1-2i** | -1266.90851 | 0.46937 | 8.8% |
| **1-2j** | -1266.90885 | 0.25728 | 12.6% |
| **1-2k** | -1266.90926 | 0 | 19.4% |
| **1-2l** | -1266.90603 | 2.02432 | 0.6% |

**Figure S45.** Optimized geometries of dominant conformers of **1-3** at the B3LYP/6-31(d,p) level in the gas phase

**Table S3.** Conformational analysis of the optimized **1-3** at the B3LYP/6-31(d,p) level in the gas phase

| *Confermors* | G (Hartree) | *Δ*G (Kcal/mol) | Population |
| --- | --- | --- | --- |
| **1-3a** | -1343.31752 | 3.68344 | 0.1% |
| **1-3b** | -1343.32094 | 1.53801 | 4.9% |
| **1-3c** | -1343.31839 | 3.13814 | 0.3% |
| **1-3d** | -1343.32125 | 1.33972 | 6.9% |
| **1-3e** | -1343.31806 | 3.34145 | 0.2% |
| **1-3f** | -1343.32234 | 0.65637 | 21.8% |
| **1-3g** | -1343.32339 | 0 | 65.8% |

**Figure S46.** Optimized geometries of dominant conformers of **1-4** at the B3LYP/6-31(d,p) level in the gas phase

**Table S4.** Conformational analysis of the optimized **1-4** at the B3LYP/6-31(d,p) level in the gas phase

| *Confermors* | G (Hartree) | *Δ*G (Kcal/mol) | Population |
| --- | --- | --- | --- |
| **1-4a** | -1343.32217 | 0.16064 | 41.6% |
| **1-4b** | -1343.31844 | 2.49872 | 0.8% |
| **1-4c** | -1343.32242 | 0 | 54.5% |
| **1-4d** | -1343.31932 | 1.94589 | 2.1% |
| **1-4e** | -1343.31869 | 2.34498 | 1.0% |

**Figure S47.** Optimized geometries of dominant conformers of **1-5** at the B3LYP/6-31(d,p) level in the gas phase

**Table S5.** Conformational analysis of the optimized **1-5** at the B3LYP/6-31(d,p) level in the gas phase

| *Confermors* | G (Hartree) | *Δ*G (Kcal/mol) | Population |
| --- | --- | --- | --- |
| **1-5a** | -1343.32045 | 2.04315 | 1.8% |
| **1-5b** | -1343.32371 | 0 | 56.1% |
| **1-5c** | -1343.32339 | 0.19515 | 40.3% |
| **1-5d** | -1343.32047 | 2.02934 | 1.8% |

**Figure S48.** Optimized geometries of dominant conformers of **1-6** at the B3LYP/6-31(d,p) level in the gas phase

**Table S6.** Conformational analysis of the optimized **1-6** at the B3LYP/6-31(d,p) level in the gas phase

| *Confermors* | G (Hartree) | *Δ*G (Kcal/mol) | Population |
| --- | --- | --- | --- |
| **1-6a** | -1343.3223 | 0.64068 | 15.8% |
| **1-6b** | -1343.3231 | 0.13491 | 37.1% |
| **1-6c** | -1343.31919 | 2.59033 | 0.6% |
| **1-6d** | -1343.32332 | 0 | 46.5% |

**Figure S49.** Regression analysis of experimental versus calculated ^13^C NMR chemical shifts of **1-1**-**1-6** at the B3LYP/6-311g(d,p) level; linear fitting is shown as a line.

**Table S7.** Comparison of ^13^C NMR experimental values and calculated chemical shifts of **1-1**.

| No. | *δ*_C, exptl_ | *δ*_C, calcd_ | Δ*δ*_C_*^a^* | *δ*_C, corr_ | Δ*δ*_C_*^b^* |
| --- | --- | --- | --- | --- | --- |
| 1 | 156.8 | 167.6 | 10.8 | 159.0 | 2.2 |
| 2 | 121.5 | 126.2 | 4.7 | 119.2 | 2.3 |
| 3 | 116.7 | 122.8 | 6.1 | 115.9 | 0.8 |
| 4 | 150.5 | 156.7 | 6.2 | 148.5 | 2.0 |
| 5 | 126.0 | 131.5 | 5.5 | 124.3 | 1.7 |
| 6 | 119.5 | 124.7 | 5.2 | 117.7 | 1.8 |
| 7 | 203.8 | 215.0 | 11.2 | 204.7 | 0.9 |
| 8 | 42.7 | 46.5 | 3.8 | 42.4 | 0.3 |
| 9 | 74.4 | 80.8 | 6.4 | 75.4 | 1.0 |
| 10 | 173.9 | 182.5 | 8.6 | 173.3 | 0.6 |
| 11 | 27.8 | 31.2 | 3.3 | 27.6 | 0.2 |
| 12 | 22.1 | 26.2 | 3.8 | 22.9 | 0.4 |
| 13 | 81.1 | 86.8 | 5.7 | 81.2 | 0.1 |
| 14 | 78.6 | 85.7 | 7.1 | 80.1 | 1.5 |
| 15 | 22.4 | 24.0 | 1.6 | 20.8 | 1.7 |
| 16 | 40.1 | 45.2 | 5.1 | 41.1 | 1.0 |
| 17 | 23.6 | 28.9 | 5.3 | 25.5 | 1.9 |
| 18 | 124.8 | 132.3 | 7.5 | 125.1 | 0.3 |
| 19 | 133.0 | 143.9 | 10.9 | 136.2 | 3.2 |
| 20 | 25.8 | 29.0 | 3.2 | 25.6 | 0.2 |
| 21 | 17.6 | 19.3 | 1.7 | 16.2 | 1.4 |
|  |  |  |  |  |  |
| MAE |  |  | 5.9 |  |  |
| CMAE |  |  |  |  | 1.2 |

Δ*δ*_C_*^a^* = |*δ*_C, calcd_ – *δ*_C exptl_|; Δ*δ*_C_*^b^* = |*δ*_C, corr_ – *δ*_C exptl_|.

**Table S8.** Comparison of ^13^C NMR experimental values and calculated chemical shifts of **1-2**.

| No. | *δ*_C, exptl_ | *δ*_C, calcd_ | Δ*δ*_C_*^a^* | *δ*_C, corr_ | Δ*δ*_C_*^b^* |
| --- | --- | --- | --- | --- | --- |
| 1 | 156.8 | 167.6 | 10.8 | 159.2 | 2.4 |
| 2 | 121.5 | 125.9 | 4.4 | 119.0 | 2.5 |
| 3 | 116.7 | 122.5 | 5.8 | 115.7 | 1.0 |
| 4 | 150.5 | 156.6 | 6.1 | 148.7 | 1.9 |
| 5 | 126 | 131.6 | 5.6 | 124.5 | 1.5 |
| 6 | 119.5 | 124.8 | 5.3 | 117.9 | 1.6 |
| 7 | 203.8 | 214.8 | 11.0 | 204.7 | 0.9 |
| 8 | 42.7 | 46.6 | 3.9 | 42.5 | 0.2 |
| 9 | 74.4 | 80.8 | 6.4 | 75.5 | 1.1 |
| 10 | 173.9 | 182.3 | 8.4 | 173.4 | 0.5 |
| 11 | 27.8 | 31.4 | 3.6 | 28.0 | 0.1 |
| 12 | 22.1 | 26.3 | 3.9 | 23.0 | 0.6 |
| 13 | 81.1 | 84.7 | 3.6 | 79.3 | 1.8 |
| 14 | 78.6 | 85.8 | 7.2 | 80.3 | 1.7 |
| 15 | 22.4 | 25.3 | 2.9 | 22.1 | 0.3 |
| 16 | 40.1 | 43.9 | 3.8 | 40.0 | 0.1 |
| 17 | 23.6 | 28.9 | 5.3 | 25.5 | 1.9 |
| 18 | 124.8 | 132.5 | 7.7 | 125.4 | 0.6 |
| 19 | 133 | 143.8 | 10.8 | 136.3 | 3.3 |
| 20 | 25.8 | 29.1 | 3.3 | 25.7 | 0.1 |
| 21 | 17.6 | 19.4 | 1.8 | 16.4 | 1.3 |
|  |  |  |  |  |  |
| MAE |  |  | 5.8 |  |  |
| CMAE |  |  |  |  | 1.2 |

Δ*δ*_C_*^a^* = |*δ*_C, calcd_ – *δ*_C exptl_|; Δ*δ*_C_*^b^* = |*δ*_C, corr_ – *δ*_C exptl_|.

**Table S9.** Comparison of ^13^C NMR experimental values and calculated chemical shifts of **1-3**.

| No. | *δ*_C, exptl_ | *δ*_C, calcd_ | Δ*δ*_C_*^a^* | *δ*_C, corr_ | Δ*δ*_C_*^b^* |
| --- | --- | --- | --- | --- | --- |
| 1 | 156.8 | 167.0 | 10.2 | 158.7 | 1.8 |
| 2 | 121.5 | 124.3 | 2.8 | 117.3 | 4.2 |
| 3 | 116.7 | 120.2 | 3.5 | 113.3 | 3.4 |
| 4 | 150.5 | 157.1 | 6.6 | 149.0 | 1.5 |
| 5 | 126.0 | 131.7 | 5.6 | 124.4 | 1.6 |
| 6 | 119.5 | 125.1 | 5.6 | 118.1 | 1.4 |
| 7 | 203.8 | 214.5 | 10.7 | 204.5 | 0.7 |
| 8 | 42.7 | 55.2 | 12.5 | 50.5 | 7.8 |
| 9 | 74.4 | 76.7 | 2.3 | 71.2 | 3.2 |
| 10 | 173.9 | 184.0 | 10.1 | 175.1 | 1.2 |
| 11 | 27.8 | 36.3 | 8.5 | 32.1 | 4.3 |
| 12 | 22.1 | 25.6 | 3.1 | 21.7 | 0.7 |
| 13 | 81.1 | 96.6 | 15.5 | 90.5 | 9.4 |
| 14 | 78.6 | 79.8 | 1.2 | 74.2 | 4.4 |
| 15 | 22.4 | 23.8 | 1.4 | 20.1 | 2.3 |
| 16 | 40.1 | 40.1 | 0.0 | 35.8 | 4.3 |
| 17 | 23.6 | 27.7 | 4.1 | 23.9 | 0.3 |
| 18 | 124.8 | 133.3 | 8.5 | 126.0 | 1.2 |
| 19 | 133.0 | 143.3 | 10.3 | 135.7 | 2.7 |
| 20 | 25.8 | 29.1 | 3.3 | 25.2 | 0.6 |
| 21 | 17.6 | 19.4 | 1.8 | 15.8 | 1.8 |
|  |  |  |  |  |  |
| MAE |  |  | 6.1 |  |  |
| CMAE |  |  |  |  | 2.8 |

Δ*δ*_C_*^a^* = |*δ*_C, calcd_ – *δ*_C exptl_|; Δ*δ*_C_*^b^* = |*δ*_C, corr_ – *δ*_C exptl_|.

**Table S10.** Comparison of ^13^C NMR experimental values and calculated chemical shifts of **1-4**.

| No. | *δ*_C, exptl_ | *δ*_C, calcd_ | Δ*δ*_C_*^a^* | *δ*_C, corr_ | Δ*δ*_C_*^b^* |
| --- | --- | --- | --- | --- | --- |
| 1 | 156.8 | 167.0 | 10.2 | 158.8 | 2.0 |
| 2 | 121.5 | 124.4 | 2.9 | 117.4 | 4.1 |
| 3 | 116.7 | 120.2 | 3.5 | 113.3 | 3.4 |
| 4 | 150.5 | 157.1 | 6.6 | 149.1 | 1.4 |
| 5 | 126 | 131.7 | 5.7 | 124.5 | 1.5 |
| 6 | 119.5 | 125.1 | 5.6 | 118.1 | 1.4 |
| 7 | 203.8 | 214.6 | 10.8 | 204.9 | 1.1 |
| 8 | 42.7 | 55.4 | 12.7 | 50.4 | 7.7 |
| 9 | 74.4 | 76.6 | 2.2 | 71.0 | 3.4 |
| 10 | 173.9 | 184.3 | 10.4 | 175.6 | 1.7 |
| 11 | 27.8 | 36.1 | 8.3 | 31.7 | 3.9 |
| 12 | 22.1 | 25.1 | 2.7 | 21.1 | 1.3 |
| 13 | 81.1 | 92.1 | 11.0 | 86.0 | 4.9 |
| 14 | 78.6 | 79.6 | 1.0 | 73.9 | 4.7 |
| 15 | 22.4 | 24.8 | 2.4 | 20.7 | 1.7 |
| 16 | 40.1 | 44.7 | 4.6 | 40.0 | 0.1 |
| 17 | 23.6 | 28.3 | 4.7 | 24.2 | 0.5 |
| 18 | 124.8 | 133.2 | 8.4 | 126.0 | 1.2 |
| 19 | 133 | 143.5 | 10.4 | 135.9 | 2.9 |
| 20 | 25.8 | 29.1 | 3.3 | 24.9 | 0.9 |
| 21 | 17.6 | 19.4 | 1.7 | 15.5 | 2.1 |
|  |  |  |  |  |  |
| MAE |  |  | 6.1 |  |  |
| CMAE |  |  |  |  | 2.5 |

Δ*δ*_C_*^a^* = |*δ*_C, calcd_ – *δ*_C exptl_|; Δ*δ*_C_*^b^* = |*δ*_C, corr_ – *δ*_C exptl_|.

**Table S11.** Comparison of ^13^C NMR experimental values and calculated chemical shifts of **1-5**.

| No. | *δ*_C, exptl_ | *δ*_C, calcd_ | Δ*δ*_C_*^a^* | *δ*_C, corr_ | Δ*δ*_C_*^b^* |
| --- | --- | --- | --- | --- | --- |
| 1 | 156.8 | 167.5 | 10.7 | 158.8 | 2.0 |
| 2 | 121.5 | 124.6 | 3.1 | 117.5 | 4.0 |
| 3 | 116.7 | 120.4 | 3.7 | 113.5 | 3.2 |
| 4 | 150.5 | 157.2 | 6.6 | 148.8 | 1.7 |
| 5 | 126.0 | 132.1 | 6.1 | 124.7 | 1.3 |
| 6 | 119.5 | 125.4 | 5.9 | 118.3 | 1.2 |
| 7 | 203.8 | 219.1 | 15.3 | 208.3 | 4.5 |
| 8 | 42.7 | 46.2 | 3.5 | 42.1 | 0.6 |
| 9 | 74.4 | 81.8 | 7.4 | 76.4 | 2.0 |
| 10 | 173.9 | 182.3 | 8.4 | 172.9 | 1.0 |
| 11 | 27.8 | 37.2 | 9.4 | 33.5 | 5.7 |
| 12 | 22.4 | 23.9 | 1.5 | 20.7 | 1.7 |
| 13 | 81.1 | 86.3 | 5.1 | 80.7 | 0.4 |
| 14 | 78.6 | 79.1 | 0.5 | 73.8 | 4.8 |
| 15 | 22.4 | 23.6 | 1.2 | 20.5 | 1.9 |
| 16 | 40.1 | 46.8 | 6.7 | 42.7 | 2.6 |
| 17 | 23.6 | 29.7 | 6.1 | 26.3 | 2.7 |
| 18 | 124.8 | 133.0 | 8.2 | 125.6 | 0.8 |
| 19 | 133.0 | 143.4 | 10.3 | 135.5 | 2.5 |
| 20 | 25.8 | 29.1 | 3.3 | 25.8 | 0.0 |
| 21 | 17.6 | 19.4 | 1.8 | 16.4 | 1.2 |
|  |  |  |  |  |  |
| MAE |  |  | 5.9 |  |  |
| CMAE |  |  |  |  | 2.2 |

Δ*δ*_C_*^a^* = |*δ*_C, calcd_ – *δ*_C exptl_|; Δ*δ*_C_*^b^* = |*δ*_C, corr_ – *δ*_C exptl_|.

**Table S12.** Comparison of ^13^C NMR experimental values and calculated chemical shifts of **1-6**.

| No. | *δ*_C, exptl_ | *δ*_C, calcd_ | Δ*δ*_C_*^a^* | *δ*_C, corr_ | Δ*δ*_C_*^b^* |
| --- | --- | --- | --- | --- | --- |
| 1 | 156.8 | 167.6 | 10.8 | 158.7 | 1.9 |
| 2 | 121.5 | 124.6 | 3.1 | 117.4 | 4.1 |
| 3 | 116.7 | 120.4 | 3.7 | 113.4 | 3.3 |
| 4 | 150.5 | 157.2 | 6.7 | 148.7 | 1.8 |
| 5 | 126.0 | 132.2 | 6.2 | 124.7 | 1.3 |
| 6 | 119.5 | 125.4 | 5.9 | 118.2 | 1.3 |
| 7 | 203.8 | 219.2 | 15.4 | 208.1 | 4.3 |
| 8 | 42.7 | 46.0 | 3.3 | 42.1 | 0.6 |
| 9 | 74.4 | 81.8 | 7.4 | 76.5 | 2.0 |
| 10 | 173.9 | 182.2 | 8.3 | 172.7 | 1.2 |
| 11 | 27.8 | 37.1 | 9.3 | 33.6 | 5.8 |
| 12 | 22.4 | 23.7 | 1.3 | 20.7 | 1.7 |
| 13 | 81.1 | 89.5 | 8.4 | 83.8 | 2.7 |
| 14 | 78.6 | 79.0 | 0.4 | 73.7 | 4.9 |
| 15 | 22.4 | 26.5 | 4.1 | 23.4 | 1.0 |
| 16 | 40.1 | 42.6 | 2.5 | 38.9 | 1.2 |
| 17 | 23.6 | 28.2 | 4.6 | 25.0 | 1.4 |
| 18 | 124.8 | 133.0 | 8.2 | 125.5 | 0.7 |
| 19 | 133.0 | 143.6 | 10.6 | 135.6 | 2.6 |
| 20 | 25.8 | 29.1 | 3.3 | 25.9 | 0.1 |
| 21 | 17.6 | 19.3 | 1.7 | 16.5 | 1.1 |
|  |  |  |  |  |  |
| MAE |  |  | 6.0 |  |  |
| CMAE |  |  |  |  | 2.1 |

Δ*δ*_C_*^a^* = |*δ*_C, calcd_ – *δ*_C exptl_|; Δ*δ*_C_*^b^* = |*δ*_C, corr_ – *δ*_C exptl_|.


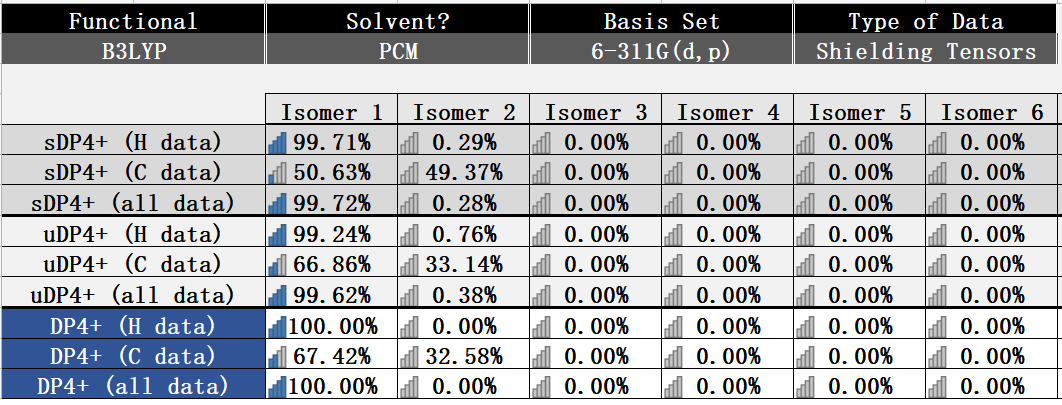


**Figure S50.** DP4+ analysis of experimental NMR data of **1** and shielding tensors of **1-1**-**1-6** (Isomer 1-6)

**ECD Computational Methods**

Conformation search were performed by using Merk Molecular Force Field (MMFF) in Gaussian 09 software package. The low energy conformations of each compounds were optimized by density functional theory (DFT) at B3LYP/6-31g(d,p) level. The ECD calculations were calculated at B3LYP/6-311G(d,p) and PBE1PBE/def2SVP level for **1** and **5**, B3LYP/6-311G(d,p) and APFD/6-311+g(2d,p) level for **2** based on Time-dependent density functional theory (TDDFT). The calculated ECD curves were averaged according to the Boltzmann distribution theory and their relative Gibbs free energy, which was generated using the SpecDis 1.62 and Origin Pro 9.0 software.


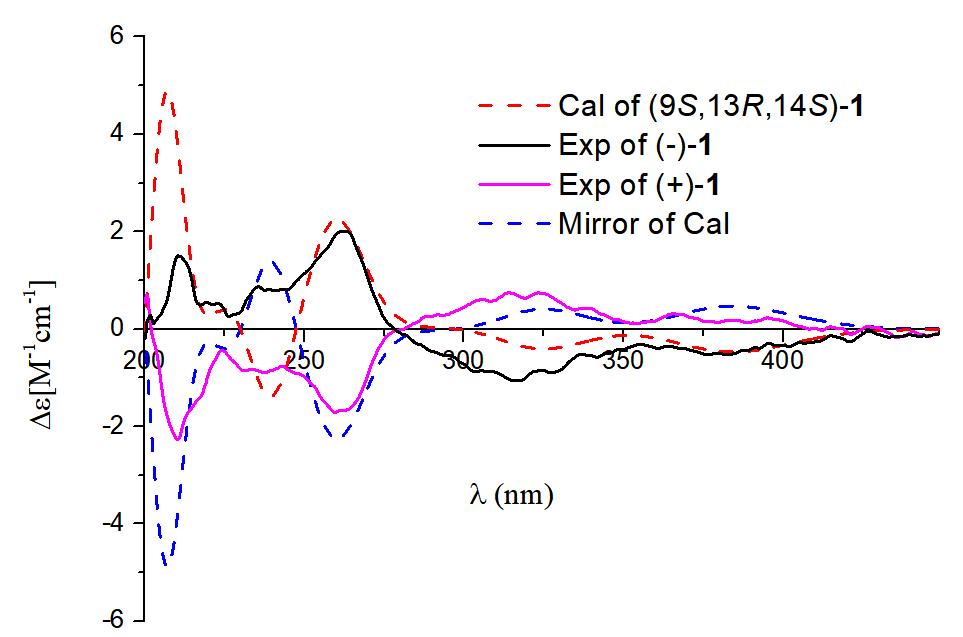


**Figure S51.** Comparison of the calculated ECD spectrum of (9*S*,13*R*,14*S*)-**1** at B3LYP/6-311G(d,p) level with the experimental one in MeOH. σ = 0.25 eV; shift = +10 nm.


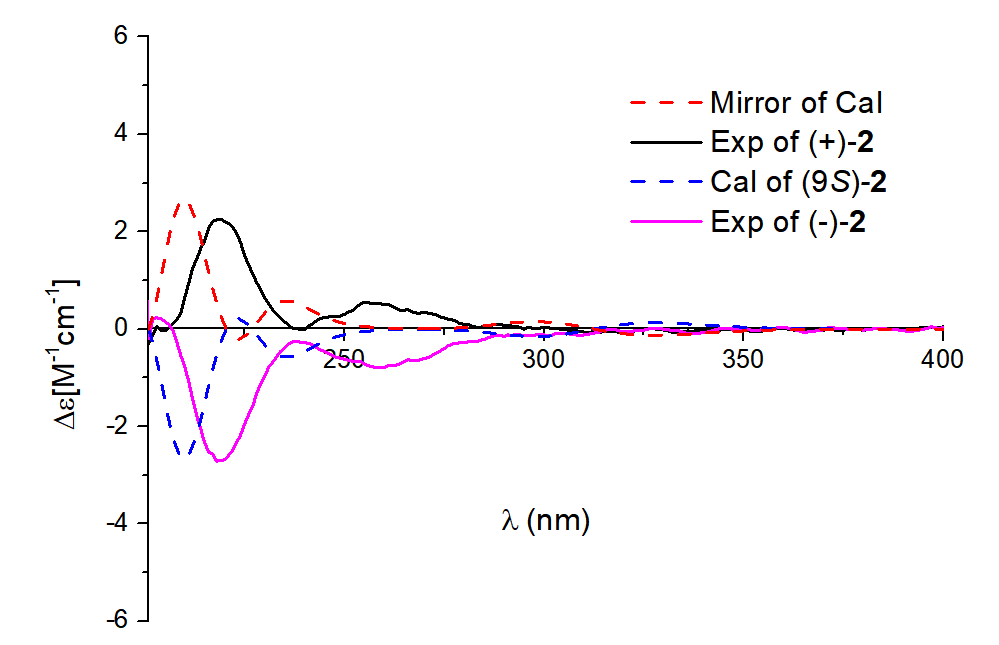


**Figure S52.** Comparison of the calculated ECD spectrum of (9*S*)-**2** at B3LYP/6-311G(d,p) level with the experimental one in MeOH. σ = 0.33 eV; shift = +30 nm.


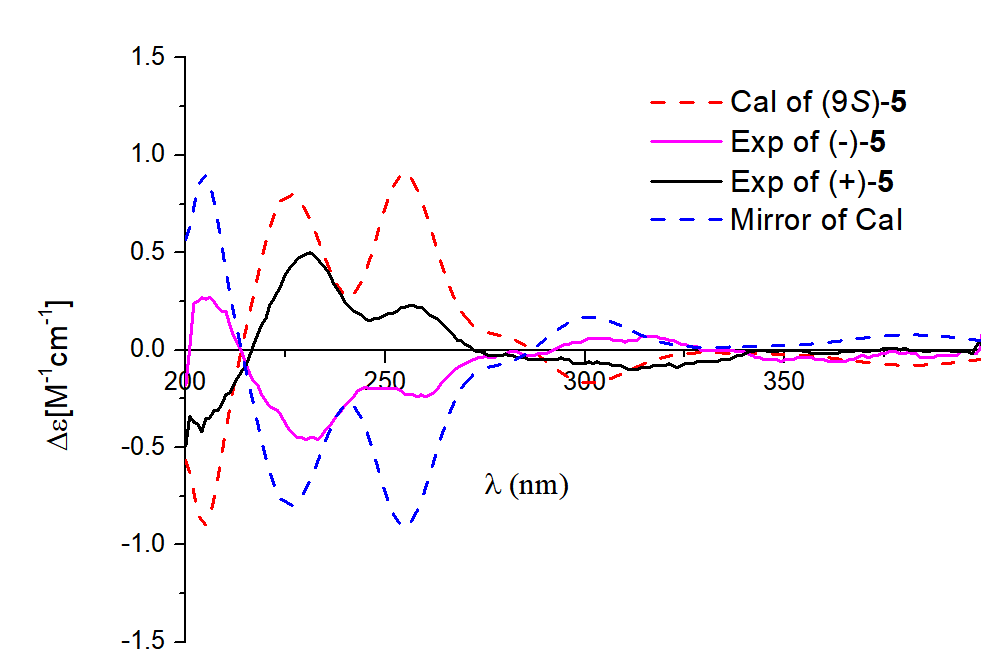


**Figure S53.** Comparison of the calculated ECD spectrum of (9*S*)-**5** at B3LYP/6-311G(d,p) level with the experimental one in MeOH. σ = 0.20 eV; shift = 0 nm.

## Specific Optical Rotation Data (ORD) Calculation Method

Conformation search of (9*S*,13*R*,14*S*)-**1** was performed by using Merk Molecular Force Field (MMFF), and then 30 low energy conformations were submitted to the density functional theory (DFT) optimization at the level of B3LYP/6-31g(d,p). The specific ORD calculations were proformed at the B3LYP/6-311+G(2d,p) level in MeOH with PCM model. The calculated specific ORD of these conformers were averaged at Boltzmann distribution theory and their relative Gibbs free energy.

**Table S13.** Specific Optical Rotation for compound **1**.

| *Confermors* | G (Hartree) | *Δ*G (Kcal/mol) | Population | [α]_D_ |
| --- | --- | --- | --- | --- |
| 1 | -1266.90854 | 0.30622 | 5.95% | 6.39 |
| 2 | -1266.90902 | 0 | 9.97% | 8.16 |
| 3 | -1266.90902 | 0.00377 | 9.91% | 13.66 |
| 4 | -1266.90847 | 0.34826 | 5.54% | -4.24 |
| 5 | -1266.90633 | 1.68987 | 0.58% | -1.11 |
| 6 | -1266.90865 | 0.23657 | 6.69% | -15.41 |
| 7 | -1266.90784 | 0.74547 | 2.83% | 1.99 |
| 8 | -1266.90835 | 0.42043 | 4.90% | -7.90 |
| 9 | -1266.90671 | 1.45141 | 0.86% | -0.35 |
| 10 | -1266.90508 | 2.47362 | 0.15% | 0.01 |
| 11 | -1266.90556 | 2.17618 | 0.25% | -0.79 |
| 12 | -1266.90641 | 1.64092 | 0.63% | -1.21 |
| 13 | -1266.90892 | 0.06526 | 8.93% | 19.55 |
| 14 | -1266.90892 | 0.06652 | 8.91% | -19.33 |
| 15 | -1266.90861 | 0.26041 | 6.42% | -3.69 |
| 16 | -1266.90883 | 0.12299 | 8.10% | 3.07 |
| 17 | -1266.90552 | 2.19563 | 0.24% | -0.28 |
| 18 | -1266.90564 | 2.12598 | 0.27% | -0.19 |
| 19 | -1266.908 | 0.64319 | 3.37% | 5.73 |
| 20 | -1266.90784 | 0.74422 | 2.84% | 1.99 |
| 21 | -1266.90556 | 2.17242 | 0.25% | -0.75 |
| 22 | -1266.90549 | 2.2176 | 0.24% | 0.06 |
| 23 | -1266.90374 | 3.31573 | 0.04% | 0.05 |
| 24 | -1266.90504 | 2.50123 | 0.15% | -0.66 |
| 25 | -1266.90781 | 0.75928 | 2.77% | 2.94 |
| 26 | -1266.90787 | 0.72665 | 2.92% | -3.31 |
| 27 | -1266.90835 | 0.42105 | 4.90% | -7.89 |
| 28 | -1266.90671 | 1.45518 | 0.86% | -0.35 |
| 29 | -1266.90625 | 1.74069 | 0.53% | -0.28 |
| Total [α]_D_ |  |  |  | -4.14 |


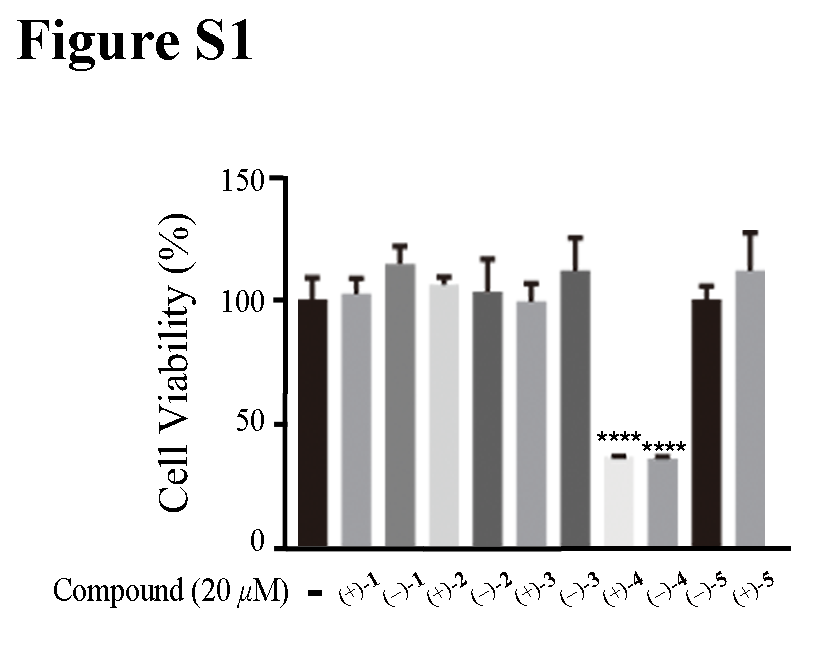


**Figure S54.** C2C12 cell proliferation in response to compounds at 20 *μ*M by CCK-8 assay. ^****^*P* < 0.0001 compared with DMSO treatment group.

**Table S14.** Cartesian Coordinates of conformers **1−1a** and **1−1b** in the gas phase (Å) at the B3LYP/6-31(d,p) level.

| **1−1a** | | | | **1−1b** | | | |
| --- | --- | --- | --- | --- | --- | --- | --- |
| C | 2.493 | -1.3129 | 1.923 | C | -3.3053 | -1.5848 | 1.6659 |
| C | 3.721 | -1.3521 | 1.2731 | C | -3.869 | -2.668 | 1.0017 |
| C | 3.7602 | -1.662 | -0.084 | C | -3.9092 | -2.6637 | -0.3904 |
| C | 2.5762 | -1.9266 | -0.7736 | C | -3.3901 | -1.58 | -1.0999 |
| C | 1.3308 | -1.8928 | -0.1197 | C | -2.8249 | -0.4778 | -0.4327 |
| C | 1.3049 | -1.5812 | 1.2446 | C | -2.7854 | -0.4963 | 0.9665 |
| C | 0.0833 | -2.176 | -0.8959 | C | -2.2867 | 0.6684 | -1.2294 |
| C | -1.2742 | -2.2112 | -0.2284 | C | -1.7766 | 1.9261 | -0.5605 |
| C | -1.9914 | -0.8638 | -0.2933 | C | -0.2645 | 1.9014 | -0.3455 |
| C | -2.1548 | -0.3112 | -1.723 | C | 0.5476 | 1.6698 | -1.6349 |
| C | -3.0095 | 0.9622 | -1.6666 | C | 2.0418 | 1.7989 | -1.3099 |
| C | -3.2592 | 1.3023 | -0.1928 | C | 2.1862 | 1.9814 | 0.2044 |
| C | -1.9333 | 1.3594 | 0.6246 | C | 1.4509 | 0.856 | 0.986 |
| C | -0.9931 | 2.4723 | 0.1039 | C | 1.9786 | -0.5659 | 0.6889 |
| C | 0.3421 | 2.5805 | 0.8519 | C | 3.459 | -0.7867 | 1.0052 |
| C | 1.2482 | 3.5897 | 0.1987 | C | 3.8752 | -2.1919 | 0.6681 |
| C | 2.2442 | 3.3397 | -0.6738 | C | 4.5918 | -2.6062 | -0.395 |
| C | 3.0982 | 4.4574 | -1.2113 | C | 4.9453 | -4.062 | -0.5603 |
| O | 2.4043 | -1.0069 | 3.2492 | O | -3.239 | -1.5474 | 3.0278 |
| O | -4.0647 | 0.2142 | 0.3269 | O | 1.5478 | 3.2447 | 0.5186 |
| C | -3.4186 | -0.9977 | 0.2861 | C | 0.2064 | 3.2577 | 0.2253 |
| O | -3.9413 | -2.0348 | 0.6678 | O | -0.4963 | 4.2418 | 0.4046 |
| C | -2.2316 | 1.5461 | 2.1213 | C | 1.4713 | 1.1296 | 2.4979 |
| C | 2.5979 | 1.9768 | -1.2043 | C | 5.0925 | -1.7216 | -1.5033 |
| O | -1.2462 | 0.0927 | 0.4871 | O | 0.0516 | 0.8544 | 0.5944 |
| O | 0.1815 | -2.3784 | -2.1104 | O | -2.2687 | 0.5705 | -2.4605 |
| O | 2.7207 | -2.2081 | -2.1073 | O | -3.4742 | -1.6733 | -2.4649 |
| H | 4.6479 | -1.1443 | 1.7981 | H | -4.2752 | -3.5169 | 1.5425 |
| H | 4.7103 | -1.695 | -0.6112 | H | -4.343 | -3.5025 | -0.9288 |
| H | 0.3799 | -1.5187 | 1.8078 | H | -2.346 | 0.3096 | 1.5447 |
| H | -1.86 | -2.9854 | -0.7422 | H | -2.0611 | 2.7666 | -1.208 |
| H | -1.1791 | -2.5674 | 0.8022 | H | -2.3132 | 2.099 | 0.3778 |
| H | -2.6255 | -1.0462 | -2.3862 | H | 0.2731 | 2.3885 | -2.4159 |
| H | -1.1797 | -0.0491 | -2.1499 | H | 0.3706 | 0.6624 | -2.0288 |
| H | -2.5242 | 1.7829 | -2.2037 | H | 2.595 | 0.9266 | -1.6718 |
| H | -3.9654 | 0.771 | -2.17 | H | 2.4448 | 2.6764 | -1.8304 |
| H | -3.8362 | 2.2276 | -0.095 | H | 3.2382 | 2.0664 | 0.492 |
| H | -0.755 | 2.2784 | -0.9489 | H | 1.3728 | -1.2907 | 1.2521 |
| H | -1.513 | 3.4384 | 0.1416 | H | 1.7835 | -0.8166 | -0.3623 |
| H | 0.1749 | 2.9104 | 1.8836 | H | 4.082 | -0.057 | 0.4824 |
| H | 0.8304 | 1.604 | 0.9237 | H | 3.6458 | -0.632 | 2.074 |
| H | 1.066 | 4.6208 | 0.5005 | H | 3.5534 | -2.9371 | 1.3966 |
| H | 2.8175 | 5.4312 | -0.7965 | H | 4.5716 | -4.6775 | 0.2648 |
| H | 4.1503 | 4.2818 | -0.9641 | H | 4.5158 | -4.4538 | -1.4882 |
| H | 3.0016 | 4.5194 | -2.3002 | H | 6.0326 | -4.1849 | -0.6002 |
| H | 3.2966 | -0.8287 | 3.5907 | H | -3.6369 | -2.3594 | 3.384 |
| H | -2.6853 | 2.5228 | 2.3173 | H | 2.4913 | 1.2407 | 2.8779 |
| H | -2.9079 | 0.7725 | 2.5003 | H | 0.9115 | 2.0366 | 2.7511 |
| H | -1.3234 | 1.4498 | 2.7256 | H | 0.9828 | 0.3155 | 3.0459 |
| H | 1.8652 | 1.2111 | -0.9406 | H | 4.7288 | -0.6939 | -1.4368 |
| H | 2.649 | 1.9995 | -2.2982 | H | 6.1869 | -1.6926 | -1.4963 |
| H | 3.5738 | 1.6643 | -0.8197 | H | 4.7643 | -2.1118 | -2.4728 |
| H | 1.8234 | -2.36 | -2.482 | H | -3.0512 | -0.8717 | -2.8481 |

**Table S15.** Cartesian Coordinates of conformers **1−1c** and **1−1d** in the gas phase (Å) at the B3LYP/6-31(d,p) level.

| **1−1c** | | | | **1−1d** | | | |
| --- | --- | --- | --- | --- | --- | --- | --- |
| C | 2.5233 | -1.2482 | 1.9208 | C | -0.2488 | 2.9924 | 1.7189 |
| C | 3.7483 | -1.3197 | 1.2701 | C | -0.7502 | 4.0324 | 0.9452 |
| C | 3.7901 | -1.6687 | -0.0764 | C | -0.2777 | 4.1995 | -0.3541 |
| C | 2.6053 | -1.9424 | -0.7589 | C | 0.6861 | 3.3284 | -0.8635 |
| C | 1.3601 | -1.8799 | -0.1056 | C | 1.1969 | 2.2735 | -0.0852 |
| C | 1.3317 | -1.5267 | 1.2511 | C | 0.7159 | 2.1179 | 1.2203 |
| C | 0.1116 | -2.1787 | -0.8756 | C | 2.2208 | 1.3533 | -0.6725 |
| C | -1.2425 | -2.2228 | -0.2012 | C | 2.769 | 0.1769 | 0.1057 |
| C | -1.9806 | -0.8873 | -0.2822 | C | 1.9539 | -1.0955 | -0.1198 |
| C | -2.1478 | -0.3543 | -1.7187 | C | 1.8248 | -1.4971 | -1.6027 |
| C | -3.0288 | 0.9009 | -1.6836 | C | 1.1108 | -2.8527 | -1.6882 |
| C | -3.2874 | 1.2572 | -0.216 | C | 0.6546 | -3.2393 | -0.2767 |
| C | -1.9639 | 1.3507 | 0.6028 | C | -0.1682 | -2.102 | 0.4012 |
| C | -1.0387 | 2.4652 | 0.0592 | C | -1.468 | -1.7968 | -0.3808 |
| C | 0.2838 | 2.6242 | 0.8202 | C | -2.3331 | -0.6834 | 0.2249 |
| C | 1.1796 | 3.6265 | 0.1438 | C | -3.4912 | -0.3509 | -0.6775 |
| C | 2.2093 | 3.365 | -0.6849 | C | -4.7699 | -0.7577 | -0.5565 |
| C | 3.048 | 4.4811 | -1.2497 | C | -5.8181 | -0.296 | -1.5352 |
| O | 2.5304 | -0.8919 | 3.2374 | O | -0.6853 | 2.7859 | 2.9946 |
| O | -4.0734 | 0.1624 | 0.3192 | O | 1.8727 | -3.4338 | 0.4864 |
| C | -3.4048 | -1.0373 | 0.2995 | C | 2.6256 | -2.2896 | 0.5958 |
| O | -3.9026 | -2.0754 | 0.7103 | O | 3.6927 | -2.2584 | 1.1913 |
| C | -2.2702 | 1.5658 | 2.094 | C | -0.4743 | -2.4607 | 1.8646 |
| C | 2.6213 | 1.99 | -1.136 | C | -5.2837 | -1.686 | 0.5091 |
| O | -1.2575 | 0.0894 | 0.4977 | O | 0.6321 | -0.8966 | 0.4208 |
| O | 0.205 | -2.3899 | -2.0885 | O | 2.6033 | 1.5619 | -1.8282 |
| O | 2.7471 | -2.2632 | -2.0838 | O | 1.0826 | 3.5757 | -2.1523 |
| H | 4.6667 | -1.1003 | 1.8076 | H | -1.5012 | 4.7152 | 1.3298 |
| H | 4.7423 | -1.7237 | -0.5972 | H | -0.6573 | 5.0067 | -0.9755 |
| H | 0.3977 | -1.4458 | 1.794 | H | 1.0538 | 1.3193 | 1.8718 |
| H | -1.8189 | -3.0127 | -0.7019 | H | 3.8073 | 0.0332 | -0.2226 |
| H | -1.1381 | -2.5636 | 0.8338 | H | 2.8415 | 0.4275 | 1.1685 |
| H | -2.5991 | -1.1067 | -2.3759 | H | 2.8058 | -1.562 | -2.0876 |
| H | -1.1757 | -0.0771 | -2.1432 | H | 1.2268 | -0.7607 | -2.1522 |
| H | -2.5597 | 1.7234 | -2.2322 | H | 0.2766 | -2.8114 | -2.3953 |
| H | -3.9797 | 0.6822 | -2.1852 | H | 1.816 | -3.6023 | -2.0682 |
| H | -3.8813 | 2.1732 | -0.1328 | H | 0.1058 | -4.1866 | -0.2863 |
| H | -0.7832 | 2.2435 | -0.984 | H | -1.2117 | -1.4839 | -1.4003 |
| H | -1.5777 | 3.4215 | 0.0608 | H | -2.064 | -2.7146 | -0.4679 |
| H | 0.0959 | 2.9876 | 1.837 | H | -2.6875 | -0.961 | 1.221 |
| H | 0.7871 | 1.6595 | 0.9337 | H | -1.7398 | 0.2286 | 0.3577 |
| H | 0.9597 | 4.6656 | 0.3887 | H | -3.2327 | 0.3137 | -1.5027 |
| H | 2.7262 | 5.4648 | -0.8917 | H | -5.4108 | 0.3832 | -2.2916 |
| H | 4.0961 | 4.3495 | -0.9618 | H | -6.253 | -1.1538 | -2.0584 |
| H | 2.9848 | 4.4881 | -2.3428 | H | -6.6189 | 0.2344 | -1.0095 |
| H | 1.6173 | -0.8522 | 3.566 | H | -1.358 | 3.454 | 3.2082 |
| H | -2.737 | 2.5406 | 2.2674 | H | -1.1534 | -3.3168 | 1.9303 |
| H | -2.9385 | 0.7919 | 2.4867 | H | 0.4364 | -2.7036 | 2.4225 |
| H | -1.3633 | 1.4938 | 2.7036 | H | -0.917 | -1.6148 | 2.4008 |
| H | 1.9001 | 1.2165 | -0.8641 | H | -4.4923 | -2.1152 | 1.1281 |
| H | 2.7142 | 1.963 | -2.2271 | H | -5.9779 | -1.1549 | 1.1683 |
| H | 3.5898 | 1.7246 | -0.7006 | H | -5.8176 | -2.5255 | 0.0507 |
| H | 1.8492 | -2.4068 | -2.4592 | H | 1.7685 | 2.911 | -2.3892 |

**Table S16.** Cartesian Coordinates of conformers **1−1e** and **1−1f** in the gas phase (Å) at the B3LYP/6-31(d,p) level.

| **1−1e** | | | | **1−1f** | | | |
| --- | --- | --- | --- | --- | --- | --- | --- |
| C | -3.729 | -1.7086 | 1.1799 | C | -2.5279 | -2.3573 | 1.5909 |
| C | -4.6348 | -2.2115 | 0.2532 | C | -3.0789 | -3.367 | 0.8104 |
| C | -4.7946 | -1.5543 | -0.9643 | C | -3.4714 | -3.0777 | -0.4942 |
| C | -4.0525 | -0.405 | -1.2393 | C | -3.3119 | -1.7874 | -1.0009 |
| C | -3.1399 | 0.1127 | -0.302 | C | -2.7612 | -0.76 | -0.213 |
| C | -2.9839 | -0.56 | 0.9158 | C | -2.3657 | -1.0643 | 1.0949 |
| C | -2.3694 | 1.3522 | -0.6315 | C | -2.6138 | 0.6123 | -0.7906 |
| C | -1.4644 | 2.0142 | 0.3844 | C | -2.1355 | 1.7766 | 0.049 |
| C | -0.0097 | 1.5686 | 0.2481 | C | -0.6309 | 2.0084 | -0.0773 |
| C | 0.588 | 1.8058 | -1.1517 | C | -0.1499 | 2.223 | -1.5258 |
| C | 2.0788 | 1.4445 | -1.1213 | C | 1.3445 | 2.5717 | -1.5054 |
| C | 2.3982 | 0.8487 | 0.2536 | C | 1.8418 | 2.4557 | -0.0609 |
| C | 1.4448 | -0.3267 | 0.6064 | C | 1.4975 | 1.0677 | 0.5486 |
| C | 1.4846 | -1.53 | -0.3672 | C | 2.1333 | -0.125 | -0.2013 |
| C | 2.7734 | -2.3588 | -0.4237 | C | 3.6591 | -0.0848 | -0.3041 |
| C | 3.882 | -1.7068 | -1.1996 | C | 4.1823 | -1.2938 | -1.0293 |
| C | 5.1231 | -1.3912 | -0.7803 | C | 4.757 | -2.3872 | -0.4913 |
| C | 6.1104 | -0.7559 | -1.7267 | C | 5.2595 | -3.5024 | -1.3713 |
| O | -3.5347 | -2.3218 | 2.3828 | O | -2.1243 | -2.5949 | 2.8721 |
| O | 2.1747 | 1.9182 | 1.2113 | O | 1.1313 | 3.4714 | 0.6909 |
| C | 0.8749 | 2.3569 | 1.2375 | C | -0.2281 | 3.2786 | 0.7048 |
| O | 0.5007 | 3.2649 | 1.9656 | O | -0.9947 | 4.0422 | 1.2738 |
| C | 1.6655 | -0.7983 | 2.0529 | C | 1.8584 | 1.0143 | 2.0417 |
| C | 5.6638 | -1.6148 | 0.6055 | C | 4.9451 | -2.6301 | 0.9811 |
| O | 0.0731 | 0.1623 | 0.5534 | O | 0.0601 | 0.8692 | 0.474 |
| O | -2.4889 | 1.8341 | -1.7624 | O | -2.8957 | 0.7815 | -1.9811 |
| O | -4.2793 | 0.1647 | -2.4653 | O | -3.7253 | -1.6061 | -2.2952 |
| H | -5.217 | -3.1049 | 0.4553 | H | -3.2073 | -4.3745 | 1.1931 |
| H | -5.4955 | -1.9332 | -1.7038 | H | -3.9017 | -3.854 | -1.1218 |
| H | -2.2835 | -0.229 | 1.6752 | H | -1.9149 | -0.327 | 1.7507 |
| H | -1.5528 | 3.0981 | 0.2297 | H | -2.6924 | 2.6609 | -0.2891 |
| H | -1.8442 | 1.8423 | 1.3965 | H | -2.4344 | 1.6375 | 1.0928 |
| H | 0.4636 | 2.8474 | -1.47 | H | -0.7124 | 3.0236 | -2.0203 |
| H | 0.0979 | 1.1669 | -1.8954 | H | -0.2781 | 1.3087 | -2.1166 |
| H | 2.3256 | 0.7591 | -1.9373 | H | 1.9044 | 1.9207 | -2.1844 |
| H | 2.6687 | 2.3562 | -1.2764 | H | 1.4748 | 3.6007 | -1.8623 |
| H | 3.4504 | 0.5721 | 0.3331 | H | 2.9097 | 2.6836 | 0.0075 |
| H | 0.6708 | -2.213 | -0.0815 | H | 1.8239 | -1.0559 | 0.2957 |
| H | 1.2056 | -1.2042 | -1.3776 | H | 1.6975 | -0.2022 | -1.2064 |
| H | 3.0769 | -2.6593 | 0.5809 | H | 3.9778 | 0.7997 | -0.8675 |
| H | 2.5406 | -3.2968 | -0.9453 | H | 4.115 | 0.0101 | 0.6848 |
| H | 3.6292 | -1.5057 | -2.242 | H | 4.0825 | -1.243 | -2.114 |
| H | 5.6908 | -0.6049 | -2.7269 | H | 5.1129 | -3.2908 | -2.4357 |
| H | 6.4224 | 0.2225 | -1.347 | H | 6.3309 | -3.659 | -1.2085 |
| H | 6.9974 | -1.3893 | -1.8304 | H | 4.732 | -4.4341 | -1.142 |
| H | -4.118 | -3.0972 | 2.4381 | H | -2.2912 | -3.5282 | 3.0858 |
| H | 2.7054 | -1.0855 | 2.2334 | H | 2.9132 | 1.2471 | 2.2153 |
| H | 1.3963 | -0.0212 | 2.7767 | H | 1.2533 | 1.7147 | 2.6278 |
| H | 1.0223 | -1.6552 | 2.2843 | H | 1.649 | 0.0213 | 2.4562 |
| H | 4.9402 | -2.0573 | 1.2921 | H | 4.4546 | -1.8835 | 1.6099 |
| H | 6.5305 | -2.283 | 0.567 | H | 4.5241 | -3.6027 | 1.2583 |
| H | 5.9827 | -0.6619 | 1.0413 | H | 6.0115 | -2.6332 | 1.2288 |
| H | -3.6825 | 0.9426 | -2.5508 | H | -3.5353 | -0.6732 | -2.544 |

**Table S17.** Cartesian Coordinates of conformers **1−1g** and **1−1h** in the gas phase (Å) at the B3LYP/6-31(d,p) level.

| **1−1g** | | | | **1−1h** | | | |
| --- | --- | --- | --- | --- | --- | --- | --- |
| C | 5.2985 | 1.9328 | 0.274 | C | 5.1394 | 2.236 | 0.225 |
| C | 6.374 | 1.1626 | -0.1547 | C | 6.3119 | 1.5056 | 0.0656 |
| C | 6.1314 | -0.0833 | -0.7297 | C | 6.2319 | 0.1616 | -0.2933 |
| C | 4.8222 | -0.5454 | -0.8667 | C | 4.9862 | -0.4364 | -0.4852 |
| C | 3.7339 | 0.2264 | -0.425 | C | 3.7993 | 0.2966 | -0.3147 |
| C | 3.9867 | 1.4807 | 0.144 | C | 3.8894 | 1.6488 | 0.0379 |
| C | 2.3409 | -0.2906 | -0.5815 | C | 2.4769 | -0.3663 | -0.5247 |
| C | 1.1936 | 0.4564 | 0.074 | C | 1.2068 | 0.3738 | -0.1464 |
| C | -0.1221 | -0.3208 | 0.1558 | C | -0.0496 | -0.496 | -0.0651 |
| C | -0.7628 | -0.6414 | -1.2088 | C | -0.4926 | -1.1096 | -1.4078 |
| C | -2.0368 | -1.4643 | -0.9786 | C | -1.7251 | -1.9911 | -1.1685 |
| C | -2.2937 | -1.5349 | 0.5281 | C | -2.1577 | -1.8223 | 0.2896 |
| C | -2.3156 | -0.1152 | 1.1622 | C | -2.3583 | -0.3225 | 0.6453 |
| C | -3.424 | 0.8058 | 0.6017 | C | -3.4573 | 0.3783 | -0.1868 |
| C | -4.8516 | 0.2851 | 0.7798 | C | -4.8446 | -0.2602 | -0.0952 |
| C | -5.8495 | 1.244 | 0.191 | C | -5.8452 | 0.5036 | -0.9183 |
| C | -6.5222 | 1.1226 | -0.9701 | C | -6.7761 | 1.3724 | -0.4778 |
| C | -7.5164 | 2.1734 | -1.3931 | C | -7.7303 | 2.0297 | -1.4411 |
| O | 5.4868 | 3.1613 | 0.8356 | O | 5.1685 | 3.5556 | 0.5681 |
| O | -1.1832 | -2.2732 | 1.0974 | O | -1.0748 | -2.3444 | 1.1 |
| C | 0.0227 | -1.6289 | 0.963 | C | 0.0887 | -1.6238 | 0.9805 |
| O | 1.0495 | -2.0435 | 1.4815 | O | 1.0712 | -1.8442 | 1.674 |
| C | -2.4118 | -0.1933 | 2.6942 | C | -2.6302 | -0.1393 | 2.1474 |
| C | -6.3678 | -0.0085 | -1.9491 | C | -6.9619 | 1.7883 | 0.9557 |
| O | -1.0552 | 0.5467 | 0.8638 | O | -1.1231 | 0.3918 | 0.3632 |
| O | 2.1701 | -1.3001 | -1.2657 | O | 2.4646 | -1.4905 | -1.0268 |
| O | 4.684 | -1.7769 | -1.4508 | O | 5.0097 | -1.7583 | -0.8443 |
| H | 7.3972 | 1.5106 | -0.0546 | H | 7.2874 | 1.9589 | 0.2112 |
| H | 6.9587 | -0.6988 | -1.074 | H | 7.1373 | -0.4253 | -0.426 |
| H | 3.1885 | 2.1299 | 0.4894 | H | 3.0104 | 2.2718 | 0.1685 |
| H | 1.4907 | 0.724 | 1.0962 | H | 1.3556 | 0.8426 | 0.8348 |
| H | 1.0325 | 1.3923 | -0.4749 | H | 1.0477 | 1.1801 | -0.8729 |
| H | -0.1027 | -1.2017 | -1.8755 | H | 0.2824 | -1.7167 | -1.8821 |
| H | -1.0296 | 0.2899 | -1.7236 | H | -0.7576 | -0.3122 | -2.1131 |
| H | -2.8827 | -1.0303 | -1.5208 | H | -2.5271 | -1.7349 | -1.8679 |
| H | -1.8835 | -2.4759 | -1.3743 | H | -1.4554 | -3.0381 | -1.3543 |
| H | -3.2047 | -2.0995 | 0.7466 | H | -3.0467 | -2.4209 | 0.5092 |
| H | -3.3341 | 1.7934 | 1.0769 | H | -3.5171 | 1.4303 | 0.1278 |
| H | -3.2329 | 1.0039 | -0.4612 | H | -3.1448 | 0.4321 | -1.2382 |
| H | -4.9606 | -0.7107 | 0.3435 | H | -4.8163 | -1.2854 | -0.4817 |
| H | -5.0852 | 0.1749 | 1.845 | H | -5.1696 | -0.3414 | 0.9452 |
| H | -6.0378 | 2.1233 | 0.8082 | H | -5.7954 | 0.2973 | -1.9878 |
| H | -7.6098 | 2.9786 | -0.6568 | H | -7.5721 | 1.7003 | -2.4735 |
| H | -7.2103 | 2.6252 | -2.3424 | H | -8.7642 | 1.7911 | -1.1713 |
| H | -8.5072 | 1.726 | -1.5232 | H | -7.6055 | 3.1173 | -1.4165 |
| H | 6.441 | 3.3409 | 0.8839 | H | 6.0941 | 3.8284 | 0.6867 |
| H | -3.2884 | -0.7623 | 3.0182 | H | -3.4929 | -0.7242 | 2.4799 |
| H | -1.5221 | -0.6577 | 3.1328 | H | -1.7661 | -0.4313 | 2.7539 |
| H | -2.4687 | 0.8107 | 3.1306 | H | -2.8149 | 0.9154 | 2.3822 |
| H | -5.5595 | -0.697 | -1.6935 | H | -6.18 | 1.4143 | 1.6205 |
| H | -7.2964 | -0.5856 | -2.0067 | H | -6.9483 | 2.8808 | 1.0348 |
| H | -6.1446 | 0.3857 | -2.9464 | H | -7.9254 | 1.4279 | 1.3302 |
| H | 3.722 | -1.9731 | -1.5158 | H | 4.0797 | -2.0508 | -0.9771 |

**Table S18.** Cartesian Coordinates of conformers **1−1i** and **1−1j** in the gas phase (Å) at the B3LYP/6-31(d,p) level.

| **1−1i** | | | | **1−1j** | | | |
| --- | --- | --- | --- | --- | --- | --- | --- |
| C | 5.5467 | 1.4224 | -0.1013 | C | -3.749 | -1.1699 | 1.5317 |
| C | 6.487 | 0.4089 | 0.0483 | C | -4.5347 | -2.0408 | 0.7858 |
| C | 6.0621 | -0.9182 | 0.0464 | C | -4.5485 | -1.9222 | -0.6017 |
| C | 4.7074 | -1.2168 | -0.1011 | C | -3.7821 | -0.9367 | -1.2253 |
| C | 3.7536 | -0.1942 | -0.2419 | C | -2.9907 | -0.0479 | -0.4749 |
| C | 4.1906 | 1.1361 | -0.2475 | C | -2.981 | -0.1807 | 0.9186 |
| C | 2.3081 | -0.5392 | -0.3972 | C | -2.1896 | 1.0002 | -1.1808 |
| C | 1.2708 | 0.5678 | -0.3451 | C | -1.4101 | 2.048 | -0.4167 |
| C | -0.1721 | 0.0951 | -0.1545 | C | 0.0502 | 1.6515 | -0.2074 |
| C | -0.7342 | -0.7353 | -1.3239 | C | 0.8073 | 1.3442 | -1.5131 |
| C | -2.1587 | -1.1835 | -0.9744 | C | 2.2846 | 1.0975 | -1.1821 |
| C | -2.5644 | -0.5093 | 0.3379 | C | 2.4398 | 1.1018 | 0.3422 |
| C | -2.3721 | 1.0309 | 0.2682 | C | 1.4501 | 0.1158 | 1.027 |
| C | -3.2064 | 1.7537 | -0.8186 | C | 1.6004 | -1.3681 | 0.6118 |
| C | -4.7311 | 1.7679 | -0.6555 | C | 2.8279 | -2.1393 | 1.1095 |
| C | -5.3954 | 0.4744 | -1.0321 | C | 4.1501 | -1.6399 | 0.6061 |
| C | -6.186 | -0.3111 | -0.2747 | C | 4.7247 | -1.9182 | -0.5801 |
| C | -6.7829 | -1.5722 | -0.8467 | C | 6.0772 | -1.3565 | -0.9328 |
| O | 5.9164 | 2.735 | -0.1133 | O | -3.6995 | -1.2519 | 2.8924 |
| O | -1.6719 | -1.0364 | 1.356 | O | 2.1064 | 2.4494 | 0.7709 |
| C | -0.3574 | -0.6943 | 1.1579 | C | 0.8136 | 2.8044 | 0.4786 |
| O | 0.5186 | -0.9524 | 1.971 | O | 0.3582 | 3.9079 | 0.7421 |
| C | -2.6077 | 1.6795 | 1.6424 | C | 1.5015 | 0.2553 | 2.5565 |
| C | -6.5596 | -0.0559 | 1.16 | C | 4.1133 | -2.7813 | -1.6499 |
| O | -0.9805 | 1.3044 | -0.0733 | O | 0.0949 | 0.4857 | 0.639 |
| O | 2.0109 | -1.7183 | -0.5904 | O | -2.1773 | 0.9966 | -2.4158 |
| O | 4.3881 | -2.5491 | -0.0993 | O | -3.8613 | -0.9052 | -2.5935 |
| H | 7.5439 | 0.6275 | 0.1632 | H | -5.1344 | -2.8116 | 1.2594 |
| H | 6.7823 | -1.7247 | 0.1586 | H | -5.154 | -2.5954 | -1.2033 |
| H | 3.5053 | 1.9689 | -0.3687 | H | -2.3784 | 0.4564 | 1.5574 |
| H | 1.5179 | 1.2383 | 0.4879 | H | -1.4786 | 2.9793 | -0.9953 |
| H | 1.3453 | 1.1541 | -1.2692 | H | -1.9068 | 2.2696 | 0.5333 |
| H | -0.1367 | -1.6213 | -1.5521 | H | 0.7191 | 2.1672 | -2.2318 |
| H | -0.7677 | -0.1229 | -2.2335 | H | 0.409 | 0.4399 | -1.9877 |
| H | -2.8469 | -0.951 | -1.7922 | H | 2.6304 | 0.1621 | -1.6311 |
| H | -2.167 | -2.273 | -0.8459 | H | 2.8859 | 1.905 | -1.6179 |
| H | -3.5755 | -0.7907 | 0.6349 | H | 3.4773 | 0.9291 | 0.6334 |
| H | -2.8728 | 2.8017 | -0.8479 | H | 0.714 | -1.9076 | 0.9777 |
| H | -2.9408 | 1.3701 | -1.8122 | H | 1.5239 | -1.4587 | -0.4784 |
| H | -5.004 | 2.1112 | 0.3441 | H | 2.8516 | -2.1403 | 2.2058 |
| H | -5.1292 | 2.5301 | -1.3387 | H | 2.7118 | -3.1959 | 0.8371 |
| H | -5.2222 | 0.1764 | -2.0675 | H | 4.696 | -1.0133 | 1.312 |
| H | -6.4948 | -1.7331 | -1.8909 | H | 6.4901 | -0.734 | -0.1321 |
| H | -6.4489 | -2.4428 | -0.2728 | H | 6.787 | -2.1681 | -1.1232 |
| H | -7.8761 | -1.5261 | -0.8068 | H | 6.0064 | -0.7364 | -1.8325 |
| H | 6.878 | 2.7909 | 0.0187 | H | -4.283 | -1.9718 | 3.1852 |
| H | -3.5951 | 1.4341 | 2.0441 | H | 2.5117 | 0.0947 | 2.9451 |
| H | -1.8553 | 1.3656 | 2.3739 | H | 1.1626 | 1.2441 | 2.884 |
| H | -2.5202 | 2.7703 | 1.5769 | H | 0.8257 | -0.464 | 3.0339 |
| H | -6.1081 | 0.8461 | 1.5765 | H | 3.125 | -3.1639 | -1.3835 |
| H | -7.6458 | 0.0481 | 1.2523 | H | 4.0021 | -2.2084 | -2.5765 |
| H | -6.2396 | -0.8962 | 1.7854 | H | 4.7574 | -3.6435 | -1.8517 |
| H | 3.4172 | -2.6262 | -0.24 | H | -3.2622 | -0.1929 | -2.9133 |

**Table S19.** Cartesian Coordinates of conformers **1−1k** and **1−1l** in the gas phase (Å) at the B3LYP/6-31(d,p) level.

| **1−1k** | | | | **1−1l** | | | |
| --- | --- | --- | --- | --- | --- | --- | --- |
| C | 3.2744 | 2.2158 | 1.0997 | C | -3.6747 | -1.7311 | 1.185 |
| C | 4.2502 | 2.683 | 0.227 | C | -4.5701 | -2.2466 | 0.2567 |
| C | 4.6817 | 1.8606 | -0.8108 | C | -4.7517 | -1.5913 | -0.9578 |
| C | 4.1374 | 0.5846 | -0.9622 | C | -4.0363 | -0.4259 | -1.231 |
| C | 3.1551 | 0.1037 | -0.0771 | C | -3.1322 | 0.1074 | -0.2935 |
| C | 2.7252 | 0.942 | 0.9584 | C | -2.957 | -0.5634 | 0.9257 |
| C | 2.6031 | -1.2737 | -0.2678 | C | -2.3852 | 1.3619 | -0.6254 |
| C | 1.6392 | -1.8837 | 0.7261 | C | -1.4748 | 2.0301 | 0.382 |
| C | 0.1798 | -1.6942 | 0.3165 | C | -0.0211 | 1.5803 | 0.2438 |
| C | -0.1558 | -2.2544 | -1.079 | C | 0.5636 | 1.7891 | -1.1657 |
| C | -1.665 | -2.1139 | -1.3185 | C | 2.0553 | 1.4341 | -1.1425 |
| C | -2.2712 | -1.3633 | -0.1276 | C | 2.3876 | 0.8609 | 0.2383 |
| C | -1.5422 | -0.0143 | 0.1251 | C | 1.4382 | -0.3093 | 0.6195 |
| C | -1.603 | 0.9741 | -1.0655 | C | 1.4609 | -1.5248 | -0.3395 |
| C | -2.9753 | 1.2647 | -1.6863 | C | 2.7462 | -2.3586 | -0.4034 |
| C | -3.8972 | 2.0458 | -0.7965 | C | 3.8489 | -1.7163 | -1.1952 |
| C | -5.1479 | 1.7305 | -0.4062 | C | 5.0948 | -1.4002 | -0.7905 |
| C | -5.9181 | 2.656 | 0.5021 | C | 6.0745 | -0.7762 | -1.7523 |
| O | 2.8168 | 2.9877 | 2.127 | O | -3.522 | -2.4119 | 2.3571 |
| O | -2.0689 | -2.2154 | 1.0282 | O | 2.1714 | 1.9455 | 1.1811 |
| C | -0.7435 | -2.434 | 1.3105 | C | 0.8714 | 2.3824 | 1.2138 |
| O | -0.3761 | -3.1389 | 2.2393 | O | 0.499 | 3.2909 | 1.942 |
| C | -2.0227 | 0.6401 | 1.4299 | C | 1.6815 | -0.763 | 2.0681 |
| C | -5.9054 | 0.4914 | -0.7964 | C | 5.6489 | -1.6138 | 0.5915 |
| O | -0.125 | -0.2851 | 0.3304 | O | 0.0648 | 0.1807 | 0.5848 |
| O | 2.9559 | -1.9147 | -1.2629 | O | -2.5236 | 1.8488 | -1.7516 |
| O | 4.6252 | -0.1442 | -2.0157 | O | -4.2781 | 0.1431 | -2.4543 |
| H | 4.6806 | 3.6736 | 0.3342 | H | -5.1222 | -3.1563 | 0.4756 |
| H | 5.4417 | 2.2084 | -1.5059 | H | -5.4474 | -1.9865 | -1.6932 |
| H | 1.9562 | 0.6445 | 1.6635 | H | -2.2598 | -0.2034 | 1.6727 |
| H | 1.8864 | -2.9518 | 0.7934 | H | -1.5607 | 3.1128 | 0.2176 |
| H | 1.8295 | -1.487 | 1.7285 | H | -1.8527 | 1.8686 | 1.3966 |
| H | 0.1397 | -3.3062 | -1.1702 | H | 0.4326 | 2.8232 | -1.5052 |
| H | 0.3668 | -1.6905 | -1.8602 | H | 0.0683 | 1.1329 | -1.8908 |
| H | -1.8594 | -1.6036 | -2.267 | H | 2.298 | 0.7375 | -1.9501 |
| H | -2.1084 | -3.1145 | -1.392 | H | 2.6406 | 2.3455 | -1.3162 |
| H | -3.3496 | -1.2327 | -0.2523 | H | 3.4408 | 0.5864 | 0.3125 |
| H | -1.1274 | 1.9201 | -0.7698 | H | 0.649 | -2.2016 | -0.0341 |
| H | -0.9531 | 0.596 | -1.8668 | H | 1.1692 | -1.2109 | -1.35 |
| H | -2.811 | 1.873 | -2.5854 | H | 3.059 | -2.6525 | 0.6004 |
| H | -3.4229 | 0.3352 | -2.0434 | H | 2.5048 | -3.2998 | -0.9152 |
| H | -3.4774 | 2.9915 | -0.4492 | H | 3.5871 | -1.5243 | -2.2371 |
| H | -5.3424 | 3.5466 | 0.7753 | H | 5.6454 | -0.6323 | -2.7495 |
| H | -6.8368 | 2.9922 | 0.0102 | H | 6.3934 | 0.2044 | -1.3841 |
| H | -6.1877 | 2.1384 | 1.4286 | H | 6.9585 | -1.4133 | -1.8595 |
| H | 3.2841 | 3.8398 | 2.1123 | H | -2.8644 | -1.9584 | 2.9098 |
| H | -3.1108 | 0.7259 | 1.4734 | H | 2.7235 | -1.0504 | 2.2354 |
| H | -1.6986 | 0.0696 | 2.3076 | H | 1.4259 | 0.0241 | 2.786 |
| H | -1.587 | 1.6386 | 1.5498 | H | 1.0401 | -1.6153 | 2.3209 |
| H | -5.3664 | -0.1505 | -1.4953 | H | 4.9319 | -2.0511 | 1.2882 |
| H | -6.1298 | -0.1062 | 0.0935 | H | 6.515 | -2.2827 | 0.5495 |
| H | -6.8518 | 0.765 | -1.275 | H | 5.9723 | -0.6578 | 1.017 |
| H | 4.1467 | -1.0039 | -2.0343 | H | -3.7017 | 0.9357 | -2.5396 |

**Table S20.** Cartesian Coordinates of conformers **1−2a** and **1−2b** in the gas phase (Å) at the B3LYP/6-31(d,p) level.

| **1−2a** | | | | **1−2b** | | | |
| --- | --- | --- | --- | --- | --- | --- | --- |
| C | 1.777 | 2.827 | -0.0592 | C | 2.8234 | 2.7309 | -0.3305 |
| C | 2.9818 | 3.2039 | 0.5221 | C | 4.0908 | 2.9151 | 0.2099 |
| C | 3.9867 | 2.2519 | 0.6776 | C | 4.8861 | 1.8014 | 0.4685 |
| C | 3.7774 | 0.9397 | 0.2506 | C | 4.4088 | 0.5217 | 0.1824 |
| C | 2.5618 | 0.5543 | -0.3429 | C | 3.1301 | 0.3301 | -0.3722 |
| C | 1.5591 | 1.5195 | -0.4905 | C | 2.3377 | 1.4568 | -0.6218 |
| C | 2.3748 | -0.8609 | -0.7915 | C | 2.6589 | -1.0575 | -0.6738 |
| C | 1.1144 | -1.304 | -1.502 | C | 1.3502 | -1.3092 | -1.39 |
| C | 0.085 | -1.8996 | -0.5433 | C | 0.1968 | -1.5806 | -0.4259 |
| C | 0.6017 | -3.1241 | 0.2374 | C | 0.4482 | -2.7578 | 0.5349 |
| C | -0.5574 | -3.7169 | 1.0495 | C | -0.8214 | -2.995 | 1.3625 |
| C | -1.768 | -2.7883 | 0.8987 | C | -1.8446 | -1.9118 | 0.9978 |
| C | -1.4151 | -1.3089 | 1.2415 | C | -1.2521 | -0.4824 | 1.1524 |
| C | -2.6242 | -0.3848 | 0.9399 | C | -2.1834 | 0.6263 | 0.6021 |
| C | -2.3781 | 1.1044 | 1.2142 | C | -3.5568 | 0.7077 | 1.2704 |
| C | -3.5594 | 1.9346 | 0.7881 | C | -4.3806 | 1.8182 | 0.6789 |
| C | -3.6676 | 2.6939 | -0.3197 | C | -5.3275 | 1.7108 | -0.2737 |
| C | -4.912 | 3.5013 | -0.5809 | C | -6.0905 | 2.9228 | -0.7406 |
| O | 0.7618 | 3.7216 | -0.2291 | O | 2.007 | 3.791 | -0.595 |
| O | -2.1369 | -2.852 | -0.5017 | O | -2.1635 | -2.1144 | -0.3995 |
| C | -1.1521 | -2.3817 | -1.3361 | C | -1.0782 | -1.9448 | -1.2247 |
| O | -1.2712 | -2.3678 | -2.5527 | O | -1.1479 | -2.0806 | -2.4376 |
| C | -0.9607 | -1.1623 | 2.6996 | C | -0.8564 | -0.1785 | 2.6024 |
| C | -2.6163 | 2.8129 | -1.3891 | C | -5.7069 | 0.4332 | -0.9719 |
| O | -0.3119 | -0.8866 | 0.4041 | O | -0.0334 | -0.398 | 0.3659 |
| O | 3.2785 | -1.6723 | -0.5675 | O | 3.3691 | -2.0069 | -0.3278 |
| O | 4.825 | 0.0788 | 0.4509 | O | 5.2651 | -0.5074 | 0.477 |
| H | 3.1563 | 4.2211 | 0.858 | H | 4.4726 | 3.9053 | 0.4373 |
| H | 4.9356 | 2.5261 | 1.1316 | H | 5.8789 | 1.9238 | 0.8941 |
| H | 0.5915 | 1.2876 | -0.9213 | H | 1.3343 | 1.3814 | -1.0275 |
| H | 0.6937 | -0.4744 | -2.0786 | H | 1.1222 | -0.4763 | -2.0628 |
| H | 1.4148 | -2.0449 | -2.2553 | H | 1.5116 | -2.1707 | -2.0521 |
| H | 1.0121 | -3.8858 | -0.4358 | H | 0.7147 | -3.6707 | -0.0105 |
| H | 1.3941 | -2.8316 | 0.936 | H | 1.2672 | -2.5286 | 1.2264 |
| H | -0.2707 | -3.8548 | 2.0966 | H | -0.5934 | -2.9957 | 2.4331 |
| H | -0.7997 | -4.7097 | 0.6503 | H | -1.2255 | -3.9849 | 1.1175 |
| H | -2.6156 | -3.1504 | 1.4896 | H | -2.7607 | -2.0462 | 1.5804 |
| H | -3.492 | -0.7223 | 1.5219 | H | -2.3106 | 0.4948 | -0.481 |
| H | -2.8973 | -0.4802 | -0.119 | H | -1.6713 | 1.5949 | 0.6966 |
| H | -1.4579 | 1.4415 | 0.7298 | H | -3.4474 | 0.9087 | 2.3422 |
| H | -2.2344 | 1.2743 | 2.2873 | H | -4.086 | -0.2462 | 1.1974 |
| H | -4.3971 | 1.9117 | 1.4851 | H | -4.1717 | 2.8037 | 1.0956 |
| H | -5.6504 | 3.3987 | 0.2213 | H | -5.7919 | 3.8324 | -0.2089 |
| H | -4.6619 | 4.5638 | -0.6679 | H | -5.9196 | 3.0909 | -1.809 |
| H | -5.3865 | 3.1772 | -1.5129 | H | -7.1637 | 2.78 | -0.5774 |
| H | 1.0339 | 4.5805 | 0.1353 | H | 2.4681 | 4.6113 | -0.3521 |
| H | -1.7595 | -1.4355 | 3.3962 | H | -1.6891 | -0.3371 | 3.2941 |
| H | -0.0846 | -1.7818 | 2.9155 | H | -0.0139 | -0.7982 | 2.9272 |
| H | -0.6367 | -0.1395 | 2.9179 | H | -0.5152 | 0.8585 | 2.7024 |
| H | -1.8024 | 2.0935 | -1.2768 | H | -5.056 | -0.4077 | -0.7205 |
| H | -3.06 | 2.6328 | -2.3743 | H | -6.7338 | 0.1544 | -0.7145 |
| H | -2.1858 | 3.8193 | -1.384 | H | -5.6474 | 0.5662 | -2.0575 |
| H | 4.5505 | -0.8094 | 0.1281 | H | 4.806 | -1.3513 | 0.2637 |

**Table S21.** Cartesian Coordinates of conformers **1−2c** and **1−2d** in the gas phase (Å) at the B3LYP/6-31(d,p) level.

| **1−2c** | | | | **1−2d** | | | |
| --- | --- | --- | --- | --- | --- | --- | --- |
| C | -2.2924 | 2.9095 | 0.5952 | C | 1.5568 | 3.0419 | -0.5225 |
| C | -3.4086 | 3.3994 | -0.0729 | C | 2.5669 | 3.6904 | 0.1778 |
| C | -4.3337 | 2.5002 | -0.5974 | C | 3.6414 | 2.9474 | 0.661 |
| C | -4.1352 | 1.1272 | -0.4461 | C | 3.6941 | 1.5706 | 0.4388 |
| C | -3.0114 | 0.6262 | 0.2362 | C | 2.6764 | 0.9099 | -0.273 |
| C | -2.0854 | 1.5398 | 0.7533 | C | 1.6016 | 1.6675 | -0.7522 |
| C | -2.8398 | -0.8525 | 0.3858 | C | 2.768 | -0.5676 | -0.491 |
| C | -1.7216 | -1.4377 | 1.2204 | C | 1.7268 | -1.3142 | -1.2962 |
| C | -0.4975 | -1.8061 | 0.3843 | C | 0.6235 | -1.9022 | -0.4181 |
| C | -0.7971 | -2.7856 | -0.766 | C | 1.1424 | -2.8599 | 0.6726 |
| C | 0.5247 | -3.1618 | -1.4474 | C | -0.0589 | -3.4922 | 1.3877 |
| C | 1.6508 | -2.3481 | -0.7977 | C | -1.3337 | -2.83 | 0.8522 |
| C | 1.3456 | -0.8241 | -0.814 | C | -1.2631 | -1.2745 | 0.9352 |
| C | 2.363 | 0.0089 | 0.005 | C | -2.5084 | -0.6444 | 0.258 |
| C | 3.8182 | -0.1218 | -0.4488 | C | -2.5155 | 0.8893 | 0.2199 |
| C | 4.7199 | 0.7598 | 0.3706 | C | -3.7026 | 1.4018 | -0.5511 |
| C | 5.2565 | 1.9432 | 0.0144 | C | -4.8535 | 1.8971 | -0.0557 |
| C | 6.1724 | 2.684 | 0.954 | C | -5.9311 | 2.403 | -0.9792 |
| O | -1.357 | 3.7518 | 1.1207 | O | 0.4819 | 3.7249 | -1.0104 |
| O | 1.7106 | -2.7757 | 0.5841 | O | -1.4093 | -3.2047 | -0.5463 |
| C | 0.5564 | -2.5033 | 1.2779 | C | -0.3564 | -2.7247 | -1.2866 |
| O | 0.4143 | -2.7954 | 2.4564 | O | -0.2393 | -2.9444 | -2.4835 |
| C | 1.231 | -0.2759 | -2.2419 | C | -1.1254 | -0.7951 | 2.3863 |
| C | 5.0091 | 2.6477 | -1.2909 | C | -5.1962 | 1.9908 | 1.4054 |
| O | 0.0556 | -0.6018 | -0.1835 | O | -0.0885 | -0.8221 | 0.2203 |
| O | -3.6409 | -1.5975 | -0.1874 | O | 3.7187 | -1.1771 | 0.0089 |
| O | -5.0995 | 0.3264 | -1.001 | O | 4.7895 | 0.9303 | 0.9576 |
| H | -3.5735 | 4.4649 | -0.1973 | H | 2.5364 | 4.7603 | 0.3576 |
| H | -5.2116 | 2.8629 | -1.1261 | H | 4.441 | 3.4355 | 1.2126 |
| H | -1.1858 | 1.2235 | 1.2708 | H | 0.7745 | 1.2223 | -1.2945 |
| H | -1.4601 | -0.7534 | 2.0337 | H | 1.317 | -0.6667 | -2.0775 |
| H | -2.1305 | -2.3265 | 1.7197 | H | 2.254 | -2.1106 | -1.8388 |
| H | -1.3003 | -3.6891 | -0.4024 | H | 1.7782 | -3.6455 | 0.2477 |
| H | -1.4458 | -2.3188 | -1.5162 | H | 1.7346 | -2.3146 | 1.4166 |
| H | 0.4685 | -2.9895 | -2.527 | H | 0.0346 | -3.3907 | 2.4733 |
| H | 0.7081 | -4.2332 | -1.3002 | H | -0.0785 | -4.5667 | 1.167 |
| H | 2.6094 | -2.5832 | -1.2701 | H | -2.2218 | -3.2233 | 1.3575 |
| H | 2.2872 | -0.2558 | 1.0684 | H | -3.4136 | -0.9994 | 0.7683 |
| H | 2.0595 | 1.0652 | -0.0315 | H | -2.5746 | -0.9952 | -0.78 |
| H | 3.9161 | 0.0968 | -1.5152 | H | -1.6098 | 1.2604 | -0.273 |
| H | 4.1629 | -1.1543 | -0.3206 | H | -2.5026 | 1.3077 | 1.2293 |
| H | 4.9572 | 0.3588 | 1.3568 | H | -3.5741 | 1.3696 | -1.6336 |
| H | 6.3355 | 2.1408 | 1.8908 | H | -5.6454 | 2.3264 | -2.0336 |
| H | 7.1499 | 2.8387 | 0.4855 | H | -6.8511 | 1.8256 | -0.8413 |
| H | 5.7479 | 3.6613 | 1.2061 | H | -6.1455 | 3.456 | -0.769 |
| H | -1.6228 | 4.669 | 0.9405 | H | 0.5697 | 4.6631 | -0.7724 |
| H | 2.1151 | -0.5104 | -2.8421 | H | -2.0001 | -1.0719 | 2.983 |
| H | 0.3494 | -0.6743 | -2.7553 | H | -0.2327 | -1.2077 | 2.8666 |
| H | 1.0986 | 0.8122 | -2.2308 | H | -0.9906 | 0.29 | 2.4401 |
| H | 4.2397 | 2.1704 | -1.9023 | H | -4.4672 | 1.4992 | 2.0538 |
| H | 4.6746 | 3.6744 | -1.1064 | H | -5.2655 | 3.0403 | 1.7092 |
| H | 5.932 | 2.6866 | -1.8785 | H | -6.1628 | 1.5122 | 1.5961 |
| H | -4.8323 | -0.6098 | -0.8577 | H | 4.7057 | -0.0275 | 0.7478 |

**Table S22.** Cartesian Coordinates of conformers **1−2e** and **1−2f** in the gas phase (Å) at the B3LYP/6-31(d,p) level.

| **1−2e** | | | | **1−2f** | | | |
| --- | --- | --- | --- | --- | --- | --- | --- |
| C | 1.93 | 2.7208 | 0.0657 | C | -3.2793 | 2.272 | 0.9687 |
| C | 3.1957 | 3.0142 | 0.5567 | C | -4.3134 | 2.6668 | 0.1279 |
| C | 4.165 | 2.0158 | 0.5956 | C | -4.7937 | 1.7703 | -0.8235 |
| C | 3.8589 | 0.7331 | 0.1419 | C | -4.2392 | 0.4934 | -0.9215 |
| C | 2.5831 | 0.4296 | -0.3678 | C | -3.1977 | 0.0859 | -0.0679 |
| C | 1.6168 | 1.4453 | -0.4031 | C | -2.7196 | 0.998 | 0.8804 |
| C | 2.293 | -0.9598 | -0.8451 | C | -2.6364 | -1.2948 | -0.1982 |
| C | 0.9787 | -1.3076 | -1.5099 | C | -1.6061 | -1.8239 | 0.7752 |
| C | -0.0509 | -1.8554 | -0.5227 | C | -0.1757 | -1.6452 | 0.2696 |
| C | 0.4207 | -3.1093 | 0.2382 | C | 0.0878 | -2.2939 | -1.102 |
| C | -0.7427 | -3.6396 | 1.0869 | C | 1.5769 | -2.1447 | -1.4377 |
| C | -1.9072 | -2.6492 | 0.972 | C | 2.2406 | -1.3172 | -0.33 |
| C | -1.4673 | -1.1913 | 1.3058 | C | 1.5121 | 0.0363 | -0.1044 |
| C | -2.6285 | -0.1983 | 1.0365 | C | 2.0201 | 0.8084 | 1.1423 |
| C | -2.2606 | 1.2765 | 1.2442 | C | 3.5339 | 1.0044 | 1.293 |
| C | -3.36 | 2.1931 | 0.7828 | C | 4.1387 | 1.9099 | 0.2593 |
| C | -3.4924 | 2.7499 | -0.4372 | C | 5.1748 | 1.6612 | -0.5658 |
| C | -4.6371 | 3.6825 | -0.7371 | C | 5.6377 | 2.7104 | -1.5451 |
| O | 1.0066 | 3.7247 | 0.0648 | O | -2.7728 | 3.1177 | 1.9115 |
| O | -2.3177 | -2.6891 | -0.418 | O | 2.1201 | -2.097 | 0.8842 |
| C | -1.3373 | -2.2602 | -1.2788 | C | 0.8148 | -2.3077 | 1.2563 |
| O | -1.4989 | -2.2098 | -2.4894 | O | 0.5113 | -2.9479 | 2.2526 |
| C | -0.9677 | -1.069 | 2.751 | C | 1.5295 | 0.9151 | -1.3612 |
| C | -2.5744 | 2.5005 | -1.602 | C | 5.9704 | 0.386 | -0.617 |
| O | -0.3657 | -0.8261 | 0.4385 | O | 0.1057 | -0.235 | 0.1646 |
| O | 3.1547 | -1.8294 | -0.6855 | O | -3.0382 | -2.007 | -1.1238 |
| O | 4.8735 | -0.1841 | 0.23 | O | -4.7782 | -0.3116 | -1.8912 |
| H | 3.4254 | 4.0142 | 0.9141 | H | -4.7522 | 3.6573 | 0.194 |
| H | 5.1572 | 2.2325 | 0.9825 | H | -5.5996 | 2.0608 | -1.4927 |
| H | 0.6175 | 1.2585 | -0.7755 | H | -1.9049 | 0.7585 | 1.5555 |
| H | 0.5922 | -0.4397 | -2.0539 | H | -1.7443 | -1.3625 | 1.7582 |
| H | 1.201 | -2.051 | -2.2875 | H | -1.8316 | -2.888 | 0.9287 |
| H | 0.7655 | -3.89 | -0.4499 | H | -0.191 | -3.3541 | -1.1042 |
| H | 1.2508 | -2.8644 | 0.9109 | H | -0.4902 | -1.7934 | -1.8874 |
| H | -0.4315 | -3.7914 | 2.125 | H | 1.7094 | -1.686 | -2.4228 |
| H | -1.0489 | -4.6185 | 0.6975 | H | 2.0333 | -3.1412 | -1.4808 |
| H | -2.756 | -2.9672 | 1.586 | H | 3.3041 | -1.184 | -0.5462 |
| H | -3.4852 | -0.4548 | 1.6732 | H | 1.667 | 0.2913 | 2.0454 |
| H | -2.9651 | -0.3097 | -0.002 | H | 1.5176 | 1.7852 | 1.1873 |
| H | -1.3241 | 1.5239 | 0.7369 | H | 4.0264 | 0.031 | 1.3424 |
| H | -2.0872 | 1.4781 | 2.3075 | H | 3.7131 | 1.4718 | 2.2702 |
| H | -4.1043 | 2.4241 | 1.5452 | H | 3.6671 | 2.8925 | 0.2058 |
| H | -5.2823 | 3.8421 | 0.1332 | H | 5.0389 | 3.6256 | -1.4896 |
| H | -4.256 | 4.6596 | -1.0516 | H | 6.6795 | 2.9822 | -1.3458 |
| H | -5.2582 | 3.275 | -1.5415 | H | 5.5662 | 2.3293 | -2.5691 |
| H | 0.1424 | 3.3736 | -0.2076 | H | -3.2524 | 3.9618 | 1.8663 |
| H | -1.7583 | -1.3102 | 3.4684 | H | 2.5325 | 1.0176 | -1.7818 |
| H | -0.1132 | -1.726 | 2.9409 | H | 0.8775 | 0.5071 | -2.1418 |
| H | -0.5962 | -0.061 | 2.963 | H | 1.1369 | 1.9153 | -1.1453 |
| H | -1.83 | 1.7253 | -1.4047 | H | 5.6744 | -0.3466 | 0.1357 |
| H | -3.1515 | 2.1728 | -2.4733 | H | 5.8584 | -0.0859 | -1.5988 |
| H | -2.0421 | 3.42 | -1.8658 | H | 7.0327 | 0.5996 | -0.4569 |
| H | 4.5278 | -1.0501 | -0.0834 | H | -4.2864 | -1.1639 | -1.8824 |

**Table S23.** Cartesian Coordinates of conformers **1−2g** and **1−2h** in the gas phase (Å) at the B3LYP/6-31(d,p) level.

| **1−2g** | | | | **1−2h** | | | |
| --- | --- | --- | --- | --- | --- | --- | --- |
| C | -3.5182 | 2.2034 | 0.7326 | C | 4.9504 | 1.7336 | 1.1178 |
| C | -4.6407 | 2.3551 | -0.0732 | C | 5.909 | 1.6916 | 0.1114 |
| C | -5.0781 | 1.2757 | -0.8369 | C | 5.7037 | 0.8611 | -0.9886 |
| C | -4.3932 | 0.0609 | -0.7846 | C | 4.546 | 0.0872 | -1.0721 |
| C | -3.2613 | -0.1005 | 0.0351 | C | 3.5705 | 0.1357 | -0.0615 |
| C | -2.828 | 0.9935 | 0.7936 | C | 3.7891 | 0.9665 | 1.0442 |
| C | -2.5599 | -1.4216 | 0.0704 | C | 2.3381 | -0.7015 | -0.1742 |
| C | -1.4164 | -1.6836 | 1.0259 | C | 1.2041 | -0.4905 | 0.8125 |
| C | -0.0502 | -1.4428 | 0.3861 | C | -0.132 | -1.1177 | 0.4087 |
| C | 0.1977 | -2.2722 | -0.8865 | C | -0.1239 | -2.6565 | 0.3414 |
| C | 1.634 | -2.0217 | -1.36 | C | -1.5103 | -3.1366 | -0.1042 |
| C | 2.2694 | -0.9653 | -0.445 | C | -2.4337 | -1.9185 | -0.2069 |
| C | 1.4082 | 0.3272 | -0.367 | C | -2.446 | -1.0988 | 1.1141 |
| C | 1.8792 | 1.3453 | 0.7056 | C | -3.197 | 0.2509 | 0.9906 |
| C | 3.182 | 2.1103 | 0.4411 | C | -4.6662 | 0.1374 | 0.5803 |
| C | 4.4183 | 1.2655 | 0.5464 | C | -5.3146 | 1.4925 | 0.5072 |
| C | 5.4 | 1.1044 | -0.3627 | C | -5.4931 | 2.2497 | -0.593 |
| C | 6.5735 | 0.2056 | -0.0638 | C | -6.1926 | 3.5808 | -0.5067 |
| O | -3.0494 | 3.2342 | 1.4931 | O | 5.1117 | 2.5253 | 2.2165 |
| O | 2.3155 | -1.556 | 0.8763 | O | -1.8881 | -1.0785 | -1.2522 |
| C | 1.0689 | -1.8335 | 1.3802 | C | -0.6516 | -0.5655 | -0.9396 |
| O | 0.9023 | -2.3319 | 2.4841 | O | -0.0888 | 0.2627 | -1.6412 |
| C | 1.2676 | 1.0069 | -1.7342 | C | -3.0053 | -1.9134 | 2.2874 |
| C | 5.4601 | 1.7579 | -1.7156 | C | -5.0181 | 1.8875 | -1.9741 |
| O | 0.0602 | -0.0485 | 0.0398 | O | -1.0746 | -0.7625 | 1.4618 |
| O | -2.9391 | -2.3084 | -0.7011 | O | 2.2985 | -1.5596 | -1.0564 |
| O | -4.9001 | -0.9372 | -1.5756 | O | 4.4378 | -0.7035 | -2.1856 |
| H | -5.1811 | 3.295 | -0.123 | H | 6.8143 | 2.288 | 0.1644 |
| H | -5.9519 | 1.3748 | -1.4759 | H | 6.4431 | 0.8117 | -1.7839 |
| H | -1.951 | 0.9461 | 1.4304 | H | 3.0816 | 1.0351 | 1.8643 |
| H | -1.5428 | -1.0891 | 1.9364 | H | 1.5164 | -0.8872 | 1.7864 |
| H | -1.5104 | -2.7273 | 1.3551 | H | 1.0445 | 0.5884 | 0.9352 |
| H | 0.0453 | -3.3424 | -0.704 | H | 0.6218 | -3.0546 | -0.3512 |
| H | -0.4835 | -1.9683 | -1.6895 | H | 0.0956 | -3.0742 | 1.3318 |
| H | 1.6497 | -1.7128 | -2.4101 | H | -1.9013 | -3.8902 | 0.5868 |
| H | 2.1992 | -2.9594 | -1.2913 | H | -1.4217 | -3.6152 | -1.0873 |
| H | 3.2921 | -0.7713 | -0.772 | H | -3.438 | -2.2293 | -0.51 |
| H | 1.933 | 0.8586 | 1.6882 | H | -2.6693 | 0.9031 | 0.2819 |
| H | 1.0823 | 2.0941 | 0.8271 | H | -3.1239 | 0.7834 | 1.9499 |
| H | 3.2681 | 2.8928 | 1.2065 | H | -5.2218 | -0.4586 | 1.3134 |
| H | 3.1119 | 2.6419 | -0.51 | H | -4.7682 | -0.3896 | -0.3724 |
| H | 4.512 | 0.7397 | 1.4987 | H | -5.678 | 1.8698 | 1.4631 |
| H | 6.5018 | -0.259 | 0.9252 | H | -6.5271 | 3.8095 | 0.5106 |
| H | 6.6317 | -0.5988 | -0.8044 | H | -5.5203 | 4.3845 | -0.8243 |
| H | 7.5067 | 0.7774 | -0.0955 | H | -7.0746 | 3.5872 | -1.1555 |
| H | -3.6231 | 4.0076 | 1.3616 | H | 5.9454 | 3.0175 | 2.1285 |
| H | 2.241 | 1.2068 | -2.1915 | H | -3.9964 | -2.3202 | 2.0656 |
| H | 0.6786 | 0.3977 | -2.428 | H | -2.3422 | -2.7435 | 2.5529 |
| H | 0.7254 | 1.9553 | -1.6419 | H | -3.0788 | -1.295 | 3.1896 |
| H | 4.6248 | 2.4311 | -1.9155 | H | -4.4009 | 0.9862 | -1.9975 |
| H | 6.3792 | 2.3462 | -1.8078 | H | -5.8749 | 1.7275 | -2.6366 |
| H | 5.46 | 0.9941 | -2.5004 | H | -4.4096 | 2.6992 | -2.3871 |
| H | -4.3181 | -1.7246 | -1.4765 | H | 3.6018 | -1.217 | -2.1101 |

**Table S24.** Cartesian Coordinates of conformers **1−2i** and **1−2j** in the gas phase (Å) at the B3LYP/6-31(d,p) level.

| **1−2i** | | | | **1−2j** | | | |
| --- | --- | --- | --- | --- | --- | --- | --- |
| C | 4.8982 | 2.2516 | 0.6104 | C | 2.7676 | 2.7377 | -0.3306 |
| C | 5.9858 | 1.8633 | -0.1641 | C | 4.0321 | 2.9378 | 0.2084 |
| C | 5.9367 | 0.648 | -0.8441 | C | 4.8501 | 1.841 | 0.4637 |
| C | 4.8046 | -0.1612 | -0.7457 | C | 4.3955 | 0.5546 | 0.1757 |
| C | 3.6996 | 0.2336 | 0.0278 | C | 3.1193 | 0.3431 | -0.3786 |
| C | 3.7612 | 1.4524 | 0.7145 | C | 2.3036 | 1.4564 | -0.6284 |
| C | 2.4978 | -0.6497 | 0.1165 | C | 2.6709 | -1.0538 | -0.6773 |
| C | 1.2394 | -0.1266 | 0.7849 | C | 1.3647 | -1.3278 | -1.3903 |
| C | -0.0253 | -0.9451 | 0.5163 | C | 0.2124 | -1.5913 | -0.4222 |
| C | 0.0027 | -2.3748 | 1.0888 | C | 0.4724 | -2.7534 | 0.554 |
| C | -1.306 | -3.0808 | 0.7147 | C | -0.7958 | -2.9931 | 1.3821 |
| C | -2.2168 | -2.0692 | 0.012 | C | -1.8249 | -1.9193 | 1.009 |
| C | -2.4137 | -0.7874 | 0.8686 | C | -1.2398 | -0.4852 | 1.1492 |
| C | -3.1575 | 0.3435 | 0.1142 | C | -2.1824 | 0.6141 | 0.5997 |
| C | -4.5469 | -0.0327 | -0.4045 | C | -3.5537 | 0.6877 | 1.2728 |
| C | -5.2097 | 1.1413 | -1.0711 | C | -4.3815 | 1.7999 | 0.6902 |
| C | -6.1876 | 1.9244 | -0.5754 | C | -5.3122 | 1.6984 | -0.2788 |
| C | -6.7635 | 3.0463 | -1.4002 | C | -6.0859 | 2.9084 | -0.7334 |
| O | 4.904 | 3.4284 | 1.2996 | O | 1.9968 | 3.8408 | -0.5538 |
| O | -1.5418 | -1.6914 | -1.2119 | O | -2.1447 | -2.1355 | -0.3863 |
| C | -0.3625 | -1.0202 | -0.9911 | C | -1.0623 | -1.9667 | -1.2149 |
| O | 0.282 | -0.5119 | -1.8974 | O | -1.1355 | -2.0987 | -2.428 |
| C | -3.1149 | -1.0902 | 2.1994 | C | -0.8329 | -0.1683 | 2.5933 |
| C | -6.7867 | 1.7968 | 0.7979 | C | -5.6647 | 0.4288 | -1.0055 |
| O | -1.1049 | -0.2508 | 1.2062 | O | -0.0292 | -0.3955 | 0.3488 |
| O | 2.5821 | -1.7913 | -0.3373 | O | 3.3947 | -1.9918 | -0.3299 |
| O | 4.8533 | -1.3398 | -1.4425 | O | 5.2686 | -0.4608 | 0.4679 |
| H | 6.8729 | 2.4831 | -0.2485 | H | 4.3784 | 3.9429 | 0.4327 |
| H | 6.7783 | 0.3253 | -1.4518 | H | 5.84 | 1.9848 | 0.8886 |
| H | 2.9476 | 1.8047 | 1.3405 | H | 1.306 | 1.345 | -1.0358 |
| H | 1.4195 | -0.0739 | 1.8657 | H | 1.1326 | -0.5081 | -2.0779 |
| H | 1.0575 | 0.8971 | 0.4337 | H | 1.5323 | -2.1996 | -2.0373 |
| H | 0.8391 | -2.9723 | 0.718 | H | 0.7487 | -3.6705 | 0.0208 |
| H | 0.0873 | -2.3392 | 2.182 | H | 1.2882 | -2.5073 | 1.2434 |
| H | -1.7811 | -3.5137 | 1.6007 | H | -0.5685 | -2.9851 | 2.4529 |
| H | -1.0804 | -3.9092 | 0.0318 | H | -1.1935 | -3.9872 | 1.1437 |
| H | -3.1691 | -2.5371 | -0.2553 | H | -2.7394 | -2.0532 | 1.5942 |
| H | -2.5411 | 0.7007 | -0.7216 | H | -2.3132 | 0.4774 | -0.4823 |
| H | -3.2368 | 1.2145 | 0.7807 | H | -1.6773 | 1.5869 | 0.6881 |
| H | -5.169 | -0.4327 | 0.4002 | H | -3.4423 | 0.8816 | 2.3458 |
| H | -4.4681 | -0.8325 | -1.1498 | H | -4.0816 | -0.2668 | 1.1943 |
| H | -4.8411 | 1.3463 | -2.0771 | H | -4.1942 | 2.7793 | 1.1309 |
| H | -6.3101 | 3.1067 | -2.3952 | H | -5.8073 | 3.8118 | -0.1807 |
| H | -7.8406 | 2.9024 | -1.5346 | H | -5.9029 | 3.099 | -1.796 |
| H | -6.5996 | 4.0071 | -0.9014 | H | -7.1591 | 2.7477 | -0.5877 |
| H | 5.74 | 3.8917 | 1.1218 | H | 1.1366 | 3.5659 | -0.912 |
| H | -4.0609 | -1.6195 | 2.0518 | H | -1.6581 | -0.3277 | 3.2937 |
| H | -2.4816 | -1.6924 | 2.8594 | H | 0.0169 | -0.7797 | 2.9147 |
| H | -3.3187 | -0.1648 | 2.7507 | H | -0.498 | 0.8717 | 2.6829 |
| H | -6.2686 | 1.0733 | 1.4316 | H | -5.0097 | -0.4093 | -0.7553 |
| H | -6.7439 | 2.7601 | 1.3177 | H | -6.6931 | 0.135 | -0.7723 |
| H | -7.8355 | 1.4916 | 0.7241 | H | -5.5861 | 0.5809 | -2.0874 |
| H | 4.0167 | -1.8276 | -1.2679 | H | 4.8247 | -1.3128 | 0.2568 |

**Table S25.** Cartesian Coordinates of conformers **1−2k** and **1−2l** in the gas phase (Å) at the B3LYP/6-31(d,p) level.

| **1−2k** | | | | **1−2l** | | | |
| --- | --- | --- | --- | --- | --- | --- | --- |
| C | -2.2242 | 2.9125 | 0.5782 | C | 5.3994 | 1.7302 | -0.0552 |
| C | -3.3431 | 3.415 | -0.0736 | C | 6.4013 | 0.8387 | -0.4226 |
| C | -4.2976 | 2.5347 | -0.5751 | C | 6.103 | -0.5195 | -0.5128 |
| C | -4.1239 | 1.1601 | -0.4182 | C | 4.8113 | -0.9698 | -0.2392 |
| C | -2.9982 | 0.6425 | 0.2493 | C | 3.7941 | -0.0698 | 0.1221 |
| C | -2.0426 | 1.5399 | 0.7479 | C | 4.105 | 1.2922 | 0.2186 |
| C | -2.8517 | -0.8399 | 0.4004 | C | 2.418 | -0.576 | 0.409 |
| C | -1.7338 | -1.4429 | 1.223 | C | 1.2863 | 0.4173 | 0.6003 |
| C | -0.5149 | -1.809 | 0.3777 | C | -0.1202 | -0.182 | 0.5352 |
| C | -0.8279 | -2.7643 | -0.7887 | C | -0.4464 | -1.1739 | 1.6673 |
| C | 0.4872 | -3.1468 | -1.4785 | C | -1.8648 | -1.7152 | 1.4545 |
| C | 1.6237 | -2.3544 | -0.8217 | C | -2.4874 | -0.983 | 0.2619 |
| C | 1.3336 | -0.8271 | -0.8146 | C | -2.4258 | 0.5587 | 0.4426 |
| C | 2.3668 | -0.0157 | 0.0057 | C | -2.8442 | 1.3472 | -0.8274 |
| C | 3.8147 | -0.1422 | -0.4709 | C | -4.1598 | 0.9528 | -1.511 |
| C | 4.7293 | 0.7188 | 0.3562 | C | -5.3858 | 1.2575 | -0.6992 |
| C | 5.2407 | 1.9215 | 0.0289 | C | -6.3812 | 0.4214 | -0.3436 |
| C | 6.1773 | 2.6385 | 0.9665 | C | -7.5387 | 0.9227 | 0.4826 |
| O | -1.3093 | 3.8117 | 1.042 | O | 5.6462 | 3.0671 | 0.0536 |
| O | 1.6842 | -2.8018 | 0.554 | O | -1.6911 | -1.3324 | -0.8963 |
| C | 0.5366 | -2.5265 | 1.2569 | C | -0.4005 | -0.8661 | -0.8225 |
| O | 0.4012 | -2.8198 | 2.4358 | O | 0.3811 | -0.9496 | -1.7593 |
| C | 1.209 | -0.2575 | -2.2332 | C | -3.2095 | 1.0224 | 1.6774 |
| C | 4.9458 | 2.6698 | -1.242 | C | -6.4761 | -1.0352 | -0.7057 |
| O | 0.0534 | -0.5985 | -0.1646 | O | -1.0394 | 0.9378 | 0.693 |
| O | -3.67 | -1.573 | -0.1628 | O | 2.2561 | -1.7927 | 0.5066 |
| O | -5.1118 | 0.3741 | -0.9519 | O | 4.6172 | -2.3217 | -0.3463 |
| H | -3.47 | 4.4872 | -0.195 | H | 7.4104 | 1.1758 | -0.6374 |
| H | -5.1751 | 2.9159 | -1.0906 | H | 6.8735 | -1.2325 | -0.7952 |
| H | -1.1498 | 1.1909 | 1.2528 | H | 3.3678 | 2.034 | 0.5086 |
| H | -1.4663 | -0.7699 | 2.044 | H | 1.4268 | 0.9186 | 1.5659 |
| H | -2.1463 | -2.3356 | 1.7125 | H | 1.3606 | 1.1852 | -0.1803 |
| H | -1.3427 | -3.6672 | -0.4401 | H | 0.2455 | -2.0185 | 1.7138 |
| H | -1.4717 | -2.2759 | -1.5294 | H | -0.4023 | -0.6634 | 2.6373 |
| H | 0.4301 | -2.9603 | -2.5557 | H | -2.4645 | -1.6014 | 2.3632 |
| H | 0.659 | -4.222 | -1.3454 | H | -1.8068 | -2.789 | 1.2377 |
| H | 2.5781 | -2.5922 | -1.301 | H | -3.5075 | -1.3383 | 0.0924 |
| H | 2.3049 | -0.3014 | 1.0646 | H | -2.0475 | 1.2576 | -1.5786 |
| H | 2.0672 | 1.0424 | -0.0051 | H | -2.8611 | 2.4209 | -0.5924 |
| H | 3.9003 | 0.1012 | -1.5332 | H | -4.1105 | -0.0914 | -1.8265 |
| H | 4.1574 | -1.1785 | -0.3712 | H | -4.2397 | 1.5343 | -2.4388 |
| H | 5.0023 | 0.2832 | 1.3182 | H | -5.4583 | 2.3006 | -0.3872 |
| H | 6.3753 | 2.0634 | 1.8772 | H | -7.442 | 1.9838 | 0.7353 |
| H | 7.138 | 2.8219 | 0.4742 | H | -8.4786 | 0.7957 | -0.0646 |
| H | 5.7503 | 3.6008 | 1.2673 | H | -7.6032 | 0.363 | 1.4215 |
| H | -0.5641 | 3.3354 | 1.4441 | H | 6.5742 | 3.2347 | -0.1829 |
| H | 2.0833 | -0.4939 | -2.8468 | H | -4.2222 | 0.6136 | 1.7032 |
| H | 0.3168 | -0.638 | -2.7417 | H | -2.7043 | 0.7281 | 2.6041 |
| H | 1.0899 | 0.8319 | -2.2051 | H | -3.2732 | 2.116 | 1.7132 |
| H | 4.1652 | 2.2039 | -1.8478 | H | -5.6704 | -1.3792 | -1.3565 |
| H | 4.6034 | 3.6841 | -1.0101 | H | -6.4525 | -1.6486 | 0.2011 |
| H | 5.8505 | 2.7431 | -1.8542 | H | -7.4178 | -1.2313 | -1.2295 |
| H | -4.8597 | -0.5665 | -0.8125 | H | 3.6837 | -2.5124 | -0.1006 |

**Table S26.** Cartesian Coordinates of conformers **1−3a** and **1−3b** in the gas phase (Å) at the B3LYP/6-31(d,p) level.

| **1−3a** | | | | **1−3b** | | | |
| --- | --- | --- | --- | --- | --- | --- | --- |
| C | -5.4365 | -0.1353 | 0.2021 | C | 5.827 | 0.933 | -0.493 |
| C | -5.6748 | 1.14 | -0.2981 | C | 6.0242 | 1.8464 | 0.5364 |
| C | -4.5999 | 1.9089 | -0.7402 | C | 4.9583 | 2.1651 | 1.3754 |
| C | -3.3041 | 1.3967 | -0.6779 | C | 3.7124 | 1.5693 | 1.178 |
| C | -3.0603 | 0.1096 | -0.1684 | C | 3.5108 | 0.6439 | 0.1396 |
| C | -4.1459 | -0.6568 | 0.2714 | C | 4.587 | 0.3313 | -0.6998 |
| C | -1.6627 | -0.4113 | -0.0993 | C | 2.1674 | 0.0197 | -0.0543 |
| C | -1.3951 | -1.7439 | 0.5734 | C | 1.9751 | -1.0089 | -1.1533 |
| C | 0.1018 | -2.0802 | 0.6891 | C | 0.5622 | -1.6175 | -1.1815 |
| C | 0.8215 | -1.1651 | 1.6796 | C | -0.4808 | -0.6234 | -1.6889 |
| C | 2.3058 | -1.0752 | 1.3659 | C | -1.8716 | -1.0001 | -1.2068 |
| C | 2.4513 | -0.57 | -0.0664 | C | -1.8527 | -1.0228 | 0.3188 |
| C | 3.9247 | -0.3292 | -0.5071 | C | -3.2346 | -1.3546 | 0.9594 |
| C | 4.723 | 0.6618 | 0.3735 | C | -4.3764 | -0.4132 | 0.517 |
| C | 4.2909 | 2.1346 | 0.364 | C | -4.1537 | 1.0682 | 0.8308 |
| C | 3.0382 | 2.4149 | 1.1458 | C | -5.3041 | 1.9024 | 0.3374 |
| C | 1.8864 | 2.9636 | 0.7113 | C | -5.33 | 2.7247 | -0.7298 |
| C | 0.7315 | 3.1704 | 1.6582 | C | -6.5731 | 3.5101 | -1.0606 |
| O | -6.4562 | -0.9248 | 0.6448 | O | 6.8388 | 0.5912 | -1.3407 |
| O | 1.8855 | -1.5547 | -0.9723 | O | -0.9159 | -2.0293 | 0.7834 |
| C | 0.7354 | -2.2058 | -0.7058 | C | 0.2479 | -2.2971 | 0.1597 |
| O | 0.2466 | -2.9735 | -1.5372 | O | 1.0182 | -3.1445 | 0.6156 |
| C | 3.9898 | 0.0865 | -1.9865 | C | -3.1291 | -1.3925 | 2.493 |
| C | 1.606 | 3.4125 | -0.6969 | C | -4.1909 | 2.9441 | -1.6866 |
| O | -0.7589 | 0.2693 | -0.5863 | O | 1.244 | 0.3673 | 0.6839 |
| O | -2.3135 | 2.2247 | -1.1363 | O | 2.7279 | 1.9478 | 2.052 |
| O | 0.1739 | -3.4142 | 1.2563 | O | 0.5983 | -2.6906 | -2.1558 |
| O | 4.625 | -1.5881 | -0.4099 | O | -3.6207 | -2.6783 | 0.5429 |
| H | -6.6788 | 1.5494 | -0.3531 | H | 6.9886 | 2.3169 | 0.7008 |
| H | -4.7673 | 2.9074 | -1.1364 | H | 5.0934 | 2.8775 | 2.1856 |
| H | -4.0242 | -1.66 | 0.6676 | H | 4.4963 | -0.3725 | -1.521 |
| H | -1.8349 | -1.7357 | 1.5784 | H | 2.1873 | -0.5422 | -2.123 |
| H | -1.9055 | -2.526 | -0.0027 | H | 2.7101 | -1.8104 | -1.0058 |
| H | 0.6959 | -1.5321 | 2.7061 | H | -0.481 | -0.592 | -2.7858 |
| H | 0.4065 | -0.1508 | 1.6625 | H | -0.2635 | 0.396 | -1.3501 |
| H | 2.777 | -0.4025 | 2.087 | H | -2.5869 | -0.2654 | -1.5879 |
| H | 2.7639 | -2.0648 | 1.4806 | H | -2.1471 | -1.9815 | -1.6105 |
| H | 1.8786 | 0.3474 | -0.2204 | H | -1.5003 | -0.0616 | 0.7112 |
| H | 5.7706 | 0.6303 | 0.0389 | H | -5.3111 | -0.7477 | 0.9905 |
| H | 4.7718 | 0.292 | 1.4057 | H | -4.5622 | -0.5442 | -0.5574 |
| H | 4.242 | 2.5056 | -0.6616 | H | -3.2071 | 1.4168 | 0.4118 |
| H | 5.091 | 2.7165 | 0.8409 | H | -4.0768 | 1.2171 | 1.914 |
| H | 3.1124 | 2.1507 | 2.2018 | H | -6.2052 | 1.8168 | 0.9458 |
| H | 0.9577 | 2.8245 | 2.6722 | H | -7.3811 | 3.3328 | -0.343 |
| H | 0.4756 | 4.2333 | 1.718 | H | -6.3548 | 4.5831 | -1.056 |
| H | -0.1478 | 2.619 | 1.3091 | H | -6.9436 | 3.2339 | -2.0531 |
| H | -7.2969 | -0.4509 | 0.5259 | H | 7.6447 | 1.0677 | -1.078 |
| H | 5.0244 | 0.2871 | -2.288 | H | -4.1059 | -1.6027 | 2.9438 |
| H | 3.6367 | -0.7151 | -2.6451 | H | -2.4646 | -2.1964 | 2.8294 |
| H | 3.3904 | 0.9795 | -2.1863 | H | -2.7553 | -0.4481 | 2.8999 |
| H | 2.4463 | 3.2697 | -1.3782 | H | -3.3382 | 2.2859 | -1.5071 |
| H | 0.7575 | 2.855 | -1.1063 | H | -4.5227 | 2.7597 | -2.7141 |
| H | 1.3552 | 4.4784 | -0.708 | H | -3.8372 | 3.978 | -1.6194 |
| H | -1.4655 | 1.7282 | -1.1026 | H | 1.9074 | 1.4611 | 1.8155 |
| H | -0.1195 | -4.0351 | 0.559 | H | 1.1265 | -3.4148 | -1.7631 |
| H | 4.1108 | -2.2321 | -0.9336 | H | -2.8853 | -3.2729 | 0.7849 |

**Table S27.** Cartesian Coordinates of conformers **1−3c** and **1−3d** in the gas phase (Å) at the B3LYP/6-31(d,p) level.

| **1−3c** | | | | **1−3d** | | | |
| --- | --- | --- | --- | --- | --- | --- | --- |
| C | 5.5762 | 0.2311 | -0.2045 | C | 5.9936 | 0.999 | -0.4631 |
| C | 5.6945 | 1.5729 | 0.1398 | C | 6.0565 | 2.2062 | 0.2235 |
| C | 4.5429 | 2.309 | 0.4114 | C | 4.8976 | 2.72 | 0.8021 |
| C | 3.2909 | 1.6982 | 0.3374 | C | 3.6937 | 2.0241 | 0.6895 |
| C | 3.1686 | 0.3415 | -0.0095 | C | 3.6278 | 0.8023 | -0.002 |
| C | 4.3308 | -0.3898 | -0.2821 | C | 4.7967 | 0.2948 | -0.582 |
| C | 1.817 | -0.2895 | -0.0878 | C | 2.3272 | 0.0753 | -0.1114 |
| C | 1.699 | -1.7602 | -0.4421 | C | 2.2799 | -1.2645 | -0.8226 |
| C | 0.2495 | -2.277 | -0.4363 | C | 0.8921 | -1.9281 | -0.7856 |
| C | -0.559 | -1.737 | -1.6161 | C | -0.1091 | -1.2153 | -1.6929 |
| C | -2.0467 | -1.7343 | -1.3051 | C | -1.5365 | -1.4931 | -1.2523 |
| C | -2.2596 | -0.8878 | -0.0532 | C | -1.6875 | -1.0146 | 0.1888 |
| C | -3.7556 | -0.709 | 0.3371 | C | -3.1245 | -1.199 | 0.763 |
| C | -4.6254 | -0.0714 | -0.773 | C | -4.2287 | -0.5107 | -0.0695 |
| C | -4.1132 | 1.211 | -1.4434 | C | -4.047 | 0.996 | -0.2673 |
| C | -4.081 | 2.4105 | -0.5418 | C | -5.1774 | 1.576 | -1.0728 |
| C | -3.0358 | 3.2107 | -0.2522 | C | -6.2151 | 2.3089 | -0.6238 |
| C | -3.2077 | 4.3769 | 0.6884 | C | -7.2493 | 2.842 | -1.5811 |
| O | 6.6739 | -0.5292 | -0.4805 | O | 7.1001 | 0.4577 | -1.0477 |
| O | -1.5886 | -1.5171 | 1.068 | O | -0.7861 | -1.7577 | 1.0499 |
| C | -0.3766 | -2.0961 | 0.9558 | C | 0.4453 | -2.1483 | 0.6673 |
| O | 0.1878 | -2.5597 | 1.9487 | O | 1.178 | -2.7546 | 1.4513 |
| C | -3.8893 | 0.046 | 1.6701 | C | -3.1894 | -0.7398 | 2.2295 |
| C | -1.6379 | 3.0634 | -0.7879 | C | -6.4707 | 2.6559 | 0.8171 |
| O | 0.8277 | 0.4116 | 0.1305 | O | 1.3201 | 0.5924 | 0.3751 |
| O | 2.2192 | 2.5031 | 0.6209 | O | 2.6106 | 2.6102 | 1.2893 |
| O | 0.3284 | -3.7121 | -0.633 | O | 1.0587 | -3.2569 | -1.3411 |
| O | -4.3206 | -2.0176 | 0.5702 | O | -3.4366 | -2.6052 | 0.7607 |
| H | 6.6628 | 2.0598 | 0.2014 | H | 6.9869 | 2.7575 | 0.3185 |
| H | 4.6158 | 3.3594 | 0.6825 | H | 4.9272 | 3.6629 | 1.3427 |
| H | 4.3042 | -1.4395 | -0.5568 | H | 4.8122 | -0.641 | -1.1314 |
| H | 2.137 | -1.9268 | -1.4338 | H | 2.5881 | -1.1281 | -1.8663 |
| H | 2.2913 | -2.3358 | 0.2805 | H | 3.011 | -1.9313 | -0.3478 |
| H | -0.3839 | -2.3431 | -2.5139 | H | 0.0124 | -1.5437 | -2.7329 |
| H | -0.2605 | -0.7137 | -1.8718 | H | 0.047 | -0.1305 | -1.6904 |
| H | -2.5883 | -1.3303 | -2.1652 | H | -2.2218 | -0.9666 | -1.9232 |
| H | -2.3886 | -2.7638 | -1.1452 | H | -1.743 | -2.5668 | -1.3331 |
| H | -1.8075 | 0.102 | -0.1744 | H | -1.4042 | 0.0414 | 0.2734 |
| H | -5.6362 | 0.1041 | -0.3781 | H | -5.1992 | -0.7001 | 0.4119 |
| H | -4.7807 | -0.818 | -1.5646 | H | -4.3146 | -1.0068 | -1.0457 |
| H | -4.7991 | 1.451 | -2.2665 | H | -3.1186 | 1.1979 | -0.8138 |
| H | -3.1494 | 1.0202 | -1.9192 | H | -3.9443 | 1.5049 | 0.6944 |
| H | -5.0456 | 2.6454 | -0.089 | H | -5.1127 | 1.372 | -2.142 |
| H | -4.2315 | 4.4587 | 1.0685 | H | -7.0299 | 2.5771 | -2.6208 |
| H | -2.5417 | 4.2685 | 1.5508 | H | -8.2371 | 2.4381 | -1.3366 |
| H | -2.967 | 5.3148 | 0.1774 | H | -7.2942 | 3.9343 | -1.5194 |
| H | 7.471 | 0.0179 | -0.3757 | H | 7.8619 | 1.0378 | -0.8784 |
| H | -4.9414 | 0.2497 | 1.8997 | H | -4.2066 | -0.845 | 2.6243 |
| H | -3.5131 | -0.5538 | 2.5072 | H | -2.5536 | -1.3593 | 2.872 |
| H | -3.3428 | 0.9916 | 1.669 | H | -2.8787 | 0.3027 | 2.346 |
| H | -1.5227 | 2.2414 | -1.4964 | H | -5.7973 | 2.1492 | 1.5119 |
| H | -1.3357 | 3.9801 | -1.3053 | H | -6.364 | 3.7349 | 0.9688 |
| H | -0.9387 | 2.8873 | 0.0361 | H | -7.4896 | 2.3678 | 1.0979 |
| H | 1.4046 | 1.9567 | 0.5571 | H | 1.8313 | 2.0289 | 1.1451 |
| H | 0.6823 | -4.0938 | 0.1958 | H | 1.556 | -3.7814 | -0.6815 |
| H | -3.7578 | -2.4479 | 1.2419 | H | -2.726 | -3.0501 | 1.2609 |

**Table S28.** Cartesian Coordinates of conformers **1−3e** and **1−3f** in the gas phase (Å) at the B3LYP/6-31(d,p) level.

| **1−3e** | | | | **1−3f** | | | |
| --- | --- | --- | --- | --- | --- | --- | --- |
| C | -5.5517 | 0.1736 | 0.3125 | C | -6.3797 | -0.8782 | -0.063 |
| C | -5.7979 | 1.2426 | -0.5417 | C | -6.964 | 0.3582 | -0.3135 |
| C | -4.7347 | 1.8169 | -1.2357 | C | -6.1529 | 1.4879 | -0.4004 |
| C | -3.4425 | 1.3181 | -1.0696 | C | -4.7723 | 1.3702 | -0.2378 |
| C | -3.1907 | 0.2381 | -0.206 | C | -4.1788 | 0.1213 | 0.013 |
| C | -4.2649 | -0.3325 | 0.4872 | C | -5.0017 | -1.0081 | 0.101 |
| C | -1.7974 | -0.2742 | -0.0386 | C | -2.6986 | 0.0192 | 0.1857 |
| C | -1.5335 | -1.4144 | 0.9269 | C | -2.0663 | -1.3414 | 0.4137 |
| C | -0.0512 | -1.819 | 1.0084 | C | -0.53 | -1.2948 | 0.4902 |
| C | 0.797 | -0.7561 | 1.7054 | C | -0.0427 | -0.6606 | 1.7923 |
| C | 2.2517 | -0.8449 | 1.2748 | C | 1.4012 | -0.2059 | 1.6601 |
| C | 2.3031 | -0.6879 | -0.2422 | C | 1.4702 | 0.8082 | 0.5258 |
| C | 3.7457 | -0.6699 | -0.8328 | C | 2.8858 | 1.4175 | 0.2798 |
| C | 4.6807 | 0.4183 | -0.2542 | C | 3.9158 | 0.3503 | -0.161 |
| C | 4.4285 | 1.879 | -0.6487 | C | 5.2873 | 0.9175 | -0.5504 |
| C | 3.0938 | 2.4421 | -0.2563 | C | 6.2055 | -0.178 | -1.0244 |
| C | 2.742 | 2.9496 | 0.9408 | C | 7.1991 | -0.7732 | -0.3361 |
| C | 1.3559 | 3.4932 | 1.1689 | C | 8.0505 | -1.8354 | -0.9813 |
| O | -6.5595 | -0.4204 | 1.0129 | O | -7.1321 | -2.0113 | 0.0329 |
| O | 1.603 | -1.803 | -0.8572 | O | 1.0324 | 0.2201 | -0.7262 |
| C | 0.4499 | -2.2965 | -0.3637 | C | 0.0526 | -0.7026 | -0.803 |
| O | -0.1507 | -3.1921 | -0.9608 | O | -0.3055 | -1.1402 | -1.898 |
| C | 3.7086 | -0.594 | -2.368 | C | 3.3646 | 2.1999 | 1.5072 |
| C | 3.646 | 3.0237 | 2.1403 | C | 7.5574 | -0.4766 | 1.0939 |
| O | -0.8946 | 0.2559 | -0.6881 | O | -2.0278 | 1.0518 | 0.1476 |
| O | -2.4647 | 1.9483 | -1.7931 | O | -4.0612 | 2.5366 | -0.3383 |
| O | 0.0028 | -2.9926 | 1.8599 | O | -0.075 | -2.6708 | 0.5219 |
| O | 4.3659 | -1.9322 | -0.5044 | O | 2.7547 | 2.3733 | -0.7882 |
| H | -6.7988 | 1.6395 | -0.6803 | H | -8.0367 | 0.4636 | -0.4422 |
| H | -4.9081 | 2.653 | -1.9087 | H | -6.5917 | 2.4634 | -0.5953 |
| H | -4.1375 | -1.1691 | 1.1668 | H | -4.6062 | -1.9998 | 0.2956 |
| H | -1.8811 | -1.1264 | 1.9267 | H | -2.4626 | -1.771 | 1.3418 |
| H | -2.1298 | -2.2786 | 0.6075 | H | -2.3665 | -2.0024 | -0.4094 |
| H | 0.7407 | -0.8705 | 2.7953 | H | -0.1139 | -1.3794 | 2.6183 |
| H | 0.4352 | 0.2543 | 1.4831 | H | -0.6546 | 0.203 | 2.0757 |
| H | 2.815 | -0.0601 | 1.7856 | H | 1.7297 | 0.2339 | 2.6068 |
| H | 2.6659 | -1.8122 | 1.583 | H | 2.0337 | -1.0762 | 1.4538 |
| H | 1.7711 | 0.2128 | -0.5583 | H | 0.7761 | 1.6375 | 0.7179 |
| H | 5.707 | 0.1773 | -0.5693 | H | 3.5218 | -0.1937 | -1.0297 |
| H | 4.7242 | 0.3316 | 0.8379 | H | 4.0519 | -0.3861 | 0.6405 |
| H | 4.5441 | 1.9943 | -1.7334 | H | 5.734 | 1.4659 | 0.283 |
| H | 5.2258 | 2.4999 | -0.2211 | H | 5.1806 | 1.6425 | -1.3661 |
| H | 2.3557 | 2.4524 | -1.0592 | H | 6.0279 | -0.4889 | -2.0543 |
| H | 0.7245 | 3.4093 | 0.2779 | H | 7.767 | -2.0203 | -2.0228 |
| H | 0.862 | 2.9437 | 1.977 | H | 9.103 | -1.5337 | -0.9723 |
| H | 1.4044 | 4.5514 | 1.4453 | H | 7.9543 | -2.7811 | -0.4379 |
| H | -7.3992 | 0.0153 | 0.7885 | H | -8.0656 | -1.7828 | -0.1157 |
| H | 4.7245 | -0.5631 | -2.7786 | H | 4.2185 | 2.842 | 1.2655 |
| H | 3.2368 | -1.4819 | -2.8036 | H | 2.5791 | 2.8754 | 1.8656 |
| H | 3.1648 | 0.2883 | -2.719 | H | 3.6514 | 1.5376 | 2.3297 |
| H | 4.6367 | 2.5993 | 1.9604 | H | 6.8496 | 0.1928 | 1.5883 |
| H | 3.7832 | 4.0666 | 2.4443 | H | 7.5757 | -1.4038 | 1.6768 |
| H | 3.2037 | 2.4746 | 2.9783 | H | 8.5499 | -0.0175 | 1.1459 |
| H | -1.6138 | 1.4879 | -1.6186 | H | -3.1091 | 2.3265 | -0.212 |
| H | -0.3816 | -3.733 | 1.3483 | H | -0.2455 | -3.0479 | -0.3647 |
| H | 3.7649 | -2.6293 | -0.8302 | H | 2.2745 | 1.9198 | -1.5086 |

**Table S29.** Cartesian Coordinates of conformer **1−3g** in the gas phase (Å) at the B3LYP/6-31(d,p) level.

| **1−3g** | | | |
| --- | --- | --- | --- |
| C | -6.2816 | -0.8913 | -0.2721 |
| C | -6.8129 | 0.2471 | -0.8675 |
| C | -5.9781 | 1.3334 | -1.1219 |
| C | -4.6268 | 1.2702 | -0.781 |
| C | -4.0866 | 0.1197 | -0.1814 |
| C | -4.9333 | -0.9661 | 0.0727 |
| C | -2.6371 | 0.075 | 0.1769 |
| C | -2.0573 | -1.1897 | 0.7829 |
| C | -0.5382 | -1.1127 | 1.0171 |
| C | -0.1872 | -0.1895 | 2.1832 |
| C | 1.2715 | 0.2306 | 2.1144 |
| C | 1.4884 | 0.9534 | 0.7914 |
| C | 2.9354 | 1.4955 | 0.5733 |
| C | 3.9853 | 0.3602 | 0.5107 |
| C | 5.403 | 0.8296 | 0.1586 |
| C | 6.3524 | -0.3369 | 0.0812 |
| C | 6.8022 | -0.9535 | -1.0292 |
| C | 7.7843 | -2.0914 | -0.9333 |
| O | -7.0593 | -1.9782 | -0.0015 |
| O | 1.1799 | 0.0884 | -0.3318 |
| C | 0.1935 | -0.8301 | -0.3047 |
| O | -0.0533 | -1.5081 | -1.3037 |
| C | 3.2931 | 2.5439 | 1.6317 |
| C | 6.3883 | -0.609 | -2.4337 |
| O | -1.9493 | 1.0775 | -0.0221 |
| O | -3.8892 | 2.388 | -1.0684 |
| O | -0.1126 | -2.4395 | 1.4167 |
| O | 2.9433 | 2.1769 | -0.6943 |
| H | -7.8625 | 0.3092 | -1.1379 |
| H | -6.3755 | 2.2325 | -1.5865 |
| H | -4.579 | -1.8814 | 0.5359 |
| H | -2.561 | -1.3956 | 1.7352 |
| H | -2.2758 | -2.0265 | 0.1074 |
| H | -0.3653 | -0.6967 | 3.1398 |
| H | -0.809 | 0.7126 | 2.1869 |
| H | 1.5009 | 0.8805 | 2.9645 |
| H | 1.905 | -0.6601 | 2.1888 |
| H | 0.7955 | 1.8013 | 0.7065 |
| H | 3.679 | -0.3702 | -0.2498 |
| H | 4.0152 | -0.1721 | 1.4693 |
| H | 5.7814 | 1.5085 | 0.9315 |
| H | 5.4033 | 1.4042 | -0.7723 |
| H | 6.7092 | -0.6865 | 1.0499 |
| H | 8.0678 | -2.31 | 0.1017 |
| H | 7.3522 | -3.0029 | -1.3591 |
| H | 8.6991 | -1.849 | -1.4839 |
| H | -7.966 | -1.7997 | -0.304 |
| H | 4.1868 | 3.1103 | 1.3479 |
| H | 2.4911 | 3.285 | 1.7282 |
| H | 3.4664 | 2.0934 | 2.6136 |
| H | 5.5772 | 0.1214 | -2.48 |
| H | 7.2407 | -0.2036 | -2.9884 |
| H | 6.0353 | -1.507 | -2.9522 |
| H | -2.9611 | 2.2224 | -0.7893 |
| H | -0.1904 | -3.0133 | 0.628 |
| H | 2.5524 | 1.559 | -1.3428 |

**Table S30.** Cartesian Coordinates of conformers **1−4a** and **1−4b** in the gas phase (Å) at the B3LYP/6-31(d,p) level.

| **1−4a** | | | | **1−4b** | | | |
| --- | --- | --- | --- | --- | --- | --- | --- |
| C | 5.4854 | 0.2761 | 0.0306 | C | -5.4942 | -0.12 | 0.2739 |
| C | 5.5319 | 1.6229 | -0.312 | C | -5.7775 | 1.0525 | -0.4175 |
| C | 4.3524 | 2.276 | -0.6646 | C | -4.7322 | 1.7702 | -0.9954 |
| C | 3.144 | 1.5792 | -0.6697 | C | -3.4206 | 1.31 | -0.877 |
| C | 3.0938 | 0.2197 | -0.317 | C | -3.1314 | 0.1259 | -0.1771 |
| C | 4.2839 | -0.4299 | 0.0311 | C | -4.1878 | -0.5889 | 0.4 |
| C | 1.788 | -0.5038 | -0.3226 | C | -1.7185 | -0.3424 | -0.0562 |
| C | 1.7157 | -1.9199 | 0.2192 | C | -1.4135 | -1.5984 | 0.7376 |
| C | 0.2765 | -2.449 | 0.3551 | C | 0.0877 | -1.9298 | 0.8003 |
| C | -0.3356 | -2.8128 | -0.9976 | C | 0.8516 | -0.9474 | 1.6877 |
| C | -1.8531 | -2.7114 | -0.9604 | C | 2.3357 | -0.9726 | 1.3629 |
| C | -2.2137 | -1.2841 | -0.5573 | C | 2.4953 | -0.5933 | -0.1033 |
| C | -3.7437 | -0.9869 | -0.5493 | C | 3.9692 | -0.4967 | -0.5913 |
| C | -4.0712 | 0.4234 | 0.0061 | C | 4.825 | 0.5694 | 0.1319 |
| C | -3.5171 | 1.588 | -0.8183 | C | 4.3451 | 2.0255 | 0.0081 |
| C | -3.7591 | 2.9041 | -0.1319 | C | 3.3269 | 2.4179 | 1.0457 |
| C | -2.871 | 3.6204 | 0.5853 | C | 2.1078 | 2.9603 | 0.856 |
| C | -3.2582 | 4.9469 | 1.1848 | C | 1.2336 | 3.3124 | 2.032 |
| O | 6.6136 | -0.4061 | 0.3782 | O | -6.4834 | -0.8559 | 0.8562 |
| O | -1.7211 | -1.0203 | 0.7796 | O | 1.826 | -1.5668 | -0.9508 |
| C | -0.5506 | -1.5025 | 1.2378 | C | 0.6533 | -2.1417 | -0.6139 |
| O | -0.1721 | -1.2426 | 2.3817 | O | 0.0917 | -2.9138 | -1.3934 |
| C | -4.3715 | -1.1854 | -1.9326 | C | 4.6905 | -1.8531 | -0.5037 |
| C | -1.4564 | 3.1976 | 0.8792 | C | 1.4906 | 3.2767 | -0.4794 |
| O | 0.799 | 0.0744 | -0.7758 | O | -0.8323 | 0.3157 | -0.6026 |
| O | 2.0405 | 2.3047 | -1.0338 | O | -2.4629 | 2.0828 | -1.4786 |
| O | 0.3651 | -3.6903 | 1.099 | O | 0.1971 | -3.2217 | 1.4508 |
| O | -4.385 | -1.9296 | 0.3299 | O | 3.9476 | -0.1485 | -1.9894 |
| H | 6.4659 | 2.1763 | -0.3127 | H | -6.7937 | 1.421 | -0.5178 |
| H | 4.3697 | 3.3283 | -0.9373 | H | -4.9343 | 2.6886 | -1.5412 |
| H | 4.3123 | -1.4803 | 0.3032 | H | -4.0312 | -1.5113 | 0.9501 |
| H | 2.2915 | -2.5845 | -0.436 | H | -1.7976 | -1.4825 | 1.7584 |
| H | 2.195 | -1.9355 | 1.2064 | H | -1.9532 | -2.4348 | 0.2756 |
| H | -0.0537 | -3.8325 | -1.2884 | H | 0.4854 | 0.0775 | 1.5621 |
| H | 0.0338 | -2.1528 | -1.7913 | H | 0.7153 | -1.2008 | 2.7465 |
| H | -2.2489 | -2.9609 | -1.9493 | H | 2.8624 | -0.2775 | 2.0221 |
| H | -2.2511 | -3.437 | -0.2413 | H | 2.7277 | -1.9777 | 1.5557 |
| H | -1.7207 | -0.5787 | -1.235 | H | 1.9942 | 0.3538 | -0.3227 |
| H | -5.1627 | 0.5264 | 0.0898 | H | 4.9631 | 0.3014 | 1.1866 |
| H | -3.713 | 0.4976 | 1.0422 | H | 5.8276 | 0.5461 | -0.3193 |
| H | -2.4531 | 1.4568 | -1.0286 | H | 5.2146 | 2.6757 | 0.1739 |
| H | -4.0112 | 1.6234 | -1.7965 | H | 4.0156 | 2.2311 | -1.0127 |
| H | -4.7667 | 3.2995 | -0.2597 | H | 3.6628 | 2.2638 | 2.0718 |
| H | -4.2899 | 5.2258 | 0.946 | H | 1.7062 | 3.0697 | 2.9895 |
| H | -3.1672 | 4.9119 | 2.2754 | H | 1.0122 | 4.3847 | 2.0321 |
| H | -2.6039 | 5.7386 | 0.8055 | H | 0.2888 | 2.7613 | 1.9799 |
| H | 7.3716 | 0.2022 | 0.3488 | H | -7.3392 | -0.4296 | 0.679 |
| H | -5.425 | -0.8819 | -1.9267 | H | 5.683 | -1.7933 | -0.9664 |
| H | -4.3703 | -2.2405 | -2.2262 | H | 4.1508 | -2.6318 | -1.0536 |
| H | -3.8514 | -0.6089 | -2.7036 | H | 4.8188 | -2.1825 | 0.5317 |
| H | -1.2357 | 2.1731 | 0.5702 | H | 2.0904 | 2.9466 | -1.3293 |
| H | -0.7513 | 3.8634 | 0.3713 | H | 0.5148 | 2.7884 | -0.5668 |
| H | -1.2648 | 3.2497 | 1.9564 | H | 1.3427 | 4.3573 | -0.5769 |
| H | 1.2667 | 1.6989 | -1.0424 | H | -1.5944 | 1.6369 | -1.361 |
| H | 0.5682 | -3.4524 | 2.0264 | H | -0.1519 | -3.886 | 0.8227 |
| H | -3.9773 | -1.8135 | 1.2091 | H | 3.2943 | -0.7406 | -2.411 |

**Table S31.** Cartesian Coordinates of conformers **1−4c** and **1−4d** in the gas phase (Å) at the B3LYP/6-31(d,p) level.

| **1−4c** | | | | **1−4d** | | | |
| --- | --- | --- | --- | --- | --- | --- | --- |
| C | -5.8719 | 0.8965 | 0.5038 | C | 5.5782 | 0.1865 | -0.0983 |
| C | -6.0739 | 1.8395 | -0.4977 | C | 5.695 | 1.5455 | 0.1713 |
| C | -5.0078 | 2.1943 | -1.3218 | C | 4.5401 | 2.3062 | 0.3422 |
| C | -3.7568 | 1.6047 | -1.1378 | C | 3.2864 | 1.7028 | 0.2431 |
| C | -3.5505 | 0.6495 | -0.1278 | C | 3.1654 | 0.3289 | -0.0285 |
| C | -4.6269 | 0.3007 | 0.697 | C | 4.3311 | -0.4273 | -0.2 |
| C | -2.2021 | 0.0327 | 0.0524 | C | 1.812 | -0.2942 | -0.1348 |
| C | -2.0032 | -1.0266 | 1.1207 | C | 1.6944 | -1.7816 | -0.4103 |
| C | -0.5841 | -1.6218 | 1.1315 | C | 0.2399 | -2.2829 | -0.4474 |
| C | 0.4454 | -0.6326 | 1.6762 | C | -0.4981 | -1.8084 | -1.6983 |
| C | 1.8494 | -1.0295 | 1.2525 | C | -2.0018 | -1.8075 | -1.4774 |
| C | 1.8917 | -1.0474 | -0.2696 | C | -2.3018 | -0.8954 | -0.2904 |
| C | 3.2854 | -1.3962 | -0.8728 | C | -3.8198 | -0.7438 | 0.0212 |
| C | 4.4152 | -0.4342 | -0.445 | C | -4.0988 | 0.0553 | 1.3231 |
| C | 4.1495 | 1.0391 | -0.7753 | C | -3.3932 | 1.4063 | 1.5048 |
| C | 5.3153 | 1.8993 | -0.367 | C | -3.8107 | 2.4571 | 0.5165 |
| C | 5.4078 | 2.7097 | 0.7055 | C | -3.0254 | 3.2078 | -0.2812 |
| C | 6.6499 | 3.5297 | 0.9406 | C | -3.6352 | 4.2096 | -1.2281 |
| O | -6.8837 | 0.519 | 1.3362 | O | 6.6793 | -0.5981 | -0.2746 |
| O | 0.9412 | -2.0067 | -0.8026 | O | -1.6713 | -1.4226 | 0.9031 |
| C | -0.2564 | -2.249 | -0.233 | C | -0.4555 | -2.0034 | 0.8938 |
| O | -1.0431 | -3.0433 | -0.7516 | O | 0.0575 | -2.3908 | 1.9455 |
| C | 3.7102 | -2.8393 | -0.5562 | C | -4.5923 | -0.1754 | -1.1747 |
| C | 4.3457 | 2.8832 | 1.7555 | C | -1.5227 | 3.1579 | -0.3279 |
| O | -1.279 | 0.4109 | -0.6706 | O | 0.8211 | 0.4261 | -0.003 |
| O | -2.773 | 2.0201 | -1.9954 | O | 2.2112 | 2.532 | 0.4254 |
| O | -0.6061 | -2.7294 | 2.0667 | O | 0.3079 | -3.728 | -0.5479 |
| O | 3.1727 | -1.313 | -2.3056 | O | -4.371 | -2.06 | 0.2457 |
| H | -7.0421 | 2.3057 | -0.6517 | H | 6.6647 | 2.0269 | 0.2514 |
| H | -5.1464 | 2.9301 | -2.1101 | H | 4.6117 | 3.3703 | 0.5539 |
| H | -4.5325 | -0.4274 | 1.4963 | H | 4.3058 | -1.4913 | -0.4128 |
| H | -2.221 | -0.5914 | 2.1036 | H | 2.1789 | -2.0094 | -1.3676 |
| H | -2.7302 | -1.8302 | 0.9479 | H | 2.2431 | -2.32 | 0.3729 |
| H | 0.4017 | -0.5989 | 2.7721 | H | -0.1922 | -0.7957 | -1.9851 |
| H | 0.2523 | 0.3872 | 1.3246 | H | -0.2644 | -2.4562 | -2.5526 |
| H | 2.5649 | -0.3107 | 1.6631 | H | -2.4932 | -1.4525 | -2.3884 |
| H | 2.0863 | -2.0159 | 1.6667 | H | -2.3434 | -2.8312 | -1.2842 |
| H | 1.5714 | -0.0792 | -0.6733 | H | -1.8765 | 0.0975 | -0.4735 |
| H | 4.613 | -0.5421 | 0.6289 | H | -5.1839 | 0.1931 | 1.4316 |
| H | 5.3398 | -0.7306 | -0.9608 | H | -3.8249 | -0.5712 | 2.184 |
| H | 3.9989 | 1.1627 | -1.8545 | H | -3.651 | 1.7845 | 2.5026 |
| H | 3.2276 | 1.3855 | -0.3019 | H | -2.3123 | 1.2512 | 1.5225 |
| H | 6.1613 | 1.8472 | -1.0533 | H | -4.8891 | 2.6146 | 0.4691 |
| H | 7.3992 | 3.3851 | 0.1552 | H | -4.7292 | 4.2163 | -1.1799 |
| H | 6.3999 | 4.5953 | 0.9688 | H | -3.2857 | 5.2194 | -0.9895 |
| H | 7.1114 | 3.2545 | 1.8947 | H | -3.3505 | 3.9763 | -2.2594 |
| H | -7.6931 | 0.9952 | 1.0841 | H | 7.4765 | -0.0525 | -0.164 |
| H | 4.6412 | -3.0903 | -1.0784 | H | -5.6238 | 0.0678 | -0.8948 |
| H | 2.9664 | -3.5636 | -0.9055 | H | -4.6739 | -0.9125 | -1.9815 |
| H | 3.8703 | -2.9933 | 0.5151 | H | -4.1233 | 0.7224 | -1.5843 |
| H | 3.5045 | 2.1961 | 1.6406 | H | -1.0832 | 2.5285 | 0.4475 |
| H | 4.7679 | 2.7036 | 2.7501 | H | -1.1894 | 2.7762 | -1.2984 |
| H | 3.9524 | 3.9046 | 1.7277 | H | -1.1082 | 4.1627 | -0.1921 |
| H | -1.9474 | 1.5351 | -1.7724 | H | 1.3954 | 1.9888 | 0.3529 |
| H | -1.147 | -3.4356 | 1.6591 | H | 0.6215 | -4.0594 | 0.3177 |
| H | 2.3882 | -1.8399 | -2.554 | H | -3.8423 | -2.467 | 0.9577 |

**Table S32.** Cartesian Coordinates of conformer **1−4e** in the gas phase (Å) at the B3LYP/6-31(d,p) level.

| **1−4e** | | | |
| --- | --- | --- | --- |
| C | 5.6129 | -0.1968 | -0.0691 |
| C | 5.8644 | 1.1595 | 0.1047 |
| C | 4.7912 | 2.0439 | 0.1921 |
| C | 3.484 | 1.5648 | 0.1063 |
| C | 3.2265 | 0.1941 | -0.0673 |
| C | 4.311 | -0.6868 | -0.1562 |
| C | 1.8174 | -0.2947 | -0.1594 |
| C | 1.5536 | -1.7812 | -0.3068 |
| C | 0.0582 | -2.1433 | -0.3142 |
| C | -0.6234 | -1.7234 | -1.6134 |
| C | -2.1339 | -1.7276 | -1.448 |
| C | -2.4986 | -0.7334 | -0.3461 |
| C | -4.0303 | -0.6566 | -0.0592 |
| C | -4.405 | 0.2437 | 1.1486 |
| C | -4.3263 | 1.7642 | 0.962 |
| C | -2.9299 | 2.302 | 0.8471 |
| C | -2.4125 | 3.0785 | -0.1255 |
| C | -0.9758 | 3.5297 | -0.0547 |
| O | 6.6303 | -1.0999 | -0.1621 |
| O | -1.8486 | -1.0983 | 0.8991 |
| C | -0.6239 | -1.6558 | 0.9719 |
| O | -0.1066 | -1.8772 | 2.0684 |
| C | -4.8303 | -0.2667 | -1.3063 |
| C | -3.1487 | 3.5756 | -1.3384 |
| O | 0.9041 | 0.5312 | -0.1218 |
| O | 2.4964 | 2.5093 | 0.2011 |
| O | -0.014 | -3.59 | -0.2689 |
| O | -4.4716 | -1.9846 | 0.3011 |
| H | 6.877 | 1.5448 | 0.1733 |
| H | 4.9681 | 3.108 | 0.3279 |
| H | 4.18 | -1.7555 | -0.2931 |
| H | 2.0163 | -2.1373 | -1.2353 |
| H | 2.0456 | -2.3006 | 0.5254 |
| H | -0.3547 | -2.4112 | -2.4249 |
| H | -0.3099 | -0.7227 | -1.9308 |
| H | -2.5955 | -1.4478 | -2.4002 |
| H | -2.4714 | -2.7392 | -1.1947 |
| H | -2.1279 | 0.256 | -0.6267 |
| H | -5.4422 | 0.0084 | 1.4298 |
| H | -3.8205 | -0.0504 | 2.0306 |
| H | -4.9651 | 2.066 | 0.1294 |
| H | -4.7708 | 2.2296 | 1.8517 |
| H | -2.2811 | 2.0254 | 1.6805 |
| H | -0.4672 | 3.1593 | 0.8414 |
| H | -0.4225 | 3.1667 | -0.927 |
| H | -0.9218 | 4.6231 | -0.0379 |
| H | 7.4773 | -0.631 | -0.0717 |
| H | -5.89 | -0.1307 | -1.0602 |
| H | -4.8041 | -1.057 | -2.0642 |
| H | -4.4586 | 0.6583 | -1.7565 |
| H | -4.1974 | 3.2752 | -1.3698 |
| H | -3.1248 | 4.6701 | -1.3698 |
| H | -2.6717 | 3.1946 | -2.2476 |
| H | 1.6318 | 2.0459 | 0.1364 |
| H | 0.2845 | -3.864 | 0.6212 |
| H | -3.9333 | -2.2562 | 1.0686 |

**Table S33.** Cartesian Coordinates of conformers **1−5a** and **1−5b** in the gas phase (Å) at the B3LYP/6-31(d,p) level.

| **1−5a** | | | | **1−5b** | | | |
| --- | --- | --- | --- | --- | --- | --- | --- |
| C | 5.6704 | -1.0187 | -0.1503 | C | 5.9487 | -0.3242 | -0.6016 |
| C | 6.2797 | 0.1489 | -0.5966 | C | 6.3908 | 0.994 | -0.5831 |
| C | 5.5324 | 1.3234 | -0.6632 | C | 5.5285 | 1.9942 | -0.1378 |
| C | 4.1888 | 1.3175 | -0.2883 | C | 4.2378 | 1.6682 | 0.2788 |
| C | 3.5681 | 0.1359 | 0.1502 | C | 3.785 | 0.3387 | 0.2482 |
| C | 4.3284 | -1.0371 | 0.2246 | C | 4.6608 | -0.6615 | -0.1902 |
| C | 2.1286 | 0.1518 | 0.5412 | C | 2.3989 | 0.0134 | 0.6935 |
| C | 1.3985 | -1.1597 | 0.7876 | C | 1.8332 | -1.377 | 0.4482 |
| C | -0.1327 | -0.9833 | 0.7338 | C | 0.2952 | -1.3989 | 0.5602 |
| C | -0.8078 | -2.3111 | 1.109 | C | -0.2025 | -2.8447 | 0.4173 |
| C | -2.3347 | -2.2226 | 1.0094 | C | -1.733 | -2.9328 | 0.4234 |
| C | -2.8047 | -1.0272 | 0.1635 | C | -2.4117 | -1.5707 | 0.2099 |
| C | -4.2508 | -1.1772 | -0.3879 | C | -3.8913 | -1.6744 | -0.2689 |
| C | -4.6786 | -0.0443 | -1.3569 | C | -4.5184 | -0.2936 | -0.5866 |
| C | -4.7399 | 1.3821 | -0.7953 | C | -4.596 | 0.6756 | 0.5946 |
| C | -3.4079 | 2.0799 | -0.7755 | C | -5.185 | 1.9945 | 0.1743 |
| C | -2.7955 | 2.6909 | 0.2577 | C | -4.5142 | 3.117 | -0.1511 |
| C | -1.4615 | 3.3659 | 0.0611 | C | -5.254 | 4.3754 | -0.5222 |
| O | 6.3633 | -2.1897 | -0.0597 | O | 6.7591 | -1.3378 | -1.0205 |
| O | -1.9122 | -0.8525 | -0.9603 | O | -1.6784 | -0.8095 | -0.779 |
| C | -0.5844 | -0.6865 | -0.7166 | C | -0.3413 | -0.6246 | -0.6154 |
| O | 0.1882 | -0.3806 | -1.6191 | O | 0.2974 | 0.0789 | -1.391 |
| C | -5.2657 | -1.3394 | 0.7495 | C | -4.7523 | -2.4526 | 0.731 |
| C | -3.3273 | 2.7826 | 1.661 | C | -3.0153 | 3.2413 | -0.2023 |
| O | 1.5651 | 1.2407 | 0.6877 | O | 1.7454 | 0.8776 | 1.2859 |
| O | 3.5398 | 2.5204 | -0.3771 | O | 3.4682 | 2.7162 | 0.7092 |
| O | -0.5402 | -0.0223 | 1.7044 | O | -0.102 | -0.9432 | 1.8525 |
| O | -4.3135 | -2.3928 | -1.1638 | O | -3.9203 | -2.4196 | -1.5019 |
| H | 7.3245 | 0.1674 | -0.8905 | H | 7.3929 | 1.2613 | -0.9036 |
| H | 5.9926 | 2.2471 | -1.005 | H | 5.8579 | 3.03 | -0.1116 |
| H | 3.9113 | -1.9751 | 0.5762 | H | 4.3766 | -1.7083 | -0.2158 |
| H | 1.7069 | -1.8973 | 0.0384 | H | 2.1311 | -1.723 | -0.5478 |
| H | 1.6997 | -1.5052 | 1.784 | H | 2.2719 | -2.0345 | 1.2085 |
| H | -0.5401 | -2.6115 | 2.1297 | H | 0.1874 | -3.4718 | 1.2289 |
| H | -0.4596 | -3.1091 | 0.4396 | H | 0.167 | -3.2755 | -0.5229 |
| H | -2.7675 | -2.1448 | 2.0141 | H | -2.0742 | -3.3622 | 1.3731 |
| H | -2.6961 | -3.1706 | 0.5941 | H | -2.0271 | -3.6452 | -0.3565 |
| H | -2.7705 | -0.1214 | 0.7752 | H | -2.3992 | -1.0059 | 1.1481 |
| H | -4.0458 | -0.0653 | -2.255 | H | -3.9623 | 0.1752 | -1.4103 |
| H | -5.6808 | -0.2946 | -1.7348 | H | -5.527 | -0.451 | -0.9953 |
| H | -5.3842 | 1.9711 | -1.4616 | H | -5.233 | 0.2609 | 1.3843 |
| H | -5.2397 | 1.389 | 0.1747 | H | -3.6136 | 0.8242 | 1.0503 |
| H | -2.9083 | 2.1005 | -1.7461 | H | -6.2746 | 2.0135 | 0.1525 |
| H | -1.0721 | 3.2337 | -0.9538 | H | -6.3406 | 4.2498 | -0.4697 |
| H | -1.5518 | 4.441 | 0.2476 | H | -4.9806 | 5.1892 | 0.1576 |
| H | -0.7214 | 2.9558 | 0.7547 | H | -5.003 | 4.6761 | -1.5446 |
| H | 7.275 | -2.039 | -0.3623 | H | 7.6154 | -0.964 | -1.2896 |
| H | -6.2873 | -1.3898 | 0.3555 | H | -5.801 | -2.4661 | 0.412 |
| H | -5.1093 | -2.2786 | 1.2917 | H | -4.4446 | -3.5024 | 0.7892 |
| H | -5.212 | -0.5153 | 1.467 | H | -4.7022 | -2.0221 | 1.7357 |
| H | -4.2846 | 2.2771 | 1.8001 | H | -2.496 | 2.2902 | -0.0622 |
| H | -2.6173 | 2.329 | 2.3609 | H | -2.7037 | 3.6314 | -1.1773 |
| H | -3.4656 | 3.8318 | 1.9424 | H | -2.6685 | 3.9327 | 0.5725 |
| H | 2.6244 | 2.3954 | -0.0422 | H | 2.6085 | 2.3543 | 1.0187 |
| H | -0.0715 | 0.8126 | 1.4824 | H | 0.2743 | -0.0407 | 1.9584 |
| H | -3.6308 | -2.3127 | -1.8566 | H | -3.3551 | -1.9346 | -2.1328 |

**Table S34.** Cartesian Coordinates of conformers **1−5c** and **1−5d** in the gas phase (Å) at the B3LYP/6-31(d,p) level.

| **1−5c** | | | | **1−5d** | | | |
| --- | --- | --- | --- | --- | --- | --- | --- |
| C | 6.2793 | -0.1542 | -0.4575 | C | -6.0099 | -0.6222 | -0.298 |
| C | 6.7003 | 1.1666 | -0.5656 | C | -6.5993 | 0.4061 | 0.4292 |
| C | 5.8061 | 2.1921 | -0.2636 | C | -5.806 | 1.4567 | 0.8865 |
| C | 4.5051 | 1.8879 | 0.1376 | C | -4.4373 | 1.4664 | 0.6168 |
| C | 4.0742 | 0.5544 | 0.2346 | C | -3.838 | 0.4217 | -0.1064 |
| C | 4.9817 | -0.4701 | -0.06 | C | -4.6436 | -0.6245 | -0.5715 |
| C | 2.677 | 0.252 | 0.661 | C | -2.3717 | 0.45 | -0.3794 |
| C | 2.1437 | -1.1679 | 0.5498 | C | -1.6875 | -0.7783 | -0.9596 |
| C | 0.6031 | -1.2091 | 0.6094 | C | -0.1582 | -0.7292 | -0.7657 |
| C | 0.1356 | -2.6714 | 0.6079 | C | 0.4803 | -1.9341 | -1.4724 |
| C | -1.3918 | -2.7904 | 0.566 | C | 1.9918 | -2.0163 | -1.2291 |
| C | -2.0876 | -1.4727 | 0.1892 | C | 2.4663 | -1.0924 | -0.0972 |
| C | -3.5437 | -1.662 | -0.3319 | C | 3.8607 | -1.47 | 0.4813 |
| C | -4.1953 | -0.335 | -0.7963 | C | 4.2767 | -0.5912 | 1.6916 |
| C | -4.3082 | 0.7478 | 0.2791 | C | 4.1588 | 0.9328 | 1.5535 |
| C | -4.9976 | 1.9724 | -0.2583 | C | 5.0834 | 1.5337 | 0.5348 |
| C | -6.2706 | 2.3587 | -0.0448 | C | 4.7708 | 2.319 | -0.5151 |
| C | -6.7925 | 3.6421 | -0.6369 | C | 5.8516 | 2.824 | -1.4371 |
| O | 7.1208 | -1.1912 | -0.7337 | O | -6.7467 | -1.6665 | -0.7736 |
| O | -1.3332 | -0.7981 | -0.846 | O | 1.5131 | -1.1269 | 0.992 |
| C | -0.003 | -0.5811 | -0.665 | C | 0.2006 | -0.8876 | 0.7288 |
| O | 0.6536 | 0.0369 | -1.4964 | O | -0.6265 | -0.8499 | 1.6338 |
| C | -4.4238 | -2.373 | 0.7018 | C | 4.9361 | -1.4958 | -0.6102 |
| C | -7.2849 | 1.5898 | 0.7561 | C | 3.3872 | 2.7752 | -0.8903 |
| O | 1.9871 | 1.1624 | 1.13 | O | -1.7443 | 1.4901 | -0.1571 |
| O | 3.7036 | 2.9611 | 0.4229 | O | -3.7431 | 2.5447 | 1.0975 |
| O | 0.1502 | -0.6238 | 1.8292 | O | 0.3674 | 0.447 | -1.3791 |
| O | -3.5055 | -2.5166 | -1.4916 | O | 3.7909 | -2.8212 | 0.9893 |
| H | 7.7099 | 1.4172 | -0.8757 | H | -7.663 | 0.4114 | 0.6453 |
| H | 6.1184 | 3.2308 | -0.3376 | H | -6.2495 | 2.2721 | 1.4525 |
| H | 4.7148 | -1.5189 | 0.0173 | H | -4.243 | -1.4479 | -1.1536 |
| H | 2.4842 | -1.6142 | -0.3911 | H | -2.087 | -1.6798 | -0.4817 |
| H | 2.5644 | -1.7296 | 1.3926 | H | -1.9271 | -0.7968 | -2.0297 |
| H | 0.5493 | -3.1951 | -0.2644 | H | 0.3018 | -1.888 | -2.5541 |
| H | 0.5035 | -3.1975 | 1.4977 | H | 0.0199 | -2.8645 | -1.114 |
| H | -1.7622 | -3.1291 | 1.5411 | H | 2.5283 | -1.7656 | -2.1521 |
| H | -1.6424 | -3.5844 | -0.1475 | H | 2.2354 | -3.0623 | -1.0081 |
| H | -2.1249 | -0.8142 | 1.0639 | H | 2.5227 | -0.0642 | -0.4666 |
| H | -3.6402 | 0.063 | -1.6573 | H | 3.6696 | -0.8836 | 2.5604 |
| H | -5.1945 | -0.5593 | -1.1967 | H | 5.3031 | -0.8524 | 1.9856 |
| H | -4.8188 | 0.3654 | 1.1666 | H | 3.1144 | 1.2052 | 1.3874 |
| H | -3.3109 | 1.055 | 0.6142 | H | 4.4219 | 1.3753 | 2.5233 |
| H | -4.3599 | 2.6057 | -0.8762 | H | 6.1351 | 1.2955 | 0.701 |
| H | -6.0271 | 4.1787 | -1.2074 | H | 6.8466 | 2.4714 | -1.1457 |
| H | -7.628 | 3.4358 | -1.3138 | H | 5.6655 | 2.482 | -2.4606 |
| H | -7.1435 | 4.3094 | 0.157 | H | 5.8726 | 3.9187 | -1.4334 |
| H | 7.9812 | -0.8319 | -1.0098 | H | -7.6725 | -1.5471 | -0.5012 |
| H | -5.4602 | -2.4382 | 0.3505 | H | 5.9309 | -1.6475 | -0.1761 |
| H | -4.0971 | -3.4069 | 0.8592 | H | 4.7813 | -2.336 | -1.297 |
| H | -4.4184 | -1.8595 | 1.6678 | H | 4.9514 | -0.5781 | -1.2024 |
| H | -6.9367 | 0.6004 | 1.0616 | H | 2.6163 | 2.4701 | -0.1805 |
| H | -7.5528 | 2.1478 | 1.6591 | H | 3.355 | 3.8685 | -0.9473 |
| H | -8.1934 | 1.4363 | 0.1637 | H | 3.1126 | 2.3712 | -1.8704 |
| H | 2.8375 | 2.6185 | 0.7363 | H | -2.8089 | 2.4592 | 0.8049 |
| H | 0.5077 | 0.2922 | 1.8492 | H | -0.0921 | 1.209 | -0.9603 |
| H | -2.9239 | -2.0781 | -2.1413 | H | 3.0545 | -2.8391 | 1.6295 |

**Table S35.** Cartesian Coordinates of conformers **1−6a** and **1−6b** in the gas phase (Å) at the B3LYP/6-31(d,p) level.

| **1−6a** | | | | **1−6b** | | | |
| --- | --- | --- | --- | --- | --- | --- | --- |
| C | 6.1062 | -0.8076 | -1.0935 | C | 6.3804 | 0.5504 | -0.9218 |
| C | 6.9506 | -0.3893 | -0.0709 | C | 7.1374 | -0.2185 | -0.0448 |
| C | 6.3999 | 0.0034 | 1.1476 | C | 6.496 | -1.148 | 0.7719 |
| C | 5.017 | -0.0222 | 1.3291 | C | 5.1104 | -1.2957 | 0.7065 |
| C | 4.1618 | -0.432 | 0.2928 | C | 4.3426 | -0.5104 | -0.1695 |
| C | 4.7233 | -0.8352 | -0.9245 | C | 4.9956 | 0.4133 | -0.9942 |
| C | 2.6855 | -0.4473 | 0.5072 | C | 2.8615 | -0.6816 | -0.2196 |
| C | 1.753 | -0.672 | -0.6731 | C | 2.0118 | 0.3134 | -0.9947 |
| C | 0.3102 | -0.2274 | -0.3578 | C | 0.5269 | 0.243 | -0.5839 |
| C | -0.5994 | -0.5854 | -1.5413 | C | -0.2909 | 1.1849 | -1.4786 |
| C | -2.0328 | -0.0817 | -1.3413 | C | -1.7645 | 1.2432 | -1.0618 |
| C | -2.1603 | 0.9374 | -0.1994 | C | -2.0233 | 0.6367 | 0.3255 |
| C | -3.4488 | 1.81 | -0.2869 | C | -3.362 | 1.1082 | 0.9684 |
| C | -4.7516 | 0.9862 | -0.384 | C | -4.5992 | 0.8682 | 0.0757 |
| C | -5.0356 | 0.0761 | 0.8138 | C | -4.8543 | -0.5929 | -0.3021 |
| C | -6.3 | -0.7115 | 0.6027 | C | -6.0747 | -0.7209 | -1.1727 |
| C | -6.4213 | -2.0285 | 0.3448 | C | -7.3005 | -1.1472 | -0.811 |
| C | -7.7832 | -2.6549 | 0.188 | C | -8.4094 | -1.2464 | -1.8262 |
| O | 6.5991 | -1.2094 | -2.2998 | O | 6.9641 | 1.4662 | -1.7466 |
| O | -1.0202 | 1.8335 | -0.2164 | O | -0.9485 | 1.0054 | 1.2265 |
| C | 0.2351 | 1.3102 | -0.2306 | C | 0.339 | 0.7746 | 0.8541 |
| O | 1.2217 | 2.0378 | -0.189 | O | 1.2692 | 1.0234 | 1.6138 |
| C | -3.5139 | 2.8046 | 0.8829 | C | -3.555 | 0.4729 | 2.3549 |
| C | -5.2789 | -2.992 | 0.177 | C | -7.7027 | -1.5417 | 0.5835 |
| O | 2.2485 | -0.3085 | 1.6537 | O | 2.3547 | -1.6547 | 0.3469 |
| O | 4.5708 | 0.371 | 2.5628 | O | 4.5723 | -2.2408 | 1.5388 |
| O | -0.1783 | -0.9399 | 0.7778 | O | 0.0279 | -1.0766 | -0.7966 |
| O | -3.3782 | 2.6002 | -1.49 | O | -3.2849 | 2.5316 | 1.1805 |
| H | 8.0283 | -0.3653 | -0.1985 | H | 8.2162 | -0.1143 | 0.0155 |
| H | 7.0443 | 0.329 | 1.9605 | H | 7.0716 | -1.7616 | 1.4605 |
| H | 4.1167 | -1.1824 | -1.7545 | H | 4.46 | 1.0326 | -1.7062 |
| H | 2.1234 | -0.1182 | -1.5429 | H | 2.3895 | 1.3278 | -0.8248 |
| H | 1.7728 | -1.7468 | -0.8902 | H | 2.1173 | 0.0586 | -2.0562 |
| H | -0.6282 | -1.6716 | -1.6939 | H | -0.2361 | 0.8682 | -2.5277 |
| H | -0.2015 | -0.1452 | -2.4655 | H | 0.1246 | 2.2004 | -1.4313 |
| H | -2.3654 | 0.3556 | -2.2906 | H | -2.0749 | 2.2947 | -1.0897 |
| H | -2.6974 | -0.9316 | -1.1477 | H | -2.3786 | 0.7246 | -1.8075 |
| H | -2.1612 | 0.4291 | 0.7713 | H | -2.0427 | -0.4576 | 0.2698 |
| H | -5.5953 | 1.6779 | -0.5216 | H | -5.4844 | 1.2709 | 0.589 |
| H | -4.7379 | 0.392 | -1.3077 | H | -4.5154 | 1.4775 | -0.8346 |
| H | -4.1871 | -0.5825 | 1.0113 | H | -4.0052 | -0.9945 | -0.8665 |
| H | -5.1686 | 0.6775 | 1.7205 | H | -4.9406 | -1.2164 | 0.5914 |
| H | -7.21 | -0.1163 | 0.6875 | H | -5.91 | -0.4416 | -2.2137 |
| H | -8.593 | -1.9298 | 0.321 | H | -8.0812 | -0.9585 | -2.8305 |
| H | -7.8878 | -3.0901 | -0.8113 | H | -8.7812 | -2.2749 | -1.8807 |
| H | -7.9225 | -3.4479 | 0.93 | H | -9.2398 | -0.5898 | -1.5469 |
| H | 7.5678 | -1.127 | -2.2861 | H | 7.9228 | 1.4687 | -1.5839 |
| H | -4.4311 | 3.4026 | 0.8321 | H | -4.5091 | 0.7854 | 2.7948 |
| H | -2.6851 | 3.5206 | 0.8456 | H | -2.7789 | 0.8002 | 3.056 |
| H | -3.4829 | 2.2982 | 1.8524 | H | -3.5346 | -0.62 | 2.3115 |
| H | -4.2982 | -2.5123 | 0.1869 | H | -6.9348 | -1.3333 | 1.332 |
| H | -5.2952 | -3.7375 | 0.9787 | H | -8.5981 | -0.9885 | 0.887 |
| H | -5.3668 | -3.5151 | -0.7813 | H | -7.93 | -2.612 | 0.621 |
| H | 3.5941 | 0.2646 | 2.5806 | H | 3.6057 | -2.2821 | 1.3666 |
| H | 0.4464 | -0.7534 | 1.5142 | H | 0.598 | -1.6792 | -0.2682 |
| H | -2.5315 | 3.0851 | -1.4537 | H | -2.4809 | 2.6898 | 1.7114 |

**Table S36.** Cartesian Coordinates of conformers **1−6c** and **1−6d** in the gas phase (Å) at the B3LYP/6-31(d,p) level.

| **1−6c** | | | | **1−6d** | | | |
| --- | --- | --- | --- | --- | --- | --- | --- |
| C | -5.8811 | -0.5763 | -0.8324 | C | -6.48 | -1.1481 | -0.1542 |
| C | -6.6033 | 0.0258 | 0.192 | C | -7.2683 | -0.0195 | 0.0416 |
| C | -5.9292 | 0.7983 | 1.136 | C | -6.6731 | 1.2401 | 0.0061 |
| C | -4.5461 | 0.9569 | 1.0488 | C | -5.3016 | 1.3577 | -0.2198 |
| C | -3.8131 | 0.3386 | 0.0221 | C | -4.5014 | 0.218 | -0.4047 |
| C | -4.499 | -0.4269 | -0.9283 | C | -5.1087 | -1.0431 | -0.3784 |
| C | -2.3338 | 0.518 | -0.0493 | C | -3.0358 | 0.3668 | -0.6402 |
| C | -1.517 | -0.3247 | -1.017 | C | -2.1383 | -0.8608 | -0.6153 |
| C | -0.0175 | -0.3235 | -0.6559 | C | -0.6535 | -0.4834 | -0.4387 |
| C | 0.762 | -1.0911 | -1.7327 | C | 0.2058 | -1.7515 | -0.5359 |
| C | 2.2488 | -1.2296 | -1.3862 | C | 1.6892 | -1.4633 | -0.2814 |
| C | 2.5649 | -0.8755 | 0.0745 | C | 1.9365 | -0.0898 | 0.3614 |
| C | 3.9175 | -1.4591 | 0.5825 | C | 3.3191 | 0.0307 | 1.0805 |
| C | 5.1535 | -1.0971 | -0.2743 | C | 4.4799 | -0.3018 | 0.1155 |
| C | 5.6509 | 0.3541 | -0.2382 | C | 5.8792 | -0.192 | 0.7343 |
| C | 4.7936 | 1.3154 | -1.0095 | C | 6.9349 | -0.6486 | -0.2382 |
| C | 4.157 | 2.4154 | -0.562 | C | 7.7399 | 0.1191 | -0.9985 |
| C | 3.3331 | 3.2559 | -1.5047 | C | 8.7744 | -0.5075 | -1.8964 |
| O | -6.4978 | -1.3339 | -1.7839 | O | -7.0188 | -2.4007 | -0.137 |
| O | 1.5165 | -1.4039 | 0.931 | O | 0.903 | 0.1804 | 1.3417 |
| C | 0.2196 | -1.1005 | 0.6578 | C | -0.4034 | 0.0725 | 0.9801 |
| O | -0.6839 | -1.4736 | 1.3989 | O | -1.3006 | 0.36 | 1.7655 |
| C | 4.1451 | -1.1019 | 2.0603 | C | 3.4754 | 1.4333 | 1.6912 |
| C | 4.168 | 2.921 | 0.8539 | C | 7.7006 | 1.6214 | -1.0532 |
| O | -1.8035 | 1.3771 | 0.6616 | O | -2.5828 | 1.4891 | -0.8848 |
| O | -3.9744 | 1.7419 | 2.0146 | O | -4.81 | 2.6357 | -0.2479 |
| O | 0.4742 | 1.0148 | -0.6542 | O | -0.2452 | 0.3843 | -1.4947 |
| O | 3.8169 | -2.9004 | 0.531 | O | 3.3541 | -0.9102 | 2.1647 |
| H | -7.6799 | -0.0887 | 0.271 | H | -8.3368 | -0.0968 | 0.2175 |
| H | -6.4774 | 1.2809 | 1.9414 | H | -7.2739 | 2.1343 | 0.1522 |
| H | -3.9915 | -0.911 | -1.7562 | H | -4.5478 | -1.9584 | -0.5361 |
| H | -1.8925 | -1.354 | -1.0127 | H | -2.4461 | -1.5228 | 0.2018 |
| H | -1.6606 | 0.1112 | -2.0131 | H | -2.2807 | -1.3748 | -1.5737 |
| H | 0.6746 | -0.59 | -2.705 | H | 0.1039 | -2.2152 | -1.5251 |
| H | 0.3388 | -2.0971 | -1.8551 | H | -0.1363 | -2.4924 | 0.1991 |
| H | 2.5434 | -2.2629 | -1.6062 | H | 2.0798 | -2.2667 | 0.3547 |
| H | 2.8399 | -0.598 | -2.0578 | H | 2.2408 | -1.5249 | -1.2272 |
| H | 2.5787 | 0.207 | 0.2219 | H | 1.8917 | 0.6995 | -0.3992 |
| H | 5.9869 | -1.7308 | 0.0636 | H | 4.3648 | -1.333 | -0.2423 |
| H | 4.9916 | -1.4111 | -1.3139 | H | 4.4202 | 0.3522 | -0.7643 |
| H | 5.8191 | 0.6698 | 0.7933 | H | 6.0799 | 0.8291 | 1.0687 |
| H | 6.643 | 0.3754 | -0.7088 | H | 5.9486 | -0.8227 | 1.6287 |
| H | 4.7114 | 1.0757 | -2.0706 | H | 7.0441 | -1.732 | -0.2941 |
| H | 3.3197 | 2.8501 | -2.5217 | H | 8.7797 | -1.6004 | -1.8257 |
| H | 2.2965 | 3.3104 | -1.1566 | H | 8.5808 | -0.243 | -2.9411 |
| H | 3.7368 | 4.2723 | -1.5568 | H | 9.7744 | -0.1526 | -1.6265 |
| H | -7.4501 | -1.3666 | -1.5902 | H | -7.972 | -2.3297 | 0.0415 |
| H | 5.1095 | -1.4893 | 2.4086 | H | 4.3646 | 1.4972 | 2.327 |
| H | 3.387 | -1.5604 | 2.7054 | H | 2.6353 | 1.6803 | 2.3498 |
| H | 4.1233 | -0.0214 | 2.2276 | H | 3.5396 | 2.2018 | 0.9143 |
| H | 4.7905 | 2.3283 | 1.5261 | H | 6.8611 | 2.0534 | -0.5035 |
| H | 4.5472 | 3.948 | 0.8828 | H | 8.6252 | 2.0374 | -0.6402 |
| H | 3.1511 | 2.9176 | 1.2606 | H | 7.6053 | 1.9572 | -2.0915 |
| H | -3.014 | 1.8125 | 1.8189 | H | -3.8516 | 2.5878 | -0.46 |
| H | -0.063 | 1.5096 | 0.0039 | H | -0.842 | 1.1652 | -1.4612 |
| H | 3.015 | -3.1377 | 1.0354 | H | 2.5777 | -0.7166 | 2.7244 |
